# Crosstalk of Eight Types of RNA Modification Regulators Defines Tumor Microenvironments, Cancer Hallmarks, and Prognosis of Lung Adenocarcinoma

**DOI:** 10.1155/2022/1285632

**Published:** 2022-07-11

**Authors:** Shuangshuang Mao, Zuhua Chen, Yingying Wu, Huihua Xiong, Xianglin Yuan

**Affiliations:** Department of Oncology, Tongji Hospital, Tongji Medical College, Huazhong University of Science and Technology, Wuhan, Hubei, China

## Abstract

RNA modification has become an exciting underexplored field in recent years. In lung adenocarcinoma (LUAD), m^6^A was the best characterized and most studied RNA modification, while knowledge about other kinds of RNA modifications in LUAD is limited. In our study, we included a total of 100 RNA modification regulators of eight types of cancer-related RNA modifications (m^6^A, m^1^A, m^5^C, Nm, m^7^G, Ψ, A-to-I, and mcm^5^s^2^U) to systematically profile their specific roles in LUAD. By gene mutation and expression analysis, we identified extensive dysregulations and complicated interactions of 100 RNA modification regulators in LUAD. Based on unsupervised clustering analysis, gene set variation analysis (GSVA), and single-sample gene-set enrichment analysis (ssGSEA), two RNA modification patterns in LUAD were defined to show distinct biological characteristics. The favorable prognostic pattern was enriched with infiltrated immune cells, including activated B cells, CD8 T cells, eosinophil cells, dendritic cells, and natural killer cells, while the unfavorable prognostic pattern was enriched with cancer hallmarks, including hypoxia, epithelial-mesenchymal transition (EMT), angiogenesis, PI3K-AKT-mTOR pathway, MYC pathway, and glycolysis pathway. We also constructed an RNA modification score (RMScore) based on five critical genes (CYP17A1, NTSR1, PITX3, KRT6A, and ANLN) to evaluate the RNA modification status of individual LUAD patients. RMScore was revealed to be related to the infiltrated immune cells and cancer hallmarks and was an independent prognostic factor in the TCGA-LUAD cohort and two external GEO-LUAD cohorts. Our study was the first to comprehensively investigate the dysregulations, crosstalk, and potential prognostic value of eight types of RNA modifications in LUAD. Our results highlighted the significance of eight types of RNA modifications in tumor microenvironments and cancer hallmarks and provided novel prognostic biomarkers and potential therapeutic targets in the management of LUAD patients in the future.

## 1. Introduction

Lung cancer, one of the most prevalent cancers, is the leading killer and represents one of the most challenging health problems worldwide [1, 2]. Lung cancer is divided into two main pathological subtypes, including nonsmall cell lung cancer (NSCLC) and small cell lung cancer (SCLC), which account for about 85% and 15% of all lung cancer cases, respectively [3]. Among all pathological subtypes of NSCLC, lung adenocarcinoma (LUAD) is the most predominant one [3]. Recent studies about the molecular biological characteristics and the tumor microenvironments have revolutionized the management of LUAD patients, leading to a transition from traditional chemotherapy to novel target therapy and immunotherapy, which significantly improved patient outcomes [4, 5]. However, only a small fraction of LUAD patients can benefit from these novel therapeutics, and some patients inevitably suffer from drug resistance, leading to the disappointing survival of some LUAD patients [6]. Thus, further efforts are still needed to excavate the underlying molecular biological mechanisms and potential drug targets to improve the outcomes of LUAD patients.

RNA modification is an important post-transcriptional program, which has become an exciting underexplored field in recent years because of the development of novel technologies. There are over 170 kinds of RNA modifications found in eukaryotes [7], many of which were revealed to show strong connections with various cancers, including LUAD. Among all LUAD-related RNA modifications, m^6^A (N^6^-methyladenosine) is the best characterized one. For example, the m^6^A modification of circIGF2BP3 mediated by METTL3 could increase the level of PD-L1 in NSCLC and facilitate the immune escape of NSCLC by blocking the CD8^+^ T cell response [8]. M^6^A-modified long noncoding RNA (lncRNA) LCAT3 promoted LUAD tumor growth and metastasis by activating the c-MYC pathway [9]. M^6^A eraser ALKBH5 functioned as an antitumor effector in LUAD by reducing the m^6^A modification and downregulating the expression of YAP [10]. Except for m^6^A modification, m^5^C (5-methylcytosin), m^1^A (N^1^-methyladenosine), Nm (2′-O-methylation), and other types of RNA modifications have also been documented to have critical roles in lung cancer or other cancers [11–13]. However, these studies just focused on the function of one regulator with the comprehensive roles, and the interactions between different regulators remain unknown. Recently, a few studies paid attention to one kind of RNA regulator and analyzed the crosstalk of different regulators within one type of RNA modification. Liu et al. constructed a prognostic model based on 13 m^6^A regulators to predict the survival of LUAD patients [14]. Xu et al. analyzed m^6^A-related lncRNA and built a risk model that was an independent prognostic factor and could predict the response of immunotherapy in LUAD [15]. Chen also identified two m^5^C modification patterns in LUAD patients and calculated the m^5^C score, which was related to the tumor microenvironments and LUAD patient outcomes [16]. However, these studies ignored the potential functions of other types of RNA modifications and could not elucidate the relationships and interactions between different types of RNA regulators. Systematically profiling the comprehensive roles and interactions of different kinds of RNA regulators is needed to improve our knowledge about RNA modifications in LUAD, and it might help to reveal novel molecular mechanisms to improve the outcomes of LUAD patients.

Thus, in our research, we systematically profiled a total of 100 RNA regulators of eight types of RNA modifications in LUAD for the first time. We revealed the extensive dysregulations and comprehensive interactions among eight types of RNA modifications and found two LUAD-related RNA modification patterns with different cancer hallmarks, infiltrated immune cells, and different prognoses. We also constructed an RNA modification score (RMScore) based on five RNA modification-related genes, which could predict the survival of LUAD patients, and it was validated to be an independent prognostic factor in different LUAD cohorts. Our study underlined the importance of eight types of RNA modifications in LUAD for the first time and provided potential therapeutic targets for the management of LUAD patients in the future.

## 2. Materials and Methods

### 2.1. LUAD Datasets Curation and Processing

Level-three RNA-seq data, raw count data, gene mutation data, copy number variation (CNV) data, and clinical data of the LUAD cohort were downloaded from the TCGA database. Fragments per kilobase of transcript per million fragments mapped (FPKM) values were log_2_ transformed to represent the mRNA expression levels of genes. Raw count data was used for differential expression gene analysis. Patients lacking age, gender, TNM stage, and survival information were excluded from our study. Genes with FPKM value below 1 in more than half of patients were also excluded from our study.

The microarray datasets of LUAD patients were obtained from the gene expression omnibus (GEO). Log_2_ transformation followed by quantile normalization was used to process microarray data. A total of three GEO-LUAD datasets (GSE40419, GSE41271, and GSE50081) were included in this study, with GSE40419 containing 87 LUAD tumors and 77 adjacent normal tissues, GSE41271 containing 178 tumor samples, and GSE50081 containing 127 tumor samples. Detailed information about TCGA-LUAD and three GEO-LUAD datasets were summarized in Table S1. The CLIP-seq data of TRM10A, FTO, ZCCHC4, YTHDC2, METTL3, and YTHDF3 was obtained from the GEO database with the GEO accession numbers GSE146207, GSE102336, GSE98085, GSE191170, and GSE86214, respectively.

Because of the huge number of RNA regulators and their physiological effects in eukaryotes, we limited our study to cancer-related RNA regulators that have been widely documented to show critical roles in LUAD or other cancers [11–13]. Finally, a total of 100 RNA regulators that belong to 8 kinds of RNA modifications (m^6^A, m^1^A, m^5^C, Nm, m^7^G (7-methylguanosine), Ψ (uridine-to-pseudouridine), A-to-I (adenosine-to-inosine transition), and mcm^5^s^2^U (5-methoxycarbonylmethyl-2-thiouridine)) were included in our study. The 100 RNA regulators were listed in Table S2.

### 2.2. Expression and Prognostic Analysis of 100 RNA Regulators

The mutation pattern of 100 RNA regulators in 8 types of RNA modifications was analyzed by the Maftools R package. Paired *t*-test and unpaired *t*-test were used in the TCGA-LUAD cohort (including 56 paired LUAD tumors and adjacent normal tissues) and GSE40419 dataset (including 87 LUAD tumors and 77 normal tissues), respectively, to find dysregulated RNA regulators. Detailed information about the 56 tumor samples and matched normal tissues of the TCGA-LUAD cohort was summarized in Table S3. RNA regulators with adjusted *p* value < 0.05 and log_2_ (fold change) ≥ 1 or log_2_ (fold change) ≤ −1 were identified as differentially expressed genes.

Univariate cox regression analysis was performed to find survival-associated RNA regulators in 8 types of RNA modifications. RNA regulators with adjusted *p* value < 0.05 in univariate cox regression analysis together with clinical characteristics (age, gender, and TNM stage) were included in multivariate cox regression analysis to find independent prognostic regulators in LUAD patients. Forest plot and Kaplan–Meier curves were used to show the prognostic value of RNA regulators. The crosstalk and correlation within one kind of RNA regulator and among different kinds of RNA regulators were analyzed using the Spearman correlation algorithm. Correlations with adjusted *p* value < 0.05 and |correlation coefficient > 0.5 were shown using Cytoscape software 3.8.0.

### 2.3. Clustering Pattern Analysis of 100 RNA Regulators

The Consensus-Clusterplus R package was utilized to conduct an unsupervised clustering analysis in LUAD patients based on the expression of 100 RNA regulators. To enhance the stability of clusters, they were repeated a total of 1000 times.

To clarify the biological significance of different clusters determined by 100 RNA regulators, the gene set variation analysis (GSVA) was performed using the GSVA R package. The hallmark gene set “h.all.v7.4.symbols.gmt” was downloaded from MSigDB datasets and was used for GSVA analysis. The single-sample gene-set enrichment analysis (ssGSEA) method was used to calculate the quantity of infiltrated immune cells in different clusters of LUAD patients. The immune scores and stromal scores of LUAD patients in different clusters were compared by the estimate R package.

### 2.4. RNA Modification Score (RMScore) Construction

To evaluate the RNA modification status for an individual patient, we construct the RMScore following the steps below.

1. Differentially expressed genes (DEGs) between different RNA modification clusters were identified using the DESeq2 R package. Raw count data was used for DEG analysis. Genes with adjusted *p* value < 0.05 and log2(fold change) ≥ 1 or log2(fold change) ≤ −1 were identified as DEGs.
2. The univariate cox regression analysis was performed to find survival-associated DEGs.
3. Rbsurv R package was utilized to perform robust likelihood-based survival analysis and further select survival-associated DEGs, which made the selected target genes more reliable.

Multivariate cox regression analysis was conducted to construct the final prognostic model. Genes with a *p* value < 0.05 in multivariate cox regression analysis were used to construct the RMScore using the following formula: RMScore = ∑_coefi _*∗*_ genei_.

### 2.5. Clinical Significance Analysis of RMScore

To illuminate the clinical significance of RMScore constructed in our study, we divide LUAD patients into two groups based on the median value of RMScore. GSVA and ssGSEA methods were used to compare tumor-specific hallmark pathways and infiltrated immune cells in the RMScore-high group and RMScore-low group. Spearman correlation analysis was performed to reveal the correlations between the individual RNA modification regulator and the RMScore. Kaplan–Meier curves, together with ROC curves, were plotted to show the prognostic value in the training cohort (TCGA dataset) and the validation cohorts (GEO datasets). All analyses were performed on RStudio 1.4.1106. The protocol of this study was approved by the ethics committee of our institute and the study was conducted in accordance with the Declaration of Helsinki. Considering that only publicly available data were used in this study, the requirement for informed consent from patients was waived by the ethics committee.^*∗*^*p* < 0.05; ^*∗∗*^*p* < 0.01; ^*∗∗∗*^*p* < 0.001; ^*∗∗∗∗*^*p* < 0.0001; ns, not significant.

## 3. Results

### 3.1. Mutation and Dysregulation of Eight Types of RNA Regulators in LUAD

According to the published data until now, a total of 100 RNA modification regulators that have been documented to be related to at least one kind of cancer have been included in our study. 100 RNA regulators belong to eight types of RNA modifications, including 33 regulators in m^6^A, 16 regulators in m^5^C, 15 regulators in Nm, 11 regulators in Ψ, 9 regulators in m^1^A, 6 regulators in m^7^G, 5 regulators in A-to-I, and 5 regulators in mcm^5^s^2^U (Table S2).

**Figure 1:**
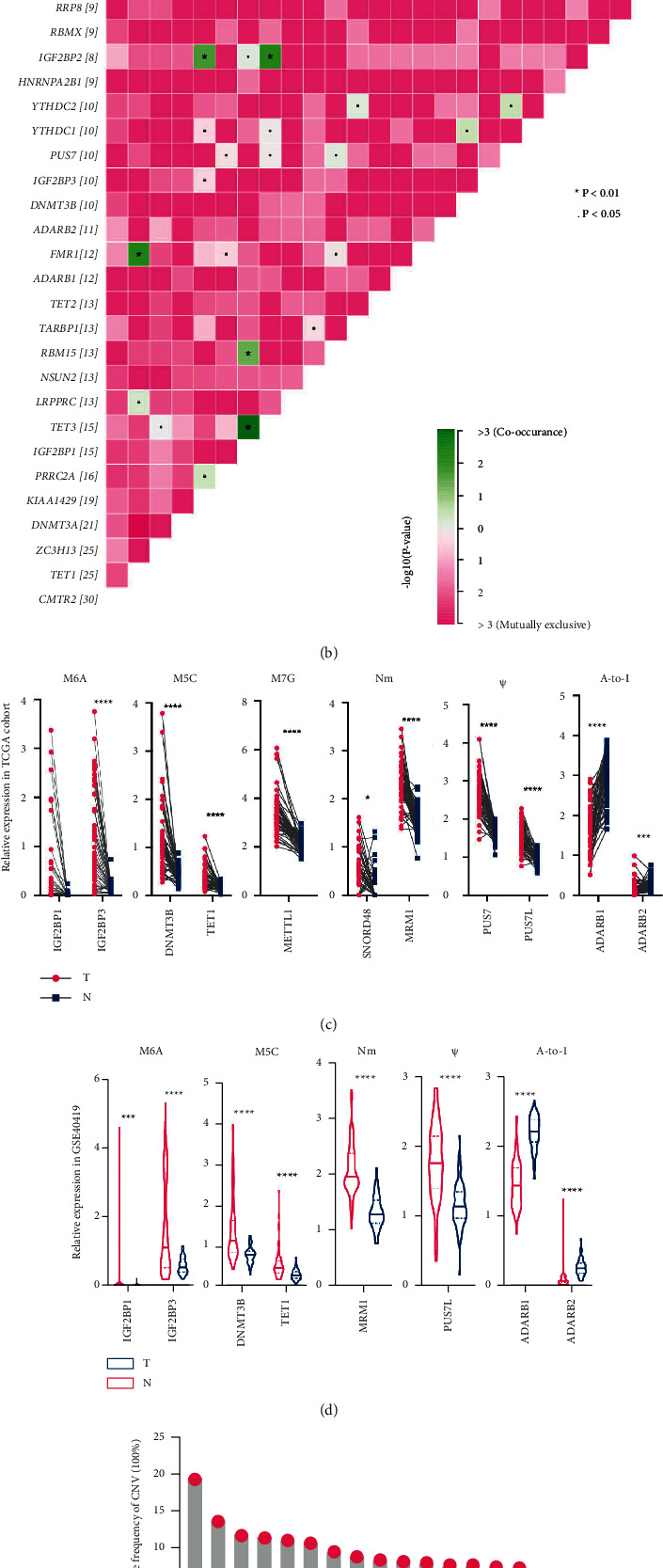
The mutation and dysregulation of eight types of RNA modification regulators in LUAD. (a) The mutation spectrum of RNA regulators with mutation rates ≥2% in the TCGA-LUAD cohort, with each column representing one patient and the percentage on the right side representing the corresponding gene mutation rate. (b) The crosstalk of gene mutations with mutation rates ≥2% in the TCGA-LUAD cohort. (c) The relative expressions of differentially expressed RNA regulators in TCGA-LUAD tumor and paired normal lung tissues. (d) The relative expressions of differentially expressed RNA regulators in the GSE40419 datasets. (e) The frequency of CNV of top 15 RNA regulators with the gain of CNV in the TCGA-LUAD cohort. (f) The frequency of CNV of top 15 RNA regulators with the loss of CNV in the TCGA-LUAD cohort. (g) The frequency of CNV of differentially expressed RNA regulators in the TCGA-LUAD cohort. T, tumor; N, normal.

To evaluate the genetic alternations of different types of RNA regulators in LUAD, we accessed the mutation profiles of eight types of RNA regulators in the TCGA-LUAD cohort, respectively. As shown in Figure S1, RNA regulators in m^6^A showed the highest mutation rate (33.16%, Figure S1A), followed by RNA regulators in m^5^C (20.63%, Figure S1B), Nm (12.17%, Figure S1C), Ψ (6.53%, Figure S1D), m^1^A (5.64%, Figure S1E), A-to-I (5.47%, Figure S1F), and m^7^G (3.53%, Figure S1G). RNA regulators in mcm^5^s^2^U showed the lowest mutation rate (1.41%, Figure S1H). We also compared the mutations of RNA regulators in diverse RNA modifications and showed the mutation profiles of genes with mutation rates ≥ 2% (Figure 1(a)). As a result, about a quarter of 100 RNA regulators in eight types of RNA modifications have a mutation rate ≥ 2%. Nm writer CMTR2 showed the highest mutation rate (5%) followed by m^6^A writer ZC3H13, m^5^C writer DNMT3A, and m^5^C eraser TET1 (4%) (Figure 1(a)). M^6^A writer KIAA1429, m^6^A reader PRRC2A, IGF2BP1, and m^5^C eraser TET3 also showed pretty high mutations with a mutation rate of 3% in LUAD patients (Figure 1(a)). Genetic interaction analysis identified that the mutations of m^5^C eraser TET3 were positively correlated with the mutations of m^6^A reader IGF2BP1 and IGF2BP2. The mutations of m^6^A reader IGF2BP2 and FMR1 were positively correlated with the mutations of m^6^A writer KIAA1429 and m^5^C eraser TET1, respectively (Figure 1(b)), which underlined the mutual exclusion or co-occurrence of mutations of different types of RNA regulators.

To evaluate the dysregulation of 100 RNA regulators, we conducted differentially expressed gene analysis in 56 TCGA-LUAD tumors and 56 paired normal samples, as well as in GSE40419 datasets, which contained 87 LUAD tumors and 77 adjacent normal lung tissues. RNA regulators with adjusted *p* value < 0.05 and log_2_(fold change) ≥ 1 or log_2_ (fold change) ≤ −1 were considered differentially expressed genes. As a result, IGF2BP1 and IGF2BP3 of m^6^A, DNMT3B, and TET1 of m^5^C, METTL1 of m^7^G, SNORD48 and MRM1 of Nm, and PUS7 and PUS7L of Ψ were found to be significantly upregulated in TCGA-LUAD tumors, while ADARB1 and ADARB2 of A-to-I were found to be significantly downregulated (Figure 1(c)). Consistent with TCGA-LUAD cohorts, differentially expressed gene analysis in GSE40419 found the same results, except for METTL1, SNORD48, and PUS7, which were not detected in GSE40419 datasets (Figure 1(d)). We also analyzed the copy number variation (CNV) of 100 RNA regulators in TCGA-LUAD cohorts. The top 15 RNA regulators with a gain of CNV or loss of CNV are shown in Figures 1(e) and 1(f). The writer of A-to-I ADAR and writer of Nm HENMT1 showed the highest frequency of gain of CNV and loss of CNV, respectively (Figures 1(e) and 1(f)). As for the differentially expressed RNA regulators, all of the upregulated genes showed a gain of CNV, which might explain their upregulation partially, while the downregulation of ADARB1 and ADARB2 might not be correlated with their alternations of CNV (Figure 1(g)).

### 3.2. Prognostic Value and Crosstalk of Eight Types of RNA Regulators in LUAD

**Figure 2:**
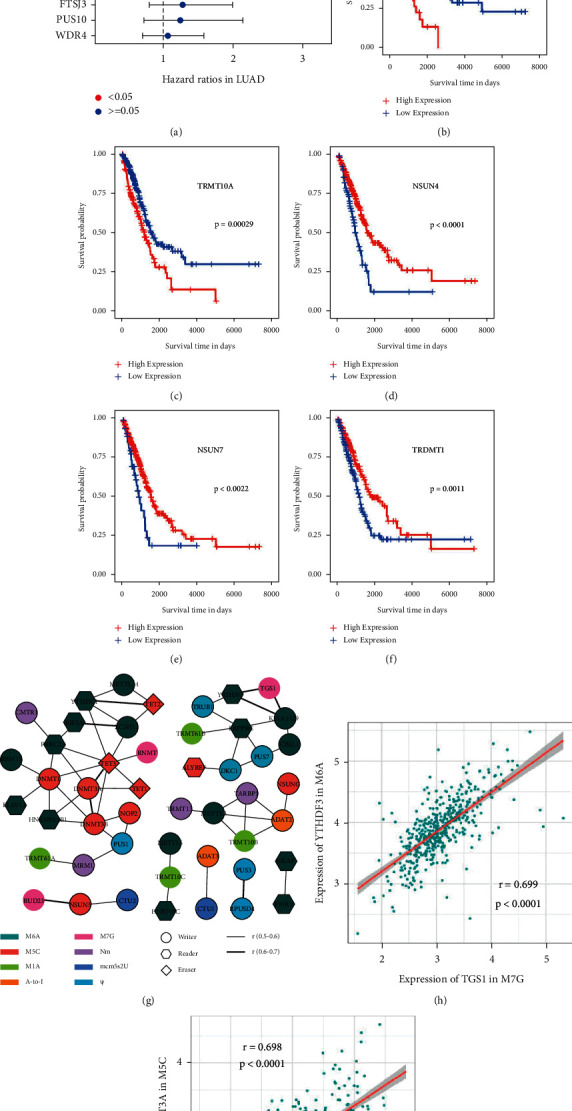
The prognostic value and interactions of eight types of RNA regulators in LUAD. (a) Multivariate cox regression analysis identified the TNM stage, IGF2BP1, TRMT10A, NSUN4, NSUN7, and TRDMT1 as independent prognostic factors in LUAD. The dotted horizontal line represents the hazard ratios and the 95% confidence interval of each gene, with the color of dot point representing the statical significance. (b)–(f) Kaplan–Meier curves showed the overall survival of LUAD patients in IGF2BP1-low or IGF2BP1-high group (b), TRMT10A-low or TRMT10A-high group (c), NSUN4-low or NSUN4-high group (d), NSUN7-low or NSUN7-high group, (e) and TRDMT1-low or TRDMT1-high group (f). (g) The interactions of eight types of RNA regulators with *p* value <0.05 and correlation coefficient ≥0.5 in LUAD. The color of the node represents RNA modification type of each gene and the shape of the node represents the working pattern of each regulator. The lines between the gene nodes represent their correlation coefficients. (h) The level of YTHDF3 of m^6^A was positively correlated with the level of TGS1 of m^7^G in LUAD. (i) The level of DNMT3A of m^5^C was positively correlated with the level of TET3 of m^5^C in LUAD.

To evaluate the prognostic value of 100 RNA regulators in LUAD, we conducted a univariate cox regression analysis in TCGA-LUAD cohorts to find survival-associated RNA regulators. As shown in Figure S2, a total of 18 RNA regulators were identified to be significantly related to the overall survival (OS) of LUAD patients, including METTL5, IGF2BP3, IGF2BP2, IGF2BP1, HNPNPC, HNRNPA2B1, and G3BP1, in m^6^A (Figure S2A), TRMT10B, TRMT10A, and ALKBH3 in m^1^A (Figure S2B), WDR4 in m^7^G (Figure S2C), TRDMT1, NSUN7, NSUN4, NOP2, ALYREF in m^5^C (Figure S2D), FTSJ3 in Nm (Figure S2E), and PUS10 in Ψ (Figure S2F), while RNA regulators in A-to-I and mcm^5^s^2^U were found to have no correlations with the prognosis of LUAD patients (Figures S2G and S2H). To further determine the independent prognostic value of RNA regulators, we performed a multivariate cox regression analysis on the above 18 regulators and patient clinical characteristics. As a result, TRMT10A, IGF2BP1, and patient TNM stage were identified as the independent unfavorable prognostic factors, while NSUN4, NSUN7, and TRDMT1 were identified as the independent favorable prognostic factors in LUAD patients (Figure 2(a), Table S4). Kaplan–Meier curves also showed that LUAD patients with a low expression of IGF2BP1 and TRMT10A or with a high expression of NSUN4, NSUN7, and TRDMT1 have longer overall survival (Figures 2(b)–2(f)). Furthermore, we used another GEO dataset (GSE50081) as an external validation cohort to further validate the prognostic value of RNA regulators in LUAD. As shown in Figure S3, the prognosis of LUAD patients with a low expression of IGF2BP1 or TRMT10A was significantly better than those with a high expression of IGF2BP1 or TRMT10A (Figures S3A and 3(b)). The prognosis of LUAD patients with a low expression of NSUN4, NSUN7, or TRDMT1 was significantly poorer than those with a high expression of NSUN4, NSUN7, or TRDMT1 (Figures S3C and 3(e)).

We also explored the relationships among eight types of RNA regulators in LUAD. As a result, extensive interactions were found within one kind of RNA regulator and between different kinds of RNA regulators (Table S5). Correlations with adjusted *p* value < 0.05 and *r* ≥ 0.5 or *r* ≤ −0.5 were shown in Figure 2(g) (no correlations with *r* ≤ −0.5 were found). We can see that the RNA regulators of m^6^A and m^5^C showed positive correlations with each other. The RNA regulators of m^6^A also showed significant correlations with the RNA regulators of Ψ, m^1^A, m^7^G, and Nm (Figure 2(g)). Besides, the RNA regulators of m^5^C, Ψ, mcm^5^s^2^U, and A-to-I were also found to have interactions with each other (Figure 2(g)). Correlations with the top 10 correlation coefficients were shown in Figures 2(h) and 2(i) and Figure S4. The YTHDF3 of m^6^A showed the strongest positive correlation with the TGS1 of m^7^G (Figure 2(h)), followed by the DNMT3A and TET3 of m^5^C (Figure 2(i)), KIAA1429 of m^6^A, TGS1 of m^7^G (Figure S4A), KIAA1429 and YTHDF3 of m^6^A (Figure S4B), NOP2 of m^5^C, PUS1 of Ψ (Figure S4C), PUS3 and RPUSD4 of Ψ (Figure S4D), YTHDC1 of m^6^A, TET2 of m^5^C (Figure S4E), DNMT3A and DNMT3B of m^5^C (Figure S4F), NSUN5 of m^5^C, BUD23 of m^7^G (Figure S4G), CBLL1 of m^6^A, and PUS7 of Ψ (Figure S4H). All of the above implied that eight types of RNA regulators might interact with each other to play critical roles in LUAD.

### 3.3. Distinct RNA Modification Patterns Showed Different Cancer Hallmarks and Tumor Microenvironments

**Figure 3:**
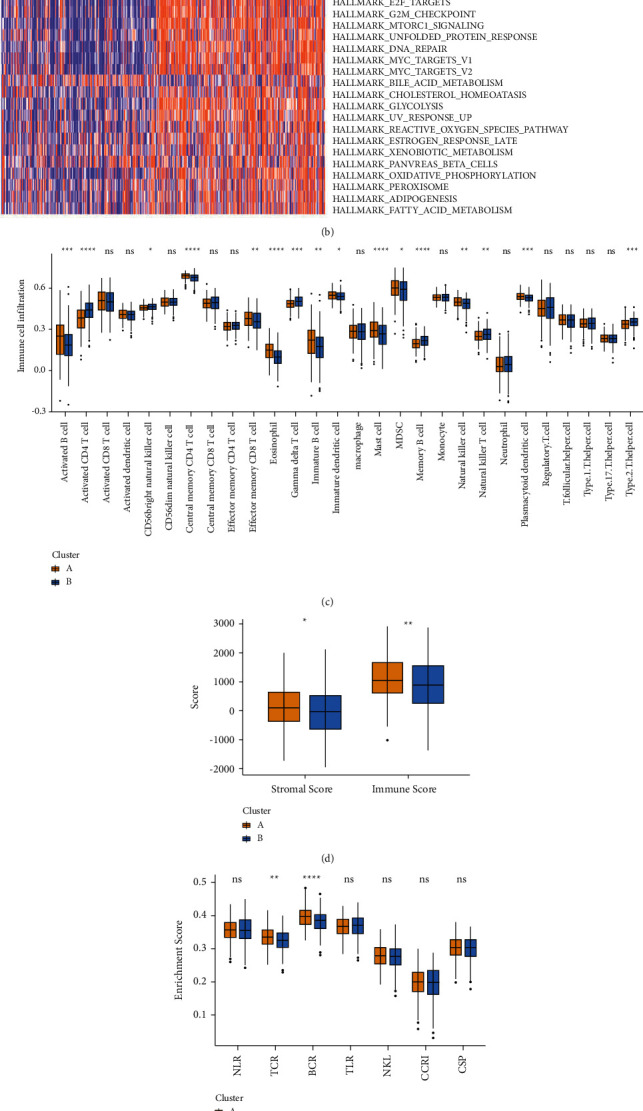
Two RNA modification patterns of LUAD showed distinct cancer hallmarks and tumor microenvironments. (a) Kaplan–Meier curves showed the different overall survival of LUAD patients in two RNA modification clusters. (b) The heatmap revealed that most cancer hallmarks were enriched in LUAD patients in the RNA modification cluster (b). (c) The box plot showed that immune cells infiltrated in tumor microenvironments were distinct in two RNA modification clusters. (d) The box plot showed the immune score and stromal score of LUAD patients in two RNA modification clusters. (e) The box plot showed the enrichment score of immune-related pathways in two RNA modification clusters. (f) The correlations between infiltrated immunes cells and RNA modification regulators. NLR, nod-like receptor signaling pathway; TCR, T cell receptor signaling pathway; BCR, B cell receptor signaling pathway; TLR, toll-like receptor signaling pathway; NKL, natural killer cell-mediated cytotoxicity; CCRI, cytokine-cytokine receptor interaction; CSP, chemokine signaling pathway.

To excavate the RNA modification patterns of LUAD, an unsupervised clustering analysis was performed in TCGA-LUAD patients based on 100 RNA regulators. As a result, two different RNA modification patterns were defined, with Cluster A having 226 LUAD patients and Cluster B having 245 LUAD patients (Table S6). We compared the mutation and expression patterns of 100 RNA regulators in Cluster A and Cluster B. As shown in Figures S5A and S5B, the mutation rate of RNA regulators in Cluster B was significantly higher than that in Cluster A. RNA regulators with mutation rates > 2% were also different in the two clusters. TET1 showed the highest mutation rate in Cluster A, followed by ZC3H13, CMTR2, and HNRNPA2B1 (Figure S5A). In Cluster B, CMTR2 showed the highest mutation rate, followed by ZC3H13, KIAA1429, and DNMT3A (Figure S5B). The expression patterns of 100 RNA regulators in two Clusters were also significantly different (Figure S5C). The prognostic analysis showed that LUAD patients in Cluster A had a longer OS than those in Cluster B (Figure 3(a)). To further determine the underlying biological characteristics of two different RNA modification clusters, GSVA and ssGSEA enrichment analyses were conducted to analyze cancer hallmarks and infiltrated immune cells in different RNA modification clusters, respectively. As shown in Figure 3(b), most cancer hallmarks, including hypoxia, epithelial–mesenchymal transition (EMT), angiogenesis, TNF*α* pathway, PI3K-AKT-mTOR pathway, E2F targets, G2M checkpoint, MYC pathway, and glycolysis pathway were enriched in Cluster B, which had a poor prognosis. However, there were more activated B cells, memory CD4 T cells, effector memory CD8 T cells, eosinophil cells, immature B cells, dendritic cells, and natural killer cells infiltrated in Cluster A, which has a favorable prognosis (Figure 3(c)). Besides, the immune score and stromal score were higher in Cluster A than in Cluster B (Figure 3(d)), and the enrichment scores of the T cell receptor (TCR) and B cell receptor (BCR) were also higher in Cluster A (Figure 3(e)), which together demonstrated that the longer OS of Cluster A might attribute to the tumor microenvironment (TME)-infiltrated immune cells, while the poor prognosis of Cluster B might be associated with the tumorigenesis of cancer hallmarks.

Many studies have documented that RNA regulators could affect the infiltration of TME-related immune cells. To further determine immune cell-related RNA regulators of eight types of RNA modifications in LUAD, Spearman correlation analysis was performed between 100 RNA regulators and 28 kinds of immune cells. Correlations with adjusted *p* value < 0.05 and *r* ≥ 0.3 or *r* ≤ −0.3 were shown in Figure 3(f). Seven of the eight types of RNA regulators showed significant correlations with TME-infiltrated immune cells, except for mcm^5^s^2^U. Among all immune cell-related RNA regulators, most RNA regulators of m^6^A, m^5^C, m^1^A, m^7^G, Nm, and Ψ showed negative correlations with infiltrated immune cells, except for WTAP, CBLL1, IGF2BP3, G3BP1, and G3BP2 of m^6^A and YBX1 of m^5^C. However, ADARB1 and ADARB2, the two RNA regulators of A-to-I, showed positive correlations with the infiltrated immune cells (Figure 3(f)). These results demonstrated that RNA regulators defined distinct RNA modification patterns in LUAD by affecting cancer hallmarks and immune microenvironments.

To further validate the RNA modification patterns in patients with LUAD, we performed an unsupervised clustering analysis in an external validation dataset (GSE41271). As shown in Figure S6, LUAD patients in the GSE41271 dataset were also divided into two clusters, with Cluster A showing a longer overall survival than Cluster B, although the *p* value was not statistically significant (Figure S6A). GSVA and ssGSEA enrichment analysis found that most cancer hallmarks were enriched in LUAD patients in Cluster B, while most immune cells were enriched in LUAD patients in Cluster A. These results validated the existence of two RNA modification patterns in LUAD and suggested that different RNA modification patterns might be closely related to cancer hallmarks and immune microenvironments.

As we collected the 100 cancer-related RNA modification regulators based on LUAD and other cancers, we further excavated whether the modification patterns existed in other cancers. Interestingly, we found similar RNA modification patterns in breast cancer patients (BRCA) in the TCGA dataset. A total of 1163 BRCA patients were divided into two clusters based on the 100 RNA regulators and patients in Cluster A, which showed a better prognosis than Cluster B (Figure S7A). GSVA and ssGSEA analysis showed that most cancer hallmarks, including TGF-*β*signaling pathway, KRAS signaling pathway, hypoxia, EMT, and androgen response pathway, were enriched in BRCA patients in Cluster B (Figure S7B), while most immune cells, including activated B cells, activated CD4 and CD8 cells, dendritic cells, macrophage, and monocytes were enriched in BRCA patients in Cluster A (Figure S7C). However, different results were obtained in patients with colon cancer (COAD). Although COAD patients in the TCGA dataset were also divided into two clusters based on the expression of 100 RNA regulators (Figure S7D), the prognosis of patients in the two clusters was not statistically significant (Figure S7E). All of the above implied that the eight kinds of RNA regulators might have defined the RNA modification patterns in LUAD and other cancers, however, the specific functions of RNA modifications might be different in different cancers, which need to be further excavated in the future.

### 3.4. Construction of RNA Modification Score in LUAD

To further evaluate the RNA modification pattern and predict the prognosis of an individual patient with LUAD, we constructed the RNA modification score (RMScore) using differential expression analysis followed by a series of survival analyses in the TCGA-LUAD cohort. The differential expression analysis between RNA modification Cluster A and Cluster B identified a total of 1348 differentially expressed genes (DEGs), and 229 DEGs were proved to be associated with the OS of LUAD patients in a univariate Cox regression analysis (Table S7). To investigate the potential correlations between the survival-associated DEGs and RNA regulators, we evaluate several publicly available CLIP-seq data in GEO datasets (GSE146207, GSE146207, GSE102336, GSE98085, GSE191170, and GSE86214) to find whether the DEGs were bonded and regulated by RNA regulators. As we expected, we found that more than half of DEGs were determined to be bonded and regulated byRNA regulators, including m1A regulator TRM10 A and m6A regulator FTO, ZCCHC4, YTHDC2, METTL3, and YTHDF3 in the CLIP-seq data. Besides, one DEG may be regulated by more than one regulator in the CLIP-seq data, and the detailed regulation network is listed in Table S8.

Next, the robust likelihood-based survival analysis followed by the multivariate Cox regression analysis was performed on the 229 survival-associated DEGs to construct the RMScore. Finally, five genes, including CYP17A1, NTSR1, PITX3, KRT6A, and ANLN, were identified to be the independent prognostic factors in LUAD patients and were used to construct the RMScore by multiplying their respective coefficients (RMScore = coef_1_ ∗ CYP17A1 + coef_2_  ∗  NTSR1  +  coef_3_  ∗  PITX3  +  coef_4_  ∗  KRT6A  +  coef_5_ ∗ ANLN) (Table S9). Among the five RMScore genes, four (ANLN, KRT6A, PITX3, and NTSR1) were identified to be upregulated, while CYP17A1 was identified to be downregulated in TCGA-LUAD tumor tissues compared with paired normal tissues (Figures S8A–S8E). Kaplan–Meier curves also showed that the high expression of ANLN, KRT6A, PITX3, and NTSR1 and the low expression of CYP17A1 were related to the poor survival of LUAD patients (Figures S8F–S8J).

**Figure 4:**
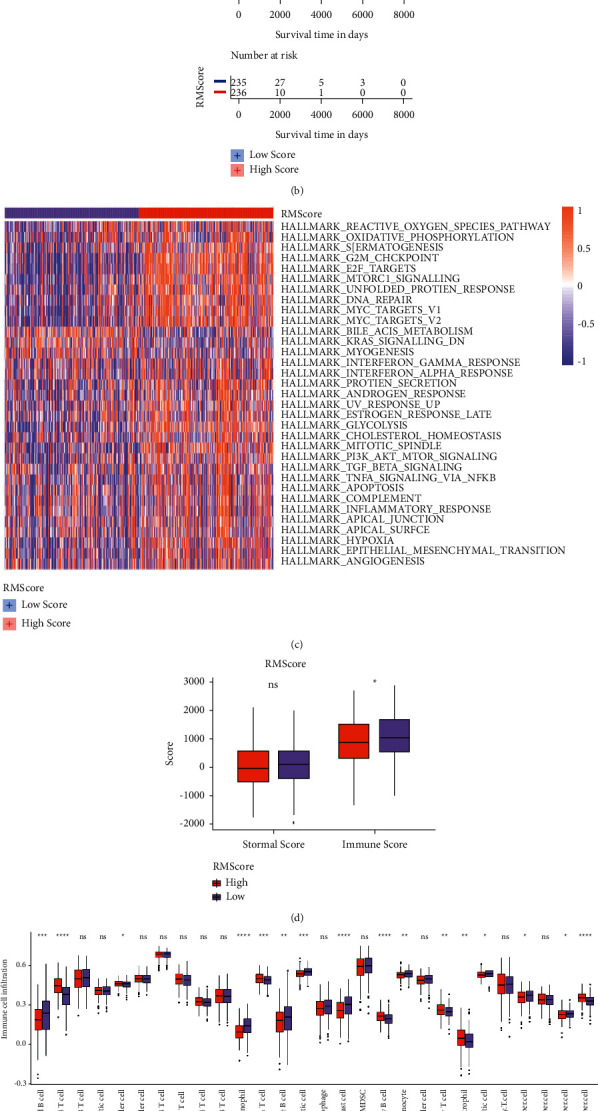
RNA modification score (RMScore) was closely related to cancer hallmarks and tumor microenvironments. (a) The heatmap showed RMScore, RMScore-related genes, RNA modification pattern, and the clinical characteristics of each LUAD patient, with each column representing individual patient. (b) Kaplan–Meier curves showed that the overall survival of patients in the RMScore-low group was longer than those in the RMScore-high group. (c) The heatmap revealed that most cancer hallmarks were enriched in LUAD patients in the RMScore-high group. (d) The box plot showed that the immune score and stromal score of LUAD patients in the RMScore-low group was higher than that in the RMScore-high group. (e) The box plot showed that the immune cells infiltrated in tumor microenvironments were distinct in two RMScore groups. (f) The correlations between RMScore, RMScore-related genes, and RNA modification regulators.

Then, we divided LUAD patients into high and low RMScore groups based on the median of RMScore. The clinical characteristics and expressions of five RMScore genes were shown in Figure 4(a). Most patients in RNA modification Cluster A were identified as RMScore-low group (Figure 4(a), Table S6), and Kaplan–Meier curves showed that patients in the RMScore-low group also had a significant longer OS than those in the RMScore-high group (Figure 4(b)), which demonstrated that two distinct RNA modification patterns defined by eight types of RNA regulators do exist and was closely related to the prognosis of LUAD patients. GSVA analysis identified that most cancer hallmarks, including G2M checkpoint, E2F targets, DNA repair, MYC pathway, glycolysis pathway, PI3K-AKT-mTOR pathway, EMT, hypoxia, and angiogenesis were significantly enriched in the RMScore-high group (Figure 4(c)), which was similar to that in RNA modification Cluster B. However, the immune score in the RMScore-high group was significantly lower than that in the RMScore-low group (Figure 4(d)), which implied that the RMScore-low group was more related to TME-associated immune cells. Further analysis verified that there were more infiltrated B cells, eosinophil cells, dendritic cells, and monocytes in the RMScore-low group compared with that in the RMScore-high group (Figure 4(e)). It could be an important factor for the better prognosis of LUAD patients in the RMScore-low group.

To better understand the crosstalk between RNA regulators and RMScore, we conducted the Spearman correlation analysis between 100 RNA regulators and the RMScore together with five RMScore genes. Correlations with adjusted *p* value < 0.05 and *r* ≥ 0.3 or *r* ≤ −0.3 were shown in Figure 4(f). As a result, RNA regulators in the six types of RNA modifications, including m^6^A, m^5^C, m^1^A, Nm, m^7^G, and Ψ were revealed to be closely related to the RMScore, among which all regulators in m^6^A, Nm, and m^7^G were positively associated with the RMScore, while TRDMT1 and NSUN7 of m^5^C, TRMB10 B and RRP8 of m^1^A, and PUS10 of Ψ were negatively associated with the RMScore. The RMScore gene ANLN was also positively associated with almost all RNA regulators, except for TRDMT1 and NSUN7 of m^5^C, TRMB10 B and RRP8 of m^1^A, and PUS10 of Ψ, while the RMScore gene CYP17A1 was negatively associated with most RNA regulators.

### 3.5. RNA Modification Score Was an Independent Prognostic Factor in LUAD

**Figure 5:**
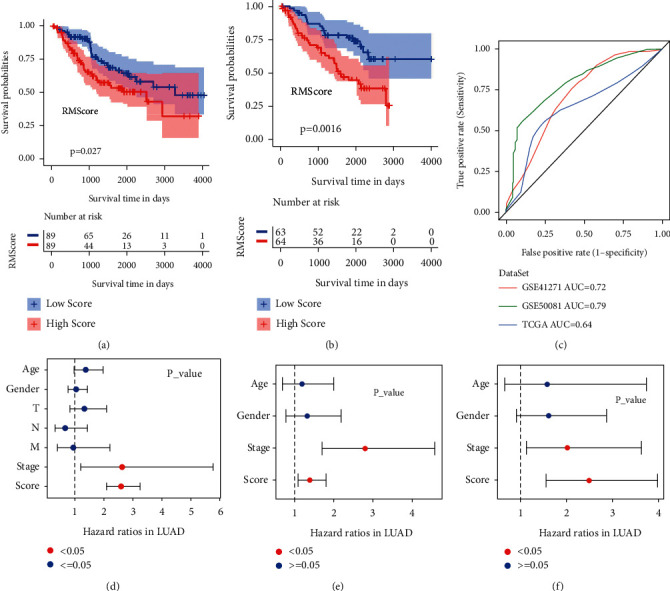
RMScore was an independent prognostic factor in LUAD. (a)-(b). Kaplan–Meier curves showed significantly different overall survival of LUAD patients in different RMScore groups in the GSE41271 dataset (a) and GSE50081 dataset (b). (c) The ROC curves of RMScore in the TCGA-LUAD dataset, GSE41271 dataset, and GSE50081 dataset. (d)–(f) The forest plot showed that RMScore was an independent prognostic factor in the LUAD dataset (d), GSE41271 dataset, (e) and GSE50081 dataset (f).

To further verify the clinical significance of the RMScore, we used the two GEO cohorts of LUAD patients as validation datasets. The RMScore was calculated based on the expression of five RMScore genes in two validation cohorts. Kaplan–Meier curves demonstrated that patients in the RMScore-low group also had a longer OS than patients in the RMScore-high group (Figures 5(a) and 5(b)). Receiver operator characteristic (ROC) curves showed that the AUCs of ROC were 0.64, 0.72, and 0.79 in TCGA and two GEO datasets, respectively (Figure 5(c)). The multivariate cox regression analysis also showed that the RMScore was an independent prognostic factor in TCGA and two GEO datasets (Figures 5(d)–5(f)). All the above underlined the clinical significance of RMScore, and RMScore might be a potential prognostic factor in LUAD patients.

## 4. Discussion

RNA modification is a novel frontier of RNA epitranscriptomics, which is characterized by abundant chemical modifications in different kinds of RNAs. Recently, increasing evidence has suggested that RNA modifications play a critical role in cancer and might serve as novel drug targets in the management of cancer patients. However, studies about RNA modifications in LUAD are mainly limited to a few RNA modification regulators, especially m^6^A-related regulators, with the roles of other kinds of RNA modifications, and their crosstalk with each other remains unknown.

In our study, a total of 100 RNA modification regulators of eight types of RNA modifications (including m^6^A, m^5^C, Nm, Ψ, m^1^A, m^7^G, A-to-I, and mcm^5^s^2^U) were included because of their potential cancer-related functions, which were reported by other pieces of research before. Among all cancer-related RNA modifications, m^6^A is the most important one. m^6^A is methylation at the N^6^ position of the adenine of RNA and is widely documented to regulate the expressions of many oncogenes or tumor suppressor genes to affect the pathological characteristics of various tumor cells or tumor microenvironments [17, 18]. M^5^C is methylation at position 5 of cytidine residues, which are abundant on DNA, mRNA, and ncRNAs [19]. Some writers of m^5^C were revealed to promote the progression of lung cancer, prostate cancer, and oesophageal squamous cell carcinoma by targeting rRNA or ncRNAs [11, 20]. Nm, also called 2′-O-methylation, is the methylation of the 2′ -hydroxyl of ribonucleotides, which is a highly conserved RNA modification in eukaryotes [21], and it was determined to affect the tumorigenesis of colorectal cancer by regulating rRNA [22]. Ψ is the transition from uridine to pseudouridine [23], and A-to-I is the conversion from adenine to inosine [24]. M^1^A is the methylation of adenosine at position 1, m^7^G is the methylation of guanosine at position 7 [25], and mcm^5^s^2^U is an important post-transcriptional modification of U34 on specific tRNAs [26]. The regulators of Ψ, m^1^A, m^7^G, A-to-I, and mcm^5^s^2^U have also been reported to be dysregulated to function as oncogenes or tumor suppressors in various tumors, including lung cancer, prostate cancer, hepatocellular cancer, breast cancer, melanoma, and glioblastoma [11]. However, how these RNA modifications interact with each other in LUAD, what roles they play exactly in LUAD, and whether they can serve as prognostic biomarkers to affect the prognosis of LUAD patients are still obscure.

In our study, we firstly profiled the mutation and expression of 100 RNA modification regulators of eight types of RNA modifications in the TCGA-LUAD cohort. At the genetic level, global mutations were identified across eight types of RNA regulators, with CMTR2 of Nm showing the highest mutation rate (5%), followed by ZC3H13 of m^6^A, DNMT3A, and TET1 of m^5^C. At the transcriptional level, the dysregulations of eight types of RNA modification regulators were also identified, and some were revealed to show significant connections with the survival of LUAD patients. The multivariate Cox regression analysis determined a few of the independent prognostic regulators in LUAD, including IGF2BP1of m^6^A, TRMT10A of m^1^A, and TRDMT1, NSUN7, and NSUN4 of m^5^C. Moreover, the complicated interactions among eight kinds of RNA modifications have also been revealed, and most RNA regulators showed positive correlations with each other. All the above implied that eight types of RNA modifications might interact with each other to affect the pathophysiological characteristics of LUAD.

We also identified two RNA modification patterns in LUAD and constructed the RMScore to evaluate the modification status and prognosis of individual patients. Patients in Cluster A or RMScore-low group showed a significantly longer overall survival than those in Cluster B or RMScore-high group. Most cancer hallmarks, such as hypoxia, EMT, angiogenesis, TNF*α* pathway, PI3K-AKT-mTOR pathway, MYC pathway, and glycolysis pathway were enriched in Cluster B and RMScore-high group, while more infiltrated immune cells were enriched in Cluster A and RMScore-low group, which might account for the different prognosis. Previous studies have also discovered different RNA modification patterns in cancer, including LUAD. For example, Huang et al. identified three m^6^A modification patterns with different tumor microenvironments and biological behaviors and constructed the m^6^A scoring system to predict the prognosis of patients with hepatocellular carcinoma [27]. Similarly, the m^6^A-related modification pattern and the scoring system have also been constructed in gastric cancer [28], pancreatic adenocarcinoma [29], prostate cancer [30], kidney renal clear cell carcinoma [31], and glioblastoma [32]. In LUAD, two distinct m^6^A modification subgroups with different clinical outcomes were also identified based on 13 m^6^A regulators [14]. The m^6^A scoring system was also constructed to predict the survival and the response of immunotherapy [33, 34]. These studies just focused on m^6^A modification, validated the existence of different m^6^A modification patterns, and highlighted the significance of m^6^A in cancer while ignoring the functions of other kinds of RNA modifications.

Recently, some researchers have paid attention to other kinds of RNA modifications, except for m^6^A, and focused on the interactions with each other. For example, Chen et al. explored four major RNA adenosine modifications (m^6^A, m^1^A, alternative polyadenylation, and A-to-I) and determined two distinct RNA modification patterns in colorectal cancer, which were characterized by infiltrated inhibitory immune cells or survival favorable signaling pathways and showed significantly different survival [35]. Ye et al. identified 9 lncRNAs that were closely related to four types of RNA modification writers, including m^6^A, m^1^A, APA, and A-I, and constructed a risk prediction model to predict the survival of patients with ovarian carcinoma [36]. Song et al. focused on the crosstalk of m^6^A and m^5^C, identified m^6^A- and m^5^C-related lncRNAs, and revealed their importance in accessing the prognosis and immunotherapy response of colorectal cancer patients [37]. In our study, by searching the literature, we included a total of 100 RNA regulators that belong to eight types of RNA modifications and have been reported to play important roles in LUAD or other cancers. It is the first study to incorporate eight types of RNA modification regulators to evaluate their interactions with each other. Our study underlined the dysregulations and potential importance of other kinds of RNA modifications more than m^6^A, m^1^A, or m^5^C and provided clues for the investigations of other kinds of RNA modifications in the future. Moreover, based on the eight types of cancer-related RNA modifications, we discovered two distinct modification patterns in LUAD. The differences between the two subgroups revealed that cancer hallmarks and tumor microenvironments were extensively affected by these regulators, which highlighted the significance of other types of RNA modifications in LUAD. The RMScore constructed based on RNA modification patterns was determined to be the independent prognostic factor to predict the overall survival of LUAD patients, which underlined the prognostic value of RNA modifications in LUAD.

Taken together, our study was the first to explore the complicated roles and comprehensive interactions of eight types of cancer-related RNA modifications in LUAD. We revealed two distinct RNA modification patterns and their underlying biological characteristics and constructed a novel scoring system to predict the survival of LUAD patients. This study highlighted the significance of eight types of RNA modifications in LUAD and provided a novel horizon for the investigations of LUAD in the future.

## Data Availability

The data that support the findings of this study are openly available in TCGA datasets at https://portal.gdc.cancer.gov/and GEO datasets at https://www.ncbi.nlm.nih.gov/geo/.

## Supplementary Materials

Supplementary Materials Supplementary Figure 1. Mutations of eight types of RNA modification regulators in LUAD. Supplementary Figure 2. The prognostic value of eight types of RNA regulators in LUAD. Supplementary Figure 3. Kaplan-Meier curves of RNA regulators in LUAD patients in the GSE50081 dataset. Supplementary Figure 4. Top 10 interactions of RNA modification regulators in LUAD. Supplementary Figure 5. The mutation and expression pattern of RNA regulators in two RNA modification clusters in the TCGA-LUAD cohort. Supplementary Figure 6. Two RNA modification patterns of LUAD in the GSE41271 dataset. Supplementary Figure 7. RNA modification patterns of BRCA and COAD patients in the TCGA datasets. Supplementary Figure 8. The dysregulation and prognostic value of five RMScore-related genes in LUAD. Supplementary Table 1. Clinical information of LUAD patients in TCGA and GEO datasets. Supplementary Table 2. 100 RNA regulators of eight types of RNA modifications included in this study. Supplementary Table 3. Clinical information of 56 tumor and paired normal tissues in the TCGA-LUAD cohort. Supplementary Table 4. Multivariate Cox regression analysis of RNA regulators in TCGA-LUAD cohort. Supplementary Table 5. Correlations among eight types of RNA regulators in LUAD. Supplementary Table 6. The RNA modification pattern and RMScore of LUAD patients. Supplementary Table 7. Survival associated DEGs in univariate Cox regression analysis in TCGA-LUAD cohort. Supplementary Table 8. A total of 117 survival-associated DEGs were determined to be bonded and regulated by several RNA regulators in publicly available CLIP-seq data in the GEO database. Supplementary Table 9. Multivariate Cox regression analysis identified five genes that were used to construct the RMScore in LUAD.

## References

[1] Chen W., Zheng R., Baade P. D. (2016). Cancer statistics in China, 2015. *CA: A Cancer Journal for Clinicians*.

[2] Siegel R. L., Miller K. D., Jemal A. (2020). Cancer statistics, 2020. *CA: A Cancer Journal for Clinicians*.

[3] Travis W. D., Brambilla E., Nicholson A. G. (2015). The 2015 world health organization classification of lung tumors: impact of genetic, clinical and radiologic advances since the 2004 classification. *Journal of Thoracic Oncology*.

[4] Yuan M., Huang L. L., Chen J. H., Wu J., Xu Q. (2019). The emerging treatment landscape of targeted therapy in non-small-cell lung cancer. *Signal Transduction and Targeted Therapy*.

[5] Ribas A., Wolchok J. D. (2018). Cancer immunotherapy using checkpoint blockade. *Science*.

[6] Spella M., Stathopoulos G. T. (2021). Immune resistance in lung adenocarcinoma. *Cancers*.

[7] Boccaletto P., Machnicka M. A., Purta E. (2018). MODOMICS: a database of RNA modification pathways. 2017 update. *Nucleic Acids Research*.

[8] Liu Z., Wang T., She Y. (2021). N(6)-methyladenosine-modified circIGF2BP3 inhibits CD8(+) T-cell responses to facilitate tumor immune evasion by promoting the deubiquitination of PD-L1 in non-small cell lung cancer. *Molecular Cancer*.

[9] Qian X., Yang J., Qiu Q. (2021). LCAT3, a novel m6A-regulated long non-coding RNA, plays an oncogenic role in lung cancer via binding with FUBP1 to activate c-MYC. *Journal of Hematology & Oncology*.

[10] Jin D., Guo J., Wu Y. (2020). m(6) a demethylase ALKBH5 inhibits tumor growth and metastasis by reducing YTHDFs-mediated YAP expression and inhibiting miR-107/LATS2-mediated YAP activity in NSCLC. *Molecular Cancer*.

[11] Han X., Wang M., Zhao Y. L., Yang Y., Yang Y. G. (2021). RNA methylations in human cancers. *Seminars in Cancer Biology*.

[12] Delaunay S., Frye M. (2019). RNA modifications regulating cell fate in cancer. *Nature Cell Biology*.

[13] Barbieri I., Kouzarides T. (2020). Role of RNA modifications in cancer. *Nature Reviews Cancer*.

[14] Liu Y., Guo X., Zhao M. (2020). Contributions and prognostic values of m(6) a RNA methylation regulators in non-small-cell lung cancer. *Journal of Cellular Physiology*.

[15] Xu F., Huang X., Li Y., Chen Y., Lin L. (2021). m(6) a-related lncRNAs are potential biomarkers for predicting prognoses and immune responses in patients with LUAD. *Molecular Therapy - Nucleic Acids*.

[16] Chen H., Ge X. L., Zhang Z. Y. (2021). M(5)C regulator-mediated methylation modification patterns and tumor microenvironment infiltration characterization in lung adenocarcinoma. *Translational Lung Cancer Research*.

[17] He L., Li H., Wu A., Peng Y., Shu G., Yin G. (2019). Functions of N6-methyladenosine and its role in cancer. *Molecular Cancer*.

[18] Quan C., Belaydi O., Hu J. (2021). N(6)-methyladenosine in cancer immunotherapy: an undervalued therapeutic target. *Frontiers in Immunology*.

[19] Bohnsack K. E., Höbartner C., Bohnsack M. T. (2019). Eukaryotic 5-methylcytosine (m5C) RNA methyltransferases: mechanisms, cellular functions, and links to disease. *Genes*.

[20] Li Y., Li J., Luo M. (2018). Novel long noncoding RNA NMR promotes tumor progression via NSUN2 and BPTF in esophageal squamous cell carcinoma. *Cancer Letters*.

[21] Somme J., van Laer B., Roovers M., Steyaert J., Versées W., Droogmans L. (2014). Characterization of two homologous 2′-O-methyltransferases showing different specificities for their tRNA substrates. *RNA*.

[22] Wu H., Qin W., Lu S. (2020). Long noncoding RNA ZFAS1 promoting small nucleolar RNA-mediated 2′-O-methylation via NOP58 recruitment in colorectal cancer. *Molecular Cancer*.

[23] Penzo M., Guerrieri A. N., Zacchini F., Treré D., Montanaro L. (2017). RNA pseudouridylation in physiology and medicine: for better and for worse. *Genes*.

[24] Wang H., Chen S., Wei J., Song G., Zhao Y. (2020). A-to-I RNA editing in cancer: from evaluating the editing level to exploring the editing effects. *Frontiers in Oncology*.

[25] Cao J., Shu X., Feng X. H., Liu J. (2021). Mapping messenger RNA methylations at single base resolution. *Current Opinion in Chemical Biology*.

[26] Nedialkova D. D., Leidel S. A. (2015). Optimization of codon translation rates via tRNA modifications maintains proteome integrity. *Cell*.

[27] Huang X., Qiu Z., Li L., Chen B., Huang P. (2021). m6A regulator-mediated methylation modification patterns and tumor microenvironment infiltration characterization in hepatocellular carcinoma. *Aging (Albany NY)*.

[28] Zhang B., Wu Q., Li B., Wang D., Wang L., Zhou Y. L. (2020). m(6) a regulator-mediated methylation modification patterns and tumor microenvironment infiltration characterization in gastric cancer. *Molecular Cancer*.

[29] Wang L., Zhang S., Li H. (2021). Quantification of m6A RNA methylation modulators pattern was a potential biomarker for prognosis and associated with tumor immune microenvironment of pancreatic adenocarcinoma. *BMC Cancer*.

[30] Liu Z., Zhong J., Zeng J. (2021). Characterization of the m6A-associated tumor immune microenvironment in prostate cancer to aid immunotherapy. *Frontiers in Immunology*.

[31] Li H., Hu J., Yu A. (2021). RNA modification of N6-methyladenosine predicts immune phenotypes and therapeutic opportunities in kidney renal clear cell carcinoma. *Frontiers in Oncology*.

[32] Cai Z., Zhang J., Liu Z. (2021). Identification of an N6-methyladenosine (m6A)-related signature associated with clinical prognosis, immune response, and chemotherapy in primary glioblastomas. *Annals of Translational Medicine*.

[33] Li Y., Gu J., Xu F. (2021). Molecular characterization, biological function, tumor microenvironment association and clinical significance of m6A regulators in lung adenocarcinoma. *Briefings in Bioinformatics*.

[34] Zhang H., Hu J., Liu A. (2021). An N6-methyladenosine-related gene set variation score as a prognostic tool for lung adenocarcinoma. *Frontiers in Cell and Developmental Biology*.

[35] Chen H., Yao J., Bao R. (2021). Cross-talk of four types of RNA modification writers defines tumor microenvironment and pharmacogenomic landscape in colorectal cancer. *Molecular Cancer*.

[36] Ye L., Pan K., Fang S. (2022). Four types of RNA modification writer-related lncRNAs are effective predictors of prognosis and immunotherapy response in serous ovarian carcinoma. *Frontiers in Immunology*.

[37] Song W., Ren J., Xiang R., Yuan W., Fu T. (2022). Cross-talk between m(6)A- and m(5)C-related lncRNAs to construct a novel signature and predict the immune landscape of colorectal cancer patients. *Frontiers in Immunology*.

