# Supplementary material for: Crosstalk of Eight Types of RNA Modification Regulators Defines Tumor Microenvironments, Cancer Hallmarks, and Prognosis of Lung Adenocarcinoma
Source: J Oncol. 2022 Jul 11;2022:1285632. doi: 10.1155/2022/1285632 (PMC9293558; doi:10.1155/2022/1285632)
Supplement: Supplementary Materials — Supplementary Figure 1. Mutations of eight types of RNA modification regulators in LUAD. Supplementary Figure 2. The prognostic value of eight types of RNA regulators in LUAD. Supplementary Figure 3. Kaplan-Meier curves of RNA regulators in LUAD patients in the GSE50081 dataset. Supplementary Figure 4. Top 10 interactions of RNA modification regulators in LUAD. Supplementary Figure 5. The mutation and expression pattern of RNA regulators in two RNA modification clusters in the TCGA-LUAD cohort. Supplementary Figure 6. Two RNA modification patterns of LUAD in the GSE41271 dataset. Supplementary Figure 7. RNA modification patterns of BRCA and COAD patients in the TCGA datasets. Supplementary Figure 8. The dysregulation and prognostic value of five RMScore-related genes in LUAD. Supplementary Table 1. Clinical information of LUAD patients in TCGA and GEO datasets. Supplementary Table 2. 100 RNA regulators of eight types of RNA modifications included in this study. Supplementary Table 3. Clinical information of 56 tumor and paired normal tissues in the TCGA-LUAD cohort. Supplementary Table 4. Multivariate Cox regression analysis of RNA regulators in TCGA-LUAD cohort. Supplementary Table 5. Correlations among eight types of RNA regulators in LUAD. Supplementary Table 6. The RNA modification pattern and RMScore of LUAD patients. Supplementary Table 7. Survival associated DEGs in univariate Cox regression analysis in TCGA-LUAD cohort. Supplementary Table 8. A total of 117 survival-associated DEGs were determined to be bonded and regulated by several RNA regulators in publicly available CLIP-seq data in the GEO database. Supplementary Table 9. Multivariate Cox regression analysis identified five genes that were used to construct the RMScore in LUAD. [file 1285632.f1.zip › 1285632.f1/Supplementary Tables_R1.docx]

Table S1. Clinical information of LUAD patients in TCGA and GEO datasets.

|  | TCGA (n = 471) | GSE40419 (n = 87) | GSE41271 (n = 178) | GSE50081 (n = 127) |
| --- | --- | --- | --- | --- |
| Age (<60) | 129 (27.4%) | 25 (28.7%) | 63 (35.4%) | 19 (15.0%) |
| Gender (Male) | 217 (46.1%) | 53 (60.9%) | 89 (50.0%) | 65 (51.2%) |
| Tumor (T1-T2) | 413 (87.7%) | - | - | 125 (98.4%) |
| Node (N0-N1) | 402 (85.4%) | - | - | 127 (100%) |
| Metastasis (M0) | 450 (95.5%) | - | - | 127 (100%) |
| TNM (I-II) | 372 (79.0%) | 68 (78.2%) | 125 (70.2%) | 127 (100%) |

Table S2. 100 RNA regulators of eight types of RNA modifications included in this study.

| Gene.Name | Modification | Type |
| --- | --- | --- |
| METTL3 | M6A | Writer |
| METTL14 | M6A | Writer |
| METTL16 | M6A | Writer |
| WTAP | M6A | Writer |
| KIAA1429 | M6A | Writer |
| RBM15 | M6A | Writer |
| RBM15B | M6A | Writer |
| ZCCHC4 | M6A | Writer |
| ZC3H13 | M6A | Writer |
| METTL5 | M6A | Writer |
| CBLL1 | M6A | Writer |
| YTHDF1 | M6A | Reader |
| YTHDF2 | M6A | Reader |
| YTHDF3 | M6A | Reader |
| YTHDC1 | M6A | Reader |
| YTHDC2 | M6A | Reader |
| HNRNPA1 | M6A | Reader |
| HNRNPA2B1 | M6A | Reader |
| HNRNPC | M6A | Reader |
| IGF2BP1 | M6A | Reader |
| IGF2BP2 | M6A | Reader |
| IGF2BP3 | M6A | Reader |
| NKAP | M6A | Reader |
| EIF3A | M6A | Reader |
| FMR1 | M6A | Reader |
| ELAVL1 | M6A | Reader |
| G3BP1 | M6A | Reader |
| G3BP2 | M6A | Reader |
| PRRC2A | M6A | Reader |
| RBMX | M6A | Reader |
| LRPPRC | M6A | Reader |
| FTO | M6A | Eraser |
| ALKBH5 | M6A | Eraser |
| NSUN2 | M5C | Writer |
| NSUN3 | M5C | Writer |
| NSUN6 | M5C | Writer |
| NSUN7 | M5C | Writer |
| NOP2 | M5C | Writer |
| NSUN4 | M5C | Writer |
| NSUN5 | M5C | Writer |
| DNMT1 | M5C | Writer |
| TRDMT1 | M5C | Writer |
| DNMT3A | M5C | Writer |
| DNMT3B | M5C | Writer |
| ALYREF | M5C | Reader |
| YBX1 | M5C | Reader |
| TET1 | M5C | Eraser |
| TET3 | M5C | Eraser |
| TET2 | M5C | Eraser |
| TRMT6 | M1A | Writer |
| TRMT61A | M1A | Writer |
| TRMT61B | M1A | Writer |
| TRMT10A | M1A | Writer |
| TRMT10B | M1A | Writer |
| TRMT10C | M1A | Writer |
| RRP8 | M1A | Writer |
| ALKBH1 | M1A | Eraser |
| ALKBH3 | M1A | Eraser |
| CMTR1 | Nm | Writer |
| FBL | Nm | Writer |
| TRMT44 | Nm | Writer |
| TRMT13 | Nm | Writer |
| TARBP1 | Nm | Writer |
| FTSJ1 | Nm | Writer |
| SNORD48 | Nm | Writer |
| HENMT1 | Nm | Writer |
| CMTR2 | Nm | Writer |
| FBLL1 | Nm | Writer |
| FTSJ3 | Nm | Writer |
| MRM1 | Nm | Writer |
| MRM2 | Nm | Writer |
| MRM3 | Nm | Writer |
| TRMT11 | Nm | Writer |
| METTL1 | M7G | Writer |
| WDR4 | M7G | Writer |
| BUD23 | M7G | Writer |
| RNMT | M7G | Writer |
| TRMT112 | M7G | Writer |
| TGS1 | M7G | Writer |
| PUS1 | Ψ | Writer |
| PUS3 | Ψ | Writer |
| TRUB1 | Ψ | Writer |
| PUS7 | Ψ | Writer |
| PUS10 | Ψ | Writer |
| PUS7L | Ψ | Writer |
| RPUSD1 | Ψ | Writer |
| RPUSD2 | Ψ | Writer |
| RPUSD3 | Ψ | Writer |
| RPUSD4 | Ψ | Writer |
| DKC1 | Ψ | Writer |
| ADAR | A-to-I | Writer |
| ADARB1 | A-to-I | Writer |
| ADAT2 | A-to-I | Writer |
| ADAT3 | A-to-I | Writer |
| ADARB2 | A-to-I | Writer |
| ELP1 | mcm5s2U | Writer |
| ELP3 | mcm5s2U | Writer |
| ALKBH8 | mcm5s2U | Writer |
| CTU1 | mcm5s2U | Writer |
| CTU2 | mcm5s2U | Writer |

Table S3. Clinical information of 56 tumor and paired normal tissues in TCGA-LUAD cohort.

|  | TCGA (Tumor = 56) | TCGA (Normal = 56) |
| --- | --- | --- |
| Age (<60) | 15 (26.8%) | 15 (26.8%) |
| Gender (Male) | 23 (41.1%) | 23 (41.1%) |
| Tumor (T1-T2) | 53 (94.6%) | 53 (94.6%) |
| Node (N0-N1) | 43 (76.8%) | 43 (76.8%) |
| Metastasis (M0) | 54 (96.4%) | 54 (96.4%) |
| TNM (I-II) | 41 (73.2%) | 41 (73.2%) |

Table S4. Multivariate Cox regression analysis of RNA regulators in TCGA-LUAD cohort.

| Factor | coef | exp(coef) | se(coef) | z | Pr(>\|z\|) | lower .95 | upper .95 |
| --- | --- | --- | --- | --- | --- | --- | --- |
| Age | 0.19 | 1.21 | 0.19 | 1.00 | 0.3164 | 0.83 | 1.77 |
| Gender | 0.00 | 1.00 | 0.16 | 0.01 | 0.9946 | 0.73 | 1.38 |
| Stage | 0.48 | 1.61 | 0.08 | 5.89 | 0.0000 | 1.37 | 1.89 |
| METTL5 | 0.01 | 1.01 | 0.22 | 0.06 | 0.9521 | 0.66 | 1.56 |
| HNRNPA2B1 | 0.53 | 1.70 | 0.27 | 1.96 | 0.0503 | 1.00 | 2.88 |
| HNRNPC | 0.59 | 1.80 | 0.31 | 1.91 | 0.0567 | 0.98 | 3.28 |
| IGF2BP1 | 0.29 | 1.33 | 0.09 | 3.22 | 0.0013 | 1.12 | 1.59 |
| IGF2BP2 | 0.07 | 1.07 | 0.09 | 0.80 | 0.4261 | 0.90 | 1.28 |
| IGF2BP3 | -0.02 | 0.98 | 0.10 | -0.17 | 0.8656 | 0.80 | 1.20 |
| G3BP1 | 0.08 | 1.08 | 0.21 | 0.39 | 0.6994 | 0.72 | 1.63 |
| NSUN7 | -0.36 | 0.70 | 0.16 | -2.23 | 0.0258 | 0.51 | 0.96 |
| NOP2 | -0.23 | 0.79 | 0.17 | -1.38 | 0.1689 | 0.57 | 1.10 |
| NSUN4 | -1.08 | 0.34 | 0.31 | -3.50 | 0.0005 | 0.19 | 0.62 |
| TRDMT1 | -0.98 | 0.37 | 0.39 | -2.54 | 0.0110 | 0.18 | 0.80 |
| ALYREF | -0.20 | 0.82 | 0.17 | -1.18 | 0.2388 | 0.58 | 1.14 |
| TRMT10A | 0.44 | 1.55 | 0.22 | 1.97 | 0.0484 | 1.00 | 2.40 |
| TRMT10B | 0.01 | 1.01 | 0.22 | 0.03 | 0.9779 | 0.65 | 1.55 |
| ALKBH3 | 0.12 | 1.13 | 0.15 | 0.85 | 0.3946 | 0.85 | 1.51 |
| FTSJ3 | 0.24 | 1.27 | 0.23 | 1.05 | 0.2943 | 0.81 | 2.00 |
| WDR4 | 0.06 | 1.06 | 0.20 | 0.29 | 0.7725 | 0.71 | 1.58 |
| PUS10 | 0.22 | 1.25 | 0.28 | 0.80 | 0.4238 | 0.73 | 2.15 |

Table S5. Correlations among eight types of RNA regulators in LUAD.

| gene.x | gene.y | t | se | r | p | p.adj |
| --- | --- | --- | --- | --- | --- | --- |
| TGS1 | YTHDF3 | 21.16 | 0.03 | 0.70 | 2.98E-70 | 1.49E-68 |
| TET3 | DNMT3A | 21.08 | 0.03 | 0.70 | 6.87E-70 | 3.40E-68 |
| TGS1 | KIAA1429 | 19.59 | 0.03 | 0.67 | 6.98E-63 | 3.42E-61 |
| YTHDF3 | KIAA1429 | 19.20 | 0.03 | 0.66 | 4.57E-61 | 2.22E-59 |
| PUS1 | NOP2 | 18.53 | 0.04 | 0.65 | 6.26E-58 | 3.01E-56 |
| RPUSD4 | PUS3 | 18.32 | 0.04 | 0.65 | 6.05E-57 | 2.88E-55 |
| TET2 | YTHDC1 | 18.16 | 0.04 | 0.64 | 3.40E-56 | 1.60E-54 |
| DNMT3B | DNMT3A | 17.99 | 0.04 | 0.64 | 2.06E-55 | 9.61E-54 |
| BUD23 | NSUN5 | 17.38 | 0.04 | 0.63 | 1.31E-52 | 6.08E-51 |
| PUS7 | CBLL1 | 17.18 | 0.04 | 0.62 | 1.12E-51 | 5.14E-50 |
| EIF3A | ZC3H13 | 16.57 | 0.04 | 0.61 | 7.26E-49 | 3.30E-47 |
| TET2 | METTL14 | 16.05 | 0.04 | 0.60 | 1.57E-46 | 7.06E-45 |
| TRUB1 | YTHDF3 | 15.91 | 0.04 | 0.59 | 7.01E-46 | 3.13E-44 |
| YTHDC1 | METTL14 | 15.79 | 0.04 | 0.59 | 2.41E-45 | 1.07E-43 |
| DKC1 | PUS7 | 15.78 | 0.04 | 0.59 | 2.67E-45 | 1.17E-43 |
| ADAT2 | METTL3 | 15.67 | 0.04 | 0.59 | 8.16E-45 | 3.55E-43 |
| TARBP1 | METTL3 | 15.53 | 0.04 | 0.58 | 3.67E-44 | 1.59E-42 |
| DNMT1 | ELAVL1 | 15.42 | 0.04 | 0.58 | 1.07E-43 | 4.59E-42 |
| CMTR1 | PRRC2A | 15.39 | 0.04 | 0.58 | 1.52E-43 | 6.44E-42 |
| ADAT2 | TARBP1 | 15.36 | 0.04 | 0.58 | 2.08E-43 | 8.74E-42 |
| TET1 | DNMT3A | 15.25 | 0.04 | 0.58 | 6.27E-43 | 2.62E-41 |
| CTU1 | ADAT3 | 15.24 | 0.04 | 0.58 | 6.52E-43 | 2.70E-41 |
| YTHDC1 | ZC3H13 | 14.86 | 0.04 | 0.57 | 3.28E-41 | 1.35E-39 |
| TET3 | DNMT1 | 14.76 | 0.04 | 0.56 | 9.30E-41 | 3.79E-39 |
| TET3 | YTHDC1 | 14.66 | 0.04 | 0.56 | 2.41E-40 | 9.74E-39 |
| TRMT61B | LRPPRC | 14.63 | 0.04 | 0.56 | 3.41E-40 | 1.37E-38 |
| PUS7 | LRPPRC | 14.29 | 0.04 | 0.55 | 9.89E-39 | 3.93E-37 |
| DKC1 | ALYREF | 14.15 | 0.04 | 0.55 | 4.14E-38 | 1.63E-36 |
| TARBP1 | TRMT10B | 14.15 | 0.04 | 0.55 | 4.36E-38 | 1.71E-36 |
| PUS1 | DNMT3B | 14.13 | 0.04 | 0.55 | 4.85E-38 | 1.88E-36 |
| TRMT10B | METTL3 | 14.06 | 0.04 | 0.54 | 9.97E-38 | 3.85E-36 |
| TRUB1 | LRPPRC | 14.05 | 0.04 | 0.54 | 1.10E-37 | 4.20E-36 |
| DNMT3B | DNMT1 | 13.97 | 0.04 | 0.54 | 2.56E-37 | 9.74E-36 |
| TET2 | ZC3H13 | 13.71 | 0.04 | 0.53 | 3.22E-36 | 1.21E-34 |
| LRPPRC | KIAA1429 | 13.65 | 0.04 | 0.53 | 6.15E-36 | 2.30E-34 |
| DKC1 | LRPPRC | 13.57 | 0.04 | 0.53 | 1.25E-35 | 4.63E-34 |
| ADAT2 | NSUN6 | 13.53 | 0.04 | 0.53 | 1.86E-35 | 6.85E-34 |
| DNMT1 | HNRNPA2B1 | 13.49 | 0.04 | 0.53 | 2.90E-35 | 1.06E-33 |
| TRMT13 | METTL3 | 13.47 | 0.04 | 0.53 | 3.28E-35 | 1.19E-33 |
| TET3 | ZC3H13 | 13.47 | 0.04 | 0.53 | 3.51E-35 | 1.27E-33 |
| TET3 | PRRC2A | 13.33 | 0.04 | 0.52 | 1.35E-34 | 4.84E-33 |
| TET3 | DNMT3B | 13.27 | 0.04 | 0.52 | 2.35E-34 | 8.34E-33 |
| DNMT3A | PRRC2A | 13.07 | 0.04 | 0.52 | 1.58E-33 | 5.58E-32 |
| MRM1 | TRMT61A | 13.02 | 0.04 | 0.52 | 2.71E-33 | 9.51E-32 |
| DNMT3B | HNRNPA2B1 | 12.93 | 0.04 | 0.51 | 6.45E-33 | 2.25E-31 |
| DNMT3A | HNRNPA2B1 | 12.93 | 0.04 | 0.51 | 6.52E-33 | 2.26E-31 |
| FMR1 | NKAP | 12.91 | 0.04 | 0.51 | 7.93E-33 | 2.73E-31 |
| RNMT | TET3 | 12.90 | 0.04 | 0.51 | 8.60E-33 | 2.93E-31 |
| DNMT1 | PRRC2A | 12.90 | 0.04 | 0.51 | 8.75E-33 | 2.96E-31 |
| TET3 | TET1 | 12.78 | 0.04 | 0.51 | 2.61E-32 | 8.77E-31 |
| PRRC2A | EIF3A | 12.78 | 0.04 | 0.51 | 2.79E-32 | 9.34E-31 |
| TRMT10C | HNRNPC | 12.76 | 0.04 | 0.51 | 3.10E-32 | 1.03E-30 |
| PUS1 | MRM1 | 12.72 | 0.04 | 0.51 | 4.89E-32 | 1.61E-30 |
| TRMT10C | METTL5 | 12.70 | 0.04 | 0.51 | 5.71E-32 | 1.87E-30 |
| ADAT2 | TRMT10B | 12.69 | 0.04 | 0.51 | 6.08E-32 | 1.98E-30 |
| DNMT3B | NOP2 | 12.68 | 0.04 | 0.51 | 6.88E-32 | 2.23E-30 |
| CTU2 | NSUN5 | 12.63 | 0.04 | 0.50 | 1.10E-31 | 3.53E-30 |
| CBLL1 | KIAA1429 | 12.59 | 0.04 | 0.50 | 1.70E-31 | 5.44E-30 |
| DNMT1 | RBM15 | 12.54 | 0.04 | 0.50 | 2.67E-31 | 8.50E-30 |
| ZC3H13 | METTL14 | 12.49 | 0.04 | 0.50 | 4.14E-31 | 1.31E-29 |
| EIF3A | KIAA1429 | 12.43 | 0.04 | 0.50 | 7.05E-31 | 2.21E-29 |
| HNRNPA2B1 | RBM15 | 12.43 | 0.04 | 0.50 | 7.26E-31 | 2.26E-29 |
| ELP1 | TET3 | 12.43 | 0.04 | 0.50 | 7.50E-31 | 2.32E-29 |
| TARBP1 | NSUN6 | 12.40 | 0.04 | 0.50 | 9.42E-31 | 2.90E-29 |
| NSUN6 | METTL3 | 12.33 | 0.04 | 0.49 | 1.82E-30 | 5.58E-29 |
| ALYREF | ELAVL1 | 12.32 | 0.04 | 0.49 | 2.03E-30 | 6.16E-29 |
| ADAR | EIF3A | 12.31 | 0.04 | 0.49 | 2.23E-30 | 6.74E-29 |
| IGF2BP3 | IGF2BP1 | 12.27 | 0.04 | 0.49 | 3.39E-30 | 1.02E-28 |
| CMTR1 | TET3 | 12.25 | 0.04 | 0.49 | 4.07E-30 | 1.22E-28 |
| FTSJ3 | PRRC2A | 12.24 | 0.04 | 0.49 | 4.45E-30 | 1.32E-28 |
| TRMT10C | LRPPRC | 12.16 | 0.04 | 0.49 | 9.62E-30 | 2.84E-28 |
| FTSJ3 | HNRNPA2B1 | 12.09 | 0.04 | 0.49 | 1.69E-29 | 4.97E-28 |
| TET3 | HNRNPA2B1 | 12.09 | 0.04 | 0.49 | 1.84E-29 | 5.36E-28 |
| PRRC2A | RBM15B | 12.03 | 0.04 | 0.49 | 3.13E-29 | 9.09E-28 |
| LRPPRC | EIF3A | 11.99 | 0.04 | 0.48 | 4.27E-29 | 1.23E-27 |
| PUS1 | DNMT3A | 11.89 | 0.04 | 0.48 | 1.08E-28 | 3.09E-27 |
| TET3 | RBM15 | 11.81 | 0.04 | 0.48 | 2.24E-28 | 6.39E-27 |
| DKC1 | DNMT3B | 11.78 | 0.04 | 0.48 | 3.11E-28 | 8.82E-27 |
| G3BP2 | METTL14 | 11.73 | 0.04 | 0.48 | 4.73E-28 | 1.33E-26 |
| ALKBH5 | METTL16 | 11.73 | 0.04 | 0.48 | 4.82E-28 | 1.35E-26 |
| PUS7L | TET1 | 11.68 | 0.04 | 0.47 | 7.82E-28 | 2.18E-26 |
| TRMT10B | YTHDC2 | 11.62 | 0.04 | 0.47 | 1.28E-27 | 3.55E-26 |
| CTU2 | RPUSD1 | 11.60 | 0.04 | 0.47 | 1.52E-27 | 4.20E-26 |
| NSUN2 | HNRNPA2B1 | 11.60 | 0.04 | 0.47 | 1.55E-27 | 4.27E-26 |
| FBL | ALYREF | 11.60 | 0.04 | 0.47 | 1.61E-27 | 4.40E-26 |
| TET2 | TET3 | 11.59 | 0.04 | 0.47 | 1.76E-27 | 4.78E-26 |
| ADAR | PRRC2A | 11.57 | 0.04 | 0.47 | 2.00E-27 | 5.40E-26 |
| ALKBH8 | METTL14 | 11.56 | 0.04 | 0.47 | 2.26E-27 | 6.08E-26 |
| TGS1 | LRPPRC | 11.56 | 0.04 | 0.47 | 2.29E-27 | 6.12E-26 |
| ELAVL1 | HNRNPA2B1 | 11.54 | 0.04 | 0.47 | 2.72E-27 | 7.23E-26 |
| ELP1 | RBM15 | 11.53 | 0.04 | 0.47 | 2.90E-27 | 7.67E-26 |
| ELP1 | TET2 | 11.50 | 0.04 | 0.47 | 3.81E-27 | 1.00E-25 |
| LRPPRC | YTHDF3 | 11.50 | 0.04 | 0.47 | 4.06E-27 | 1.06E-25 |
| RPUSD1 | NSUN5 | 11.45 | 0.04 | 0.47 | 5.89E-27 | 1.53E-25 |
| ADAT2 | TRMT13 | 11.42 | 0.04 | 0.47 | 7.80E-27 | 2.02E-25 |
| RPUSD1 | TRMT61A | 11.40 | 0.04 | 0.47 | 9.63E-27 | 2.48E-25 |
| TET1 | DNMT3B | 11.36 | 0.04 | 0.46 | 1.42E-26 | 3.64E-25 |
| PUS1 | TRMT61A | 11.36 | 0.04 | 0.46 | 1.43E-26 | 3.64E-25 |
| ADAR | RBM15 | 11.34 | 0.04 | 0.46 | 1.73E-26 | 4.39E-25 |
| TET2 | YTHDC2 | 11.32 | 0.04 | 0.46 | 2.04E-26 | 5.15E-25 |
| FTSJ3 | DNMT1 | 11.31 | 0.04 | 0.46 | 2.13E-26 | 5.36E-25 |
| DKC1 | NSUN2 | 11.31 | 0.04 | 0.46 | 2.16E-26 | 5.40E-25 |
| ELP1 | YTHDC1 | 11.29 | 0.04 | 0.46 | 2.49E-26 | 6.18E-25 |
| FTSJ3 | TET3 | 11.24 | 0.04 | 0.46 | 4.21E-26 | 1.04E-24 |
| ADAR | CBLL1 | 11.17 | 0.04 | 0.46 | 7.45E-26 | 1.83E-24 |
| ALYREF | DNMT3B | 11.15 | 0.04 | 0.46 | 9.20E-26 | 2.26E-24 |
| TRMT13 | TRMT10B | 11.14 | 0.04 | 0.46 | 9.54E-26 | 2.33E-24 |
| TRMT10B | NSUN6 | 11.13 | 0.04 | 0.46 | 1.05E-25 | 2.54E-24 |
| TRUB1 | TRMT61B | 11.13 | 0.04 | 0.46 | 1.07E-25 | 2.59E-24 |
| CTU2 | TRMT61A | 11.13 | 0.04 | 0.46 | 1.09E-25 | 2.61E-24 |
| CMTR1 | YTHDC1 | 11.13 | 0.04 | 0.46 | 1.11E-25 | 2.66E-24 |
| DNMT3B | NSUN2 | 11.11 | 0.04 | 0.46 | 1.24E-25 | 2.94E-24 |
| TARBP1 | TRMT13 | 11.11 | 0.04 | 0.46 | 1.27E-25 | 3.02E-24 |
| DKC1 | ELAVL1 | 11.09 | 0.04 | 0.46 | 1.49E-25 | 3.51E-24 |
| TRMT61A | NSUN5 | 11.09 | 0.04 | 0.46 | 1.55E-25 | 3.64E-24 |
| ADAR | TET3 | 11.08 | 0.04 | 0.46 | 1.67E-25 | 3.90E-24 |
| PUS7L | LRPPRC | 11.07 | 0.04 | 0.46 | 1.76E-25 | 4.10E-24 |
| G3BP1 | EIF3A | 11.07 | 0.04 | 0.46 | 1.81E-25 | 4.19E-24 |
| PUS7 | KIAA1429 | 11.07 | 0.04 | 0.46 | 1.82E-25 | 4.20E-24 |
| DNMT3B | RBM15 | 11.05 | 0.04 | 0.45 | 2.14E-25 | 4.91E-24 |
| ADAT2 | TRMT11 | 11.04 | 0.04 | 0.45 | 2.36E-25 | 5.38E-24 |
| LRPPRC | HNRNPC | 11.04 | 0.04 | 0.45 | 2.47E-25 | 5.62E-24 |
| ALKBH8 | RPUSD4 | 11.03 | 0.04 | 0.45 | 2.55E-25 | 5.77E-24 |
| IGF2BP3 | IGF2BP2 | 10.97 | 0.04 | 0.45 | 4.61E-25 | 1.04E-23 |
| CTU2 | METTL1 | 10.96 | 0.04 | 0.45 | 4.86E-25 | 1.09E-23 |
| HNRNPA2B1 | YTHDC1 | 10.95 | 0.04 | 0.45 | 5.18E-25 | 1.16E-23 |
| EIF3A | YTHDC1 | 10.93 | 0.04 | 0.45 | 6.17E-25 | 1.37E-23 |
| PUS1 | METTL1 | 10.92 | 0.04 | 0.45 | 7.05E-25 | 1.56E-23 |
| ELP1 | METTL14 | 10.91 | 0.04 | 0.45 | 7.66E-25 | 1.69E-23 |
| TET3 | EIF3A | 10.89 | 0.04 | 0.45 | 9.03E-25 | 1.98E-23 |
| LRPPRC | CBLL1 | 10.89 | 0.04 | 0.45 | 9.11E-25 | 1.99E-23 |
| CMTR1 | DNMT1 | 10.86 | 0.04 | 0.45 | 1.12E-24 | 2.43E-23 |
| MRM2 | NSUN5 | 10.86 | 0.04 | 0.45 | 1.19E-24 | 2.58E-23 |
| YBX1 | ALYREF | 10.85 | 0.04 | 0.45 | 1.26E-24 | 2.71E-23 |
| ALYREF | METTL5 | 10.85 | 0.04 | 0.45 | 1.30E-24 | 2.80E-23 |
| TRMT13 | NSUN6 | 10.82 | 0.04 | 0.45 | 1.67E-24 | 3.58E-23 |
| TRUB1 | TGS1 | 10.82 | 0.04 | 0.45 | 1.69E-24 | 3.61E-23 |
| ADAR | FTSJ3 | 10.82 | 0.04 | 0.45 | 1.70E-24 | 3.61E-23 |
| DNMT3A | DNMT1 | 10.82 | 0.04 | 0.45 | 1.71E-24 | 3.61E-23 |
| G3BP1 | KIAA1429 | 10.81 | 0.04 | 0.45 | 1.80E-24 | 3.78E-23 |
| DKC1 | TRMT6 | 10.80 | 0.04 | 0.45 | 1.95E-24 | 4.10E-23 |
| DKC1 | PUS1 | 10.79 | 0.04 | 0.45 | 2.06E-24 | 4.30E-23 |
| TRMT10C | TRMT10A | 10.78 | 0.04 | 0.45 | 2.42E-24 | 5.02E-23 |
| ELP1 | EIF3A | 10.76 | 0.04 | 0.45 | 2.73E-24 | 5.65E-23 |
| TET1 | YTHDC1 | 10.74 | 0.04 | 0.44 | 3.27E-24 | 6.75E-23 |
| FTSJ3 | NSUN2 | 10.74 | 0.04 | 0.44 | 3.35E-24 | 6.88E-23 |
| PUS7 | TGS1 | 10.73 | 0.04 | 0.44 | 3.48E-24 | 7.12E-23 |
| RBMX | YTHDC1 | 10.70 | 0.04 | 0.44 | 4.73E-24 | 9.64E-23 |
| DKC1 | METTL1 | 10.69 | 0.04 | 0.44 | 4.96E-24 | 1.01E-22 |
| FBL | METTL5 | 10.68 | 0.04 | 0.44 | 5.73E-24 | 1.16E-22 |
| HNRNPC | METTL5 | 10.66 | 0.04 | 0.44 | 6.77E-24 | 1.36E-22 |
| PUS7 | NSUN2 | 10.64 | 0.04 | 0.44 | 7.71E-24 | 1.55E-22 |
| RBMX | FMR1 | 10.61 | 0.04 | 0.44 | 9.81E-24 | 1.96E-22 |
| DKC1 | WDR4 | 10.61 | 0.04 | 0.44 | 1.02E-23 | 2.04E-22 |
| YTHDC1 | RBM15 | 10.59 | 0.04 | 0.44 | 1.17E-23 | 2.31E-22 |
| ADAR | YTHDC1 | 10.57 | 0.04 | 0.44 | 1.49E-23 | 2.94E-22 |
| TRUB1 | TRMT10C | 10.55 | 0.04 | 0.44 | 1.72E-23 | 3.39E-22 |
| DNMT3A | YTHDC1 | 10.54 | 0.04 | 0.44 | 1.80E-23 | 3.52E-22 |
| PUS7L | RBM15 | 10.54 | 0.04 | 0.44 | 1.81E-23 | 3.53E-22 |
| TRMT13 | RBMX | 10.54 | 0.04 | 0.44 | 1.90E-23 | 3.69E-22 |
| TRMT11 | WTAP | 10.53 | 0.04 | 0.44 | 1.95E-23 | 3.78E-22 |
| PUS7L | RNMT | 10.52 | 0.04 | 0.44 | 2.19E-23 | 4.23E-22 |
| DNMT3B | IGF2BP3 | 10.48 | 0.04 | 0.44 | 3.01E-23 | 5.76E-22 |
| ADAR | PUS7L | 10.48 | 0.04 | 0.44 | 3.03E-23 | 5.78E-22 |
| METTL1 | ALYREF | 10.48 | 0.04 | 0.44 | 3.12E-23 | 5.92E-22 |
| DKC1 | KIAA1429 | 10.46 | 0.04 | 0.43 | 3.87E-23 | 7.31E-22 |
| ZC3H13 | METTL16 | 10.45 | 0.04 | 0.43 | 4.17E-23 | 7.86E-22 |
| DNMT3A | METTL3 | 10.44 | 0.04 | 0.43 | 4.23E-23 | 7.94E-22 |
| PUS7 | TRMT61B | 10.44 | 0.04 | 0.43 | 4.36E-23 | 8.16E-22 |
| TRMT10C | TRMT61B | 10.39 | 0.04 | 0.43 | 6.66E-23 | 1.24E-21 |
| DKC1 | RBMX | 10.39 | 0.04 | 0.43 | 6.99E-23 | 1.30E-21 |
| G3BP1 | YTHDC2 | 10.36 | 0.04 | 0.43 | 8.55E-23 | 1.58E-21 |
| PUS7L | KIAA1429 | 10.35 | 0.04 | 0.43 | 9.56E-23 | 1.76E-21 |
| ELAVL1 | HNRNPA1 | 10.34 | 0.04 | 0.43 | 1.00E-22 | 1.84E-21 |
| DKC1 | NKAP | 10.33 | 0.04 | 0.43 | 1.13E-22 | 2.08E-21 |
| NSUN4 | YTHDF2 | 10.33 | 0.04 | 0.43 | 1.16E-22 | 2.12E-21 |
| ALYREF | NSUN5 | 10.33 | 0.04 | 0.43 | 1.17E-22 | 2.12E-21 |
| TGS1 | CBLL1 | 10.32 | 0.04 | 0.43 | 1.18E-22 | 2.13E-21 |
| WDR4 | DNMT3B | 10.32 | 0.04 | 0.43 | 1.20E-22 | 2.17E-21 |
| RBMX | HNRNPC | 10.32 | 0.04 | 0.43 | 1.21E-22 | 2.18E-21 |
| ALYREF | HNRNPC | 10.31 | 0.04 | 0.43 | 1.31E-22 | 2.35E-21 |
| TET1 | KIAA1429 | 10.29 | 0.04 | 0.43 | 1.56E-22 | 2.79E-21 |
| PUS7L | TRUB1 | 10.29 | 0.04 | 0.43 | 1.58E-22 | 2.81E-21 |
| DKC1 | HNRNPA2B1 | 10.27 | 0.04 | 0.43 | 1.94E-22 | 3.44E-21 |
| WDR4 | NOP2 | 10.25 | 0.04 | 0.43 | 2.18E-22 | 3.85E-21 |
| CMTR1 | METTL3 | 10.24 | 0.04 | 0.43 | 2.37E-22 | 4.16E-21 |
| DKC1 | CBLL1 | 10.24 | 0.04 | 0.43 | 2.37E-22 | 4.16E-21 |
| WDR4 | HNRNPA2B1 | 10.24 | 0.04 | 0.43 | 2.41E-22 | 4.22E-21 |
| ADAR | RNMT | 10.18 | 0.04 | 0.43 | 3.98E-22 | 6.94E-21 |
| CMTR1 | DNMT3A | 10.17 | 0.04 | 0.43 | 4.18E-22 | 7.25E-21 |
| FMR1 | YTHDC1 | 10.11 | 0.04 | 0.42 | 7.18E-22 | 1.24E-20 |
| CTU1 | RPUSD1 | 10.11 | 0.04 | 0.42 | 7.23E-22 | 1.25E-20 |
| DNMT3A | NOP2 | 10.10 | 0.04 | 0.42 | 8.05E-22 | 1.38E-20 |
| TARBP1 | DNMT3A | 10.09 | 0.04 | 0.42 | 8.33E-22 | 1.43E-20 |
| WDR4 | DNMT1 | 10.07 | 0.04 | 0.42 | 9.99E-22 | 1.70E-20 |
| WDR4 | NSUN5 | 10.05 | 0.04 | 0.42 | 1.17E-21 | 2.00E-20 |
| TET1 | EIF3A | 10.05 | 0.04 | 0.42 | 1.19E-21 | 2.01E-20 |
| ADAR | DNMT1 | 10.04 | 0.04 | 0.42 | 1.28E-21 | 2.16E-20 |
| FTSJ1 | ALYREF | 10.03 | 0.04 | 0.42 | 1.35E-21 | 2.28E-20 |
| DNMT3A | NSUN2 | 10.01 | 0.04 | 0.42 | 1.68E-21 | 2.82E-20 |
| DNMT3A | RBM15B | 10.00 | 0.04 | 0.42 | 1.79E-21 | 3.00E-20 |
| DNMT3B | ELAVL1 | 10.00 | 0.04 | 0.42 | 1.82E-21 | 3.03E-20 |
| NOP2 | NSUN2 | 9.99 | 0.04 | 0.42 | 1.89E-21 | 3.14E-20 |
| ELP1 | RNMT | 9.99 | 0.04 | 0.42 | 1.98E-21 | 3.28E-20 |
| PUS7L | RBMX | 9.99 | 0.04 | 0.42 | 1.99E-21 | 3.29E-20 |
| PUS1 | NSUN5 | 9.98 | 0.04 | 0.42 | 2.16E-21 | 3.55E-20 |
| DKC1 | HNRNPC | 9.97 | 0.04 | 0.42 | 2.35E-21 | 3.85E-20 |
| DKC1 | NOP2 | 9.95 | 0.04 | 0.42 | 2.65E-21 | 4.32E-20 |
| PUS7L | DNMT1 | 9.93 | 0.04 | 0.42 | 3.29E-21 | 5.36E-20 |
| YTHDC2 | METTL14 | 9.92 | 0.04 | 0.42 | 3.37E-21 | 5.46E-20 |
| DNMT3B | IGF2BP1 | 9.91 | 0.04 | 0.42 | 3.67E-21 | 5.95E-20 |
| RPUSD1 | PUS1 | 9.90 | 0.04 | 0.42 | 4.07E-21 | 6.57E-20 |
| RPUSD2 | MRM3 | 9.90 | 0.04 | 0.42 | 4.14E-21 | 6.67E-20 |
| TRMT112 | METTL1 | 9.88 | 0.04 | 0.42 | 4.84E-21 | 7.77E-20 |
| DNMT1 | CBLL1 | 9.88 | 0.04 | 0.42 | 4.92E-21 | 7.87E-20 |
| DNMT3B | METTL3 | 9.84 | 0.04 | 0.41 | 6.55E-21 | 1.04E-19 |
| PUS1 | ALYREF | 9.82 | 0.04 | 0.41 | 7.76E-21 | 1.23E-19 |
| NSUN2 | RBM15 | 9.81 | 0.04 | 0.41 | 8.35E-21 | 1.32E-19 |
| RNMT | DNMT3A | 9.78 | 0.04 | 0.41 | 1.07E-20 | 1.69E-19 |
| CTU1 | PUS1 | 9.76 | 0.04 | 0.41 | 1.35E-20 | 2.12E-19 |
| RNMT | YTHDC1 | 9.76 | 0.04 | 0.41 | 1.35E-20 | 2.12E-19 |
| FBL | HNRNPA1 | 9.76 | 0.04 | 0.41 | 1.36E-20 | 2.12E-19 |
| CMTR1 | DNMT3B | 9.75 | 0.04 | 0.41 | 1.37E-20 | 2.13E-19 |
| TRUB1 | HNRNPC | 9.74 | 0.04 | 0.41 | 1.60E-20 | 2.47E-19 |
| METTL1 | METTL5 | 9.73 | 0.04 | 0.41 | 1.66E-20 | 2.57E-19 |
| TRMT61B | YTHDF3 | 9.72 | 0.04 | 0.41 | 1.81E-20 | 2.79E-19 |
| DKC1 | FMR1 | 9.71 | 0.04 | 0.41 | 2.00E-20 | 3.08E-19 |
| RPUSD3 | MRM1 | 9.70 | 0.04 | 0.41 | 2.13E-20 | 3.26E-19 |
| FTSJ3 | EIF3A | 9.70 | 0.04 | 0.41 | 2.16E-20 | 3.29E-19 |
| TRMT11 | TRMT13 | 9.68 | 0.04 | 0.41 | 2.50E-20 | 3.80E-19 |
| ADAR | FMR1 | 9.66 | 0.04 | 0.41 | 2.84E-20 | 4.31E-19 |
| ELP1 | ZC3H13 | 9.64 | 0.04 | 0.41 | 3.48E-20 | 5.26E-19 |
| ELP1 | YTHDC2 | 9.64 | 0.04 | 0.41 | 3.57E-20 | 5.38E-19 |
| CTU2 | TRMT112 | 9.64 | 0.04 | 0.41 | 3.58E-20 | 5.38E-19 |
| ADAR | G3BP1 | 9.63 | 0.04 | 0.41 | 3.65E-20 | 5.47E-19 |
| ALKBH1 | RBMX | 9.63 | 0.04 | 0.41 | 3.68E-20 | 5.49E-19 |
| RNMT | KIAA1429 | 9.63 | 0.04 | 0.41 | 3.71E-20 | 5.53E-19 |
| NOP2 | HNRNPA2B1 | 9.63 | 0.04 | 0.41 | 3.76E-20 | 5.58E-19 |
| ELP1 | ADAR | 9.63 | 0.04 | 0.41 | 3.91E-20 | 5.79E-19 |
| PRRC2A | YTHDC1 | 9.62 | 0.04 | 0.41 | 4.21E-20 | 6.21E-19 |
| RNMT | ZC3H13 | 9.61 | 0.04 | 0.41 | 4.28E-20 | 6.30E-19 |
| FTSJ3 | ALYREF | 9.61 | 0.04 | 0.41 | 4.49E-20 | 6.60E-19 |
| TET1 | ZC3H13 | 9.61 | 0.04 | 0.41 | 4.60E-20 | 6.73E-19 |
| RBMX | HNRNPA1 | 9.60 | 0.04 | 0.41 | 4.67E-20 | 6.81E-19 |
| PUS7L | YTHDF3 | 9.60 | 0.04 | 0.41 | 4.85E-20 | 7.06E-19 |
| ADAR | DNMT3A | 9.60 | 0.04 | 0.41 | 4.93E-20 | 7.16E-19 |
| YTHDC2 | YTHDC1 | 9.59 | 0.04 | 0.40 | 5.37E-20 | 7.78E-19 |
| PUS1 | WDR4 | 9.57 | 0.04 | 0.40 | 6.12E-20 | 8.83E-19 |
| FTSJ3 | DNMT3A | 9.56 | 0.04 | 0.40 | 6.60E-20 | 9.49E-19 |
| PRRC2A | HNRNPA2B1 | 9.56 | 0.04 | 0.40 | 6.62E-20 | 9.50E-19 |
| FMR1 | RBM15 | 9.56 | 0.04 | 0.40 | 6.79E-20 | 9.71E-19 |
| TRUB1 | RBMX | 9.56 | 0.04 | 0.40 | 6.85E-20 | 9.78E-19 |
| FBL | ELAVL1 | 9.55 | 0.04 | 0.40 | 7.16E-20 | 1.02E-18 |
| YBX1 | HNRNPC | 9.54 | 0.04 | 0.40 | 7.54E-20 | 1.07E-18 |
| TGS1 | TRMT61B | 9.52 | 0.04 | 0.40 | 9.51E-20 | 1.34E-18 |
| PUS7L | TET3 | 9.51 | 0.04 | 0.40 | 1.01E-19 | 1.42E-18 |
| TRUB1 | EIF3A | 9.50 | 0.04 | 0.40 | 1.09E-19 | 1.54E-18 |
| MRM2 | HNRNPA2B1 | 9.49 | 0.04 | 0.40 | 1.22E-19 | 1.71E-18 |
| TGS1 | EIF3A | 9.46 | 0.04 | 0.40 | 1.47E-19 | 2.06E-18 |
| DNMT1 | FMR1 | 9.45 | 0.04 | 0.40 | 1.60E-19 | 2.23E-18 |
| DNMT3A | RBM15 | 9.45 | 0.04 | 0.40 | 1.62E-19 | 2.26E-18 |
| TRMT112 | ALYREF | 9.45 | 0.04 | 0.40 | 1.65E-19 | 2.29E-18 |
| CTU2 | FTSJ1 | 9.45 | 0.04 | 0.40 | 1.66E-19 | 2.30E-18 |
| PUS7L | G3BP1 | 9.43 | 0.04 | 0.40 | 1.96E-19 | 2.71E-18 |
| FBL | YBX1 | 9.41 | 0.04 | 0.40 | 2.24E-19 | 3.08E-18 |
| YBX1 | IGF2BP3 | 9.41 | 0.04 | 0.40 | 2.27E-19 | 3.11E-18 |
| PUS1 | NSUN2 | 9.39 | 0.04 | 0.40 | 2.61E-19 | 3.57E-18 |
| TRMT13 | RBM15 | 9.39 | 0.04 | 0.40 | 2.67E-19 | 3.65E-18 |
| PUS7L | EIF3A | 9.38 | 0.04 | 0.40 | 2.77E-19 | 3.77E-18 |
| CTU2 | ALYREF | 9.37 | 0.04 | 0.40 | 2.97E-19 | 4.04E-18 |
| RNMT | TET1 | 9.37 | 0.04 | 0.40 | 3.19E-19 | 4.32E-18 |
| TRMT10A | ZCCHC4 | 9.35 | 0.04 | 0.40 | 3.51E-19 | 4.74E-18 |
| PUS7L | TGS1 | 9.35 | 0.04 | 0.40 | 3.63E-19 | 4.89E-18 |
| ALYREF | IGF2BP3 | 9.34 | 0.04 | 0.40 | 3.75E-19 | 5.04E-18 |
| TET3 | RBM15B | 9.34 | 0.04 | 0.40 | 4.04E-19 | 5.41E-18 |
| TRMT61B | RBMX | 9.30 | 0.04 | 0.39 | 5.51E-19 | 7.37E-18 |
| TRMT10B | YTHDC1 | 9.29 | 0.04 | 0.39 | 5.62E-19 | 7.48E-18 |
| TRMT10A | HNRNPC | 9.29 | 0.04 | 0.39 | 5.94E-19 | 7.90E-18 |
| TET1 | PRRC2A | 9.28 | 0.04 | 0.39 | 6.27E-19 | 8.31E-18 |
| TRMT6 | LRPPRC | 9.26 | 0.04 | 0.39 | 7.23E-19 | 9.55E-18 |
| RNMT | EIF3A | 9.24 | 0.04 | 0.39 | 8.93E-19 | 1.18E-17 |
| FMR1 | HNRNPA2B1 | 9.20 | 0.04 | 0.39 | 1.19E-18 | 1.56E-17 |
| PUS7L | TRMT6 | 9.20 | 0.04 | 0.39 | 1.22E-18 | 1.61E-17 |
| DNMT3B | PRRC2A | 9.19 | 0.04 | 0.39 | 1.30E-18 | 1.70E-17 |
| EIF3A | RBM15B | 9.18 | 0.04 | 0.39 | 1.43E-18 | 1.87E-17 |
| TRUB1 | KIAA1429 | 9.15 | 0.04 | 0.39 | 1.78E-18 | 2.32E-17 |
| ELP1 | DNMT1 | 9.14 | 0.04 | 0.39 | 1.88E-18 | 2.44E-17 |
| DNMT3A | ELAVL1 | 9.14 | 0.04 | 0.39 | 1.91E-18 | 2.47E-17 |
| TRMT61B | DNMT3A | 9.14 | 0.04 | 0.39 | 1.92E-18 | 2.47E-17 |
| G3BP1 | CBLL1 | 9.13 | 0.04 | 0.39 | 2.00E-18 | 2.57E-17 |
| PRRC2A | ZC3H13 | 9.13 | 0.04 | 0.39 | 2.08E-18 | 2.67E-17 |
| PUS7L | CBLL1 | 9.11 | 0.04 | 0.39 | 2.42E-18 | 3.10E-17 |
| PUS7 | ALYREF | 9.10 | 0.04 | 0.39 | 2.55E-18 | 3.26E-17 |
| DKC1 | DNMT1 | 9.10 | 0.04 | 0.39 | 2.56E-18 | 3.26E-17 |
| LRPPRC | RBMX | 9.08 | 0.04 | 0.39 | 2.98E-18 | 3.80E-17 |
| TGS1 | G3BP1 | 9.06 | 0.04 | 0.39 | 3.68E-18 | 4.66E-17 |
| PUS7L | YTHDC1 | 9.04 | 0.04 | 0.39 | 4.01E-18 | 5.08E-17 |
| RNMT | TET2 | 9.04 | 0.04 | 0.39 | 4.12E-18 | 5.20E-17 |
| TET1 | RBMX | 9.03 | 0.04 | 0.38 | 4.40E-18 | 5.54E-17 |
| WDR4 | TRMT61A | 9.03 | 0.04 | 0.38 | 4.60E-18 | 5.78E-17 |
| CMTR1 | TRMT10B | 9.03 | 0.04 | 0.38 | 4.61E-18 | 5.78E-17 |
| TRMT6 | ALYREF | 9.01 | 0.04 | 0.38 | 5.30E-18 | 6.63E-17 |
| CTU2 | PUS1 | 9.00 | 0.04 | 0.38 | 5.57E-18 | 6.94E-17 |
| PRRC2A | ELAVL1 | 9.00 | 0.04 | 0.38 | 5.62E-18 | 6.99E-17 |
| TARBP1 | RBM15 | 8.99 | 0.04 | 0.38 | 6.01E-18 | 7.45E-17 |
| WDR4 | NSUN2 | 8.98 | 0.04 | 0.38 | 6.70E-18 | 8.29E-17 |
| G3BP2 | G3BP1 | 8.98 | 0.04 | 0.38 | 6.80E-18 | 8.40E-17 |
| TRMT10C | NSUN3 | 8.97 | 0.04 | 0.38 | 7.06E-18 | 8.70E-17 |
| CMTR1 | FMR1 | 8.95 | 0.04 | 0.38 | 8.02E-18 | 9.83E-17 |
| EIF3A | CBLL1 | 8.95 | 0.04 | 0.38 | 8.60E-18 | 1.05E-16 |
| G3BP2 | YTHDF3 | 8.94 | 0.04 | 0.38 | 8.74E-18 | 1.07E-16 |
| FTSJ1 | NSUN5 | 8.94 | 0.04 | 0.38 | 9.14E-18 | 1.11E-16 |
| TRMT10C | YBX1 | 8.93 | 0.04 | 0.38 | 1.00E-17 | 1.22E-16 |
| ELP1 | TRMT10B | 8.92 | 0.04 | 0.38 | 1.02E-17 | 1.24E-16 |
| TARBP1 | HNRNPA2B1 | 8.92 | 0.04 | 0.38 | 1.03E-17 | 1.25E-16 |
| DKC1 | IGF2BP3 | 8.92 | 0.04 | 0.38 | 1.07E-17 | 1.29E-16 |
| ADAR | CMTR1 | 8.89 | 0.04 | 0.38 | 1.31E-17 | 1.57E-16 |
| RNMT | LRPPRC | 8.89 | 0.04 | 0.38 | 1.33E-17 | 1.59E-16 |
| ADAR | HNRNPA2B1 | 8.88 | 0.04 | 0.38 | 1.41E-17 | 1.69E-16 |
| TRDMT1 | RBMX | 8.87 | 0.04 | 0.38 | 1.56E-17 | 1.86E-16 |
| TRMT13 | TRDMT1 | 8.86 | 0.04 | 0.38 | 1.61E-17 | 1.92E-16 |
| TRMT112 | FTSJ1 | 8.86 | 0.04 | 0.38 | 1.63E-17 | 1.93E-16 |
| ADAR | KIAA1429 | 8.86 | 0.04 | 0.38 | 1.70E-17 | 2.01E-16 |
| TRMT61A | DNMT3B | 8.85 | 0.04 | 0.38 | 1.84E-17 | 2.17E-16 |
| PUS7 | NOP2 | 8.82 | 0.04 | 0.38 | 2.21E-17 | 2.60E-16 |
| DNMT3A | NSUN6 | 8.82 | 0.04 | 0.38 | 2.25E-17 | 2.65E-16 |
| DNMT1 | NSUN2 | 8.80 | 0.04 | 0.38 | 2.67E-17 | 3.14E-16 |
| NOP2 | ELAVL1 | 8.79 | 0.04 | 0.38 | 2.79E-17 | 3.27E-16 |
| TRMT10A | LRPPRC | 8.79 | 0.04 | 0.38 | 2.81E-17 | 3.29E-16 |
| DNMT3A | YTHDF1 | 8.77 | 0.04 | 0.38 | 3.31E-17 | 3.85E-16 |
| ELAVL1 | YTHDF1 | 8.75 | 0.04 | 0.37 | 3.91E-17 | 4.54E-16 |
| TRUB1 | ZCCHC4 | 8.75 | 0.04 | 0.37 | 3.93E-17 | 4.55E-16 |
| RBMX | HNRNPA2B1 | 8.75 | 0.04 | 0.37 | 3.95E-17 | 4.57E-16 |
| ALKBH8 | ZC3H13 | 8.75 | 0.04 | 0.37 | 4.01E-17 | 4.62E-16 |
| DKC1 | TRMT61B | 8.74 | 0.04 | 0.37 | 4.03E-17 | 4.63E-16 |
| RPUSD3 | MRM3 | 8.74 | 0.04 | 0.37 | 4.24E-17 | 4.87E-16 |
| METTL1 | NSUN5 | 8.72 | 0.04 | 0.37 | 5.00E-17 | 5.71E-16 |
| TRMT61A | METTL3 | 8.72 | 0.04 | 0.37 | 5.03E-17 | 5.74E-16 |
| FBL | HNRNPC | 8.71 | 0.04 | 0.37 | 5.32E-17 | 6.05E-16 |
| METTL1 | NOP2 | 8.70 | 0.04 | 0.37 | 5.58E-17 | 6.33E-16 |
| TARBP1 | YTHDC2 | 8.70 | 0.04 | 0.37 | 5.72E-17 | 6.46E-16 |
| TRMT61B | KIAA1429 | 8.70 | 0.04 | 0.37 | 5.72E-17 | 6.46E-16 |
| G3BP1 | METTL14 | 8.68 | 0.04 | 0.37 | 6.66E-17 | 7.51E-16 |
| RBMX | G3BP1 | 8.67 | 0.04 | 0.37 | 6.90E-17 | 7.76E-16 |
| TRMT10C | TRMT6 | 8.66 | 0.04 | 0.37 | 7.47E-17 | 8.39E-16 |
| DNMT3A | LRPPRC | 8.66 | 0.04 | 0.37 | 7.55E-17 | 8.46E-16 |
| TET3 | NSUN2 | 8.66 | 0.04 | 0.37 | 7.67E-17 | 8.55E-16 |
| TGS1 | TET1 | 8.66 | 0.04 | 0.37 | 7.67E-17 | 8.55E-16 |
| ADARB1 | FTO | 8.66 | 0.04 | 0.37 | 7.77E-17 | 8.64E-16 |
| DKC1 | TGS1 | 8.65 | 0.04 | 0.37 | 8.30E-17 | 9.21E-16 |
| ADAR | LRPPRC | 8.64 | 0.04 | 0.37 | 9.20E-17 | 1.02E-15 |
| CMTR1 | TET1 | 8.63 | 0.04 | 0.37 | 9.49E-17 | 1.05E-15 |
| PUS7 | TRMT6 | 8.63 | 0.04 | 0.37 | 9.62E-17 | 1.06E-15 |
| RNMT | DNMT1 | 8.62 | 0.04 | 0.37 | 9.99E-17 | 1.10E-15 |
| PUS7L | DNMT3A | 8.62 | 0.04 | 0.37 | 1.01E-16 | 1.11E-15 |
| DNMT3A | NSUN7 | 8.61 | 0.04 | 0.37 | 1.12E-16 | 1.22E-15 |
| NSUN2 | LRPPRC | 8.61 | 0.04 | 0.37 | 1.14E-16 | 1.25E-15 |
| PUS7L | FMR1 | 8.58 | 0.04 | 0.37 | 1.42E-16 | 1.55E-15 |
| TET3 | FMR1 | 8.57 | 0.04 | 0.37 | 1.51E-16 | 1.65E-15 |
| TRUB1 | TRMT10A | 8.57 | 0.04 | 0.37 | 1.54E-16 | 1.67E-15 |
| DNMT1 | IGF2BP3 | 8.56 | 0.04 | 0.37 | 1.59E-16 | 1.72E-15 |
| TRMT112 | METTL5 | 8.56 | 0.04 | 0.37 | 1.63E-16 | 1.77E-15 |
| TET1 | LRPPRC | 8.54 | 0.04 | 0.37 | 1.90E-16 | 2.05E-15 |
| DKC1 | METTL5 | 8.54 | 0.04 | 0.37 | 1.92E-16 | 2.07E-15 |
| ADAT2 | PUS1 | 8.53 | 0.04 | 0.37 | 2.09E-16 | 2.25E-15 |
| TRMT6 | RBMX | 8.52 | 0.04 | 0.37 | 2.13E-16 | 2.29E-15 |
| ADARB2 | ADARB1 | 8.51 | 0.04 | 0.37 | 2.30E-16 | 2.46E-15 |
| YTHDC1 | METTL16 | 8.51 | 0.04 | 0.37 | 2.34E-16 | 2.50E-15 |
| HNRNPA2B1 | METTL3 | 8.51 | 0.04 | 0.37 | 2.42E-16 | 2.58E-15 |
| CTU2 | BUD23 | 8.51 | 0.04 | 0.37 | 2.44E-16 | 2.59E-15 |
| PUS7 | EIF3A | 8.50 | 0.04 | 0.37 | 2.46E-16 | 2.61E-15 |
| TET3 | KIAA1429 | 8.50 | 0.04 | 0.37 | 2.62E-16 | 2.78E-15 |
| TRMT10C | ALYREF | 8.49 | 0.04 | 0.37 | 2.67E-16 | 2.82E-15 |
| TARBP1 | NSUN7 | 8.49 | 0.04 | 0.37 | 2.68E-16 | 2.82E-15 |
| ELP1 | PRRC2A | 8.48 | 0.04 | 0.36 | 2.90E-16 | 3.05E-15 |
| ADAR | ZC3H13 | 8.48 | 0.04 | 0.36 | 2.97E-16 | 3.12E-15 |
| FTSJ3 | CMTR1 | 8.48 | 0.04 | 0.36 | 3.00E-16 | 3.15E-15 |
| ALYREF | DNMT1 | 8.46 | 0.04 | 0.36 | 3.42E-16 | 3.58E-15 |
| ALKBH8 | FTSJ3 | 8.46 | 0.04 | 0.36 | 3.44E-16 | 3.59E-15 |
| MRM3 | ALKBH5 | 8.46 | 0.04 | 0.36 | 3.52E-16 | 3.66E-15 |
| ADAT2 | SNORD48 | 8.45 | 0.04 | 0.36 | 3.71E-16 | 3.86E-15 |
| EIF3A | HNRNPA1 | 8.45 | 0.04 | 0.36 | 3.73E-16 | 3.86E-15 |
| PUS7 | TRMT10C | 8.43 | 0.04 | 0.36 | 4.40E-16 | 4.54E-15 |
| PUS7 | DNMT3B | 8.42 | 0.04 | 0.36 | 4.49E-16 | 4.63E-15 |
| RNMT | NSUN2 | 8.42 | 0.04 | 0.36 | 4.50E-16 | 4.63E-15 |
| DKC1 | DNMT3A | 8.42 | 0.04 | 0.36 | 4.71E-16 | 4.84E-15 |
| TRMT61B | TRMT6 | 8.41 | 0.04 | 0.36 | 5.13E-16 | 5.25E-15 |
| G3BP2 | KIAA1429 | 8.40 | 0.04 | 0.36 | 5.20E-16 | 5.31E-15 |
| NSUN6 | YTHDC1 | 8.40 | 0.04 | 0.36 | 5.23E-16 | 5.34E-15 |
| TRMT44 | CMTR1 | 8.38 | 0.04 | 0.36 | 6.41E-16 | 6.52E-15 |
| FBL | TRMT6 | 8.37 | 0.04 | 0.36 | 6.49E-16 | 6.60E-15 |
| G3BP1 | ZC3H13 | 8.37 | 0.04 | 0.36 | 6.51E-16 | 6.60E-15 |
| PRRC2A | KIAA1429 | 8.37 | 0.04 | 0.36 | 6.78E-16 | 6.86E-15 |
| TET1 | DNMT1 | 8.36 | 0.04 | 0.36 | 7.13E-16 | 7.21E-15 |
| CMTR1 | HNRNPA2B1 | 8.36 | 0.04 | 0.36 | 7.46E-16 | 7.52E-15 |
| DNMT1 | KIAA1429 | 8.34 | 0.04 | 0.36 | 8.06E-16 | 8.11E-15 |
| WDR4 | MRM1 | 8.34 | 0.04 | 0.36 | 8.46E-16 | 8.49E-15 |
| RPUSD1 | NOP2 | 8.32 | 0.04 | 0.36 | 9.40E-16 | 9.42E-15 |
| FTSJ3 | DNMT3B | 8.32 | 0.04 | 0.36 | 9.47E-16 | 9.47E-15 |
| TRMT112 | HNRNPC | 8.32 | 0.04 | 0.36 | 9.52E-16 | 9.50E-15 |
| ALKBH8 | EIF3A | 8.32 | 0.04 | 0.36 | 9.62E-16 | 9.58E-15 |
| ELP1 | CBLL1 | 8.32 | 0.04 | 0.36 | 9.67E-16 | 9.61E-15 |
| TRMT13 | FMR1 | 8.30 | 0.04 | 0.36 | 1.08E-15 | 1.07E-14 |
| MRM1 | NOP2 | 8.30 | 0.04 | 0.36 | 1.13E-15 | 1.11E-14 |
| RNMT | METTL14 | 8.30 | 0.04 | 0.36 | 1.15E-15 | 1.14E-14 |
| TRMT13 | YTHDC2 | 8.29 | 0.04 | 0.36 | 1.22E-15 | 1.20E-14 |
| PUS7 | NSUN5 | 8.29 | 0.04 | 0.36 | 1.22E-15 | 1.20E-14 |
| NSUN2 | PRRC2A | 8.27 | 0.04 | 0.36 | 1.37E-15 | 1.35E-14 |
| RBMX | METTL3 | 8.26 | 0.04 | 0.36 | 1.46E-15 | 1.43E-14 |
| TRMT6 | HNRNPC | 8.25 | 0.04 | 0.36 | 1.62E-15 | 1.58E-14 |
| PUS1 | HNRNPA2B1 | 8.25 | 0.04 | 0.36 | 1.63E-15 | 1.58E-14 |
| TRMT44 | NSUN7 | 8.25 | 0.04 | 0.36 | 1.67E-15 | 1.62E-14 |
| SNORD48 | TARBP1 | 8.24 | 0.04 | 0.36 | 1.69E-15 | 1.64E-14 |
| TGS1 | RBMX | 8.24 | 0.04 | 0.36 | 1.74E-15 | 1.68E-14 |
| ALYREF | NSUN2 | 8.24 | 0.04 | 0.36 | 1.76E-15 | 1.70E-14 |
| G3BP2 | CBLL1 | 8.23 | 0.04 | 0.36 | 1.82E-15 | 1.75E-14 |
| DNMT3A | IGF2BP1 | 8.23 | 0.04 | 0.36 | 1.83E-15 | 1.76E-14 |
| RNMT | CMTR1 | 8.22 | 0.04 | 0.35 | 1.96E-15 | 1.88E-14 |
| PUS7 | PUS1 | 8.22 | 0.04 | 0.35 | 1.99E-15 | 1.91E-14 |
| DKC1 | FBL | 8.22 | 0.04 | 0.35 | 2.03E-15 | 1.95E-14 |
| METTL1 | HNRNPC | 8.21 | 0.04 | 0.35 | 2.14E-15 | 2.04E-14 |
| TRMT61B | ZCCHC4 | 8.20 | 0.04 | 0.35 | 2.28E-15 | 2.17E-14 |
| CTU2 | MRM1 | 8.20 | 0.04 | 0.35 | 2.31E-15 | 2.20E-14 |
| RPUSD2 | TRMT10C | 8.18 | 0.04 | 0.35 | 2.65E-15 | 2.51E-14 |
| PUS7 | HNRNPC | 8.18 | 0.04 | 0.35 | 2.72E-15 | 2.58E-14 |
| HNRNPC | HNRNPA1 | 8.16 | 0.04 | 0.35 | 3.01E-15 | 2.84E-14 |
| TRMT13 | CMTR1 | 8.16 | 0.04 | 0.35 | 3.05E-15 | 2.88E-14 |
| TET1 | HNRNPA2B1 | 8.16 | 0.04 | 0.35 | 3.12E-15 | 2.94E-14 |
| TRUB1 | NSUN3 | 8.14 | 0.04 | 0.35 | 3.56E-15 | 3.34E-14 |
| SNORD48 | METTL3 | 8.14 | 0.04 | 0.35 | 3.56E-15 | 3.34E-14 |
| NSUN2 | KIAA1429 | 8.14 | 0.04 | 0.35 | 3.58E-15 | 3.35E-14 |
| MRM1 | NSUN5 | 8.14 | 0.04 | 0.35 | 3.70E-15 | 3.46E-14 |
| LRPPRC | G3BP1 | 8.13 | 0.04 | 0.35 | 3.77E-15 | 3.52E-14 |
| FTSJ3 | ELAVL1 | 8.13 | 0.04 | 0.35 | 3.85E-15 | 3.59E-14 |
| CMTR2 | FTO | 8.13 | 0.04 | 0.35 | 3.90E-15 | 3.62E-14 |
| YTHDC1 | RBM15B | 8.13 | 0.04 | 0.35 | 3.90E-15 | 3.62E-14 |
| ADAT2 | CMTR1 | 8.13 | 0.04 | 0.35 | 3.91E-15 | 3.62E-14 |
| CTU1 | FBL | 8.11 | 0.04 | 0.35 | 4.49E-15 | 4.15E-14 |
| TRMT44 | YTHDC1 | 8.11 | 0.04 | 0.35 | 4.53E-15 | 4.18E-14 |
| DNMT3B | KIAA1429 | 8.10 | 0.04 | 0.35 | 4.70E-15 | 4.33E-14 |
| MRM3 | METTL16 | 8.10 | 0.04 | 0.35 | 4.75E-15 | 4.37E-14 |
| YTHDF3 | CBLL1 | 8.08 | 0.04 | 0.35 | 5.71E-15 | 5.23E-14 |
| PUS7 | WDR4 | 8.07 | 0.04 | 0.35 | 5.81E-15 | 5.31E-14 |
| DKC1 | FTSJ3 | 8.07 | 0.04 | 0.35 | 6.03E-15 | 5.50E-14 |
| TRUB1 | G3BP2 | 8.07 | 0.04 | 0.35 | 6.09E-15 | 5.54E-14 |
| TET3 | CBLL1 | 8.07 | 0.04 | 0.35 | 6.10E-15 | 5.54E-14 |
| MRM1 | METTL3 | 8.06 | 0.04 | 0.35 | 6.17E-15 | 5.60E-14 |
| DKC1 | TRMT10C | 8.06 | 0.04 | 0.35 | 6.21E-15 | 5.62E-14 |
| DNMT1 | YTHDF1 | 8.05 | 0.04 | 0.35 | 6.93E-15 | 6.26E-14 |
| RNMT | RBM15 | 8.04 | 0.04 | 0.35 | 7.61E-15 | 6.87E-14 |
| TET2 | TET1 | 8.03 | 0.04 | 0.35 | 7.65E-15 | 6.89E-14 |
| TRMT61B | NSUN3 | 8.03 | 0.04 | 0.35 | 7.75E-15 | 6.96E-14 |
| EIF3A | METTL14 | 8.03 | 0.04 | 0.35 | 8.03E-15 | 7.21E-14 |
| ADARB1 | ZC3H13 | 8.02 | 0.04 | 0.35 | 8.73E-15 | 7.82E-14 |
| DKC1 | PUS7L | 8.01 | 0.04 | 0.35 | 8.87E-15 | 7.93E-14 |
| YTHDC1 | KIAA1429 | 8.01 | 0.04 | 0.35 | 9.00E-15 | 8.03E-14 |
| ADAR | NSUN2 | 8.01 | 0.04 | 0.35 | 9.21E-15 | 8.20E-14 |
| RNMT | CBLL1 | 8.01 | 0.04 | 0.35 | 9.35E-15 | 8.32E-14 |
| RNMT | G3BP1 | 8.00 | 0.04 | 0.35 | 9.53E-15 | 8.46E-14 |
| CMTR2 | METTL14 | 8.00 | 0.04 | 0.35 | 9.82E-15 | 8.70E-14 |
| FMR1 | CBLL1 | 8.00 | 0.04 | 0.35 | 9.88E-15 | 8.74E-14 |
| TARBP1 | YTHDC1 | 8.00 | 0.04 | 0.35 | 9.95E-15 | 8.79E-14 |
| CTU2 | WDR4 | 7.98 | 0.04 | 0.35 | 1.10E-14 | 9.71E-14 |
| TRMT11 | NSUN6 | 7.98 | 0.04 | 0.35 | 1.16E-14 | 1.02E-13 |
| EIF3A | HNRNPA2B1 | 7.98 | 0.04 | 0.35 | 1.17E-14 | 1.02E-13 |
| ELP1 | METTL16 | 7.97 | 0.04 | 0.35 | 1.21E-14 | 1.06E-13 |
| WDR4 | FTSJ3 | 7.97 | 0.04 | 0.35 | 1.21E-14 | 1.06E-13 |
| TET2 | METTL16 | 7.97 | 0.04 | 0.35 | 1.22E-14 | 1.07E-13 |
| NSUN2 | ELAVL1 | 7.96 | 0.04 | 0.35 | 1.29E-14 | 1.13E-13 |
| HENMT1 | IGF2BP3 | 7.96 | 0.04 | 0.35 | 1.29E-14 | 1.13E-13 |
| TRMT10C | RBMX | 7.96 | 0.04 | 0.34 | 1.31E-14 | 1.13E-13 |
| PUS1 | FBL | 7.94 | 0.04 | 0.34 | 1.53E-14 | 1.33E-13 |
| YBX1 | METTL5 | 7.92 | 0.04 | 0.34 | 1.68E-14 | 1.46E-13 |
| TRDMT1 | NSUN6 | 7.92 | 0.04 | 0.34 | 1.70E-14 | 1.47E-13 |
| RNMT | TRMT6 | 7.92 | 0.04 | 0.34 | 1.74E-14 | 1.50E-13 |
| TRMT61B | CBLL1 | 7.92 | 0.04 | 0.34 | 1.79E-14 | 1.55E-13 |
| ADAR | RBMX | 7.90 | 0.04 | 0.34 | 1.96E-14 | 1.69E-13 |
| TET2 | EIF3A | 7.90 | 0.04 | 0.34 | 2.03E-14 | 1.74E-13 |
| RBM15 | KIAA1429 | 7.90 | 0.04 | 0.34 | 2.07E-14 | 1.77E-13 |
| TRMT6 | METTL5 | 7.89 | 0.04 | 0.34 | 2.19E-14 | 1.87E-13 |
| TARBP1 | TET3 | 7.88 | 0.04 | 0.34 | 2.24E-14 | 1.91E-13 |
| TRMT6 | YTHDF1 | 7.88 | 0.04 | 0.34 | 2.32E-14 | 1.98E-13 |
| ADAR | METTL14 | 7.88 | 0.04 | 0.34 | 2.37E-14 | 2.02E-13 |
| TRMT13 | NSUN7 | 7.88 | 0.04 | 0.34 | 2.38E-14 | 2.02E-13 |
| FBL | TRMT10C | 7.87 | 0.04 | 0.34 | 2.48E-14 | 2.11E-13 |
| RBMX | ZCCHC4 | 7.87 | 0.04 | 0.34 | 2.54E-14 | 2.15E-13 |
| ADAR | TET1 | 7.87 | 0.04 | 0.34 | 2.54E-14 | 2.15E-13 |
| ADAT2 | HNRNPA2B1 | 7.86 | 0.04 | 0.34 | 2.67E-14 | 2.26E-13 |
| CMTR1 | METTL14 | 7.85 | 0.04 | 0.34 | 2.81E-14 | 2.37E-13 |
| TRMT6 | DNMT3B | 7.84 | 0.04 | 0.34 | 3.06E-14 | 2.57E-13 |
| RNMT | PRRC2A | 7.84 | 0.04 | 0.34 | 3.16E-14 | 2.65E-13 |
| PUS1 | ELAVL1 | 7.83 | 0.04 | 0.34 | 3.28E-14 | 2.75E-13 |
| BUD23 | METTL5 | 7.83 | 0.04 | 0.34 | 3.34E-14 | 2.79E-13 |
| ADAT2 | YTHDC2 | 7.83 | 0.04 | 0.34 | 3.35E-14 | 2.80E-13 |
| CTU1 | NOP2 | 7.82 | 0.04 | 0.34 | 3.43E-14 | 2.86E-13 |
| HNRNPA1 | YTHDC1 | 7.82 | 0.04 | 0.34 | 3.52E-14 | 2.93E-13 |
| WDR4 | ALYREF | 7.82 | 0.04 | 0.34 | 3.56E-14 | 2.96E-13 |
| ALYREF | LRPPRC | 7.82 | 0.04 | 0.34 | 3.59E-14 | 2.98E-13 |
| ELP1 | PUS7L | 7.81 | 0.04 | 0.34 | 3.81E-14 | 3.15E-13 |
| ZC3H13 | RBM15B | 7.80 | 0.04 | 0.34 | 4.08E-14 | 3.36E-13 |
| ADAT3 | RPUSD1 | 7.79 | 0.04 | 0.34 | 4.25E-14 | 3.50E-13 |
| FTSJ3 | RBM15 | 7.78 | 0.04 | 0.34 | 4.52E-14 | 3.72E-13 |
| CTU1 | TRMT61A | 7.78 | 0.04 | 0.34 | 4.56E-14 | 3.75E-13 |
| ALYREF | IGF2BP2 | 7.78 | 0.04 | 0.34 | 4.69E-14 | 3.84E-13 |
| NSUN7 | METTL3 | 7.78 | 0.04 | 0.34 | 4.69E-14 | 3.84E-13 |
| DKC1 | NSUN5 | 7.77 | 0.04 | 0.34 | 5.15E-14 | 4.21E-13 |
| ALKBH8 | YTHDC1 | 7.76 | 0.04 | 0.34 | 5.35E-14 | 4.36E-13 |
| RBMX | NKAP | 7.74 | 0.04 | 0.34 | 6.02E-14 | 4.90E-13 |
| RNMT | FMR1 | 7.74 | 0.04 | 0.34 | 6.02E-14 | 4.90E-13 |
| PUS7L | DNMT3B | 7.74 | 0.04 | 0.34 | 6.05E-14 | 4.91E-13 |
| ADAR | PUS7 | 7.74 | 0.04 | 0.34 | 6.24E-14 | 5.06E-13 |
| PUS7L | YTHDF1 | 7.73 | 0.04 | 0.34 | 6.74E-14 | 5.46E-13 |
| IGF2BP2 | IGF2BP1 | 7.72 | 0.04 | 0.34 | 6.88E-14 | 5.55E-13 |
| TRMT13 | TRMT61B | 7.72 | 0.04 | 0.34 | 6.88E-14 | 5.55E-13 |
| PUS7L | TRMT13 | 7.72 | 0.04 | 0.34 | 6.97E-14 | 5.62E-13 |
| RNMT | FTSJ3 | 7.72 | 0.04 | 0.34 | 7.00E-14 | 5.63E-13 |
| PUS7 | FTSJ3 | 7.72 | 0.04 | 0.34 | 7.04E-14 | 5.65E-13 |
| PUS7 | DNMT1 | 7.71 | 0.04 | 0.34 | 7.41E-14 | 5.94E-13 |
| TRMT11 | METTL3 | 7.71 | 0.04 | 0.34 | 7.69E-14 | 6.15E-13 |
| TRMT10B | TET3 | 7.71 | 0.04 | 0.34 | 7.78E-14 | 6.21E-13 |
| PRRC2A | YTHDF1 | 7.70 | 0.04 | 0.34 | 8.12E-14 | 6.48E-13 |
| DNMT3B | LRPPRC | 7.69 | 0.04 | 0.33 | 8.84E-14 | 7.03E-13 |
| NSUN2 | YTHDF1 | 7.69 | 0.04 | 0.33 | 8.93E-14 | 7.09E-13 |
| DKC1 | G3BP1 | 7.68 | 0.04 | 0.33 | 9.60E-14 | 7.61E-13 |
| PUS7L | TRMT61B | 7.66 | 0.04 | 0.33 | 1.08E-13 | 8.51E-13 |
| NSUN3 | METTL14 | 7.65 | 0.04 | 0.33 | 1.19E-13 | 9.35E-13 |
| TRMT10B | TET2 | 7.64 | 0.04 | 0.33 | 1.20E-13 | 9.41E-13 |
| DNMT3A | FMR1 | 7.64 | 0.04 | 0.33 | 1.24E-13 | 9.77E-13 |
| NSUN6 | HNRNPA2B1 | 7.64 | 0.04 | 0.33 | 1.26E-13 | 9.91E-13 |
| RPUSD1 | METTL1 | 7.63 | 0.04 | 0.33 | 1.31E-13 | 1.02E-12 |
| DNMT3B | NSUN6 | 7.63 | 0.04 | 0.33 | 1.34E-13 | 1.05E-12 |
| TET3 | LRPPRC | 7.63 | 0.04 | 0.33 | 1.37E-13 | 1.07E-12 |
| CMTR1 | NSUN6 | 7.62 | 0.04 | 0.33 | 1.39E-13 | 1.08E-12 |
| LRPPRC | IGF2BP3 | 7.62 | 0.04 | 0.33 | 1.39E-13 | 1.08E-12 |
| RPUSD3 | TRMT112 | 7.62 | 0.04 | 0.33 | 1.40E-13 | 1.09E-12 |
| METTL1 | TRMT61A | 7.61 | 0.04 | 0.33 | 1.53E-13 | 1.19E-12 |
| CMTR1 | RBM15 | 7.60 | 0.04 | 0.33 | 1.57E-13 | 1.21E-12 |
| EIF3A | YTHDF3 | 7.59 | 0.04 | 0.33 | 1.74E-13 | 1.34E-12 |
| BUD23 | METTL1 | 7.58 | 0.04 | 0.33 | 1.83E-13 | 1.41E-12 |
| TET2 | FMR1 | 7.57 | 0.04 | 0.33 | 1.98E-13 | 1.52E-12 |
| TARBP1 | CMTR1 | 7.57 | 0.04 | 0.33 | 2.03E-13 | 1.55E-12 |
| ADAT2 | DNMT3B | 7.57 | 0.04 | 0.33 | 2.05E-13 | 1.57E-12 |
| PUS1 | METTL3 | 7.57 | 0.04 | 0.33 | 2.05E-13 | 1.57E-12 |
| PUS1 | BUD23 | 7.56 | 0.04 | 0.33 | 2.11E-13 | 1.61E-12 |
| ELP1 | G3BP1 | 7.56 | 0.04 | 0.33 | 2.13E-13 | 1.62E-12 |
| G3BP1 | RBM15 | 7.56 | 0.04 | 0.33 | 2.19E-13 | 1.67E-12 |
| ELAVL1 | RBM15B | 7.55 | 0.04 | 0.33 | 2.29E-13 | 1.74E-12 |
| HNRNPA2B1 | YTHDF1 | 7.55 | 0.04 | 0.33 | 2.30E-13 | 1.74E-12 |
| ELP1 | DNMT3A | 7.55 | 0.04 | 0.33 | 2.30E-13 | 1.74E-12 |
| RPUSD4 | LRPPRC | 7.55 | 0.04 | 0.33 | 2.33E-13 | 1.76E-12 |
| LRPPRC | RBM15 | 7.55 | 0.04 | 0.33 | 2.33E-13 | 1.76E-12 |
| YBX1 | WTAP | 7.54 | 0.04 | 0.33 | 2.44E-13 | 1.84E-12 |
| PUS10 | TRMT13 | 7.51 | 0.04 | 0.33 | 3.09E-13 | 2.31E-12 |
| DNMT3A | RBMX | 7.50 | 0.04 | 0.33 | 3.22E-13 | 2.41E-12 |
| YTHDF1 | CBLL1 | 7.49 | 0.04 | 0.33 | 3.41E-13 | 2.55E-12 |
| PUS3 | METTL5 | 7.49 | 0.04 | 0.33 | 3.47E-13 | 2.59E-12 |
| DNMT3A | IGF2BP3 | 7.49 | 0.04 | 0.33 | 3.48E-13 | 2.59E-12 |
| TET3 | IGF2BP2 | 7.48 | 0.04 | 0.33 | 3.76E-13 | 2.79E-12 |
| NOP2 | PRRC2A | 7.48 | 0.04 | 0.33 | 3.76E-13 | 2.79E-12 |
| DKC1 | PRRC2A | 7.48 | 0.04 | 0.33 | 3.81E-13 | 2.83E-12 |
| NOP2 | HNRNPA1 | 7.47 | 0.04 | 0.33 | 3.86E-13 | 2.86E-12 |
| RPUSD1 | MRM1 | 7.46 | 0.04 | 0.33 | 4.17E-13 | 3.09E-12 |
| TRMT61B | TET1 | 7.46 | 0.04 | 0.33 | 4.24E-13 | 3.13E-12 |
| ADAT3 | TRMT61A | 7.44 | 0.04 | 0.32 | 4.90E-13 | 3.61E-12 |
| PUS7 | RBM15 | 7.42 | 0.04 | 0.32 | 5.42E-13 | 3.99E-12 |
| PUS1 | METTL5 | 7.42 | 0.04 | 0.32 | 5.52E-13 | 4.06E-12 |
| FMR1 | KIAA1429 | 7.42 | 0.04 | 0.32 | 5.64E-13 | 4.14E-12 |
| DNMT1 | EIF3A | 7.41 | 0.04 | 0.32 | 5.80E-13 | 4.25E-12 |
| DKC1 | RBM15 | 7.41 | 0.04 | 0.32 | 5.85E-13 | 4.28E-12 |
| NSUN3 | CBLL1 | 7.40 | 0.04 | 0.32 | 6.47E-13 | 4.72E-12 |
| ELP1 | FMR1 | 7.40 | 0.04 | 0.32 | 6.47E-13 | 4.72E-12 |
| METTL16 | METTL14 | 7.39 | 0.04 | 0.32 | 6.71E-13 | 4.88E-12 |
| TRMT10A | YTHDF3 | 7.39 | 0.04 | 0.32 | 6.97E-13 | 5.06E-12 |
| PUS7L | IGF2BP3 | 7.38 | 0.04 | 0.32 | 7.12E-13 | 5.17E-12 |
| WDR4 | RBM15 | 7.38 | 0.04 | 0.32 | 7.22E-13 | 5.23E-12 |
| TRMT61A | NOP2 | 7.38 | 0.04 | 0.32 | 7.35E-13 | 5.31E-12 |
| TRMT6 | ELAVL1 | 7.38 | 0.04 | 0.32 | 7.45E-13 | 5.38E-12 |
| PUS7L | PUS7 | 7.37 | 0.04 | 0.32 | 7.62E-13 | 5.49E-12 |
| PUS7L | NSUN2 | 7.37 | 0.04 | 0.32 | 7.78E-13 | 5.60E-12 |
| TET1 | RBM15 | 7.36 | 0.04 | 0.32 | 8.39E-13 | 6.03E-12 |
| LRPPRC | PRRC2A | 7.36 | 0.04 | 0.32 | 8.47E-13 | 6.08E-12 |
| DNMT1 | YTHDC1 | 7.35 | 0.04 | 0.32 | 8.65E-13 | 6.19E-12 |
| TRMT11 | RBMX | 7.35 | 0.04 | 0.32 | 8.82E-13 | 6.31E-12 |
| TET3 | IGF2BP3 | 7.35 | 0.04 | 0.32 | 8.86E-13 | 6.33E-12 |
| FTSJ3 | RBM15B | 7.35 | 0.04 | 0.32 | 8.91E-13 | 6.35E-12 |
| TRMT112 | FBL | 7.35 | 0.04 | 0.32 | 9.13E-13 | 6.50E-12 |
| CMTR1 | RBMX | 7.34 | 0.04 | 0.32 | 9.32E-13 | 6.63E-12 |
| METTL1 | FTSJ1 | 7.33 | 0.04 | 0.32 | 1.03E-12 | 7.32E-12 |
| FTO | ZC3H13 | 7.33 | 0.04 | 0.32 | 1.04E-12 | 7.35E-12 |
| ADAR | DNMT3B | 7.32 | 0.04 | 0.32 | 1.06E-12 | 7.54E-12 |
| TGS1 | HNRNPC | 7.32 | 0.04 | 0.32 | 1.09E-12 | 7.69E-12 |
| FTSJ3 | NOP2 | 7.32 | 0.04 | 0.32 | 1.09E-12 | 7.73E-12 |
| NSUN2 | IGF2BP3 | 7.32 | 0.04 | 0.32 | 1.10E-12 | 7.79E-12 |
| TRMT10C | YTHDF3 | 7.32 | 0.04 | 0.32 | 1.11E-12 | 7.84E-12 |
| ADARB1 | YTHDC2 | 7.32 | 0.04 | 0.32 | 1.12E-12 | 7.88E-12 |
| TRMT6 | NSUN2 | 7.31 | 0.04 | 0.32 | 1.13E-12 | 7.93E-12 |
| RPUSD1 | FTSJ1 | 7.31 | 0.04 | 0.32 | 1.14E-12 | 8.01E-12 |
| ELP1 | HNRNPA2B1 | 7.31 | 0.04 | 0.32 | 1.18E-12 | 8.29E-12 |
| RBMX | RBM15 | 7.29 | 0.04 | 0.32 | 1.29E-12 | 9.00E-12 |
| PUS1 | IGF2BP1 | 7.29 | 0.04 | 0.32 | 1.36E-12 | 9.46E-12 |
| RBMX | KIAA1429 | 7.29 | 0.04 | 0.32 | 1.37E-12 | 9.55E-12 |
| MRM1 | FTSJ3 | 7.28 | 0.04 | 0.32 | 1.41E-12 | 9.80E-12 |
| PRRC2A | RBM15 | 7.27 | 0.04 | 0.32 | 1.47E-12 | 1.02E-11 |
| ADAR | ELAVL1 | 7.27 | 0.04 | 0.32 | 1.51E-12 | 1.05E-11 |
| PUS7 | TRUB1 | 7.27 | 0.04 | 0.32 | 1.52E-12 | 1.05E-11 |
| PUS1 | NSUN6 | 7.27 | 0.04 | 0.32 | 1.52E-12 | 1.05E-11 |
| FMR1 | YTHDC2 | 7.27 | 0.04 | 0.32 | 1.53E-12 | 1.06E-11 |
| TET1 | FMR1 | 7.27 | 0.04 | 0.32 | 1.54E-12 | 1.06E-11 |
| RNMT | G3BP2 | 7.26 | 0.04 | 0.32 | 1.61E-12 | 1.11E-11 |
| ADAR | TGS1 | 7.26 | 0.04 | 0.32 | 1.64E-12 | 1.13E-11 |
| HNRNPC | YTHDF3 | 7.25 | 0.04 | 0.32 | 1.69E-12 | 1.16E-11 |
| METTL1 | TRMT10C | 7.25 | 0.04 | 0.32 | 1.70E-12 | 1.17E-11 |
| SNORD48 | NSUN6 | 7.25 | 0.04 | 0.32 | 1.76E-12 | 1.21E-11 |
| NOP2 | IGF2BP1 | 7.23 | 0.04 | 0.32 | 1.93E-12 | 1.32E-11 |
| TGS1 | RNMT | 7.23 | 0.04 | 0.32 | 1.94E-12 | 1.32E-11 |
| DNMT3A | KIAA1429 | 7.22 | 0.04 | 0.32 | 2.08E-12 | 1.42E-11 |
| TET3 | YTHDF1 | 7.22 | 0.04 | 0.32 | 2.09E-12 | 1.42E-11 |
| PUS7L | HNRNPA2B1 | 7.22 | 0.04 | 0.32 | 2.16E-12 | 1.47E-11 |
| RPUSD4 | PUS7 | 7.22 | 0.04 | 0.32 | 2.17E-12 | 1.47E-11 |
| YTHDF1 | RBM15 | 7.22 | 0.04 | 0.32 | 2.18E-12 | 1.47E-11 |
| CTU1 | NSUN5 | 7.21 | 0.04 | 0.32 | 2.21E-12 | 1.50E-11 |
| NSUN6 | RBM15 | 7.21 | 0.04 | 0.32 | 2.23E-12 | 1.51E-11 |
| TET2 | RBM15 | 7.21 | 0.04 | 0.32 | 2.25E-12 | 1.52E-11 |
| NSUN5 | METTL5 | 7.21 | 0.04 | 0.32 | 2.25E-12 | 1.52E-11 |
| CMTR2 | G3BP2 | 7.19 | 0.04 | 0.31 | 2.64E-12 | 1.77E-11 |
| TRMT13 | HNRNPA2B1 | 7.18 | 0.04 | 0.31 | 2.68E-12 | 1.80E-11 |
| BUD23 | TRMT61A | 7.18 | 0.04 | 0.31 | 2.72E-12 | 1.82E-11 |
| TRMT10C | WTAP | 7.18 | 0.04 | 0.31 | 2.83E-12 | 1.89E-11 |
| ALKBH8 | TET3 | 7.17 | 0.04 | 0.31 | 2.91E-12 | 1.94E-11 |
| TRMT6 | IGF2BP3 | 7.17 | 0.04 | 0.31 | 2.92E-12 | 1.95E-11 |
| PUS7L | ZC3H13 | 7.16 | 0.04 | 0.31 | 3.07E-12 | 2.05E-11 |
| ALKBH8 | CBLL1 | 7.16 | 0.04 | 0.31 | 3.14E-12 | 2.09E-11 |
| MRM2 | DNMT1 | 7.16 | 0.04 | 0.31 | 3.15E-12 | 2.09E-11 |
| METTL1 | DNMT3B | 7.15 | 0.04 | 0.31 | 3.28E-12 | 2.18E-11 |
| CBLL1 | RBM15 | 7.15 | 0.04 | 0.31 | 3.30E-12 | 2.19E-11 |
| TRDMT1 | METTL3 | 7.15 | 0.04 | 0.31 | 3.41E-12 | 2.26E-11 |
| NSUN5 | NSUN2 | 7.14 | 0.04 | 0.31 | 3.52E-12 | 2.33E-11 |
| EIF3A | RBM15 | 7.14 | 0.04 | 0.31 | 3.57E-12 | 2.36E-11 |
| ALYREF | NOP2 | 7.13 | 0.04 | 0.31 | 3.75E-12 | 2.47E-11 |
| DKC1 | YBX1 | 7.12 | 0.04 | 0.31 | 3.95E-12 | 2.60E-11 |
| HNRNPC | WTAP | 7.12 | 0.04 | 0.31 | 3.96E-12 | 2.60E-11 |
| TRMT10B | NSUN7 | 7.12 | 0.04 | 0.31 | 4.00E-12 | 2.62E-11 |
| HNRNPA2B1 | RBM15B | 7.12 | 0.04 | 0.31 | 4.01E-12 | 2.63E-11 |
| CTU2 | METTL5 | 7.11 | 0.04 | 0.31 | 4.23E-12 | 2.77E-11 |
| CBLL1 | METTL14 | 7.11 | 0.04 | 0.31 | 4.30E-12 | 2.81E-11 |
| G3BP2 | EIF3A | 7.11 | 0.04 | 0.31 | 4.34E-12 | 2.84E-11 |
| DKC1 | MRM2 | 7.10 | 0.04 | 0.31 | 4.69E-12 | 3.06E-11 |
| CMTR1 | TET2 | 7.09 | 0.04 | 0.31 | 4.93E-12 | 3.22E-11 |
| ELP1 | CMTR1 | 7.09 | 0.04 | 0.31 | 5.04E-12 | 3.28E-11 |
| RNMT | RBM15B | 7.09 | 0.04 | 0.31 | 5.09E-12 | 3.31E-11 |
| PUS7L | G3BP2 | 7.08 | 0.04 | 0.31 | 5.45E-12 | 3.53E-11 |
| DKC1 | YTHDF1 | 7.08 | 0.04 | 0.31 | 5.45E-12 | 3.53E-11 |
| WDR4 | PRRC2A | 7.07 | 0.04 | 0.31 | 5.60E-12 | 3.62E-11 |
| NSUN3 | LRPPRC | 7.06 | 0.04 | 0.31 | 6.19E-12 | 4.01E-11 |
| RBMX | CBLL1 | 7.05 | 0.04 | 0.31 | 6.36E-12 | 4.11E-11 |
| DNMT1 | RBM15B | 7.05 | 0.04 | 0.31 | 6.42E-12 | 4.14E-11 |
| ALKBH8 | YTHDC2 | 7.05 | 0.04 | 0.31 | 6.64E-12 | 4.27E-11 |
| TET3 | METTL14 | 7.04 | 0.04 | 0.31 | 6.73E-12 | 4.32E-11 |
| TRMT112 | BUD23 | 7.04 | 0.04 | 0.31 | 6.74E-12 | 4.32E-11 |
| RPUSD1 | BUD23 | 7.03 | 0.04 | 0.31 | 7.30E-12 | 4.67E-11 |
| NSUN2 | CBLL1 | 7.03 | 0.04 | 0.31 | 7.34E-12 | 4.69E-11 |
| ZC3H13 | KIAA1429 | 7.02 | 0.04 | 0.31 | 7.96E-12 | 5.08E-11 |
| METTL1 | NSUN2 | 7.02 | 0.04 | 0.31 | 8.03E-12 | 5.12E-11 |
| TRMT61B | TET3 | 7.01 | 0.04 | 0.31 | 8.41E-12 | 5.36E-11 |
| NSUN7 | YTHDC1 | 7.01 | 0.04 | 0.31 | 8.56E-12 | 5.45E-11 |
| PUS7 | METTL5 | 7.00 | 0.04 | 0.31 | 9.14E-12 | 5.81E-11 |
| ADAT2 | DNMT3A | 6.99 | 0.04 | 0.31 | 9.29E-12 | 5.90E-11 |
| TET3 | ELAVL1 | 6.99 | 0.04 | 0.31 | 9.70E-12 | 6.14E-11 |
| TRUB1 | TRMT6 | 6.98 | 0.04 | 0.31 | 9.97E-12 | 6.31E-11 |
| ALKBH8 | RNMT | 6.98 | 0.04 | 0.31 | 1.02E-11 | 6.42E-11 |
| DNMT3B | IGF2BP2 | 6.98 | 0.04 | 0.31 | 1.02E-11 | 6.44E-11 |
| TET2 | NSUN3 | 6.97 | 0.04 | 0.31 | 1.10E-11 | 6.92E-11 |
| TRMT61B | DNMT3B | 6.96 | 0.04 | 0.31 | 1.12E-11 | 7.06E-11 |
| CTU2 | RPUSD3 | 6.96 | 0.04 | 0.31 | 1.12E-11 | 7.06E-11 |
| TRMT10B | FMR1 | 6.96 | 0.04 | 0.31 | 1.13E-11 | 7.09E-11 |
| PRRC2A | CBLL1 | 6.96 | 0.04 | 0.31 | 1.19E-11 | 7.46E-11 |
| DKC1 | HNRNPA1 | 6.95 | 0.04 | 0.31 | 1.19E-11 | 7.48E-11 |
| DNMT1 | RBMX | 6.95 | 0.04 | 0.31 | 1.21E-11 | 7.55E-11 |
| TGS1 | NSUN3 | 6.95 | 0.04 | 0.31 | 1.21E-11 | 7.59E-11 |
| ELP1 | LRPPRC | 6.95 | 0.04 | 0.31 | 1.23E-11 | 7.66E-11 |
| ALKBH8 | KIAA1429 | 6.95 | 0.04 | 0.31 | 1.26E-11 | 7.81E-11 |
| DNMT3B | YTHDF1 | 6.95 | 0.04 | 0.31 | 1.26E-11 | 7.81E-11 |
| YTHDC1 | YTHDF2 | 6.94 | 0.04 | 0.31 | 1.32E-11 | 8.17E-11 |
| NSUN6 | RBMX | 6.94 | 0.04 | 0.31 | 1.33E-11 | 8.24E-11 |
| DNMT1 | YTHDC2 | 6.94 | 0.04 | 0.31 | 1.33E-11 | 8.25E-11 |
| PUS3 | TRMT10C | 6.94 | 0.04 | 0.31 | 1.33E-11 | 8.25E-11 |
| YTHDC1 | YTHDF1 | 6.94 | 0.04 | 0.31 | 1.34E-11 | 8.26E-11 |
| DKC1 | FTSJ1 | 6.93 | 0.04 | 0.30 | 1.39E-11 | 8.59E-11 |
| EIF3A | METTL16 | 6.93 | 0.04 | 0.30 | 1.43E-11 | 8.80E-11 |
| TRMT13 | ZCCHC4 | 6.92 | 0.04 | 0.30 | 1.45E-11 | 8.93E-11 |
| TET3 | RBMX | 6.92 | 0.04 | 0.30 | 1.53E-11 | 9.40E-11 |
| G3BP1 | HNRNPA1 | 6.91 | 0.04 | 0.30 | 1.54E-11 | 9.45E-11 |
| ALKBH8 | CMTR1 | 6.91 | 0.04 | 0.30 | 1.54E-11 | 9.46E-11 |
| IGF2BP3 | WTAP | 6.91 | 0.04 | 0.30 | 1.58E-11 | 9.63E-11 |
| YTHDF1 | RBM15B | 6.90 | 0.04 | 0.30 | 1.66E-11 | 1.01E-10 |
| LRPPRC | G3BP2 | 6.90 | 0.04 | 0.30 | 1.69E-11 | 1.03E-10 |
| RBMX | WTAP | 6.90 | 0.04 | 0.30 | 1.71E-11 | 1.04E-10 |
| MRM1 | DNMT3A | 6.89 | 0.04 | 0.30 | 1.80E-11 | 1.09E-10 |
| TET3 | NSUN6 | 6.89 | 0.04 | 0.30 | 1.81E-11 | 1.10E-10 |
| TRMT10B | DNMT3A | 6.89 | 0.04 | 0.30 | 1.86E-11 | 1.13E-10 |
| G3BP1 | YTHDC1 | 6.88 | 0.04 | 0.30 | 1.87E-11 | 1.14E-10 |
| TRMT6 | CBLL1 | 6.88 | 0.04 | 0.30 | 1.94E-11 | 1.18E-10 |
| MRM2 | FTSJ3 | 6.88 | 0.04 | 0.30 | 1.97E-11 | 1.19E-10 |
| ADAT3 | ELAVL1 | 6.87 | 0.04 | 0.30 | 2.03E-11 | 1.23E-10 |
| FTO | METTL14 | 6.87 | 0.04 | 0.30 | 2.11E-11 | 1.27E-10 |
| ALKBH8 | G3BP2 | 6.86 | 0.04 | 0.30 | 2.13E-11 | 1.28E-10 |
| ADAT3 | PUS1 | 6.86 | 0.04 | 0.30 | 2.14E-11 | 1.29E-10 |
| FMR1 | YTHDF1 | 6.86 | 0.04 | 0.30 | 2.14E-11 | 1.29E-10 |
| PUS7 | ELAVL1 | 6.85 | 0.04 | 0.30 | 2.30E-11 | 1.38E-10 |
| NSUN7 | ZCCHC4 | 6.85 | 0.04 | 0.30 | 2.30E-11 | 1.38E-10 |
| TET2 | NSUN4 | 6.85 | 0.04 | 0.30 | 2.31E-11 | 1.38E-10 |
| TRMT112 | NSUN5 | 6.85 | 0.04 | 0.30 | 2.31E-11 | 1.38E-10 |
| PUS7 | DNMT3A | 6.84 | 0.04 | 0.30 | 2.48E-11 | 1.48E-10 |
| HENMT1 | YBX1 | 6.84 | 0.04 | 0.30 | 2.48E-11 | 1.48E-10 |
| ZCCHC4 | METTL14 | 6.83 | 0.04 | 0.30 | 2.62E-11 | 1.56E-10 |
| TGS1 | TRMT10C | 6.83 | 0.04 | 0.30 | 2.63E-11 | 1.56E-10 |
| PUS7 | YTHDF1 | 6.83 | 0.04 | 0.30 | 2.65E-11 | 1.57E-10 |
| BUD23 | MRM1 | 6.83 | 0.04 | 0.30 | 2.67E-11 | 1.58E-10 |
| PUS7L | ELAVL1 | 6.82 | 0.04 | 0.30 | 2.80E-11 | 1.66E-10 |
| TET3 | YTHDC2 | 6.82 | 0.04 | 0.30 | 2.87E-11 | 1.70E-10 |
| PUS7 | YTHDF3 | 6.82 | 0.04 | 0.30 | 2.88E-11 | 1.70E-10 |
| TET2 | DNMT3A | 6.81 | 0.04 | 0.30 | 2.91E-11 | 1.71E-10 |
| PUS10 | RRP8 | 6.81 | 0.04 | 0.30 | 2.92E-11 | 1.72E-10 |
| TGS1 | PRRC2A | 6.81 | 0.04 | 0.30 | 2.99E-11 | 1.76E-10 |
| PUS7 | PRRC2A | 6.81 | 0.04 | 0.30 | 2.99E-11 | 1.76E-10 |
| TRMT10B | TRDMT1 | 6.81 | 0.04 | 0.30 | 3.05E-11 | 1.79E-10 |
| G3BP2 | ZC3H13 | 6.81 | 0.04 | 0.30 | 3.06E-11 | 1.79E-10 |
| FBL | DNMT3B | 6.80 | 0.04 | 0.30 | 3.15E-11 | 1.84E-10 |
| LRPPRC | ZCCHC4 | 6.80 | 0.04 | 0.30 | 3.20E-11 | 1.87E-10 |
| ALKBH8 | PUS3 | 6.80 | 0.04 | 0.30 | 3.22E-11 | 1.88E-10 |
| CTU2 | CTU1 | 6.80 | 0.04 | 0.30 | 3.29E-11 | 1.91E-10 |
| FBL | NOP2 | 6.79 | 0.04 | 0.30 | 3.38E-11 | 1.97E-10 |
| FTSJ3 | YTHDF1 | 6.79 | 0.04 | 0.30 | 3.46E-11 | 2.01E-10 |
| FTSJ1 | METTL5 | 6.78 | 0.04 | 0.30 | 3.65E-11 | 2.12E-10 |
| CBLL1 | ZC3H13 | 6.77 | 0.04 | 0.30 | 3.90E-11 | 2.26E-10 |
| RPUSD3 | RPUSD2 | 6.76 | 0.04 | 0.30 | 4.05E-11 | 2.34E-10 |
| TET1 | NSUN6 | 6.75 | 0.04 | 0.30 | 4.36E-11 | 2.52E-10 |
| RNMT | YTHDC2 | 6.74 | 0.04 | 0.30 | 4.78E-11 | 2.76E-10 |
| NKAP | HNRNPA2B1 | 6.74 | 0.04 | 0.30 | 4.79E-11 | 2.76E-10 |
| TRMT6 | DNMT3A | 6.73 | 0.04 | 0.30 | 4.85E-11 | 2.79E-10 |
| ADARB2 | TET2 | 6.73 | 0.04 | 0.30 | 5.04E-11 | 2.89E-10 |
| PUS7 | G3BP1 | 6.73 | 0.04 | 0.30 | 5.06E-11 | 2.91E-10 |
| DKC1 | TET1 | 6.72 | 0.04 | 0.30 | 5.16E-11 | 2.96E-10 |
| DNMT1 | NSUN5 | 6.72 | 0.04 | 0.30 | 5.29E-11 | 3.03E-10 |
| TRMT13 | YTHDC1 | 6.71 | 0.04 | 0.30 | 5.63E-11 | 3.22E-10 |
| ELP1 | KIAA1429 | 6.70 | 0.04 | 0.30 | 5.81E-11 | 3.32E-10 |
| METTL1 | FBL | 6.70 | 0.04 | 0.30 | 5.89E-11 | 3.36E-10 |
| TET2 | RBM15B | 6.70 | 0.04 | 0.30 | 5.99E-11 | 3.41E-10 |
| WDR4 | ELAVL1 | 6.70 | 0.04 | 0.30 | 6.00E-11 | 3.42E-10 |
| YBX1 | LRPPRC | 6.70 | 0.04 | 0.30 | 6.04E-11 | 3.43E-10 |
| TARBP1 | DNMT3B | 6.70 | 0.04 | 0.30 | 6.04E-11 | 3.43E-10 |
| RBMX | YTHDC2 | 6.70 | 0.04 | 0.30 | 6.06E-11 | 3.44E-10 |
| SNORD48 | DNMT3A | 6.70 | 0.04 | 0.30 | 6.12E-11 | 3.47E-10 |
| TRMT10B | RBM15 | 6.69 | 0.04 | 0.30 | 6.41E-11 | 3.63E-10 |
| FTSJ3 | CBLL1 | 6.69 | 0.04 | 0.30 | 6.46E-11 | 3.65E-10 |
| TRMT61A | YTHDF1 | 6.69 | 0.04 | 0.30 | 6.50E-11 | 3.67E-10 |
| DNMT3B | NSUN5 | 6.69 | 0.04 | 0.29 | 6.56E-11 | 3.70E-10 |
| MRM1 | TARBP1 | 6.68 | 0.04 | 0.29 | 6.58E-11 | 3.71E-10 |
| RNMT | DNMT3B | 6.68 | 0.04 | 0.29 | 6.75E-11 | 3.80E-10 |
| TRMT44 | TRMT10B | 6.68 | 0.04 | 0.29 | 6.84E-11 | 3.84E-10 |
| TET1 | YTHDF3 | 6.68 | 0.04 | 0.29 | 6.94E-11 | 3.90E-10 |
| PUS7 | FBL | 6.68 | 0.04 | 0.29 | 6.95E-11 | 3.90E-10 |
| PUS7 | HNRNPA2B1 | 6.67 | 0.04 | 0.29 | 7.13E-11 | 3.99E-10 |
| TET2 | G3BP2 | 6.67 | 0.04 | 0.29 | 7.13E-11 | 3.99E-10 |
| CTU1 | ELAVL1 | 6.67 | 0.04 | 0.29 | 7.28E-11 | 4.07E-10 |
| ELP1 | NSUN3 | 6.67 | 0.04 | 0.29 | 7.31E-11 | 4.08E-10 |
| ALKBH1 | METTL3 | 6.67 | 0.04 | 0.29 | 7.38E-11 | 4.12E-10 |
| DNMT3A | EIF3A | 6.66 | 0.04 | 0.29 | 7.45E-11 | 4.15E-10 |
| WDR4 | MRM2 | 6.66 | 0.04 | 0.29 | 7.68E-11 | 4.28E-10 |
| MRM3 | MRM1 | 6.66 | 0.04 | 0.29 | 7.80E-11 | 4.34E-10 |
| TGS1 | G3BP2 | 6.65 | 0.04 | 0.29 | 8.02E-11 | 4.46E-10 |
| ALKBH8 | TGS1 | 6.65 | 0.04 | 0.29 | 8.05E-11 | 4.47E-10 |
| ELP1 | NSUN2 | 6.65 | 0.04 | 0.29 | 8.37E-11 | 4.64E-10 |
| ADAR | DKC1 | 6.65 | 0.04 | 0.29 | 8.38E-11 | 4.64E-10 |
| MRM1 | DNMT3B | 6.64 | 0.04 | 0.29 | 8.50E-11 | 4.70E-10 |
| TET1 | IGF2BP3 | 6.64 | 0.04 | 0.29 | 8.74E-11 | 4.83E-10 |
| ADARB1 | TET3 | 6.64 | 0.04 | 0.29 | 8.89E-11 | 4.90E-10 |
| PUS7 | TET1 | 6.64 | 0.04 | 0.29 | 8.95E-11 | 4.93E-10 |
| TRMT61B | HNRNPC | 6.63 | 0.04 | 0.29 | 9.02E-11 | 4.97E-10 |
| ELP1 | NSUN6 | 6.63 | 0.04 | 0.29 | 9.04E-11 | 4.98E-10 |
| LRPPRC | METTL5 | 6.63 | 0.04 | 0.29 | 9.38E-11 | 5.15E-10 |
| TRMT13 | NSUN4 | 6.62 | 0.04 | 0.29 | 9.58E-11 | 5.26E-10 |
| TRMT10A | G3BP2 | 6.62 | 0.04 | 0.29 | 9.89E-11 | 5.42E-10 |
| IGF2BP3 | CBLL1 | 6.62 | 0.04 | 0.29 | 1.00E-10 | 5.47E-10 |
| PUS1 | PRRC2A | 6.62 | 0.04 | 0.29 | 1.00E-10 | 5.49E-10 |
| TRMT10B | RBMX | 6.62 | 0.04 | 0.29 | 1.01E-10 | 5.49E-10 |
| YTHDC2 | ZC3H13 | 6.61 | 0.04 | 0.29 | 1.05E-10 | 5.70E-10 |
| FTSJ3 | YTHDC1 | 6.61 | 0.04 | 0.29 | 1.06E-10 | 5.76E-10 |
| RBMX | METTL14 | 6.60 | 0.04 | 0.29 | 1.11E-10 | 6.04E-10 |
| TET3 | NOP2 | 6.60 | 0.04 | 0.29 | 1.12E-10 | 6.09E-10 |
| IGF2BP3 | KIAA1429 | 6.60 | 0.04 | 0.29 | 1.12E-10 | 6.09E-10 |
| FTSJ3 | KIAA1429 | 6.60 | 0.04 | 0.29 | 1.13E-10 | 6.14E-10 |
| TRMT13 | ALKBH1 | 6.59 | 0.04 | 0.29 | 1.21E-10 | 6.55E-10 |
| RPUSD2 | TRMT112 | 6.58 | 0.04 | 0.29 | 1.23E-10 | 6.63E-10 |
| RBM15 | METTL3 | 6.58 | 0.04 | 0.29 | 1.26E-10 | 6.82E-10 |
| ALKBH8 | G3BP1 | 6.57 | 0.04 | 0.29 | 1.31E-10 | 7.08E-10 |
| TRMT61B | IGF2BP3 | 6.57 | 0.04 | 0.29 | 1.33E-10 | 7.15E-10 |
| TRMT11 | TRMT10B | 6.57 | 0.04 | 0.29 | 1.34E-10 | 7.20E-10 |
| HNRNPA2B1 | ZC3H13 | 6.57 | 0.04 | 0.29 | 1.36E-10 | 7.28E-10 |
| ALYREF | HNRNPA2B1 | 6.57 | 0.04 | 0.29 | 1.37E-10 | 7.36E-10 |
| PUS7 | METTL1 | 6.56 | 0.04 | 0.29 | 1.41E-10 | 7.53E-10 |
| RNMT | HNRNPA2B1 | 6.56 | 0.04 | 0.29 | 1.43E-10 | 7.63E-10 |
| TRUB1 | CBLL1 | 6.55 | 0.04 | 0.29 | 1.51E-10 | 8.08E-10 |
| PUS1 | MRM3 | 6.55 | 0.04 | 0.29 | 1.54E-10 | 8.22E-10 |
| CTU2 | MRM3 | 6.54 | 0.04 | 0.29 | 1.60E-10 | 8.51E-10 |
| WDR4 | DNMT3A | 6.53 | 0.04 | 0.29 | 1.70E-10 | 9.04E-10 |
| PUS7 | PUS3 | 6.53 | 0.04 | 0.29 | 1.72E-10 | 9.16E-10 |
| NSUN5 | ELAVL1 | 6.52 | 0.04 | 0.29 | 1.79E-10 | 9.48E-10 |
| ELAVL1 | RBM15 | 6.52 | 0.04 | 0.29 | 1.79E-10 | 9.48E-10 |
| PUS7L | NSUN3 | 6.52 | 0.04 | 0.29 | 1.80E-10 | 9.55E-10 |
| PUS1 | TRMT10C | 6.52 | 0.04 | 0.29 | 1.81E-10 | 9.56E-10 |
| DNMT1 | NOP2 | 6.52 | 0.04 | 0.29 | 1.81E-10 | 9.57E-10 |
| NSUN3 | RBMX | 6.52 | 0.04 | 0.29 | 1.82E-10 | 9.62E-10 |
| ALKBH8 | RBM15 | 6.52 | 0.04 | 0.29 | 1.85E-10 | 9.76E-10 |
| CTU2 | RPUSD2 | 6.51 | 0.04 | 0.29 | 1.91E-10 | 1.01E-09 |
| PUS10 | TARBP1 | 6.51 | 0.04 | 0.29 | 1.93E-10 | 1.02E-09 |
| FTSJ3 | LRPPRC | 6.50 | 0.04 | 0.29 | 2.01E-10 | 1.06E-09 |
| NSUN2 | EIF3A | 6.50 | 0.04 | 0.29 | 2.02E-10 | 1.06E-09 |
| YTHDF2 | METTL14 | 6.50 | 0.04 | 0.29 | 2.03E-10 | 1.07E-09 |
| YTHDF2 | RBM15 | 6.50 | 0.04 | 0.29 | 2.04E-10 | 1.07E-09 |
| ALKBH8 | ADAR | 6.50 | 0.04 | 0.29 | 2.05E-10 | 1.07E-09 |
| PRRC2A | HNRNPA1 | 6.49 | 0.04 | 0.29 | 2.13E-10 | 1.11E-09 |
| TET2 | RBMX | 6.49 | 0.04 | 0.29 | 2.15E-10 | 1.12E-09 |
| PUS10 | YTHDC2 | 6.48 | 0.04 | 0.29 | 2.34E-10 | 1.22E-09 |
| TET3 | NSUN3 | 6.48 | 0.04 | 0.29 | 2.34E-10 | 1.22E-09 |
| DNMT1 | IGF2BP2 | 6.47 | 0.04 | 0.29 | 2.44E-10 | 1.27E-09 |
| CTU2 | DKC1 | 6.47 | 0.04 | 0.29 | 2.45E-10 | 1.27E-09 |
| TET1 | METTL3 | 6.47 | 0.04 | 0.29 | 2.45E-10 | 1.27E-09 |
| TRUB1 | TET1 | 6.47 | 0.04 | 0.29 | 2.45E-10 | 1.27E-09 |
| HNRNPA2B1 | HNRNPA1 | 6.47 | 0.04 | 0.29 | 2.48E-10 | 1.29E-09 |
| TRMT13 | DNMT3A | 6.47 | 0.04 | 0.29 | 2.53E-10 | 1.31E-09 |
| TARBP1 | TET2 | 6.47 | 0.04 | 0.29 | 2.53E-10 | 1.31E-09 |
| G3BP1 | YTHDF3 | 6.46 | 0.04 | 0.29 | 2.57E-10 | 1.33E-09 |
| CMTR1 | ZC3H13 | 6.46 | 0.04 | 0.29 | 2.58E-10 | 1.33E-09 |
| TRMT6 | RBM15 | 6.46 | 0.04 | 0.29 | 2.65E-10 | 1.37E-09 |
| PUS7 | BUD23 | 6.45 | 0.04 | 0.29 | 2.76E-10 | 1.42E-09 |
| TRMT11 | FMR1 | 6.45 | 0.04 | 0.29 | 2.80E-10 | 1.44E-09 |
| NSUN4 | METTL14 | 6.45 | 0.04 | 0.29 | 2.81E-10 | 1.45E-09 |
| YTHDC2 | RBM15 | 6.44 | 0.04 | 0.29 | 2.97E-10 | 1.52E-09 |
| DKC1 | TRMT61A | 6.44 | 0.04 | 0.28 | 2.99E-10 | 1.54E-09 |
| TET1 | RBM15B | 6.44 | 0.04 | 0.28 | 3.04E-10 | 1.56E-09 |
| SNORD48 | CMTR1 | 6.43 | 0.04 | 0.28 | 3.10E-10 | 1.59E-09 |
| ELP1 | TARBP1 | 6.43 | 0.04 | 0.28 | 3.11E-10 | 1.59E-09 |
| NSUN5 | METTL3 | 6.43 | 0.04 | 0.28 | 3.15E-10 | 1.61E-09 |
| MRM2 | ALYREF | 6.43 | 0.04 | 0.28 | 3.20E-10 | 1.63E-09 |
| TRMT61B | METTL5 | 6.42 | 0.04 | 0.28 | 3.38E-10 | 1.73E-09 |
| RBM15 | METTL14 | 6.42 | 0.04 | 0.28 | 3.43E-10 | 1.75E-09 |
| NSUN3 | G3BP2 | 6.41 | 0.04 | 0.28 | 3.51E-10 | 1.79E-09 |
| HNRNPC | KIAA1429 | 6.41 | 0.04 | 0.28 | 3.53E-10 | 1.80E-09 |
| NSUN3 | YTHDF3 | 6.41 | 0.04 | 0.28 | 3.54E-10 | 1.80E-09 |
| TRMT61A | ALYREF | 6.40 | 0.04 | 0.28 | 3.74E-10 | 1.89E-09 |
| ALYREF | DNMT3A | 6.40 | 0.04 | 0.28 | 3.83E-10 | 1.94E-09 |
| WDR4 | TET3 | 6.39 | 0.04 | 0.28 | 4.02E-10 | 2.03E-09 |
| TRUB1 | G3BP1 | 6.38 | 0.04 | 0.28 | 4.33E-10 | 2.18E-09 |
| RPUSD2 | RPUSD1 | 6.38 | 0.04 | 0.28 | 4.36E-10 | 2.20E-09 |
| NOP2 | EIF3A | 6.37 | 0.04 | 0.28 | 4.43E-10 | 2.23E-09 |
| TGS1 | DNMT1 | 6.37 | 0.04 | 0.28 | 4.56E-10 | 2.30E-09 |
| ADAR | TET2 | 6.37 | 0.04 | 0.28 | 4.57E-10 | 2.30E-09 |
| TGS1 | TRMT6 | 6.37 | 0.04 | 0.28 | 4.62E-10 | 2.32E-09 |
| TRMT6 | HNRNPA2B1 | 6.37 | 0.04 | 0.28 | 4.64E-10 | 2.33E-09 |
| WDR4 | FTSJ1 | 6.36 | 0.04 | 0.28 | 4.66E-10 | 2.33E-09 |
| ALKBH8 | DNMT1 | 6.35 | 0.04 | 0.28 | 4.95E-10 | 2.48E-09 |
| PUS1 | TARBP1 | 6.35 | 0.04 | 0.28 | 4.96E-10 | 2.48E-09 |
| TRMT10A | RBMX | 6.35 | 0.04 | 0.28 | 4.99E-10 | 2.49E-09 |
| TET3 | METTL16 | 6.34 | 0.04 | 0.28 | 5.28E-10 | 2.63E-09 |
| TRMT10C | CBLL1 | 6.34 | 0.04 | 0.28 | 5.32E-10 | 2.65E-09 |
| TRMT112 | YBX1 | 6.34 | 0.04 | 0.28 | 5.44E-10 | 2.70E-09 |
| FBL | NSUN5 | 6.34 | 0.04 | 0.28 | 5.46E-10 | 2.71E-09 |
| PUS7 | TRMT10A | 6.34 | 0.04 | 0.28 | 5.48E-10 | 2.72E-09 |
| FTSJ1 | NSUN2 | 6.33 | 0.04 | 0.28 | 5.64E-10 | 2.79E-09 |
| YTHDC1 | METTL3 | 6.33 | 0.04 | 0.28 | 5.74E-10 | 2.84E-09 |
| TET2 | TRDMT1 | 6.33 | 0.04 | 0.28 | 5.84E-10 | 2.89E-09 |
| TRMT13 | TET3 | 6.33 | 0.04 | 0.28 | 5.89E-10 | 2.91E-09 |
| TGS1 | RBM15 | 6.32 | 0.04 | 0.28 | 5.94E-10 | 2.93E-09 |
| RPUSD3 | TRMT61A | 6.31 | 0.04 | 0.28 | 6.41E-10 | 3.15E-09 |
| TRMT44 | TET3 | 6.31 | 0.04 | 0.28 | 6.61E-10 | 3.25E-09 |
| ELAVL1 | CBLL1 | 6.30 | 0.04 | 0.28 | 6.67E-10 | 3.27E-09 |
| ELP1 | TET1 | 6.30 | 0.04 | 0.28 | 6.87E-10 | 3.37E-09 |
| CTU2 | FBL | 6.30 | 0.04 | 0.28 | 7.05E-10 | 3.45E-09 |
| HENMT1 | WTAP | 6.29 | 0.04 | 0.28 | 7.21E-10 | 3.53E-09 |
| DKC1 | PUS3 | 6.29 | 0.04 | 0.28 | 7.22E-10 | 3.53E-09 |
| NSUN4 | RBMX | 6.29 | 0.04 | 0.28 | 7.25E-10 | 3.54E-09 |
| YTHDF2 | RBM15B | 6.29 | 0.04 | 0.28 | 7.37E-10 | 3.59E-09 |
| TRMT10A | METTL5 | 6.29 | 0.04 | 0.28 | 7.48E-10 | 3.65E-09 |
| PUS7L | YTHDC2 | 6.28 | 0.04 | 0.28 | 7.77E-10 | 3.78E-09 |
| PUS1 | FTSJ1 | 6.28 | 0.04 | 0.28 | 7.95E-10 | 3.87E-09 |
| METTL1 | TRMT6 | 6.27 | 0.04 | 0.28 | 7.97E-10 | 3.87E-09 |
| RPUSD3 | PUS1 | 6.27 | 0.04 | 0.28 | 8.31E-10 | 4.03E-09 |
| HENMT1 | ALYREF | 6.26 | 0.04 | 0.28 | 8.46E-10 | 4.10E-09 |
| TARBP1 | TRMT44 | 6.25 | 0.04 | 0.28 | 9.08E-10 | 4.39E-09 |
| ALKBH1 | HNRNPC | 6.24 | 0.04 | 0.28 | 9.76E-10 | 4.72E-09 |
| ELP1 | RBM15B | 6.24 | 0.04 | 0.28 | 1.00E-09 | 4.83E-09 |
| ADAT2 | TET3 | 6.23 | 0.04 | 0.28 | 1.04E-09 | 5.02E-09 |
| DNMT3A | ZC3H13 | 6.23 | 0.04 | 0.28 | 1.05E-09 | 5.04E-09 |
| ALKBH1 | WTAP | 6.23 | 0.04 | 0.28 | 1.05E-09 | 5.07E-09 |
| FTSJ1 | YBX1 | 6.22 | 0.04 | 0.28 | 1.08E-09 | 5.17E-09 |
| TRMT6 | NOP2 | 6.22 | 0.04 | 0.28 | 1.09E-09 | 5.24E-09 |
| NSUN3 | ZC3H13 | 6.21 | 0.04 | 0.28 | 1.14E-09 | 5.48E-09 |
| NOP2 | LRPPRC | 6.21 | 0.04 | 0.28 | 1.17E-09 | 5.58E-09 |
| TRMT44 | ZCCHC4 | 6.20 | 0.04 | 0.28 | 1.26E-09 | 6.03E-09 |
| HNRNPA2B1 | CBLL1 | 6.19 | 0.04 | 0.28 | 1.28E-09 | 6.09E-09 |
| WDR4 | FBL | 6.18 | 0.04 | 0.27 | 1.39E-09 | 6.64E-09 |
| TRMT112 | TRMT10C | 6.18 | 0.04 | 0.27 | 1.43E-09 | 6.83E-09 |
| ALYREF | CBLL1 | 6.17 | 0.04 | 0.27 | 1.44E-09 | 6.83E-09 |
| NSUN3 | YTHDC1 | 6.17 | 0.04 | 0.27 | 1.44E-09 | 6.84E-09 |
| WDR4 | METTL3 | 6.17 | 0.04 | 0.27 | 1.48E-09 | 7.03E-09 |
| ADARB2 | TET3 | 6.16 | 0.04 | 0.27 | 1.54E-09 | 7.32E-09 |
| ALKBH8 | TET2 | 6.16 | 0.04 | 0.27 | 1.55E-09 | 7.35E-09 |
| NSUN7 | RBMX | 6.15 | 0.04 | 0.27 | 1.64E-09 | 7.78E-09 |
| CMTR2 | TRMT13 | 6.15 | 0.04 | 0.27 | 1.66E-09 | 7.87E-09 |
| DNMT1 | METTL3 | 6.14 | 0.04 | 0.27 | 1.72E-09 | 8.11E-09 |
| CTU1 | DNMT3B | 6.14 | 0.04 | 0.27 | 1.74E-09 | 8.22E-09 |
| TRMT6 | KIAA1429 | 6.14 | 0.04 | 0.27 | 1.76E-09 | 8.30E-09 |
| NSUN5 | HNRNPA2B1 | 6.14 | 0.04 | 0.27 | 1.78E-09 | 8.38E-09 |
| ADARB2 | ZC3H13 | 6.13 | 0.04 | 0.27 | 1.90E-09 | 8.95E-09 |
| DNMT3B | FMR1 | 6.12 | 0.04 | 0.27 | 1.93E-09 | 9.11E-09 |
| PUS7L | CMTR1 | 6.12 | 0.04 | 0.27 | 1.97E-09 | 9.24E-09 |
| NOP2 | METTL5 | 6.12 | 0.04 | 0.27 | 2.02E-09 | 9.49E-09 |
| DKC1 | TRUB1 | 6.11 | 0.04 | 0.27 | 2.04E-09 | 9.60E-09 |
| RPUSD3 | METTL3 | 6.11 | 0.04 | 0.27 | 2.07E-09 | 9.70E-09 |
| CMTR1 | KIAA1429 | 6.11 | 0.04 | 0.27 | 2.11E-09 | 9.87E-09 |
| MRM3 | TRMT10C | 6.10 | 0.04 | 0.27 | 2.17E-09 | 1.02E-08 |
| METTL1 | IGF2BP1 | 6.10 | 0.04 | 0.27 | 2.23E-09 | 1.04E-08 |
| LRPPRC | YTHDF1 | 6.10 | 0.04 | 0.27 | 2.24E-09 | 1.05E-08 |
| TRMT10C | ZCCHC4 | 6.10 | 0.04 | 0.27 | 2.25E-09 | 1.05E-08 |
| CMTR1 | YTHDF1 | 6.10 | 0.04 | 0.27 | 2.28E-09 | 1.06E-08 |
| DNMT1 | ZC3H13 | 6.09 | 0.04 | 0.27 | 2.33E-09 | 1.09E-08 |
| TRMT61B | ALYREF | 6.08 | 0.04 | 0.27 | 2.48E-09 | 1.15E-08 |
| TRDMT1 | FMR1 | 6.08 | 0.04 | 0.27 | 2.56E-09 | 1.19E-08 |
| TRMT6 | DNMT1 | 6.07 | 0.04 | 0.27 | 2.59E-09 | 1.20E-08 |
| RPUSD4 | TRMT13 | 6.07 | 0.04 | 0.27 | 2.61E-09 | 1.21E-08 |
| MRM3 | FBL | 6.07 | 0.04 | 0.27 | 2.62E-09 | 1.21E-08 |
| CTU1 | MRM1 | 6.07 | 0.04 | 0.27 | 2.64E-09 | 1.22E-08 |
| KIAA1429 | METTL14 | 6.07 | 0.04 | 0.27 | 2.66E-09 | 1.23E-08 |
| RPUSD1 | TRMT112 | 6.06 | 0.04 | 0.27 | 2.74E-09 | 1.26E-08 |
| FMR1 | METTL3 | 6.06 | 0.04 | 0.27 | 2.81E-09 | 1.29E-08 |
| ADAT3 | NSUN5 | 6.06 | 0.04 | 0.27 | 2.86E-09 | 1.32E-08 |
| TRMT11 | ZCCHC4 | 6.05 | 0.04 | 0.27 | 2.88E-09 | 1.33E-08 |
| HENMT1 | TRMT6 | 6.05 | 0.04 | 0.27 | 2.94E-09 | 1.35E-08 |
| PUS1 | LRPPRC | 6.05 | 0.04 | 0.27 | 2.96E-09 | 1.36E-08 |
| HENMT1 | RBM15 | 6.05 | 0.04 | 0.27 | 2.97E-09 | 1.36E-08 |
| ELP1 | FTSJ3 | 6.04 | 0.04 | 0.27 | 3.06E-09 | 1.40E-08 |
| PUS10 | TRMT10B | 6.04 | 0.04 | 0.27 | 3.09E-09 | 1.41E-08 |
| RNMT | YTHDF3 | 6.04 | 0.04 | 0.27 | 3.20E-09 | 1.46E-08 |
| PUS7L | PRRC2A | 6.03 | 0.04 | 0.27 | 3.27E-09 | 1.49E-08 |
| CTU1 | DNMT3A | 6.03 | 0.04 | 0.27 | 3.27E-09 | 1.49E-08 |
| ALKBH8 | ADARB1 | 6.03 | 0.04 | 0.27 | 3.34E-09 | 1.52E-08 |
| ADAR | YTHDF1 | 6.03 | 0.04 | 0.27 | 3.35E-09 | 1.52E-08 |
| PUS3 | TRMT6 | 6.03 | 0.04 | 0.27 | 3.37E-09 | 1.53E-08 |
| RPUSD4 | RBMX | 6.02 | 0.04 | 0.27 | 3.44E-09 | 1.56E-08 |
| ADAT2 | RBM15 | 6.02 | 0.04 | 0.27 | 3.47E-09 | 1.58E-08 |
| ADAR | G3BP2 | 6.02 | 0.04 | 0.27 | 3.56E-09 | 1.61E-08 |
| FTSJ1 | IGF2BP3 | 6.02 | 0.04 | 0.27 | 3.61E-09 | 1.63E-08 |
| TRMT11 | HNRNPA2B1 | 6.01 | 0.04 | 0.27 | 3.69E-09 | 1.67E-08 |
| DNMT3B | RBMX | 6.01 | 0.04 | 0.27 | 3.72E-09 | 1.68E-08 |
| ELP1 | RBMX | 6.01 | 0.04 | 0.27 | 3.76E-09 | 1.70E-08 |
| BUD23 | WDR4 | 6.01 | 0.04 | 0.27 | 3.81E-09 | 1.72E-08 |
| RNMT | RBMX | 6.00 | 0.04 | 0.27 | 3.84E-09 | 1.73E-08 |
| HENMT1 | DNMT3B | 6.00 | 0.04 | 0.27 | 3.85E-09 | 1.73E-08 |
| PUS7L | NSUN6 | 6.00 | 0.04 | 0.27 | 3.93E-09 | 1.77E-08 |
| TRDMT1 | YTHDF3 | 6.00 | 0.04 | 0.27 | 3.95E-09 | 1.77E-08 |
| ELP1 | TGS1 | 5.99 | 0.04 | 0.27 | 4.08E-09 | 1.83E-08 |
| YTHDC1 | CBLL1 | 5.99 | 0.04 | 0.27 | 4.09E-09 | 1.83E-08 |
| METTL1 | ELAVL1 | 5.99 | 0.04 | 0.27 | 4.12E-09 | 1.85E-08 |
| RNMT | TRMT61B | 5.99 | 0.04 | 0.27 | 4.21E-09 | 1.88E-08 |
| PUS1 | RBM15 | 5.99 | 0.04 | 0.27 | 4.29E-09 | 1.92E-08 |
| PUS7 | RBMX | 5.99 | 0.04 | 0.27 | 4.30E-09 | 1.92E-08 |
| RBMX | YTHDF3 | 5.98 | 0.04 | 0.27 | 4.36E-09 | 1.95E-08 |
| TRMT6 | YBX1 | 5.98 | 0.04 | 0.27 | 4.39E-09 | 1.96E-08 |
| TRMT10B | HNRNPA2B1 | 5.98 | 0.04 | 0.27 | 4.45E-09 | 1.98E-08 |
| MRM2 | ELAVL1 | 5.98 | 0.04 | 0.27 | 4.47E-09 | 1.99E-08 |
| G3BP2 | YTHDC1 | 5.98 | 0.04 | 0.27 | 4.52E-09 | 2.01E-08 |
| TRMT6 | TET3 | 5.97 | 0.04 | 0.27 | 4.61E-09 | 2.04E-08 |
| NSUN3 | EIF3A | 5.97 | 0.04 | 0.27 | 4.66E-09 | 2.07E-08 |
| RPUSD4 | WDR4 | 5.97 | 0.04 | 0.27 | 4.70E-09 | 2.08E-08 |
| TRUB1 | WTAP | 5.97 | 0.04 | 0.27 | 4.79E-09 | 2.12E-08 |
| TRMT44 | METTL14 | 5.96 | 0.04 | 0.27 | 4.94E-09 | 2.18E-08 |
| TET2 | FTO | 5.96 | 0.04 | 0.27 | 4.99E-09 | 2.20E-08 |
| ELP1 | G3BP2 | 5.95 | 0.04 | 0.27 | 5.13E-09 | 2.26E-08 |
| YTHDF2 | YTHDF1 | 5.95 | 0.04 | 0.26 | 5.21E-09 | 2.30E-08 |
| ADARB1 | TRMT10B | 5.95 | 0.04 | 0.26 | 5.35E-09 | 2.36E-08 |
| ADAR | RBM15B | 5.94 | 0.04 | 0.26 | 5.44E-09 | 2.39E-08 |
| TRMT44 | TET2 | 5.94 | 0.04 | 0.26 | 5.49E-09 | 2.41E-08 |
| ZCCHC4 | KIAA1429 | 5.94 | 0.04 | 0.26 | 5.71E-09 | 2.51E-08 |
| NSUN3 | G3BP1 | 5.93 | 0.04 | 0.26 | 5.74E-09 | 2.51E-08 |
| NOP2 | RBM15B | 5.93 | 0.04 | 0.26 | 5.81E-09 | 2.55E-08 |
| PUS10 | TRDMT1 | 5.92 | 0.04 | 0.26 | 6.40E-09 | 2.80E-08 |
| YTHDF3 | ZCCHC4 | 5.90 | 0.04 | 0.26 | 6.78E-09 | 2.96E-08 |
| IGF2BP3 | HNRNPA2B1 | 5.90 | 0.04 | 0.26 | 6.78E-09 | 2.96E-08 |
| TRMT112 | MRM3 | 5.90 | 0.04 | 0.26 | 7.02E-09 | 3.06E-08 |
| ADAT2 | NOP2 | 5.90 | 0.04 | 0.26 | 7.02E-09 | 3.06E-08 |
| PUS1 | DNMT1 | 5.90 | 0.04 | 0.26 | 7.09E-09 | 3.09E-08 |
| DNMT1 | G3BP1 | 5.90 | 0.04 | 0.26 | 7.12E-09 | 3.09E-08 |
| PUS7L | ZCCHC4 | 5.90 | 0.04 | 0.26 | 7.12E-09 | 3.09E-08 |
| NSUN6 | FMR1 | 5.89 | 0.04 | 0.26 | 7.21E-09 | 3.13E-08 |
| MRM2 | CBLL1 | 5.89 | 0.04 | 0.26 | 7.28E-09 | 3.16E-08 |
| LRPPRC | HNRNPA1 | 5.89 | 0.04 | 0.26 | 7.44E-09 | 3.22E-08 |
| TET1 | G3BP1 | 5.88 | 0.04 | 0.26 | 7.67E-09 | 3.32E-08 |
| ELAVL1 | KIAA1429 | 5.88 | 0.04 | 0.26 | 7.84E-09 | 3.39E-08 |
| ALKBH1 | TRMT61B | 5.87 | 0.04 | 0.26 | 8.34E-09 | 3.60E-08 |
| TRMT10A | NSUN3 | 5.86 | 0.04 | 0.26 | 8.52E-09 | 3.68E-08 |
| ADAR | NSUN3 | 5.85 | 0.04 | 0.26 | 9.04E-09 | 3.89E-08 |
| PUS7L | TARBP1 | 5.85 | 0.04 | 0.26 | 9.14E-09 | 3.93E-08 |
| TRMT10A | METTL14 | 5.85 | 0.04 | 0.26 | 9.16E-09 | 3.94E-08 |
| TRMT6 | WTAP | 5.85 | 0.04 | 0.26 | 9.29E-09 | 3.99E-08 |
| TET1 | NSUN2 | 5.85 | 0.04 | 0.26 | 9.29E-09 | 3.99E-08 |
| RPUSD1 | ALYREF | 5.85 | 0.04 | 0.26 | 9.30E-09 | 3.99E-08 |
| RRP8 | NSUN4 | 5.84 | 0.04 | 0.26 | 9.63E-09 | 4.13E-08 |
| NSUN7 | NSUN6 | 5.84 | 0.04 | 0.26 | 9.66E-09 | 4.14E-08 |
| RPUSD3 | NSUN5 | 5.84 | 0.04 | 0.26 | 9.74E-09 | 4.16E-08 |
| PUS10 | CMTR2 | 5.83 | 0.04 | 0.26 | 1.01E-08 | 4.30E-08 |
| FTO | METTL16 | 5.83 | 0.04 | 0.26 | 1.02E-08 | 4.36E-08 |
| ADARB2 | TRMT10B | 5.83 | 0.04 | 0.26 | 1.02E-08 | 4.37E-08 |
| ALKBH5 | PRRC2A | 5.83 | 0.04 | 0.26 | 1.05E-08 | 4.46E-08 |
| TGS1 | TET3 | 5.82 | 0.04 | 0.26 | 1.10E-08 | 4.68E-08 |
| RNMT | IGF2BP3 | 5.82 | 0.04 | 0.26 | 1.10E-08 | 4.70E-08 |
| HENMT1 | FTSJ1 | 5.81 | 0.04 | 0.26 | 1.15E-08 | 4.89E-08 |
| ELP1 | TRMT13 | 5.81 | 0.04 | 0.26 | 1.15E-08 | 4.89E-08 |
| RPUSD2 | METTL1 | 5.81 | 0.04 | 0.26 | 1.15E-08 | 4.89E-08 |
| ALKBH8 | TET1 | 5.81 | 0.04 | 0.26 | 1.18E-08 | 5.00E-08 |
| RNMT | NSUN3 | 5.81 | 0.04 | 0.26 | 1.19E-08 | 5.02E-08 |
| MRM2 | NSUN2 | 5.80 | 0.04 | 0.26 | 1.20E-08 | 5.07E-08 |
| G3BP2 | ZCCHC4 | 5.80 | 0.04 | 0.26 | 1.22E-08 | 5.14E-08 |
| FMR1 | METTL14 | 5.80 | 0.04 | 0.26 | 1.23E-08 | 5.18E-08 |
| TRMT61B | PRRC2A | 5.80 | 0.04 | 0.26 | 1.25E-08 | 5.28E-08 |
| WDR4 | CMTR1 | 5.79 | 0.04 | 0.26 | 1.28E-08 | 5.38E-08 |
| TARBP1 | NSUN2 | 5.79 | 0.04 | 0.26 | 1.28E-08 | 5.38E-08 |
| TET3 | G3BP1 | 5.79 | 0.04 | 0.26 | 1.30E-08 | 5.47E-08 |
| ALYREF | YTHDF1 | 5.79 | 0.04 | 0.26 | 1.32E-08 | 5.56E-08 |
| HNRNPA2B1 | KIAA1429 | 5.78 | 0.04 | 0.26 | 1.38E-08 | 5.80E-08 |
| DNMT3B | RBM15B | 5.78 | 0.04 | 0.26 | 1.39E-08 | 5.83E-08 |
| TRMT6 | IGF2BP2 | 5.78 | 0.04 | 0.26 | 1.39E-08 | 5.84E-08 |
| NSUN7 | HNRNPA2B1 | 5.77 | 0.04 | 0.26 | 1.41E-08 | 5.91E-08 |
| BUD23 | ALYREF | 5.77 | 0.04 | 0.26 | 1.42E-08 | 5.96E-08 |
| TRMT6 | TET1 | 5.77 | 0.04 | 0.26 | 1.43E-08 | 6.00E-08 |
| TRUB1 | TRDMT1 | 5.77 | 0.04 | 0.26 | 1.45E-08 | 6.07E-08 |
| ALYREF | WTAP | 5.76 | 0.04 | 0.26 | 1.53E-08 | 6.37E-08 |
| TRMT61A | ELAVL1 | 5.76 | 0.04 | 0.26 | 1.54E-08 | 6.40E-08 |
| BUD23 | FTSJ1 | 5.76 | 0.04 | 0.26 | 1.56E-08 | 6.47E-08 |
| ALKBH8 | NSUN3 | 5.75 | 0.04 | 0.26 | 1.57E-08 | 6.52E-08 |
| SNORD48 | PRRC2A | 5.75 | 0.04 | 0.26 | 1.58E-08 | 6.58E-08 |
| PUS3 | HNRNPC | 5.75 | 0.04 | 0.26 | 1.60E-08 | 6.64E-08 |
| RRP8 | TRMT10B | 5.75 | 0.04 | 0.26 | 1.62E-08 | 6.72E-08 |
| NSUN6 | YTHDC2 | 5.75 | 0.04 | 0.26 | 1.65E-08 | 6.83E-08 |
| MRM3 | METTL5 | 5.74 | 0.04 | 0.26 | 1.68E-08 | 6.94E-08 |
| RPUSD2 | PUS1 | 5.74 | 0.04 | 0.26 | 1.69E-08 | 6.99E-08 |
| FTSJ1 | FBL | 5.74 | 0.04 | 0.26 | 1.70E-08 | 7.04E-08 |
| NSUN4 | ZCCHC4 | 5.73 | 0.04 | 0.26 | 1.78E-08 | 7.35E-08 |
| PUS3 | LRPPRC | 5.72 | 0.04 | 0.26 | 1.87E-08 | 7.73E-08 |
| NKAP | METTL3 | 5.72 | 0.04 | 0.26 | 1.88E-08 | 7.73E-08 |
| WDR4 | IGF2BP3 | 5.72 | 0.04 | 0.26 | 1.88E-08 | 7.75E-08 |
| TRMT10B | METTL14 | 5.72 | 0.04 | 0.26 | 1.90E-08 | 7.83E-08 |
| METTL1 | IGF2BP3 | 5.72 | 0.04 | 0.26 | 1.92E-08 | 7.89E-08 |
| PUS3 | TRMT112 | 5.72 | 0.04 | 0.26 | 1.94E-08 | 7.98E-08 |
| G3BP1 | FMR1 | 5.72 | 0.04 | 0.26 | 1.95E-08 | 7.99E-08 |
| TRMT13 | DNMT3B | 5.71 | 0.04 | 0.26 | 1.97E-08 | 8.10E-08 |
| RPUSD4 | ALKBH1 | 5.71 | 0.04 | 0.25 | 1.99E-08 | 8.17E-08 |
| WDR4 | LRPPRC | 5.71 | 0.04 | 0.25 | 2.00E-08 | 8.17E-08 |
| RBM15B | METTL16 | 5.71 | 0.04 | 0.25 | 2.04E-08 | 8.32E-08 |
| TRDMT1 | KIAA1429 | 5.71 | 0.04 | 0.25 | 2.05E-08 | 8.39E-08 |
| TRMT13 | TET2 | 5.70 | 0.04 | 0.25 | 2.07E-08 | 8.44E-08 |
| TGS1 | NSUN2 | 5.70 | 0.04 | 0.25 | 2.15E-08 | 8.75E-08 |
| TRMT10A | TRMT61B | 5.69 | 0.04 | 0.25 | 2.22E-08 | 9.05E-08 |
| G3BP1 | HNRNPC | 5.69 | 0.04 | 0.25 | 2.27E-08 | 9.21E-08 |
| MRM2 | YTHDF1 | 5.69 | 0.04 | 0.25 | 2.27E-08 | 9.22E-08 |
| TET3 | METTL3 | 5.69 | 0.04 | 0.25 | 2.29E-08 | 9.27E-08 |
| TRMT10B | DNMT1 | 5.67 | 0.04 | 0.25 | 2.47E-08 | 9.99E-08 |
| METTL1 | MRM3 | 5.67 | 0.04 | 0.25 | 2.49E-08 | 1.01E-07 |
| TGS1 | IGF2BP3 | 5.67 | 0.04 | 0.25 | 2.53E-08 | 1.02E-07 |
| DKC1 | TET3 | 5.66 | 0.04 | 0.25 | 2.63E-08 | 1.06E-07 |
| DKC1 | BUD23 | 5.66 | 0.04 | 0.25 | 2.67E-08 | 1.08E-07 |
| MRM2 | IGF2BP3 | 5.66 | 0.04 | 0.25 | 2.68E-08 | 1.08E-07 |
| DNMT1 | NSUN6 | 5.65 | 0.04 | 0.25 | 2.77E-08 | 1.11E-07 |
| NSUN5 | NOP2 | 5.65 | 0.04 | 0.25 | 2.81E-08 | 1.13E-07 |
| ADARB1 | TET2 | 5.64 | 0.04 | 0.25 | 2.92E-08 | 1.17E-07 |
| FTSJ3 | TET1 | 5.64 | 0.04 | 0.25 | 2.98E-08 | 1.19E-07 |
| FTSJ3 | METTL14 | 5.64 | 0.04 | 0.25 | 2.98E-08 | 1.20E-07 |
| FTO | YTHDC1 | 5.63 | 0.04 | 0.25 | 3.03E-08 | 1.21E-07 |
| METTL5 | ZCCHC4 | 5.63 | 0.04 | 0.25 | 3.15E-08 | 1.26E-07 |
| ADAT3 | TARBP1 | 5.63 | 0.04 | 0.25 | 3.18E-08 | 1.27E-07 |
| TET1 | CBLL1 | 5.61 | 0.04 | 0.25 | 3.46E-08 | 1.38E-07 |
| RBMX | YTHDF2 | 5.60 | 0.04 | 0.25 | 3.58E-08 | 1.43E-07 |
| TRMT13 | NSUN3 | 5.60 | 0.04 | 0.25 | 3.58E-08 | 1.43E-07 |
| FTSJ1 | IGF2BP1 | 5.60 | 0.04 | 0.25 | 3.61E-08 | 1.44E-07 |
| DNMT1 | LRPPRC | 5.60 | 0.04 | 0.25 | 3.71E-08 | 1.48E-07 |
| RPUSD4 | TRMT61B | 5.59 | 0.04 | 0.25 | 3.78E-08 | 1.50E-07 |
| PUS7 | IGF2BP3 | 5.59 | 0.04 | 0.25 | 3.80E-08 | 1.51E-07 |
| TRMT112 | TRMT61A | 5.59 | 0.04 | 0.25 | 3.84E-08 | 1.52E-07 |
| CMTR1 | NSUN2 | 5.59 | 0.04 | 0.25 | 3.84E-08 | 1.52E-07 |
| TRMT61A | HNRNPA2B1 | 5.59 | 0.04 | 0.25 | 3.86E-08 | 1.53E-07 |
| PRRC2A | FMR1 | 5.59 | 0.04 | 0.25 | 3.88E-08 | 1.54E-07 |
| RNMT | IGF2BP2 | 5.59 | 0.04 | 0.25 | 3.93E-08 | 1.56E-07 |
| TET1 | IGF2BP1 | 5.59 | 0.04 | 0.25 | 3.95E-08 | 1.56E-07 |
| TRMT13 | TET1 | 5.58 | 0.04 | 0.25 | 4.06E-08 | 1.61E-07 |
| TRMT13 | NKAP | 5.58 | 0.04 | 0.25 | 4.11E-08 | 1.62E-07 |
| CMTR2 | TRDMT1 | 5.58 | 0.04 | 0.25 | 4.16E-08 | 1.64E-07 |
| ALKBH1 | TRMT61A | 5.57 | 0.04 | 0.25 | 4.34E-08 | 1.71E-07 |
| ALKBH8 | ELP1 | 5.56 | 0.04 | 0.25 | 4.43E-08 | 1.74E-07 |
| TET2 | PRRC2A | 5.56 | 0.04 | 0.25 | 4.48E-08 | 1.76E-07 |
| LRPPRC | YTHDC1 | 5.56 | 0.04 | 0.25 | 4.56E-08 | 1.79E-07 |
| HNRNPC | ZCCHC4 | 5.56 | 0.04 | 0.25 | 4.60E-08 | 1.81E-07 |
| ELP1 | PUS7 | 5.55 | 0.04 | 0.25 | 4.72E-08 | 1.85E-07 |
| PUS7L | NSUN4 | 5.55 | 0.04 | 0.25 | 4.74E-08 | 1.86E-07 |
| TET1 | HNRNPA1 | 5.55 | 0.04 | 0.25 | 4.88E-08 | 1.91E-07 |
| TRMT11 | DNMT1 | 5.55 | 0.04 | 0.25 | 4.91E-08 | 1.92E-07 |
| RPUSD2 | MRM1 | 5.54 | 0.04 | 0.25 | 5.02E-08 | 1.96E-07 |
| NSUN7 | FMR1 | 5.54 | 0.04 | 0.25 | 5.02E-08 | 1.96E-07 |
| METTL1 | MRM1 | 5.54 | 0.04 | 0.25 | 5.09E-08 | 1.99E-07 |
| WDR4 | TRMT61B | 5.54 | 0.04 | 0.25 | 5.17E-08 | 2.02E-07 |
| PUS1 | RBM15B | 5.54 | 0.04 | 0.25 | 5.18E-08 | 2.02E-07 |
| CMTR1 | NSUN7 | 5.53 | 0.04 | 0.25 | 5.21E-08 | 2.03E-07 |
| TRUB1 | CMTR2 | 5.53 | 0.04 | 0.25 | 5.32E-08 | 2.07E-07 |
| RPUSD1 | MRM3 | 5.53 | 0.04 | 0.25 | 5.36E-08 | 2.08E-07 |
| PUS7L | YTHDF2 | 5.52 | 0.04 | 0.25 | 5.62E-08 | 2.18E-07 |
| ADAR | TRMT61B | 5.52 | 0.04 | 0.25 | 5.74E-08 | 2.23E-07 |
| ADAT2 | TRMT61A | 5.51 | 0.04 | 0.25 | 5.78E-08 | 2.24E-07 |
| TRUB1 | PUS3 | 5.51 | 0.04 | 0.25 | 5.82E-08 | 2.25E-07 |
| DKC1 | EIF3A | 5.51 | 0.04 | 0.25 | 5.85E-08 | 2.26E-07 |
| ALKBH8 | PUS7 | 5.51 | 0.04 | 0.25 | 5.94E-08 | 2.30E-07 |
| PUS7 | RNMT | 5.51 | 0.04 | 0.25 | 6.00E-08 | 2.32E-07 |
| NSUN2 | IGF2BP1 | 5.51 | 0.04 | 0.25 | 6.00E-08 | 2.32E-07 |
| TRMT61A | PRRC2A | 5.51 | 0.04 | 0.25 | 6.05E-08 | 2.34E-07 |
| ELAVL1 | HNRNPC | 5.49 | 0.04 | 0.25 | 6.50E-08 | 2.51E-07 |
| TRMT61A | DNMT3A | 5.49 | 0.04 | 0.25 | 6.65E-08 | 2.56E-07 |
| DKC1 | ALKBH1 | 5.49 | 0.04 | 0.25 | 6.66E-08 | 2.56E-07 |
| ALYREF | IGF2BP1 | 5.49 | 0.04 | 0.25 | 6.73E-08 | 2.59E-07 |
| FTSJ1 | DNMT3B | 5.48 | 0.04 | 0.25 | 6.80E-08 | 2.61E-07 |
| RPUSD4 | NSUN4 | 5.48 | 0.04 | 0.25 | 7.11E-08 | 2.73E-07 |
| ALYREF | KIAA1429 | 5.47 | 0.04 | 0.24 | 7.24E-08 | 2.78E-07 |
| CMTR2 | YTHDF3 | 5.47 | 0.04 | 0.24 | 7.26E-08 | 2.78E-07 |
| TRMT11 | DNMT3B | 5.47 | 0.04 | 0.24 | 7.36E-08 | 2.82E-07 |
| ALKBH3 | RRP8 | 5.46 | 0.04 | 0.24 | 7.53E-08 | 2.88E-07 |
| PRRC2A | METTL16 | 5.46 | 0.04 | 0.24 | 7.56E-08 | 2.89E-07 |
| TGS1 | ZCCHC4 | 5.46 | 0.04 | 0.24 | 7.68E-08 | 2.93E-07 |
| HENMT1 | NSUN3 | 5.46 | 0.04 | 0.24 | 7.81E-08 | 2.98E-07 |
| FTO | YTHDC2 | 5.45 | 0.04 | 0.24 | 8.10E-08 | 3.09E-07 |
| TRMT6 | FMR1 | 5.45 | 0.04 | 0.24 | 8.19E-08 | 3.12E-07 |
| ADAT2 | WDR4 | 5.44 | 0.04 | 0.24 | 8.41E-08 | 3.20E-07 |
| FTSJ3 | IGF2BP2 | 5.44 | 0.04 | 0.24 | 8.47E-08 | 3.22E-07 |
| YTHDC1 | ZCCHC4 | 5.44 | 0.04 | 0.24 | 8.57E-08 | 3.26E-07 |
| ADAR | YTHDF2 | 5.43 | 0.04 | 0.24 | 8.97E-08 | 3.41E-07 |
| TET1 | ELAVL1 | 5.43 | 0.04 | 0.24 | 9.27E-08 | 3.52E-07 |
| HENMT1 | TRMT13 | 5.42 | 0.04 | 0.24 | 9.36E-08 | 3.56E-07 |
| TARBP1 | NSUN4 | 5.42 | 0.04 | 0.24 | 9.62E-08 | 3.65E-07 |
| ELAVL1 | FMR1 | 5.41 | 0.04 | 0.24 | 9.93E-08 | 3.76E-07 |
| RPUSD2 | FTSJ1 | 5.41 | 0.04 | 0.24 | 1.02E-07 | 3.88E-07 |
| ADAT3 | DNMT1 | 5.41 | 0.04 | 0.24 | 1.03E-07 | 3.90E-07 |
| ADAT2 | MRM1 | 5.40 | 0.04 | 0.24 | 1.07E-07 | 4.06E-07 |
| LRPPRC | ZC3H13 | 5.39 | 0.04 | 0.24 | 1.09E-07 | 4.13E-07 |
| ADAT3 | MRM1 | 5.39 | 0.04 | 0.24 | 1.13E-07 | 4.26E-07 |
| ZC3H13 | RBM15 | 5.38 | 0.04 | 0.24 | 1.20E-07 | 4.52E-07 |
| ADAR | YTHDC2 | 5.38 | 0.04 | 0.24 | 1.21E-07 | 4.54E-07 |
| TGS1 | YTHDC1 | 5.37 | 0.04 | 0.24 | 1.21E-07 | 4.55E-07 |
| MRM3 | TRMT61A | 5.37 | 0.04 | 0.24 | 1.23E-07 | 4.63E-07 |
| RPUSD3 | BUD23 | 5.37 | 0.04 | 0.24 | 1.26E-07 | 4.71E-07 |
| BUD23 | MRM2 | 5.37 | 0.04 | 0.24 | 1.26E-07 | 4.73E-07 |
| ELP1 | ADARB1 | 5.37 | 0.04 | 0.24 | 1.27E-07 | 4.75E-07 |
| PUS7 | MRM2 | 5.36 | 0.04 | 0.24 | 1.31E-07 | 4.92E-07 |
| DKC1 | IGF2BP1 | 5.35 | 0.04 | 0.24 | 1.35E-07 | 5.05E-07 |
| FBL | RBMX | 5.35 | 0.04 | 0.24 | 1.36E-07 | 5.08E-07 |
| ELP1 | TRMT61B | 5.35 | 0.04 | 0.24 | 1.38E-07 | 5.16E-07 |
| TRUB1 | YBX1 | 5.35 | 0.04 | 0.24 | 1.40E-07 | 5.21E-07 |
| TRMT61A | DNMT1 | 5.34 | 0.04 | 0.24 | 1.43E-07 | 5.34E-07 |
| RRP8 | TRDMT1 | 5.34 | 0.04 | 0.24 | 1.44E-07 | 5.36E-07 |
| RPUSD4 | TRUB1 | 5.34 | 0.04 | 0.24 | 1.45E-07 | 5.38E-07 |
| TET3 | IGF2BP1 | 5.34 | 0.04 | 0.24 | 1.46E-07 | 5.44E-07 |
| DKC1 | MRM1 | 5.34 | 0.04 | 0.24 | 1.47E-07 | 5.44E-07 |
| CMTR1 | IGF2BP2 | 5.33 | 0.04 | 0.24 | 1.53E-07 | 5.68E-07 |
| RRP8 | RBMX | 5.33 | 0.04 | 0.24 | 1.54E-07 | 5.72E-07 |
| ADARB1 | CMTR1 | 5.33 | 0.04 | 0.24 | 1.56E-07 | 5.79E-07 |
| PUS10 | NSUN4 | 5.32 | 0.04 | 0.24 | 1.59E-07 | 5.91E-07 |
| TRMT44 | RBM15 | 5.32 | 0.04 | 0.24 | 1.60E-07 | 5.91E-07 |
| RNMT | YTHDF1 | 5.32 | 0.04 | 0.24 | 1.60E-07 | 5.91E-07 |
| PUS7L | TET2 | 5.32 | 0.04 | 0.24 | 1.60E-07 | 5.92E-07 |
| PUS7L | HNRNPA1 | 5.31 | 0.04 | 0.24 | 1.67E-07 | 6.16E-07 |
| NSUN4 | RBM15 | 5.31 | 0.04 | 0.24 | 1.67E-07 | 6.16E-07 |
| YBX1 | IGF2BP1 | 5.31 | 0.04 | 0.24 | 1.72E-07 | 6.34E-07 |
| IGF2BP3 | HNRNPC | 5.31 | 0.04 | 0.24 | 1.72E-07 | 6.35E-07 |
| TGS1 | DNMT3B | 5.31 | 0.04 | 0.24 | 1.74E-07 | 6.40E-07 |
| DKC1 | RNMT | 5.30 | 0.04 | 0.24 | 1.78E-07 | 6.55E-07 |
| ALKBH1 | ZCCHC4 | 5.29 | 0.04 | 0.24 | 1.86E-07 | 6.84E-07 |
| MRM3 | TRMT10A | 5.29 | 0.04 | 0.24 | 1.87E-07 | 6.88E-07 |
| NSUN7 | RBM15 | 5.29 | 0.04 | 0.24 | 1.87E-07 | 6.88E-07 |
| PUS7 | MRM1 | 5.29 | 0.04 | 0.24 | 1.90E-07 | 6.98E-07 |
| ADAR | HNRNPA1 | 5.28 | 0.04 | 0.24 | 1.94E-07 | 7.12E-07 |
| NSUN2 | RBM15B | 5.28 | 0.04 | 0.24 | 1.95E-07 | 7.15E-07 |
| SNORD48 | YTHDC1 | 5.28 | 0.04 | 0.24 | 1.96E-07 | 7.17E-07 |
| ADARB2 | YTHDC1 | 5.28 | 0.04 | 0.24 | 2.01E-07 | 7.34E-07 |
| LRPPRC | ELAVL1 | 5.27 | 0.04 | 0.24 | 2.10E-07 | 7.67E-07 |
| TET1 | NOP2 | 5.27 | 0.04 | 0.24 | 2.10E-07 | 7.67E-07 |
| RPUSD4 | TRMT10C | 5.26 | 0.04 | 0.24 | 2.22E-07 | 8.12E-07 |
| SNORD48 | DNMT3B | 5.26 | 0.04 | 0.24 | 2.25E-07 | 8.20E-07 |
| PUS7L | METTL14 | 5.25 | 0.04 | 0.24 | 2.26E-07 | 8.23E-07 |
| TRMT44 | NSUN6 | 5.25 | 0.04 | 0.24 | 2.32E-07 | 8.46E-07 |
| CMTR1 | CBLL1 | 5.25 | 0.04 | 0.24 | 2.34E-07 | 8.51E-07 |
| TRUB1 | ALKBH1 | 5.24 | 0.04 | 0.24 | 2.37E-07 | 8.64E-07 |
| YTHDF1 | KIAA1429 | 5.24 | 0.04 | 0.24 | 2.39E-07 | 8.70E-07 |
| LRPPRC | FMR1 | 5.24 | 0.04 | 0.24 | 2.41E-07 | 8.77E-07 |
| DNMT3A | HNRNPA1 | 5.24 | 0.04 | 0.24 | 2.43E-07 | 8.82E-07 |
| ADAR | TRMT6 | 5.23 | 0.04 | 0.23 | 2.52E-07 | 9.12E-07 |
| RBMX | PRRC2A | 5.23 | 0.04 | 0.23 | 2.57E-07 | 9.32E-07 |
| NSUN3 | KIAA1429 | 5.23 | 0.04 | 0.23 | 2.57E-07 | 9.32E-07 |
| TRMT44 | YTHDF1 | 5.23 | 0.04 | 0.23 | 2.62E-07 | 9.48E-07 |
| TRUB1 | METTL5 | 5.22 | 0.04 | 0.23 | 2.69E-07 | 9.73E-07 |
| PUS3 | CBLL1 | 5.21 | 0.04 | 0.23 | 2.79E-07 | 1.01E-06 |
| TGS1 | ALYREF | 5.21 | 0.04 | 0.23 | 2.86E-07 | 1.03E-06 |
| TET2 | YTHDF2 | 5.20 | 0.04 | 0.23 | 2.99E-07 | 1.08E-06 |
| TRMT44 | DNMT3A | 5.20 | 0.04 | 0.23 | 3.05E-07 | 1.10E-06 |
| RPUSD3 | METTL1 | 5.20 | 0.04 | 0.23 | 3.06E-07 | 1.10E-06 |
| TRMT10C | G3BP1 | 5.19 | 0.04 | 0.23 | 3.07E-07 | 1.10E-06 |
| RPUSD1 | WDR4 | 5.19 | 0.04 | 0.23 | 3.12E-07 | 1.12E-06 |
| FTSJ3 | ZC3H13 | 5.19 | 0.04 | 0.23 | 3.16E-07 | 1.13E-06 |
| TET1 | METTL14 | 5.19 | 0.04 | 0.23 | 3.21E-07 | 1.15E-06 |
| DKC1 | YTHDF3 | 5.18 | 0.04 | 0.23 | 3.29E-07 | 1.18E-06 |
| YBX1 | IGF2BP2 | 5.17 | 0.04 | 0.23 | 3.40E-07 | 1.22E-06 |
| TRMT11 | NKAP | 5.17 | 0.04 | 0.23 | 3.41E-07 | 1.22E-06 |
| PUS7 | TRMT13 | 5.17 | 0.04 | 0.23 | 3.42E-07 | 1.22E-06 |
| DNMT1 | NKAP | 5.17 | 0.04 | 0.23 | 3.43E-07 | 1.23E-06 |
| IGF2BP3 | METTL5 | 5.17 | 0.04 | 0.23 | 3.45E-07 | 1.23E-06 |
| TRDMT1 | YTHDC2 | 5.17 | 0.04 | 0.23 | 3.45E-07 | 1.23E-06 |
| ADAT2 | YTHDC1 | 5.17 | 0.04 | 0.23 | 3.55E-07 | 1.26E-06 |
| HNRNPA1 | RBM15B | 5.17 | 0.04 | 0.23 | 3.55E-07 | 1.27E-06 |
| ADARB2 | YTHDC2 | 5.16 | 0.04 | 0.23 | 3.57E-07 | 1.27E-06 |
| CMTR1 | ALKBH1 | 5.16 | 0.04 | 0.23 | 3.58E-07 | 1.27E-06 |
| TRMT44 | METTL3 | 5.16 | 0.04 | 0.23 | 3.58E-07 | 1.27E-06 |
| PUS7L | FTSJ3 | 5.16 | 0.04 | 0.23 | 3.59E-07 | 1.28E-06 |
| HNRNPA2B1 | YTHDC2 | 5.16 | 0.04 | 0.23 | 3.60E-07 | 1.28E-06 |
| NOP2 | RBM15 | 5.15 | 0.04 | 0.23 | 3.77E-07 | 1.34E-06 |
| CTU2 | ELAVL1 | 5.15 | 0.04 | 0.23 | 3.89E-07 | 1.38E-06 |
| NSUN4 | YTHDC1 | 5.15 | 0.04 | 0.23 | 3.91E-07 | 1.38E-06 |
| ADARB1 | METTL14 | 5.15 | 0.04 | 0.23 | 3.92E-07 | 1.39E-06 |
| CTU1 | DNMT1 | 5.14 | 0.04 | 0.23 | 4.05E-07 | 1.43E-06 |
| TRMT61B | WTAP | 5.14 | 0.04 | 0.23 | 4.06E-07 | 1.44E-06 |
| LRPPRC | METTL16 | 5.14 | 0.04 | 0.23 | 4.09E-07 | 1.44E-06 |
| ALKBH8 | HNRNPA2B1 | 5.14 | 0.04 | 0.23 | 4.09E-07 | 1.44E-06 |
| FTSJ3 | NSUN5 | 5.14 | 0.04 | 0.23 | 4.11E-07 | 1.45E-06 |
| PUS7L | TRMT11 | 5.14 | 0.04 | 0.23 | 4.12E-07 | 1.45E-06 |
| SNORD48 | TET1 | 5.14 | 0.04 | 0.23 | 4.12E-07 | 1.45E-06 |
| HENMT1 | NSUN2 | 5.12 | 0.04 | 0.23 | 4.45E-07 | 1.56E-06 |
| PRRC2A | G3BP1 | 5.12 | 0.04 | 0.23 | 4.46E-07 | 1.57E-06 |
| LRPPRC | IGF2BP1 | 5.12 | 0.04 | 0.23 | 4.50E-07 | 1.58E-06 |
| TRMT44 | NSUN4 | 5.12 | 0.04 | 0.23 | 4.50E-07 | 1.58E-06 |
| FTSJ3 | IGF2BP3 | 5.11 | 0.04 | 0.23 | 4.73E-07 | 1.66E-06 |
| YTHDF3 | WTAP | 5.10 | 0.04 | 0.23 | 4.87E-07 | 1.71E-06 |
| CMTR1 | TRMT61B | 5.10 | 0.04 | 0.23 | 4.88E-07 | 1.71E-06 |
| DNMT3B | METTL5 | 5.10 | 0.04 | 0.23 | 4.93E-07 | 1.73E-06 |
| TRMT6 | IGF2BP1 | 5.10 | 0.04 | 0.23 | 4.94E-07 | 1.73E-06 |
| PUS1 | TET3 | 5.10 | 0.04 | 0.23 | 4.99E-07 | 1.74E-06 |
| SNORD48 | TET3 | 5.09 | 0.04 | 0.23 | 5.17E-07 | 1.81E-06 |
| TGS1 | ALKBH1 | 5.09 | 0.04 | 0.23 | 5.25E-07 | 1.83E-06 |
| DKC1 | HENMT1 | 5.09 | 0.04 | 0.23 | 5.26E-07 | 1.83E-06 |
| CMTR1 | NKAP | 5.08 | 0.04 | 0.23 | 5.36E-07 | 1.86E-06 |
| LRPPRC | HNRNPA2B1 | 5.08 | 0.04 | 0.23 | 5.42E-07 | 1.88E-06 |
| CMTR2 | NSUN3 | 5.08 | 0.04 | 0.23 | 5.46E-07 | 1.90E-06 |
| SNORD48 | TRMT13 | 5.08 | 0.04 | 0.23 | 5.54E-07 | 1.92E-06 |
| RRP8 | METTL3 | 5.07 | 0.04 | 0.23 | 5.61E-07 | 1.95E-06 |
| ADAT2 | TRMT44 | 5.07 | 0.04 | 0.23 | 5.72E-07 | 1.99E-06 |
| WDR4 | SNORD48 | 5.07 | 0.04 | 0.23 | 5.73E-07 | 1.99E-06 |
| PUS7L | TRDMT1 | 5.07 | 0.04 | 0.23 | 5.74E-07 | 1.99E-06 |
| DNMT3A | IGF2BP2 | 5.07 | 0.04 | 0.23 | 5.85E-07 | 2.02E-06 |
| PUS7L | NSUN7 | 5.07 | 0.04 | 0.23 | 5.87E-07 | 2.03E-06 |
| RNMT | TRMT13 | 5.06 | 0.04 | 0.23 | 5.90E-07 | 2.04E-06 |
| MRM1 | HNRNPA2B1 | 5.06 | 0.04 | 0.23 | 5.98E-07 | 2.07E-06 |
| RPUSD4 | PRRC2A | 5.06 | 0.04 | 0.23 | 5.99E-07 | 2.07E-06 |
| ELP1 | DNMT3B | 5.06 | 0.04 | 0.23 | 6.02E-07 | 2.07E-06 |
| FBL | IGF2BP1 | 5.06 | 0.04 | 0.23 | 6.06E-07 | 2.09E-06 |
| TRMT11 | RBM15 | 5.04 | 0.04 | 0.23 | 6.58E-07 | 2.26E-06 |
| PRRC2A | METTL14 | 5.04 | 0.04 | 0.23 | 6.58E-07 | 2.26E-06 |
| MRM1 | NSUN2 | 5.04 | 0.04 | 0.23 | 6.65E-07 | 2.28E-06 |
| TARBP1 | TET1 | 5.04 | 0.04 | 0.23 | 6.71E-07 | 2.30E-06 |
| TRMT13 | METTL14 | 5.04 | 0.04 | 0.23 | 6.72E-07 | 2.31E-06 |
| TGS1 | FTSJ3 | 5.04 | 0.04 | 0.23 | 6.77E-07 | 2.32E-06 |
| PUS10 | NSUN7 | 5.03 | 0.04 | 0.23 | 6.83E-07 | 2.34E-06 |
| PUS7L | NOP2 | 5.03 | 0.04 | 0.23 | 6.89E-07 | 2.36E-06 |
| BUD23 | MRM3 | 5.03 | 0.04 | 0.23 | 7.00E-07 | 2.40E-06 |
| WDR4 | RBM15B | 5.03 | 0.04 | 0.23 | 7.11E-07 | 2.43E-06 |
| PUS7 | TRMT61A | 5.02 | 0.04 | 0.23 | 7.24E-07 | 2.47E-06 |
| SNORD48 | TRMT10B | 5.02 | 0.04 | 0.23 | 7.24E-07 | 2.47E-06 |
| CMTR1 | YTHDC2 | 5.02 | 0.04 | 0.23 | 7.31E-07 | 2.49E-06 |
| PUS7L | WTAP | 5.01 | 0.04 | 0.23 | 7.54E-07 | 2.57E-06 |
| CTU2 | MRM2 | 5.01 | 0.04 | 0.23 | 7.55E-07 | 2.57E-06 |
| FMR1 | ZC3H13 | 5.01 | 0.04 | 0.23 | 7.77E-07 | 2.64E-06 |
| TRMT44 | PRRC2A | 5.00 | 0.04 | 0.22 | 8.17E-07 | 2.78E-06 |
| FTSJ1 | HNRNPC | 5.00 | 0.04 | 0.22 | 8.31E-07 | 2.82E-06 |
| PUS7 | TET3 | 4.99 | 0.04 | 0.22 | 8.37E-07 | 2.84E-06 |
| RPUSD2 | HNRNPC | 4.99 | 0.04 | 0.22 | 8.38E-07 | 2.84E-06 |
| PUS1 | FTSJ3 | 4.99 | 0.04 | 0.22 | 8.38E-07 | 2.84E-06 |
| ELP1 | ADAT2 | 4.99 | 0.04 | 0.22 | 8.40E-07 | 2.84E-06 |
| ALKBH5 | ELAVL1 | 4.99 | 0.04 | 0.22 | 8.41E-07 | 2.85E-06 |
| RPUSD4 | PUS7L | 4.99 | 0.04 | 0.22 | 8.46E-07 | 2.86E-06 |
| TRDMT1 | NSUN7 | 4.99 | 0.04 | 0.22 | 8.52E-07 | 2.88E-06 |
| TRDMT1 | YTHDC1 | 4.99 | 0.04 | 0.22 | 8.53E-07 | 2.88E-06 |
| ADAR | NOP2 | 4.99 | 0.04 | 0.22 | 8.54E-07 | 2.88E-06 |
| TGS1 | TRMT13 | 4.99 | 0.04 | 0.22 | 8.69E-07 | 2.93E-06 |
| BUD23 | NOP2 | 4.99 | 0.04 | 0.22 | 8.70E-07 | 2.93E-06 |
| ALKBH8 | CMTR2 | 4.98 | 0.04 | 0.22 | 8.82E-07 | 2.97E-06 |
| TRMT61B | EIF3A | 4.98 | 0.05 | 0.22 | 8.96E-07 | 3.02E-06 |
| TRMT10C | KIAA1429 | 4.98 | 0.05 | 0.22 | 8.99E-07 | 3.03E-06 |
| RPUSD4 | DNMT3A | 4.98 | 0.05 | 0.22 | 9.00E-07 | 3.03E-06 |
| YTHDC2 | METTL3 | 4.98 | 0.05 | 0.22 | 9.04E-07 | 3.04E-06 |
| PUS1 | YTHDF1 | 4.98 | 0.05 | 0.22 | 9.15E-07 | 3.07E-06 |
| HENMT1 | TET3 | 4.97 | 0.05 | 0.22 | 9.19E-07 | 3.08E-06 |
| NOP2 | YTHDF1 | 4.97 | 0.05 | 0.22 | 9.31E-07 | 3.12E-06 |
| RPUSD3 | METTL5 | 4.97 | 0.05 | 0.22 | 9.58E-07 | 3.21E-06 |
| RPUSD4 | ZCCHC4 | 4.96 | 0.05 | 0.22 | 1.01E-06 | 3.36E-06 |
| TRMT6 | G3BP1 | 4.95 | 0.05 | 0.22 | 1.02E-06 | 3.42E-06 |
| MRM1 | NSUN6 | 4.95 | 0.05 | 0.22 | 1.03E-06 | 3.44E-06 |
| ELAVL1 | NKAP | 4.95 | 0.05 | 0.22 | 1.03E-06 | 3.45E-06 |
| PUS7 | RBM15B | 4.95 | 0.05 | 0.22 | 1.04E-06 | 3.48E-06 |
| RPUSD1 | ELAVL1 | 4.94 | 0.05 | 0.22 | 1.07E-06 | 3.57E-06 |
| TGS1 | TRMT10A | 4.94 | 0.05 | 0.22 | 1.08E-06 | 3.58E-06 |
| RBMX | ELAVL1 | 4.94 | 0.05 | 0.22 | 1.08E-06 | 3.59E-06 |
| TET2 | NSUN7 | 4.94 | 0.05 | 0.22 | 1.09E-06 | 3.62E-06 |
| FTO | G3BP2 | 4.94 | 0.05 | 0.22 | 1.10E-06 | 3.64E-06 |
| TRUB1 | NSUN4 | 4.94 | 0.05 | 0.22 | 1.10E-06 | 3.67E-06 |
| TRMT11 | YTHDC2 | 4.94 | 0.05 | 0.22 | 1.11E-06 | 3.68E-06 |
| TET2 | DNMT1 | 4.94 | 0.05 | 0.22 | 1.11E-06 | 3.68E-06 |
| DNMT1 | HNRNPA1 | 4.93 | 0.05 | 0.22 | 1.12E-06 | 3.71E-06 |
| DNMT3B | CBLL1 | 4.93 | 0.05 | 0.22 | 1.13E-06 | 3.74E-06 |
| ADAT3 | METTL3 | 4.93 | 0.05 | 0.22 | 1.13E-06 | 3.75E-06 |
| TGS1 | CMTR1 | 4.93 | 0.05 | 0.22 | 1.14E-06 | 3.78E-06 |
| TET2 | KIAA1429 | 4.92 | 0.05 | 0.22 | 1.19E-06 | 3.93E-06 |
| TRMT6 | YTHDF3 | 4.92 | 0.05 | 0.22 | 1.20E-06 | 3.96E-06 |
| ELP1 | TRMT11 | 4.91 | 0.05 | 0.22 | 1.23E-06 | 4.06E-06 |
| PUS10 | YTHDF2 | 4.91 | 0.05 | 0.22 | 1.23E-06 | 4.06E-06 |
| TRMT11 | CMTR1 | 4.91 | 0.05 | 0.22 | 1.25E-06 | 4.10E-06 |
| ELP1 | YTHDF1 | 4.91 | 0.05 | 0.22 | 1.26E-06 | 4.14E-06 |
| FMR1 | YTHDF2 | 4.91 | 0.05 | 0.22 | 1.26E-06 | 4.15E-06 |
| TGS1 | ZC3H13 | 4.91 | 0.05 | 0.22 | 1.27E-06 | 4.18E-06 |
| TGS1 | METTL14 | 4.90 | 0.05 | 0.22 | 1.33E-06 | 4.35E-06 |
| TRMT13 | TRMT44 | 4.90 | 0.05 | 0.22 | 1.33E-06 | 4.35E-06 |
| MRM1 | SNORD48 | 4.90 | 0.05 | 0.22 | 1.34E-06 | 4.40E-06 |
| WDR4 | TRMT6 | 4.90 | 0.05 | 0.22 | 1.34E-06 | 4.40E-06 |
| NSUN2 | FMR1 | 4.89 | 0.05 | 0.22 | 1.39E-06 | 4.54E-06 |
| CMTR1 | TRMT61A | 4.89 | 0.05 | 0.22 | 1.40E-06 | 4.58E-06 |
| BUD23 | METTL3 | 4.89 | 0.05 | 0.22 | 1.42E-06 | 4.64E-06 |
| DNMT3B | YTHDC1 | 4.88 | 0.05 | 0.22 | 1.42E-06 | 4.65E-06 |
| TRMT44 | YTHDC2 | 4.88 | 0.05 | 0.22 | 1.43E-06 | 4.68E-06 |
| TARBP1 | RBMX | 4.88 | 0.05 | 0.22 | 1.44E-06 | 4.68E-06 |
| NSUN6 | NSUN2 | 4.88 | 0.05 | 0.22 | 1.44E-06 | 4.69E-06 |
| HENMT1 | IGF2BP1 | 4.88 | 0.05 | 0.22 | 1.46E-06 | 4.76E-06 |
| RPUSD3 | ALYREF | 4.87 | 0.05 | 0.22 | 1.51E-06 | 4.91E-06 |
| TARBP1 | FMR1 | 4.87 | 0.05 | 0.22 | 1.55E-06 | 5.05E-06 |
| TRMT13 | TRMT6 | 4.87 | 0.05 | 0.22 | 1.56E-06 | 5.06E-06 |
| RPUSD1 | MRM2 | 4.85 | 0.05 | 0.22 | 1.67E-06 | 5.41E-06 |
| TRMT11 | TARBP1 | 4.85 | 0.05 | 0.22 | 1.70E-06 | 5.50E-06 |
| RPUSD4 | TRMT11 | 4.85 | 0.05 | 0.22 | 1.70E-06 | 5.50E-06 |
| FMR1 | WTAP | 4.85 | 0.05 | 0.22 | 1.71E-06 | 5.52E-06 |
| CMTR1 | IGF2BP3 | 4.84 | 0.05 | 0.22 | 1.72E-06 | 5.57E-06 |
| TGS1 | FMR1 | 4.84 | 0.05 | 0.22 | 1.72E-06 | 5.57E-06 |
| NSUN2 | METTL3 | 4.84 | 0.05 | 0.22 | 1.81E-06 | 5.83E-06 |
| PUS1 | TRMT6 | 4.83 | 0.05 | 0.22 | 1.82E-06 | 5.87E-06 |
| RNMT | METTL16 | 4.83 | 0.05 | 0.22 | 1.84E-06 | 5.93E-06 |
| ELAVL1 | EIF3A | 4.83 | 0.05 | 0.22 | 1.88E-06 | 6.06E-06 |
| NSUN4 | NSUN7 | 4.82 | 0.05 | 0.22 | 1.91E-06 | 6.13E-06 |
| HENMT1 | TRMT61B | 4.82 | 0.05 | 0.22 | 1.91E-06 | 6.14E-06 |
| METTL1 | MRM2 | 4.82 | 0.05 | 0.22 | 1.92E-06 | 6.17E-06 |
| CTU1 | METTL1 | 4.82 | 0.05 | 0.22 | 1.94E-06 | 6.23E-06 |
| CTU1 | WDR4 | 4.82 | 0.05 | 0.22 | 1.94E-06 | 6.23E-06 |
| BUD23 | NSUN2 | 4.82 | 0.05 | 0.22 | 1.98E-06 | 6.36E-06 |
| FTSJ3 | TRMT61A | 4.81 | 0.05 | 0.22 | 2.01E-06 | 6.43E-06 |
| IGF2BP2 | HNRNPA2B1 | 4.81 | 0.05 | 0.22 | 2.06E-06 | 6.59E-06 |
| EIF3A | YTHDC2 | 4.80 | 0.05 | 0.22 | 2.10E-06 | 6.74E-06 |
| WDR4 | METTL1 | 4.80 | 0.05 | 0.22 | 2.14E-06 | 6.83E-06 |
| ADAT3 | NOP2 | 4.79 | 0.05 | 0.22 | 2.19E-06 | 7.01E-06 |
| TRMT61B | NSUN6 | 4.79 | 0.05 | 0.22 | 2.21E-06 | 7.06E-06 |
| NSUN2 | METTL5 | 4.79 | 0.05 | 0.22 | 2.24E-06 | 7.14E-06 |
| TRMT10A | CBLL1 | 4.79 | 0.05 | 0.22 | 2.25E-06 | 7.17E-06 |
| TET2 | HNRNPA2B1 | 4.79 | 0.05 | 0.22 | 2.25E-06 | 7.18E-06 |
| TARBP1 | NOP2 | 4.79 | 0.05 | 0.22 | 2.27E-06 | 7.25E-06 |
| PUS3 | YTHDF3 | 4.79 | 0.05 | 0.22 | 2.28E-06 | 7.27E-06 |
| MRM2 | RBM15 | 4.79 | 0.05 | 0.22 | 2.29E-06 | 7.29E-06 |
| ADAR | YTHDF3 | 4.78 | 0.05 | 0.22 | 2.38E-06 | 7.57E-06 |
| HENMT1 | DNMT3A | 4.78 | 0.05 | 0.22 | 2.40E-06 | 7.61E-06 |
| RBMX | G3BP2 | 4.78 | 0.05 | 0.22 | 2.40E-06 | 7.62E-06 |
| CMTR1 | EIF3A | 4.77 | 0.05 | 0.22 | 2.41E-06 | 7.66E-06 |
| FBLL1 | TRMT61A | 4.77 | 0.05 | 0.22 | 2.43E-06 | 7.71E-06 |
| RPUSD1 | NSUN2 | 4.77 | 0.05 | 0.22 | 2.48E-06 | 7.84E-06 |
| FTO | G3BP1 | 4.76 | 0.05 | 0.21 | 2.56E-06 | 8.09E-06 |
| HENMT1 | TRMT10C | 4.76 | 0.05 | 0.21 | 2.57E-06 | 8.14E-06 |
| TRMT10B | NKAP | 4.76 | 0.05 | 0.21 | 2.58E-06 | 8.15E-06 |
| TRMT61B | G3BP2 | 4.76 | 0.05 | 0.21 | 2.58E-06 | 8.16E-06 |
| RPUSD4 | EIF3A | 4.75 | 0.05 | 0.21 | 2.73E-06 | 8.62E-06 |
| ADAT2 | NSUN7 | 4.75 | 0.05 | 0.21 | 2.77E-06 | 8.72E-06 |
| RBM15B | METTL14 | 4.74 | 0.05 | 0.21 | 2.80E-06 | 8.83E-06 |
| TRMT10A | KIAA1429 | 4.74 | 0.05 | 0.21 | 2.83E-06 | 8.92E-06 |
| TRMT13 | KIAA1429 | 4.74 | 0.05 | 0.21 | 2.85E-06 | 8.95E-06 |
| PUS7L | SNORD48 | 4.74 | 0.05 | 0.21 | 2.90E-06 | 9.10E-06 |
| LRPPRC | RBM15B | 4.73 | 0.05 | 0.21 | 2.93E-06 | 9.18E-06 |
| RPUSD4 | DNMT3B | 4.73 | 0.05 | 0.21 | 2.93E-06 | 9.19E-06 |
| RPUSD2 | METTL5 | 4.73 | 0.05 | 0.21 | 2.94E-06 | 9.21E-06 |
| TRMT13 | RRP8 | 4.73 | 0.05 | 0.21 | 2.95E-06 | 9.25E-06 |
| NSUN5 | YTHDF1 | 4.73 | 0.05 | 0.21 | 2.97E-06 | 9.29E-06 |
| ADAT3 | ADAT2 | 4.73 | 0.05 | 0.21 | 3.00E-06 | 9.38E-06 |
| TARBP1 | YTHDF1 | 4.73 | 0.05 | 0.21 | 3.02E-06 | 9.45E-06 |
| NSUN4 | LRPPRC | 4.73 | 0.05 | 0.21 | 3.03E-06 | 9.46E-06 |
| METTL5 | WTAP | 4.73 | 0.05 | 0.21 | 3.04E-06 | 9.50E-06 |
| DKC1 | IGF2BP2 | 4.73 | 0.05 | 0.21 | 3.04E-06 | 9.50E-06 |
| CTU2 | ADAT3 | 4.71 | 0.05 | 0.21 | 3.20E-06 | 9.98E-06 |
| TRMT44 | FMR1 | 4.71 | 0.05 | 0.21 | 3.21E-06 | 1.00E-05 |
| TET1 | TRDMT1 | 4.71 | 0.05 | 0.21 | 3.22E-06 | 1.00E-05 |
| FBL | LRPPRC | 4.71 | 0.05 | 0.21 | 3.22E-06 | 1.00E-05 |
| TRMT6 | PRRC2A | 4.71 | 0.05 | 0.21 | 3.22E-06 | 1.00E-05 |
| NOP2 | NSUN6 | 4.71 | 0.05 | 0.21 | 3.23E-06 | 1.00E-05 |
| FMR1 | EIF3A | 4.71 | 0.05 | 0.21 | 3.24E-06 | 1.01E-05 |
| RPUSD4 | TET3 | 4.71 | 0.05 | 0.21 | 3.24E-06 | 1.01E-05 |
| MRM2 | FTSJ1 | 4.71 | 0.05 | 0.21 | 3.28E-06 | 1.02E-05 |
| PUS3 | HNRNPA1 | 4.71 | 0.05 | 0.21 | 3.31E-06 | 1.03E-05 |
| ALKBH1 | TRMT10C | 4.70 | 0.05 | 0.21 | 3.47E-06 | 1.07E-05 |
| PUS1 | HNRNPC | 4.70 | 0.05 | 0.21 | 3.47E-06 | 1.08E-05 |
| NSUN7 | YTHDF1 | 4.70 | 0.05 | 0.21 | 3.50E-06 | 1.08E-05 |
| WDR4 | YBX1 | 4.69 | 0.05 | 0.21 | 3.62E-06 | 1.12E-05 |
| G3BP1 | ELAVL1 | 4.69 | 0.05 | 0.21 | 3.65E-06 | 1.13E-05 |
| HENMT1 | METTL5 | 4.69 | 0.05 | 0.21 | 3.65E-06 | 1.13E-05 |
| ELP3 | METTL16 | 4.69 | 0.05 | 0.21 | 3.66E-06 | 1.13E-05 |
| PUS10 | METTL14 | 4.68 | 0.05 | 0.21 | 3.67E-06 | 1.13E-05 |
| ADAT2 | RBMX | 4.68 | 0.05 | 0.21 | 3.73E-06 | 1.15E-05 |
| RPUSD4 | TET1 | 4.68 | 0.05 | 0.21 | 3.73E-06 | 1.15E-05 |
| ALKBH8 | PUS7L | 4.68 | 0.05 | 0.21 | 3.74E-06 | 1.15E-05 |
| ELAVL1 | IGF2BP3 | 4.68 | 0.05 | 0.21 | 3.82E-06 | 1.18E-05 |
| ADAT2 | NSUN2 | 4.67 | 0.05 | 0.21 | 3.86E-06 | 1.19E-05 |
| DNMT3A | CBLL1 | 4.67 | 0.05 | 0.21 | 3.86E-06 | 1.19E-05 |
| TET1 | NSUN3 | 4.67 | 0.05 | 0.21 | 3.88E-06 | 1.19E-05 |
| DKC1 | METTL3 | 4.67 | 0.05 | 0.21 | 3.92E-06 | 1.20E-05 |
| TRMT61B | NSUN2 | 4.67 | 0.05 | 0.21 | 3.96E-06 | 1.21E-05 |
| PUS1 | SNORD48 | 4.66 | 0.05 | 0.21 | 4.11E-06 | 1.26E-05 |
| FBL | DNMT1 | 4.66 | 0.05 | 0.21 | 4.12E-06 | 1.26E-05 |
| NKAP | YTHDC1 | 4.66 | 0.05 | 0.21 | 4.12E-06 | 1.26E-05 |
| ALKBH1 | TRMT10A | 4.66 | 0.05 | 0.21 | 4.12E-06 | 1.26E-05 |
| PUS3 | RBMX | 4.66 | 0.05 | 0.21 | 4.13E-06 | 1.26E-05 |
| RPUSD4 | TGS1 | 4.65 | 0.05 | 0.21 | 4.27E-06 | 1.31E-05 |
| NSUN6 | ZCCHC4 | 4.65 | 0.05 | 0.21 | 4.31E-06 | 1.32E-05 |
| NSUN4 | METTL16 | 4.65 | 0.05 | 0.21 | 4.34E-06 | 1.32E-05 |
| RPUSD2 | FBL | 4.65 | 0.05 | 0.21 | 4.36E-06 | 1.33E-05 |
| RPUSD4 | CMTR1 | 4.65 | 0.05 | 0.21 | 4.38E-06 | 1.33E-05 |
| SNORD48 | RBMX | 4.65 | 0.05 | 0.21 | 4.41E-06 | 1.34E-05 |
| HENMT1 | IGF2BP2 | 4.65 | 0.05 | 0.21 | 4.42E-06 | 1.35E-05 |
| NSUN6 | NKAP | 4.64 | 0.05 | 0.21 | 4.43E-06 | 1.35E-05 |
| RPUSD4 | METTL16 | 4.64 | 0.05 | 0.21 | 4.49E-06 | 1.37E-05 |
| ALKBH1 | YTHDF3 | 4.64 | 0.05 | 0.21 | 4.49E-06 | 1.37E-05 |
| RPUSD4 | DNMT1 | 4.64 | 0.05 | 0.21 | 4.55E-06 | 1.38E-05 |
| NSUN2 | G3BP1 | 4.63 | 0.05 | 0.21 | 4.70E-06 | 1.43E-05 |
| G3BP1 | HNRNPA2B1 | 4.63 | 0.05 | 0.21 | 4.72E-06 | 1.43E-05 |
| METTL1 | YTHDF1 | 4.63 | 0.05 | 0.21 | 4.79E-06 | 1.45E-05 |
| RPUSD4 | CBLL1 | 4.63 | 0.05 | 0.21 | 4.84E-06 | 1.46E-05 |
| ALKBH5 | FTO | 4.62 | 0.05 | 0.21 | 4.93E-06 | 1.49E-05 |
| TET2 | CBLL1 | 4.62 | 0.05 | 0.21 | 5.01E-06 | 1.51E-05 |
| TRMT13 | DNMT1 | 4.62 | 0.05 | 0.21 | 5.04E-06 | 1.52E-05 |
| ADAR | IGF2BP3 | 4.61 | 0.05 | 0.21 | 5.10E-06 | 1.54E-05 |
| ALKBH8 | TRMT13 | 4.61 | 0.05 | 0.21 | 5.12E-06 | 1.54E-05 |
| TET1 | YTHDF1 | 4.61 | 0.05 | 0.21 | 5.16E-06 | 1.56E-05 |
| NOP2 | IGF2BP3 | 4.61 | 0.05 | 0.21 | 5.21E-06 | 1.57E-05 |
| WDR4 | TARBP1 | 4.60 | 0.05 | 0.21 | 5.33E-06 | 1.60E-05 |
| ALKBH1 | KIAA1429 | 4.60 | 0.05 | 0.21 | 5.35E-06 | 1.61E-05 |
| DKC1 | RPUSD4 | 4.60 | 0.05 | 0.21 | 5.40E-06 | 1.62E-05 |
| CMTR2 | ZCCHC4 | 4.60 | 0.05 | 0.21 | 5.44E-06 | 1.63E-05 |
| TGS1 | WTAP | 4.60 | 0.05 | 0.21 | 5.51E-06 | 1.65E-05 |
| RNMT | TRDMT1 | 4.59 | 0.05 | 0.21 | 5.76E-06 | 1.73E-05 |
| ALKBH1 | NSUN4 | 4.59 | 0.05 | 0.21 | 5.80E-06 | 1.74E-05 |
| PUS1 | IGF2BP3 | 4.58 | 0.05 | 0.21 | 5.85E-06 | 1.75E-05 |
| PUS3 | FBL | 4.58 | 0.05 | 0.21 | 6.08E-06 | 1.82E-05 |
| ALKBH8 | PRRC2A | 4.57 | 0.05 | 0.21 | 6.15E-06 | 1.84E-05 |
| IGF2BP2 | CBLL1 | 4.57 | 0.05 | 0.21 | 6.19E-06 | 1.85E-05 |
| ELP3 | NSUN4 | 4.57 | 0.05 | 0.21 | 6.32E-06 | 1.89E-05 |
| IGF2BP1 | KIAA1429 | 4.56 | 0.05 | 0.21 | 6.61E-06 | 1.97E-05 |
| ELP1 | NSUN4 | 4.55 | 0.05 | 0.21 | 6.76E-06 | 2.02E-05 |
| MRM1 | PRRC2A | 4.55 | 0.05 | 0.21 | 6.86E-06 | 2.04E-05 |
| FBL | IGF2BP3 | 4.55 | 0.05 | 0.21 | 6.89E-06 | 2.05E-05 |
| FTSJ1 | TRMT61B | 4.55 | 0.05 | 0.21 | 6.90E-06 | 2.05E-05 |
| CTU2 | DNMT3B | 4.55 | 0.05 | 0.21 | 6.92E-06 | 2.05E-05 |
| TGS1 | TRDMT1 | 4.55 | 0.05 | 0.21 | 6.95E-06 | 2.06E-05 |
| PUS7 | METTL3 | 4.54 | 0.05 | 0.21 | 7.20E-06 | 2.14E-05 |
| HNRNPA1 | ZC3H13 | 4.53 | 0.05 | 0.20 | 7.39E-06 | 2.19E-05 |
| HNRNPA1 | RBM15 | 4.53 | 0.05 | 0.20 | 7.57E-06 | 2.24E-05 |
| FTO | EIF3A | 4.53 | 0.05 | 0.20 | 7.60E-06 | 2.25E-05 |
| ADARB1 | TARBP1 | 4.52 | 0.05 | 0.20 | 7.69E-06 | 2.27E-05 |
| YTHDF3 | METTL14 | 4.52 | 0.05 | 0.20 | 7.90E-06 | 2.33E-05 |
| HNRNPA2B1 | METTL14 | 4.52 | 0.05 | 0.20 | 7.99E-06 | 2.36E-05 |
| NSUN7 | YTHDF2 | 4.51 | 0.05 | 0.20 | 8.05E-06 | 2.37E-05 |
| RNMT | NSUN6 | 4.51 | 0.05 | 0.20 | 8.11E-06 | 2.39E-05 |
| ELP3 | TET2 | 4.51 | 0.05 | 0.20 | 8.13E-06 | 2.40E-05 |
| NKAP | CBLL1 | 4.51 | 0.05 | 0.20 | 8.32E-06 | 2.45E-05 |
| WDR4 | G3BP1 | 4.51 | 0.05 | 0.20 | 8.33E-06 | 2.45E-05 |
| TRMT61A | NSUN2 | 4.50 | 0.05 | 0.20 | 8.41E-06 | 2.47E-05 |
| NOP2 | METTL3 | 4.50 | 0.05 | 0.20 | 8.41E-06 | 2.47E-05 |
| DKC1 | TRMT112 | 4.50 | 0.05 | 0.20 | 8.42E-06 | 2.47E-05 |
| NSUN2 | YTHDC1 | 4.50 | 0.05 | 0.20 | 8.42E-06 | 2.47E-05 |
| TRMT10A | ALYREF | 4.50 | 0.05 | 0.20 | 8.48E-06 | 2.49E-05 |
| PUS7L | PUS3 | 4.50 | 0.05 | 0.20 | 8.49E-06 | 2.49E-05 |
| PUS1 | HNRNPA1 | 4.50 | 0.05 | 0.20 | 8.57E-06 | 2.51E-05 |
| PUS3 | TGS1 | 4.50 | 0.05 | 0.20 | 8.59E-06 | 2.51E-05 |
| CMTR1 | NSUN4 | 4.50 | 0.05 | 0.20 | 8.62E-06 | 2.52E-05 |
| ELAVL1 | IGF2BP2 | 4.50 | 0.05 | 0.20 | 8.75E-06 | 2.56E-05 |
| METTL1 | YBX1 | 4.48 | 0.05 | 0.20 | 9.25E-06 | 2.70E-05 |
| TET1 | NSUN7 | 4.48 | 0.05 | 0.20 | 9.30E-06 | 2.71E-05 |
| DNMT3A | ALKBH5 | 4.48 | 0.05 | 0.20 | 9.31E-06 | 2.71E-05 |
| TRMT10A | YTHDF2 | 4.48 | 0.05 | 0.20 | 9.31E-06 | 2.71E-05 |
| CMTR1 | TRMT6 | 4.48 | 0.05 | 0.20 | 9.43E-06 | 2.75E-05 |
| TRMT44 | YTHDF2 | 4.48 | 0.05 | 0.20 | 9.48E-06 | 2.76E-05 |
| RPUSD1 | YTHDF1 | 4.48 | 0.05 | 0.20 | 9.56E-06 | 2.78E-05 |
| RPUSD2 | TRMT61A | 4.47 | 0.05 | 0.20 | 9.66E-06 | 2.81E-05 |
| TARBP1 | DNMT1 | 4.47 | 0.05 | 0.20 | 9.83E-06 | 2.85E-05 |
| CMTR2 | TET2 | 4.47 | 0.05 | 0.20 | 9.83E-06 | 2.86E-05 |
| RBM15B | KIAA1429 | 4.47 | 0.05 | 0.20 | 9.89E-06 | 2.87E-05 |
| PUS3 | RBM15 | 4.46 | 0.05 | 0.20 | 1.02E-05 | 2.95E-05 |
| TRMT61B | IGF2BP1 | 4.46 | 0.05 | 0.20 | 1.02E-05 | 2.96E-05 |
| TRMT11 | NSUN3 | 4.46 | 0.05 | 0.20 | 1.03E-05 | 2.97E-05 |
| NSUN3 | ZCCHC4 | 4.46 | 0.05 | 0.20 | 1.03E-05 | 2.98E-05 |
| TRMT13 | WTAP | 4.46 | 0.05 | 0.20 | 1.05E-05 | 3.03E-05 |
| RNMT | FTO | 4.45 | 0.05 | 0.20 | 1.06E-05 | 3.06E-05 |
| ADAT3 | DNMT3B | 4.45 | 0.05 | 0.20 | 1.08E-05 | 3.13E-05 |
| MRM3 | NSUN5 | 4.45 | 0.05 | 0.20 | 1.09E-05 | 3.13E-05 |
| TRMT6 | HNRNPA1 | 4.45 | 0.05 | 0.20 | 1.09E-05 | 3.13E-05 |
| TRMT13 | CBLL1 | 4.45 | 0.05 | 0.20 | 1.09E-05 | 3.14E-05 |
| CMTR2 | FMR1 | 4.44 | 0.05 | 0.20 | 1.14E-05 | 3.27E-05 |
| FBL | TRMT61A | 4.43 | 0.05 | 0.20 | 1.15E-05 | 3.30E-05 |
| TRMT61B | TRDMT1 | 4.43 | 0.05 | 0.20 | 1.16E-05 | 3.33E-05 |
| IGF2BP1 | METTL5 | 4.43 | 0.05 | 0.20 | 1.17E-05 | 3.37E-05 |
| WDR4 | TRMT13 | 4.43 | 0.05 | 0.20 | 1.17E-05 | 3.37E-05 |
| ALKBH8 | MRM2 | 4.43 | 0.05 | 0.20 | 1.18E-05 | 3.37E-05 |
| RBMX | EIF3A | 4.42 | 0.05 | 0.20 | 1.21E-05 | 3.45E-05 |
| ADARB1 | RNMT | 4.42 | 0.05 | 0.20 | 1.23E-05 | 3.51E-05 |
| TRMT10C | NOP2 | 4.42 | 0.05 | 0.20 | 1.23E-05 | 3.51E-05 |
| TRMT44 | DNMT1 | 4.42 | 0.05 | 0.20 | 1.24E-05 | 3.53E-05 |
| HENMT1 | CMTR1 | 4.41 | 0.05 | 0.20 | 1.26E-05 | 3.61E-05 |
| RPUSD1 | FBL | 4.41 | 0.05 | 0.20 | 1.29E-05 | 3.68E-05 |
| TRUB1 | IGF2BP3 | 4.41 | 0.05 | 0.20 | 1.30E-05 | 3.69E-05 |
| MRM2 | TRMT61A | 4.41 | 0.05 | 0.20 | 1.30E-05 | 3.71E-05 |
| TRMT13 | YTHDF3 | 4.41 | 0.05 | 0.20 | 1.31E-05 | 3.72E-05 |
| RBMX | METTL5 | 4.40 | 0.05 | 0.20 | 1.32E-05 | 3.76E-05 |
| TRMT10C | DNMT3B | 4.40 | 0.05 | 0.20 | 1.33E-05 | 3.77E-05 |
| ADAR | HNRNPC | 4.40 | 0.05 | 0.20 | 1.33E-05 | 3.77E-05 |
| CTU1 | RPUSD2 | 4.40 | 0.05 | 0.20 | 1.34E-05 | 3.80E-05 |
| NSUN5 | CBLL1 | 4.40 | 0.05 | 0.20 | 1.36E-05 | 3.85E-05 |
| TRDMT1 | WTAP | 4.39 | 0.05 | 0.20 | 1.43E-05 | 4.04E-05 |
| CMTR1 | RBM15B | 4.38 | 0.05 | 0.20 | 1.45E-05 | 4.10E-05 |
| TRMT112 | MRM2 | 4.38 | 0.05 | 0.20 | 1.47E-05 | 4.16E-05 |
| ELP1 | YTHDF2 | 4.38 | 0.05 | 0.20 | 1.47E-05 | 4.17E-05 |
| TET2 | G3BP1 | 4.38 | 0.05 | 0.20 | 1.49E-05 | 4.20E-05 |
| WDR4 | TET1 | 4.37 | 0.05 | 0.20 | 1.50E-05 | 4.24E-05 |
| TRMT10C | YTHDF2 | 4.37 | 0.05 | 0.20 | 1.53E-05 | 4.33E-05 |
| TRMT10A | NSUN4 | 4.37 | 0.05 | 0.20 | 1.55E-05 | 4.38E-05 |
| ADAT3 | DNMT3A | 4.37 | 0.05 | 0.20 | 1.56E-05 | 4.39E-05 |
| NOP2 | HNRNPC | 4.36 | 0.05 | 0.20 | 1.60E-05 | 4.49E-05 |
| FMR1 | IGF2BP3 | 4.36 | 0.05 | 0.20 | 1.60E-05 | 4.51E-05 |
| PRRC2A | METTL3 | 4.36 | 0.05 | 0.20 | 1.60E-05 | 4.51E-05 |
| ELP1 | TRMT6 | 4.36 | 0.05 | 0.20 | 1.60E-05 | 4.51E-05 |
| RPUSD3 | RRP8 | 4.36 | 0.05 | 0.20 | 1.62E-05 | 4.54E-05 |
| FTSJ1 | ELAVL1 | 4.36 | 0.05 | 0.20 | 1.63E-05 | 4.59E-05 |
| NSUN6 | KIAA1429 | 4.35 | 0.05 | 0.20 | 1.66E-05 | 4.66E-05 |
| G3BP2 | HNRNPC | 4.35 | 0.05 | 0.20 | 1.67E-05 | 4.67E-05 |
| TRMT10B | METTL16 | 4.35 | 0.05 | 0.20 | 1.67E-05 | 4.67E-05 |
| DNMT1 | NSUN7 | 4.35 | 0.05 | 0.20 | 1.69E-05 | 4.74E-05 |
| G3BP1 | YTHDF2 | 4.35 | 0.05 | 0.20 | 1.69E-05 | 4.74E-05 |
| HNRNPA2B1 | ZCCHC4 | 4.35 | 0.05 | 0.20 | 1.70E-05 | 4.74E-05 |
| DNMT3B | NKAP | 4.34 | 0.05 | 0.20 | 1.71E-05 | 4.78E-05 |
| BUD23 | HNRNPA2B1 | 4.34 | 0.05 | 0.20 | 1.72E-05 | 4.80E-05 |
| TGS1 | HNRNPA2B1 | 4.34 | 0.05 | 0.20 | 1.75E-05 | 4.88E-05 |
| RPUSD4 | TRMT6 | 4.33 | 0.05 | 0.20 | 1.79E-05 | 4.99E-05 |
| PUS10 | TET2 | 4.33 | 0.05 | 0.20 | 1.81E-05 | 5.05E-05 |
| TET3 | NSUN7 | 4.33 | 0.05 | 0.20 | 1.81E-05 | 5.05E-05 |
| NSUN7 | YTHDC2 | 4.33 | 0.05 | 0.20 | 1.85E-05 | 5.16E-05 |
| ALKBH8 | NSUN2 | 4.32 | 0.05 | 0.20 | 1.88E-05 | 5.24E-05 |
| NSUN6 | PRRC2A | 4.32 | 0.05 | 0.20 | 1.89E-05 | 5.26E-05 |
| ADAT2 | DNMT1 | 4.32 | 0.05 | 0.20 | 1.89E-05 | 5.26E-05 |
| ELP1 | TRMT44 | 4.32 | 0.05 | 0.20 | 1.90E-05 | 5.29E-05 |
| BUD23 | TRMT10C | 4.32 | 0.05 | 0.20 | 1.91E-05 | 5.29E-05 |
| DKC1 | CMTR1 | 4.32 | 0.05 | 0.20 | 1.92E-05 | 5.33E-05 |
| TRMT11 | LRPPRC | 4.32 | 0.05 | 0.20 | 1.93E-05 | 5.35E-05 |
| TGS1 | ELAVL1 | 4.32 | 0.05 | 0.20 | 1.93E-05 | 5.36E-05 |
| FBL | NSUN2 | 4.32 | 0.05 | 0.20 | 1.93E-05 | 5.36E-05 |
| FTSJ3 | TRMT6 | 4.32 | 0.05 | 0.20 | 1.94E-05 | 5.36E-05 |
| YBX1 | NSUN2 | 4.31 | 0.05 | 0.20 | 1.98E-05 | 5.49E-05 |
| NSUN3 | RBM15 | 4.31 | 0.05 | 0.20 | 2.00E-05 | 5.52E-05 |
| DNMT3A | NSUN5 | 4.31 | 0.05 | 0.20 | 2.00E-05 | 5.52E-05 |
| ALKBH8 | ZCCHC4 | 4.30 | 0.05 | 0.19 | 2.04E-05 | 5.63E-05 |
| WDR4 | METTL5 | 4.29 | 0.05 | 0.19 | 2.15E-05 | 5.93E-05 |
| RNMT | ELAVL1 | 4.29 | 0.05 | 0.19 | 2.15E-05 | 5.93E-05 |
| RPUSD4 | RBM15B | 4.29 | 0.05 | 0.19 | 2.15E-05 | 5.93E-05 |
| ALYREF | PRRC2A | 4.29 | 0.05 | 0.19 | 2.19E-05 | 6.03E-05 |
| RBM15B | RBM15 | 4.29 | 0.05 | 0.19 | 2.21E-05 | 6.11E-05 |
| TGS1 | DNMT3A | 4.28 | 0.05 | 0.19 | 2.26E-05 | 6.23E-05 |
| RPUSD4 | RBM15 | 4.28 | 0.05 | 0.19 | 2.27E-05 | 6.24E-05 |
| TRUB1 | RNMT | 4.28 | 0.05 | 0.19 | 2.28E-05 | 6.28E-05 |
| NSUN5 | IGF2BP3 | 4.28 | 0.05 | 0.19 | 2.30E-05 | 6.32E-05 |
| ALKBH8 | METTL16 | 4.27 | 0.05 | 0.19 | 2.40E-05 | 6.58E-05 |
| ADARB1 | TET1 | 4.27 | 0.05 | 0.19 | 2.41E-05 | 6.62E-05 |
| NSUN4 | METTL3 | 4.26 | 0.05 | 0.19 | 2.45E-05 | 6.72E-05 |
| CBLL1 | RBM15B | 4.26 | 0.05 | 0.19 | 2.50E-05 | 6.83E-05 |
| ELP1 | ALKBH5 | 4.25 | 0.05 | 0.19 | 2.56E-05 | 6.99E-05 |
| PUS7L | TRMT10C | 4.25 | 0.05 | 0.19 | 2.57E-05 | 7.02E-05 |
| FTSJ1 | TRMT6 | 4.25 | 0.05 | 0.19 | 2.60E-05 | 7.09E-05 |
| MRM3 | ALYREF | 4.25 | 0.05 | 0.19 | 2.62E-05 | 7.14E-05 |
| NSUN2 | IGF2BP2 | 4.25 | 0.05 | 0.19 | 2.62E-05 | 7.14E-05 |
| TRMT11 | TRMT61B | 4.24 | 0.05 | 0.19 | 2.69E-05 | 7.34E-05 |
| ALKBH1 | RRP8 | 4.24 | 0.05 | 0.19 | 2.73E-05 | 7.43E-05 |
| SNORD48 | NOP2 | 4.23 | 0.05 | 0.19 | 2.76E-05 | 7.51E-05 |
| MRM1 | RBM15B | 4.23 | 0.05 | 0.19 | 2.79E-05 | 7.59E-05 |
| FTSJ3 | G3BP1 | 4.23 | 0.05 | 0.19 | 2.81E-05 | 7.62E-05 |
| RPUSD4 | HNRNPA1 | 4.23 | 0.05 | 0.19 | 2.81E-05 | 7.63E-05 |
| TRMT11 | NSUN2 | 4.23 | 0.05 | 0.19 | 2.84E-05 | 7.70E-05 |
| ELP3 | TRUB1 | 4.22 | 0.05 | 0.19 | 2.88E-05 | 7.79E-05 |
| ALYREF | RBM15 | 4.22 | 0.05 | 0.19 | 2.88E-05 | 7.79E-05 |
| TRMT13 | TRMT10C | 4.22 | 0.05 | 0.19 | 2.96E-05 | 8.00E-05 |
| FTSJ3 | TRMT44 | 4.21 | 0.05 | 0.19 | 3.01E-05 | 8.15E-05 |
| NKAP | METTL14 | 4.21 | 0.05 | 0.19 | 3.02E-05 | 8.17E-05 |
| DKC1 | TRMT10A | 4.21 | 0.05 | 0.19 | 3.06E-05 | 8.26E-05 |
| SNORD48 | RBM15 | 4.21 | 0.05 | 0.19 | 3.06E-05 | 8.26E-05 |
| ALKBH8 | WDR4 | 4.20 | 0.05 | 0.19 | 3.13E-05 | 8.44E-05 |
| TGS1 | HNRNPA1 | 4.20 | 0.05 | 0.19 | 3.13E-05 | 8.45E-05 |
| TARBP1 | TRMT61A | 4.20 | 0.05 | 0.19 | 3.19E-05 | 8.59E-05 |
| TET1 | ZCCHC4 | 4.20 | 0.05 | 0.19 | 3.19E-05 | 8.60E-05 |
| WDR4 | RBMX | 4.20 | 0.05 | 0.19 | 3.23E-05 | 8.70E-05 |
| DNMT3B | HNRNPA1 | 4.20 | 0.05 | 0.19 | 3.25E-05 | 8.74E-05 |
| FTSJ3 | FMR1 | 4.20 | 0.05 | 0.19 | 3.25E-05 | 8.74E-05 |
| CMTR1 | NSUN3 | 4.19 | 0.05 | 0.19 | 3.26E-05 | 8.77E-05 |
| ADARB1 | YTHDC1 | 4.19 | 0.05 | 0.19 | 3.29E-05 | 8.84E-05 |
| FTSJ3 | TRMT61B | 4.19 | 0.05 | 0.19 | 3.33E-05 | 8.94E-05 |
| PUS7L | HNRNPC | 4.19 | 0.05 | 0.19 | 3.37E-05 | 9.03E-05 |
| ELP3 | RBMX | 4.18 | 0.05 | 0.19 | 3.45E-05 | 9.25E-05 |
| ALYREF | HNRNPA1 | 4.18 | 0.05 | 0.19 | 3.50E-05 | 9.37E-05 |
| PRRC2A | IGF2BP2 | 4.18 | 0.05 | 0.19 | 3.52E-05 | 9.43E-05 |
| PUS7L | PUS1 | 4.17 | 0.05 | 0.19 | 3.56E-05 | 9.53E-05 |
| ELP1 | METTL3 | 4.17 | 0.05 | 0.19 | 3.64E-05 | 9.71E-05 |
| FTSJ1 | NOP2 | 4.17 | 0.05 | 0.19 | 3.64E-05 | 9.71E-05 |
| CMTR2 | NKAP | 4.17 | 0.05 | 0.19 | 3.66E-05 | 9.78E-05 |
| PUS3 | ALKBH1 | 4.17 | 0.05 | 0.19 | 3.67E-05 | 9.79E-05 |
| ELP1 | ADARB2 | 4.16 | 0.05 | 0.19 | 3.82E-05 | 0.00010162 |
| TRMT11 | HENMT1 | 4.16 | 0.05 | 0.19 | 3.84E-05 | 0.00010212 |
| RPUSD2 | BUD23 | 4.16 | 0.05 | 0.19 | 3.87E-05 | 0.0001028 |
| TET1 | METTL16 | 4.15 | 0.05 | 0.19 | 3.91E-05 | 0.00010404 |
| PUS7 | FTSJ1 | 4.15 | 0.05 | 0.19 | 3.97E-05 | 0.00010538 |
| RPUSD3 | NOP2 | 4.15 | 0.05 | 0.19 | 4.02E-05 | 0.00010676 |
| RPUSD3 | RPUSD1 | 4.14 | 0.05 | 0.19 | 4.04E-05 | 0.0001071 |
| CTU2 | METTL3 | 4.14 | 0.05 | 0.19 | 4.06E-05 | 0.0001077 |
| FTSJ3 | FTSJ1 | 4.14 | 0.05 | 0.19 | 4.07E-05 | 0.00010788 |
| TRMT61B | G3BP1 | 4.14 | 0.05 | 0.19 | 4.08E-05 | 0.00010804 |
| DKC1 | RPUSD1 | 4.14 | 0.05 | 0.19 | 4.08E-05 | 0.00010808 |
| ELAVL1 | YTHDC1 | 4.14 | 0.05 | 0.19 | 4.11E-05 | 0.00010888 |
| TRMT61B | FMR1 | 4.14 | 0.05 | 0.19 | 4.12E-05 | 0.00010905 |
| FBL | TRMT61B | 4.14 | 0.05 | 0.19 | 4.17E-05 | 0.00011013 |
| RPUSD4 | FTSJ3 | 4.14 | 0.05 | 0.19 | 4.19E-05 | 0.00011068 |
| PUS1 | FBLL1 | 4.14 | 0.05 | 0.19 | 4.20E-05 | 0.00011093 |
| ELAVL1 | YTHDF2 | 4.13 | 0.05 | 0.19 | 4.35E-05 | 0.00011478 |
| ADARB1 | DNMT1 | 4.13 | 0.05 | 0.19 | 4.37E-05 | 0.0001152 |
| NSUN5 | PRRC2A | 4.13 | 0.05 | 0.19 | 4.38E-05 | 0.00011546 |
| CTU1 | FTSJ1 | 4.13 | 0.05 | 0.19 | 4.38E-05 | 0.00011546 |
| ADARB2 | TRDMT1 | 4.12 | 0.05 | 0.19 | 4.41E-05 | 0.00011619 |
| HENMT1 | DNMT1 | 4.12 | 0.05 | 0.19 | 4.47E-05 | 0.00011772 |
| WDR4 | KIAA1429 | 4.12 | 0.05 | 0.19 | 4.51E-05 | 0.00011872 |
| YBX1 | DNMT3B | 4.12 | 0.05 | 0.19 | 4.52E-05 | 0.0001189 |
| PUS1 | TRMT61B | 4.12 | 0.05 | 0.19 | 4.57E-05 | 0.00012017 |
| PUS3 | KIAA1429 | 4.11 | 0.05 | 0.19 | 4.59E-05 | 0.00012056 |
| METTL1 | NKAP | 4.11 | 0.05 | 0.19 | 4.60E-05 | 0.00012082 |
| ADAT3 | NSUN7 | 4.11 | 0.05 | 0.19 | 4.64E-05 | 0.0001218 |
| ALKBH1 | NSUN7 | 4.10 | 0.05 | 0.19 | 4.78E-05 | 0.00012525 |
| DNMT3B | HNRNPC | 4.10 | 0.05 | 0.19 | 4.79E-05 | 0.00012544 |
| TGS1 | WDR4 | 4.10 | 0.05 | 0.19 | 4.84E-05 | 0.00012658 |
| YTHDC2 | METTL16 | 4.10 | 0.05 | 0.19 | 4.84E-05 | 0.00012669 |
| MRM2 | YTHDF2 | 4.10 | 0.05 | 0.19 | 4.87E-05 | 0.00012735 |
| RPUSD4 | NOP2 | 4.10 | 0.05 | 0.19 | 4.92E-05 | 0.00012857 |
| RPUSD4 | METTL3 | 4.10 | 0.05 | 0.19 | 4.93E-05 | 0.0001288 |
| RPUSD3 | DNMT3B | 4.09 | 0.05 | 0.19 | 5.08E-05 | 0.00013252 |
| TRMT13 | LRPPRC | 4.09 | 0.05 | 0.19 | 5.10E-05 | 0.00013302 |
| SNORD48 | NSUN2 | 4.09 | 0.05 | 0.19 | 5.17E-05 | 0.00013469 |
| TRMT61B | NSUN7 | 4.08 | 0.05 | 0.19 | 5.22E-05 | 0.00013585 |
| ALKBH8 | FTO | 4.08 | 0.05 | 0.19 | 5.32E-05 | 0.00013823 |
| PUS7 | HNRNPA1 | 4.08 | 0.05 | 0.19 | 5.35E-05 | 0.00013909 |
| TRDMT1 | METTL14 | 4.07 | 0.05 | 0.18 | 5.42E-05 | 0.00014084 |
| FTO | NKAP | 4.07 | 0.05 | 0.18 | 5.46E-05 | 0.00014161 |
| NSUN2 | NKAP | 4.07 | 0.05 | 0.18 | 5.52E-05 | 0.00014324 |
| ADAR | ALKBH5 | 4.07 | 0.05 | 0.18 | 5.59E-05 | 0.00014495 |
| WDR4 | YTHDF1 | 4.06 | 0.05 | 0.18 | 5.64E-05 | 0.00014596 |
| ADARB1 | METTL16 | 4.06 | 0.05 | 0.18 | 5.77E-05 | 0.00014938 |
| TET2 | METTL3 | 4.06 | 0.05 | 0.18 | 5.83E-05 | 0.00015066 |
| CBLL1 | METTL5 | 4.06 | 0.05 | 0.18 | 5.87E-05 | 0.00015163 |
| PUS1 | TET1 | 4.05 | 0.05 | 0.18 | 6.08E-05 | 0.00015701 |
| RNMT | TRMT10B | 4.05 | 0.05 | 0.18 | 6.10E-05 | 0.00015749 |
| FMR1 | YTHDF3 | 4.04 | 0.05 | 0.18 | 6.13E-05 | 0.00015804 |
| ALKBH1 | METTL14 | 4.04 | 0.05 | 0.18 | 6.17E-05 | 0.0001589 |
| ALKBH5 | ZC3H13 | 4.04 | 0.05 | 0.18 | 6.30E-05 | 0.0001621 |
| MRM3 | ALKBH1 | 4.03 | 0.05 | 0.18 | 6.41E-05 | 0.00016492 |
| RBMX | YTHDF1 | 4.03 | 0.05 | 0.18 | 6.42E-05 | 0.00016504 |
| CTU1 | TRMT6 | 4.03 | 0.05 | 0.18 | 6.47E-05 | 0.00016617 |
| DNMT1 | METTL14 | 4.03 | 0.05 | 0.18 | 6.48E-05 | 0.00016635 |
| ALKBH8 | TRMT10B | 4.03 | 0.05 | 0.18 | 6.55E-05 | 0.00016796 |
| IGF2BP3 | YTHDF3 | 4.03 | 0.05 | 0.18 | 6.62E-05 | 0.00016974 |
| TRMT61A | RBM15B | 4.02 | 0.05 | 0.18 | 6.74E-05 | 0.00017283 |
| IGF2BP3 | RBM15 | 4.02 | 0.05 | 0.18 | 6.79E-05 | 0.00017376 |
| ALKBH5 | YTHDF1 | 4.02 | 0.05 | 0.18 | 6.81E-05 | 0.00017435 |
| CMTR1 | ELAVL1 | 4.02 | 0.05 | 0.18 | 6.86E-05 | 0.0001756 |
| FBL | IGF2BP2 | 4.01 | 0.05 | 0.18 | 6.93E-05 | 0.00017715 |
| ADARB1 | EIF3A | 4.01 | 0.05 | 0.18 | 6.96E-05 | 0.0001779 |
| FBL | HNRNPA2B1 | 4.01 | 0.05 | 0.18 | 7.07E-05 | 0.0001806 |
| PUS7 | YBX1 | 4.01 | 0.05 | 0.18 | 7.08E-05 | 0.00018068 |
| CMTR2 | YTHDC2 | 4.01 | 0.05 | 0.18 | 7.09E-05 | 0.00018071 |
| G3BP2 | YTHDF2 | 4.01 | 0.05 | 0.18 | 7.10E-05 | 0.00018085 |
| TET3 | HNRNPA1 | 4.01 | 0.05 | 0.18 | 7.10E-05 | 0.00018085 |
| PUS7L | HENMT1 | 4.00 | 0.05 | 0.18 | 7.22E-05 | 0.00018384 |
| FTSJ1 | TRMT10C | 4.00 | 0.05 | 0.18 | 7.29E-05 | 0.00018553 |
| BUD23 | FBL | 4.00 | 0.05 | 0.18 | 7.43E-05 | 0.00018877 |
| ADAR | METTL16 | 3.99 | 0.05 | 0.18 | 7.54E-05 | 0.0001914 |
| CTU1 | DKC1 | 3.99 | 0.05 | 0.18 | 7.60E-05 | 0.00019275 |
| CMTR1 | TRDMT1 | 3.99 | 0.05 | 0.18 | 7.68E-05 | 0.0001947 |
| CTU1 | YTHDF1 | 3.99 | 0.05 | 0.18 | 7.72E-05 | 0.00019551 |
| TRMT10C | IGF2BP1 | 3.98 | 0.05 | 0.18 | 7.84E-05 | 0.00019863 |
| TRMT10B | DNMT3B | 3.98 | 0.05 | 0.18 | 7.97E-05 | 0.00020176 |
| HNRNPC | CBLL1 | 3.98 | 0.05 | 0.18 | 7.98E-05 | 0.00020185 |
| MRM2 | DNMT3B | 3.98 | 0.05 | 0.18 | 8.10E-05 | 0.0002048 |
| RNMT | HENMT1 | 3.98 | 0.05 | 0.18 | 8.13E-05 | 0.0002053 |
| DNMT3A | METTL16 | 3.97 | 0.05 | 0.18 | 8.21E-05 | 0.00020731 |
| WDR4 | EIF3A | 3.97 | 0.05 | 0.18 | 8.23E-05 | 0.00020765 |
| ALKBH1 | CBLL1 | 3.97 | 0.05 | 0.18 | 8.23E-05 | 0.00020765 |
| CMTR2 | YTHDC1 | 3.97 | 0.05 | 0.18 | 8.24E-05 | 0.00020777 |
| ADARB1 | PRRC2A | 3.97 | 0.05 | 0.18 | 8.40E-05 | 0.00021171 |
| FTSJ1 | TRMT61A | 3.96 | 0.05 | 0.18 | 8.58E-05 | 0.00021605 |
| ALKBH5 | YTHDC1 | 3.96 | 0.05 | 0.18 | 8.64E-05 | 0.00021747 |
| NSUN5 | NKAP | 3.96 | 0.05 | 0.18 | 8.72E-05 | 0.00021933 |
| TRDMT1 | ZCCHC4 | 3.96 | 0.05 | 0.18 | 8.76E-05 | 0.0002202 |
| ELP1 | WDR4 | 3.96 | 0.05 | 0.18 | 8.79E-05 | 0.00022086 |
| RPUSD3 | FBL | 3.95 | 0.05 | 0.18 | 9.01E-05 | 0.00022604 |
| CMTR2 | CMTR1 | 3.95 | 0.05 | 0.18 | 9.03E-05 | 0.00022644 |
| PUS10 | METTL3 | 3.95 | 0.05 | 0.18 | 9.18E-05 | 0.00023019 |
| RPUSD2 | ALKBH1 | 3.95 | 0.05 | 0.18 | 9.18E-05 | 0.00023019 |
| TRMT11 | SNORD48 | 3.94 | 0.05 | 0.18 | 9.23E-05 | 0.00023122 |
| PUS7L | NKAP | 3.94 | 0.05 | 0.18 | 9.43E-05 | 0.00023607 |
| SNORD48 | HNRNPA2B1 | 3.94 | 0.05 | 0.18 | 9.45E-05 | 0.00023641 |
| HENMT1 | HNRNPA2B1 | 3.94 | 0.05 | 0.18 | 9.48E-05 | 0.00023691 |
| PUS3 | MRM2 | 3.94 | 0.05 | 0.18 | 9.48E-05 | 0.00023691 |
| RPUSD4 | NSUN2 | 3.94 | 0.05 | 0.18 | 9.48E-05 | 0.00023691 |
| PUS7 | FMR1 | 3.94 | 0.05 | 0.18 | 9.50E-05 | 0.00023735 |
| ADAR | TRUB1 | 3.93 | 0.05 | 0.18 | 9.63E-05 | 0.00024047 |
| MRM3 | HNRNPC | 3.93 | 0.05 | 0.18 | 9.65E-05 | 0.00024085 |
| FTSJ3 | TARBP1 | 3.92 | 0.05 | 0.18 | 0.00010127 | 0.00025255 |
| MRM1 | ALYREF | 3.92 | 0.05 | 0.18 | 0.00010208 | 0.00025444 |
| TRMT11 | TRDMT1 | 3.92 | 0.05 | 0.18 | 0.00010241 | 0.00025514 |
| TRDMT1 | NSUN4 | 3.92 | 0.05 | 0.18 | 0.00010341 | 0.0002575 |
| PUS7 | SNORD48 | 3.91 | 0.05 | 0.18 | 0.0001052 | 0.0002617 |
| TARBP1 | PRRC2A | 3.91 | 0.05 | 0.18 | 0.00010581 | 0.00026309 |
| ADAT2 | ADARB1 | 3.91 | 0.05 | 0.18 | 0.00010593 | 0.00026327 |
| MRM2 | TRMT6 | 3.91 | 0.05 | 0.18 | 0.00010602 | 0.00026336 |
| RPUSD2 | TRUB1 | 3.91 | 0.05 | 0.18 | 0.00010639 | 0.00026414 |
| TARBP1 | TRDMT1 | 3.91 | 0.05 | 0.18 | 0.00010763 | 0.00026696 |
| TRMT6 | NSUN6 | 3.90 | 0.05 | 0.18 | 0.00011211 | 0.00027767 |
| PRRC2A | IGF2BP1 | 3.89 | 0.05 | 0.18 | 0.00011271 | 0.00027902 |
| RPUSD4 | MRM1 | 3.89 | 0.05 | 0.18 | 0.00011322 | 0.00028013 |
| G3BP1 | WTAP | 3.89 | 0.05 | 0.18 | 0.00011372 | 0.00028124 |
| RPUSD2 | NSUN5 | 3.89 | 0.05 | 0.18 | 0.0001139 | 0.00028153 |
| ADAT3 | NSUN6 | 3.89 | 0.05 | 0.18 | 0.00011469 | 0.00028335 |
| ADARB2 | CMTR1 | 3.89 | 0.05 | 0.18 | 0.00011518 | 0.00028442 |
| CTU1 | ALYREF | 3.89 | 0.05 | 0.18 | 0.00011613 | 0.0002865 |
| PUS7L | TRMT10A | 3.89 | 0.05 | 0.18 | 0.0001164 | 0.00028701 |
| DKC1 | RBM15B | 3.88 | 0.05 | 0.18 | 0.00011746 | 0.0002895 |
| TRMT61A | RBM15 | 3.88 | 0.05 | 0.18 | 0.00011784 | 0.00029028 |
| NSUN3 | YTHDC2 | 3.88 | 0.05 | 0.18 | 0.0001181 | 0.00029078 |
| CTU1 | RPUSD3 | 3.88 | 0.05 | 0.18 | 0.00011858 | 0.00029183 |
| CMTR2 | CBLL1 | 3.88 | 0.05 | 0.18 | 0.00011944 | 0.00029379 |
| RPUSD4 | YTHDC1 | 3.88 | 0.05 | 0.18 | 0.00011962 | 0.00029409 |
| PUS7 | TRMT11 | 3.88 | 0.05 | 0.18 | 0.00012072 | 0.00029666 |
| TRMT61B | METTL3 | 3.87 | 0.05 | 0.18 | 0.00012423 | 0.00030484 |
| TRMT10B | TET1 | 3.87 | 0.05 | 0.18 | 0.00012432 | 0.0003049 |
| ALKBH5 | RBM15B | 3.87 | 0.05 | 0.18 | 0.00012603 | 0.0003089 |
| BUD23 | TRMT10A | 3.87 | 0.05 | 0.18 | 0.00012673 | 0.00031037 |
| PUS7L | CMTR2 | 3.86 | 0.05 | 0.18 | 0.0001273 | 0.00031161 |
| ELP1 | NSUN7 | 3.86 | 0.05 | 0.18 | 0.00012893 | 0.00031545 |
| DKC1 | TRMT13 | 3.86 | 0.05 | 0.18 | 0.00012919 | 0.00031594 |
| TRMT112 | ALKBH1 | 3.85 | 0.05 | 0.18 | 0.00013244 | 0.00032372 |
| ALKBH8 | IGF2BP2 | 3.85 | 0.05 | 0.18 | 0.00013318 | 0.00032523 |
| MRM1 | TRMT44 | 3.85 | 0.05 | 0.17 | 0.0001367 | 0.00033365 |
| IGF2BP2 | KIAA1429 | 3.85 | 0.05 | 0.17 | 0.00013704 | 0.00033431 |
| LRPPRC | WTAP | 3.85 | 0.05 | 0.17 | 0.0001371 | 0.00033431 |
| MRM2 | CMTR1 | 3.84 | 0.05 | 0.17 | 0.00014021 | 0.00034173 |
| PUS7L | TRMT44 | 3.84 | 0.05 | 0.17 | 0.00014103 | 0.00034355 |
| DKC1 | TRMT11 | 3.83 | 0.05 | 0.17 | 0.00014335 | 0.00034904 |
| PUS3 | YBX1 | 3.83 | 0.05 | 0.17 | 0.00014437 | 0.00035137 |
| ADAR | NKAP | 3.83 | 0.05 | 0.17 | 0.00014473 | 0.00035206 |
| TRMT13 | NSUN2 | 3.83 | 0.05 | 0.17 | 0.00014537 | 0.00035345 |
| MRM2 | METTL5 | 3.83 | 0.05 | 0.17 | 0.00014572 | 0.00035413 |
| MRM1 | TRMT10B | 3.83 | 0.05 | 0.17 | 0.00014652 | 0.00035589 |
| PUS3 | NSUN2 | 3.83 | 0.05 | 0.17 | 0.00014792 | 0.00035914 |
| MRM2 | NKAP | 3.82 | 0.05 | 0.17 | 0.00014898 | 0.00036154 |
| TRMT11 | TRMT6 | 3.82 | 0.05 | 0.17 | 0.00015336 | 0.00037144 |
| ELP1 | NKAP | 3.82 | 0.05 | 0.17 | 0.00015369 | 0.00037208 |
| CBLL1 | WTAP | 3.81 | 0.05 | 0.17 | 0.00015631 | 0.00037822 |
| RNMT | NKAP | 3.81 | 0.05 | 0.17 | 0.00015979 | 0.00038647 |
| FTO | FMR1 | 3.80 | 0.05 | 0.17 | 0.00016115 | 0.00038956 |
| TRMT10B | NSUN4 | 3.80 | 0.05 | 0.17 | 0.00016207 | 0.00039161 |
| ELP3 | ADARB2 | 3.80 | 0.05 | 0.17 | 0.00016378 | 0.00039555 |
| ADAT2 | BUD23 | 3.80 | 0.05 | 0.17 | 0.00016518 | 0.00039854 |
| PUS7 | NSUN6 | 3.80 | 0.05 | 0.17 | 0.00016557 | 0.00039929 |
| ALYREF | RBMX | 3.80 | 0.05 | 0.17 | 0.00016635 | 0.000401 |
| TRUB1 | TRMT13 | 3.80 | 0.05 | 0.17 | 0.00016698 | 0.00040231 |
| ALKBH1 | TET1 | 3.79 | 0.05 | 0.17 | 0.00016902 | 0.00040684 |
| PUS7L | WDR4 | 3.79 | 0.05 | 0.17 | 0.00016928 | 0.00040727 |
| PUS3 | MRM3 | 3.79 | 0.05 | 0.17 | 0.00017005 | 0.00040892 |
| RRP8 | YTHDC2 | 3.79 | 0.05 | 0.17 | 0.00017047 | 0.00040974 |
| CBLL1 | ZCCHC4 | 3.78 | 0.05 | 0.17 | 0.00017668 | 0.00042447 |
| NSUN2 | HNRNPC | 3.77 | 0.05 | 0.17 | 0.00018202 | 0.00043669 |
| NSUN3 | IGF2BP3 | 3.77 | 0.05 | 0.17 | 0.00018462 | 0.00044269 |
| TRMT10C | NSUN2 | 3.77 | 0.05 | 0.17 | 0.00018569 | 0.00044506 |
| PUS1 | TRMT112 | 3.76 | 0.05 | 0.17 | 0.00019063 | 0.00045646 |
| HENMT1 | NSUN7 | 3.76 | 0.05 | 0.17 | 0.0001909 | 0.00045688 |
| FTSJ3 | TET2 | 3.76 | 0.05 | 0.17 | 0.00019275 | 0.00046111 |
| TRMT10C | IGF2BP3 | 3.76 | 0.05 | 0.17 | 0.00019486 | 0.00046592 |
| PUS10 | ALKBH3 | 3.75 | 0.05 | 0.17 | 0.0001985 | 0.00047419 |
| G3BP1 | IGF2BP3 | 3.75 | 0.05 | 0.17 | 0.00019951 | 0.00047637 |
| ELAVL1 | IGF2BP1 | 3.74 | 0.05 | 0.17 | 0.00020409 | 0.00048707 |
| TET2 | NSUN6 | 3.74 | 0.05 | 0.17 | 0.0002086 | 0.00049738 |
| CTU1 | BUD23 | 3.74 | 0.05 | 0.17 | 0.00020892 | 0.00049789 |
| G3BP1 | RBM15B | 3.73 | 0.05 | 0.17 | 0.00021369 | 0.00050854 |
| RPUSD4 | KIAA1429 | 3.73 | 0.05 | 0.17 | 0.00021475 | 0.00051082 |
| TGS1 | YBX1 | 3.73 | 0.05 | 0.17 | 0.00021514 | 0.00051151 |
| PRRC2A | IGF2BP3 | 3.73 | 0.05 | 0.17 | 0.00021538 | 0.00051185 |
| TRMT11 | NSUN7 | 3.73 | 0.05 | 0.17 | 0.00021577 | 0.00051253 |
| ALKBH1 | LRPPRC | 3.72 | 0.05 | 0.17 | 0.00022485 | 0.0005336 |
| MRM3 | NOP2 | 3.71 | 0.05 | 0.17 | 0.00022808 | 0.00054075 |
| RPUSD2 | WDR4 | 3.71 | 0.05 | 0.17 | 0.00022922 | 0.00054302 |
| DNMT3B | NSUN7 | 3.71 | 0.05 | 0.17 | 0.00023318 | 0.00055207 |
| TRMT61B | RBM15 | 3.71 | 0.05 | 0.17 | 0.00023408 | 0.00055393 |
| RPUSD3 | TRMT10C | 3.70 | 0.05 | 0.17 | 0.00023685 | 0.00056024 |
| WDR4 | CBLL1 | 3.70 | 0.05 | 0.17 | 0.00023754 | 0.0005616 |
| YTHDF2 | ZCCHC4 | 3.70 | 0.05 | 0.17 | 0.00024106 | 0.0005694 |
| NSUN3 | FMR1 | 3.70 | 0.05 | 0.17 | 0.00024174 | 0.00057072 |
| HENMT1 | FMR1 | 3.70 | 0.05 | 0.17 | 0.00024407 | 0.00057568 |
| DKC1 | MRM3 | 3.70 | 0.05 | 0.17 | 0.00024541 | 0.00057831 |
| DNMT3A | ZCCHC4 | 3.70 | 0.05 | 0.17 | 0.00024553 | 0.00057833 |
| YBX1 | ELAVL1 | 3.70 | 0.05 | 0.17 | 0.00024579 | 0.00057866 |
| METTL1 | METTL3 | 3.69 | 0.05 | 0.17 | 0.00024679 | 0.00058075 |
| FTSJ3 | METTL16 | 3.69 | 0.05 | 0.17 | 0.00024768 | 0.00058258 |
| ELP1 | ELAVL1 | 3.69 | 0.05 | 0.17 | 0.00025268 | 0.00059406 |
| ADARB2 | DNMT3A | 3.69 | 0.05 | 0.17 | 0.00025324 | 0.00059509 |
| TRMT6 | ZCCHC4 | 3.69 | 0.05 | 0.17 | 0.00025456 | 0.00059792 |
| IGF2BP2 | ZC3H13 | 3.68 | 0.05 | 0.17 | 0.0002577 | 0.00060501 |
| RPUSD2 | ALYREF | 3.68 | 0.05 | 0.17 | 0.00025821 | 0.00060588 |
| RNMT | TARBP1 | 3.68 | 0.05 | 0.17 | 0.00025831 | 0.00060588 |
| FMR1 | IGF2BP2 | 3.68 | 0.05 | 0.17 | 0.00026022 | 0.00060966 |
| NKAP | YTHDF1 | 3.68 | 0.05 | 0.17 | 0.00026028 | 0.00060966 |
| TRMT112 | ELAVL1 | 3.68 | 0.05 | 0.17 | 0.00026082 | 0.00061036 |
| CTU1 | HNRNPA2B1 | 3.68 | 0.05 | 0.17 | 0.00026388 | 0.00061695 |
| KIAA1429 | WTAP | 3.67 | 0.05 | 0.17 | 0.00026834 | 0.0006265 |
| HNRNPA1 | KIAA1429 | 3.67 | 0.05 | 0.17 | 0.00027053 | 0.00063132 |
| MRM1 | CMTR1 | 3.67 | 0.05 | 0.17 | 0.00027081 | 0.00063168 |
| NOP2 | IGF2BP2 | 3.67 | 0.05 | 0.17 | 0.00027334 | 0.00063729 |
| TRMT61B | DNMT1 | 3.66 | 0.05 | 0.17 | 0.00027642 | 0.00064417 |
| TGS1 | MRM2 | 3.66 | 0.05 | 0.17 | 0.00027785 | 0.0006469 |
| NSUN6 | LRPPRC | 3.65 | 0.05 | 0.17 | 0.00028818 | 0.00067033 |
| DNMT3B | EIF3A | 3.65 | 0.05 | 0.17 | 0.00029036 | 0.00067509 |
| CTU1 | MRM3 | 3.65 | 0.05 | 0.17 | 0.00029081 | 0.00067584 |
| NSUN3 | WTAP | 3.65 | 0.05 | 0.17 | 0.00029248 | 0.0006794 |
| TRMT11 | METTL5 | 3.65 | 0.05 | 0.17 | 0.00029495 | 0.00068483 |
| FBL | WTAP | 3.64 | 0.05 | 0.17 | 0.00029945 | 0.00069463 |
| ADAT2 | RPUSD3 | 3.64 | 0.05 | 0.17 | 0.00030145 | 0.00069896 |
| ADAT3 | WDR4 | 3.64 | 0.05 | 0.17 | 0.00030197 | 0.00069984 |
| TRMT11 | IGF2BP3 | 3.64 | 0.05 | 0.17 | 0.00030591 | 0.00070865 |
| MRM2 | NSUN4 | 3.64 | 0.05 | 0.17 | 0.0003079 | 0.0007126 |
| RPUSD4 | CMTR2 | 3.63 | 0.05 | 0.17 | 0.00031184 | 0.00072107 |
| CTU1 | METTL3 | 3.63 | 0.05 | 0.17 | 0.00031208 | 0.00072128 |
| ADARB2 | ADAT2 | 3.63 | 0.05 | 0.17 | 0.00031396 | 0.0007253 |
| ALKBH1 | YTHDF1 | 3.63 | 0.05 | 0.17 | 0.00031501 | 0.0007274 |
| ADAT2 | NSUN5 | 3.63 | 0.05 | 0.17 | 0.00031578 | 0.00072883 |
| CTU1 | NSUN2 | 3.62 | 0.05 | 0.16 | 0.00032477 | 0.00074856 |
| YTHDF1 | METTL3 | 3.62 | 0.05 | 0.16 | 0.00032692 | 0.00075316 |
| ZCCHC4 | METTL3 | 3.62 | 0.05 | 0.16 | 0.00032913 | 0.00075791 |
| TRMT112 | TRMT6 | 3.62 | 0.05 | 0.16 | 0.00032982 | 0.00075881 |
| NSUN6 | NSUN3 | 3.61 | 0.05 | 0.16 | 0.00033548 | 0.00077148 |
| ALYREF | RBM15B | 3.61 | 0.05 | 0.16 | 0.00033893 | 0.00077895 |
| RPUSD4 | TARBP1 | 3.61 | 0.05 | 0.16 | 0.00033904 | 0.00077895 |
| RPUSD2 | PUS3 | 3.61 | 0.05 | 0.16 | 0.00034304 | 0.00078708 |
| FTSJ1 | IGF2BP2 | 3.61 | 0.05 | 0.16 | 0.00034519 | 0.00079164 |
| YTHDF2 | CBLL1 | 3.60 | 0.05 | 0.16 | 0.00035192 | 0.00080648 |
| TRMT11 | ALKBH1 | 3.60 | 0.05 | 0.16 | 0.00035198 | 0.00080648 |
| TRMT44 | TRMT61A | 3.60 | 0.05 | 0.16 | 0.0003574 | 0.00081854 |
| TET1 | IGF2BP2 | 3.59 | 0.05 | 0.16 | 0.00036291 | 0.00083078 |
| RPUSD2 | TRMT6 | 3.59 | 0.05 | 0.16 | 0.00036484 | 0.00083481 |
| ALKBH1 | NKAP | 3.59 | 0.05 | 0.16 | 0.00037152 | 0.00084896 |
| MRM2 | MRM1 | 3.59 | 0.05 | 0.16 | 0.00037182 | 0.00084926 |
| CTU2 | NOP2 | 3.58 | 0.05 | 0.16 | 0.00037452 | 0.00085504 |
| SNORD48 | TRMT61A | 3.58 | 0.05 | 0.16 | 0.00037665 | 0.0008595 |
| RPUSD1 | DNMT3B | 3.58 | 0.05 | 0.16 | 0.00038075 | 0.00086846 |
| ADAR | WDR4 | 3.58 | 0.05 | 0.16 | 0.00038159 | 0.00086998 |
| RPUSD2 | NOP2 | 3.58 | 0.05 | 0.16 | 0.00038417 | 0.00087548 |
| NSUN5 | NSUN7 | 3.57 | 0.05 | 0.16 | 0.00038758 | 0.00088285 |
| LRPPRC | YTHDF2 | 3.57 | 0.05 | 0.16 | 0.00038979 | 0.00088748 |
| RBMX | ZC3H13 | 3.57 | 0.05 | 0.16 | 0.00039505 | 0.00089906 |
| ADARB2 | PRRC2A | 3.57 | 0.05 | 0.16 | 0.00039634 | 0.00090158 |
| TRMT11 | YTHDC1 | 3.57 | 0.05 | 0.16 | 0.00039993 | 0.00090935 |
| YTHDF3 | ZC3H13 | 3.57 | 0.05 | 0.16 | 0.00040046 | 0.00091013 |
| METTL1 | TRMT61B | 3.57 | 0.05 | 0.16 | 0.00040112 | 0.00091124 |
| PUS10 | TRMT44 | 3.56 | 0.05 | 0.16 | 0.00040745 | 0.00092401 |
| CMTR2 | TRMT44 | 3.56 | 0.05 | 0.16 | 0.00040748 | 0.00092401 |
| MRM2 | HENMT1 | 3.56 | 0.05 | 0.16 | 0.00041358 | 0.00093741 |
| CMTR2 | WTAP | 3.56 | 0.05 | 0.16 | 0.00041524 | 0.00094076 |
| NSUN7 | METTL16 | 3.55 | 0.05 | 0.16 | 0.00041764 | 0.00094578 |
| PUS7 | ZCCHC4 | 3.55 | 0.05 | 0.16 | 0.0004201 | 0.00095091 |
| ALKBH1 | DNMT3B | 3.55 | 0.05 | 0.16 | 0.00042225 | 0.00095535 |
| TRMT6 | YTHDF2 | 3.55 | 0.05 | 0.16 | 0.00042246 | 0.00095541 |
| ADARB2 | FMR1 | 3.55 | 0.05 | 0.16 | 0.00042628 | 0.00096361 |
| RPUSD1 | PUS7 | 3.55 | 0.05 | 0.16 | 0.00042874 | 0.00096875 |
| TRMT10C | NSUN4 | 3.54 | 0.05 | 0.16 | 0.00043211 | 0.00097592 |
| ALKBH8 | PUS10 | 3.54 | 0.05 | 0.16 | 0.00043286 | 0.00097678 |
| SNORD48 | DNMT1 | 3.54 | 0.05 | 0.16 | 0.00043288 | 0.00097678 |
| ADAR | TRMT10B | 3.54 | 0.05 | 0.16 | 0.00043627 | 0.00098399 |
| CMTR1 | FTO | 3.54 | 0.05 | 0.16 | 0.00043845 | 0.00098848 |
| RBM15 | METTL16 | 3.54 | 0.05 | 0.16 | 0.00043892 | 0.00098909 |
| TRUB1 | IGF2BP1 | 3.54 | 0.05 | 0.16 | 0.00044397 | 0.00100003 |
| NSUN4 | FMR1 | 3.54 | 0.05 | 0.16 | 0.00044781 | 0.00100823 |
| CTU1 | TRMT112 | 3.53 | 0.05 | 0.16 | 0.0004486 | 0.00100954 |
| RPUSD4 | METTL5 | 3.53 | 0.05 | 0.16 | 0.00045459 | 0.00102258 |
| TRMT44 | RBM15B | 3.53 | 0.05 | 0.16 | 0.00045768 | 0.00102895 |
| CMTR2 | RRP8 | 3.53 | 0.05 | 0.16 | 0.00045944 | 0.00103165 |
| CMTR2 | RBMX | 3.52 | 0.05 | 0.16 | 0.00047337 | 0.00106104 |
| ADARB2 | TRMT44 | 3.52 | 0.05 | 0.16 | 0.00047392 | 0.0010618 |
| CTU2 | HNRNPC | 3.52 | 0.05 | 0.16 | 0.00047485 | 0.00106342 |
| RPUSD4 | YBX1 | 3.52 | 0.05 | 0.16 | 0.00048062 | 0.00107587 |
| PUS7 | ALKBH1 | 3.52 | 0.05 | 0.16 | 0.00048088 | 0.00107597 |
| HENMT1 | LRPPRC | 3.52 | 0.05 | 0.16 | 0.00048117 | 0.00107613 |
| NSUN4 | NSUN3 | 3.52 | 0.05 | 0.16 | 0.00048209 | 0.00107772 |
| G3BP2 | YTHDC2 | 3.51 | 0.05 | 0.16 | 0.0004852 | 0.0010837 |
| CTU2 | NSUN2 | 3.51 | 0.05 | 0.16 | 0.00048843 | 0.00108997 |
| FTO | KIAA1429 | 3.51 | 0.05 | 0.16 | 0.00048892 | 0.00109058 |
| ADAT2 | RPUSD4 | 3.51 | 0.05 | 0.16 | 0.00048959 | 0.00109159 |
| CTU1 | RBM15B | 3.51 | 0.05 | 0.16 | 0.00049005 | 0.00109212 |
| RPUSD4 | HNRNPC | 3.51 | 0.05 | 0.16 | 0.00049464 | 0.00110186 |
| ADAT2 | ZCCHC4 | 3.51 | 0.05 | 0.16 | 0.00049584 | 0.00110406 |
| DKC1 | WTAP | 3.50 | 0.05 | 0.16 | 0.00050453 | 0.00112291 |
| CMTR2 | KIAA1429 | 3.50 | 0.05 | 0.16 | 0.00050654 | 0.00112678 |
| TET1 | HNRNPC | 3.50 | 0.05 | 0.16 | 0.00050672 | 0.00112678 |
| FBL | DNMT3A | 3.50 | 0.05 | 0.16 | 0.00051115 | 0.00113612 |
| KIAA1429 | METTL3 | 3.50 | 0.05 | 0.16 | 0.00051137 | 0.00113612 |
| YBX1 | NOP2 | 3.50 | 0.05 | 0.16 | 0.00051197 | 0.00113645 |
| NSUN6 | ELAVL1 | 3.50 | 0.05 | 0.16 | 0.00051329 | 0.0011389 |
| PUS7 | TARBP1 | 3.49 | 0.05 | 0.16 | 0.00051914 | 0.00115136 |
| ELP1 | FTO | 3.49 | 0.05 | 0.16 | 0.00052473 | 0.00116325 |
| TRMT11 | TRMT10C | 3.49 | 0.05 | 0.16 | 0.00052768 | 0.00116927 |
| PUS3 | METTL16 | 3.49 | 0.05 | 0.16 | 0.00053032 | 0.0011746 |
| TRMT10B | ZC3H13 | 3.49 | 0.05 | 0.16 | 0.00053315 | 0.00118036 |
| ELP3 | ZC3H13 | 3.48 | 0.05 | 0.16 | 0.00054265 | 0.00120088 |
| PUS7L | METTL1 | 3.48 | 0.05 | 0.16 | 0.00055472 | 0.00122651 |
| TET2 | ZCCHC4 | 3.47 | 0.05 | 0.16 | 0.00056291 | 0.00124353 |
| ALKBH8 | LRPPRC | 3.47 | 0.05 | 0.16 | 0.00056741 | 0.00125236 |
| NSUN7 | NKAP | 3.47 | 0.05 | 0.16 | 0.00057099 | 0.00125971 |
| RPUSD4 | METTL14 | 3.47 | 0.05 | 0.16 | 0.00057518 | 0.0012684 |
| HNRNPC | RBM15 | 3.47 | 0.05 | 0.16 | 0.00057682 | 0.00127147 |
| CMTR2 | TRMT10B | 3.47 | 0.05 | 0.16 | 0.00057823 | 0.00127402 |
| CTU2 | TRMT6 | 3.46 | 0.05 | 0.16 | 0.0005801 | 0.00127759 |
| EIF3A | IGF2BP2 | 3.46 | 0.05 | 0.16 | 0.00058309 | 0.00128361 |
| RNMT | NSUN4 | 3.46 | 0.05 | 0.16 | 0.0005879 | 0.00129364 |
| TRDMT1 | FTO | 3.46 | 0.05 | 0.16 | 0.00059244 | 0.00130306 |
| ADAR | ZCCHC4 | 3.46 | 0.05 | 0.16 | 0.00059329 | 0.00130435 |
| METTL1 | ALKBH1 | 3.46 | 0.05 | 0.16 | 0.00059464 | 0.00130676 |
| FTSJ3 | NKAP | 3.45 | 0.05 | 0.16 | 0.00060177 | 0.00132186 |
| ADAR | NSUN4 | 3.45 | 0.05 | 0.16 | 0.00060229 | 0.00132242 |
| PUS3 | NSUN4 | 3.45 | 0.05 | 0.16 | 0.00060902 | 0.00133654 |
| RNMT | IGF2BP1 | 3.45 | 0.05 | 0.16 | 0.00060925 | 0.00133654 |
| CTU1 | PUS7 | 3.45 | 0.05 | 0.16 | 0.00061109 | 0.00134 |
| PUS7L | TRMT10B | 3.45 | 0.05 | 0.16 | 0.00061145 | 0.00134009 |
| PUS3 | YTHDF2 | 3.45 | 0.05 | 0.16 | 0.00061166 | 0.00134009 |
| YTHDF2 | METTL16 | 3.45 | 0.05 | 0.16 | 0.0006179 | 0.00135316 |
| ELAVL1 | METTL5 | 3.45 | 0.05 | 0.16 | 0.00061879 | 0.00135394 |
| DKC1 | NSUN6 | 3.45 | 0.05 | 0.16 | 0.00061914 | 0.00135411 |
| TRUB1 | METTL14 | 3.44 | 0.05 | 0.16 | 0.00063434 | 0.00138676 |
| SNORD48 | TRMT61B | 3.43 | 0.05 | 0.16 | 0.00065886 | 0.00143974 |
| ELP3 | G3BP1 | 3.42 | 0.05 | 0.16 | 0.00067228 | 0.00146844 |
| NSUN7 | NSUN2 | 3.42 | 0.05 | 0.16 | 0.00067446 | 0.00147256 |
| TRMT6 | NSUN5 | 3.42 | 0.05 | 0.16 | 0.0006764 | 0.00147615 |
| ADARB2 | RNMT | 3.42 | 0.05 | 0.16 | 0.0006789 | 0.00148097 |
| PUS10 | YTHDC1 | 3.42 | 0.05 | 0.16 | 0.00067925 | 0.00148109 |
| PUS3 | METTL1 | 3.42 | 0.05 | 0.16 | 0.00069003 | 0.00150394 |
| RPUSD2 | MRM2 | 3.41 | 0.05 | 0.16 | 0.00069567 | 0.00151494 |
| FBLL1 | HENMT1 | 3.41 | 0.05 | 0.16 | 0.00070958 | 0.0015439 |
| ELP1 | DKC1 | 3.41 | 0.05 | 0.16 | 0.00071093 | 0.00154617 |
| ADARB1 | ADAR | 3.41 | 0.05 | 0.16 | 0.00071297 | 0.00154993 |
| TRDMT1 | NKAP | 3.41 | 0.05 | 0.16 | 0.00071482 | 0.00155262 |
| MRM2 | KIAA1429 | 3.41 | 0.05 | 0.16 | 0.00071565 | 0.00155308 |
| TRMT6 | NSUN3 | 3.40 | 0.05 | 0.16 | 0.00071897 | 0.00155962 |
| CTU1 | NSUN7 | 3.40 | 0.05 | 0.16 | 0.0007218 | 0.00156508 |
| ELP3 | METTL14 | 3.39 | 0.05 | 0.15 | 0.00074796 | 0.00162042 |
| ADARB2 | EIF3A | 3.39 | 0.05 | 0.15 | 0.0007539 | 0.00163189 |
| TRMT44 | RRP8 | 3.39 | 0.05 | 0.15 | 0.00075739 | 0.00163874 |
| HENMT1 | RBMX | 3.39 | 0.05 | 0.15 | 0.00075795 | 0.00163926 |
| TRMT61B | NSUN4 | 3.38 | 0.05 | 0.15 | 0.00077198 | 0.00166889 |
| ELP3 | RRP8 | 3.38 | 0.05 | 0.15 | 0.00078745 | 0.00170159 |
| ADAT2 | TRDMT1 | 3.38 | 0.05 | 0.15 | 0.00079791 | 0.00172345 |
| RPUSD4 | FBL | 3.37 | 0.05 | 0.15 | 0.00080794 | 0.00174355 |
| ALKBH1 | TRDMT1 | 3.37 | 0.05 | 0.15 | 0.00080856 | 0.00174355 |
| TRMT44 | NSUN3 | 3.37 | 0.05 | 0.15 | 0.00080859 | 0.00174355 |
| IGF2BP1 | HNRNPC | 3.37 | 0.05 | 0.15 | 0.00081248 | 0.00175119 |
| PRRC2A | YTHDF2 | 3.37 | 0.05 | 0.15 | 0.00082243 | 0.00177113 |
| CTU1 | NSUN6 | 3.36 | 0.05 | 0.15 | 0.00083382 | 0.00179488 |
| YBX1 | G3BP1 | 3.36 | 0.05 | 0.15 | 0.00084303 | 0.00181316 |
| TET3 | YTHDF2 | 3.36 | 0.05 | 0.15 | 0.00085015 | 0.0018277 |
| MRM1 | FBLL1 | 3.35 | 0.05 | 0.15 | 0.00085899 | 0.00184592 |
| PRRC2A | NKAP | 3.35 | 0.05 | 0.15 | 0.0008597 | 0.00184666 |
| TRMT13 | TRMT10A | 3.35 | 0.05 | 0.15 | 0.00086523 | 0.00185773 |
| ALKBH1 | YTHDC1 | 3.35 | 0.05 | 0.15 | 0.00086576 | 0.00185809 |
| ADAT3 | TRMT10B | 3.35 | 0.05 | 0.15 | 0.00086627 | 0.0018584 |
| TRMT10B | YTHDF1 | 3.35 | 0.05 | 0.15 | 0.00087044 | 0.00186655 |
| TET1 | NKAP | 3.35 | 0.05 | 0.15 | 0.00087208 | 0.00186927 |
| ADAR | TRMT44 | 3.35 | 0.05 | 0.15 | 0.00087333 | 0.00187116 |
| TRMT6 | NKAP | 3.35 | 0.05 | 0.15 | 0.00087453 | 0.00187293 |
| ALKBH5 | YTHDF2 | 3.35 | 0.05 | 0.15 | 0.00088458 | 0.0018933 |
| G3BP2 | IGF2BP3 | 3.35 | 0.05 | 0.15 | 0.00088479 | 0.0018933 |
| ALKBH1 | TRMT6 | 3.34 | 0.05 | 0.15 | 0.00088955 | 0.00190268 |
| NKAP | RBM15 | 3.34 | 0.05 | 0.15 | 0.00090316 | 0.00193096 |
| ADAT3 | RPUSD3 | 3.34 | 0.05 | 0.15 | 0.0009043 | 0.00193259 |
| NSUN2 | YTHDF2 | 3.34 | 0.05 | 0.15 | 0.00090858 | 0.00194093 |
| PUS3 | G3BP1 | 3.34 | 0.05 | 0.15 | 0.00091493 | 0.00195365 |
| YBX1 | HNRNPA1 | 3.34 | 0.05 | 0.15 | 0.00091905 | 0.00196163 |
| TARBP1 | NSUN5 | 3.33 | 0.05 | 0.15 | 0.00093001 | 0.00198396 |
| PUS7L | IGF2BP1 | 3.33 | 0.05 | 0.15 | 0.0009303 | 0.00198396 |
| ADAT3 | HNRNPA2B1 | 3.33 | 0.05 | 0.15 | 0.00094347 | 0.00201119 |
| RPUSD2 | YTHDF2 | 3.33 | 0.05 | 0.15 | 0.00094819 | 0.00201955 |
| EIF3A | IGF2BP3 | 3.33 | 0.05 | 0.15 | 0.00095127 | 0.00202526 |
| IGF2BP2 | WTAP | 3.33 | 0.05 | 0.15 | 0.00095244 | 0.00202689 |
| YBX1 | CBLL1 | 3.32 | 0.05 | 0.15 | 0.00097001 | 0.00206254 |
| ADAT2 | TET2 | 3.32 | 0.05 | 0.15 | 0.00098167 | 0.00208646 |
| BUD23 | TRMT11 | 3.32 | 0.05 | 0.15 | 0.00098479 | 0.00209222 |
| PUS3 | WDR4 | 3.31 | 0.05 | 0.15 | 0.00098926 | 0.00209994 |
| NSUN4 | G3BP2 | 3.31 | 0.05 | 0.15 | 0.00101733 | 0.00215863 |
| TRMT61B | YTHDF1 | 3.31 | 0.05 | 0.15 | 0.00102167 | 0.00216692 |
| ALKBH5 | HNRNPA1 | 3.30 | 0.05 | 0.15 | 0.00103255 | 0.00218816 |
| TGS1 | RBM15B | 3.30 | 0.05 | 0.15 | 0.00103655 | 0.00219572 |
| ALKBH3 | RBMX | 3.30 | 0.05 | 0.15 | 0.00104415 | 0.00221087 |
| HENMT1 | CBLL1 | 3.30 | 0.05 | 0.15 | 0.00105631 | 0.0022357 |
| TRMT11 | NSUN4 | 3.30 | 0.05 | 0.15 | 0.001058 | 0.00223833 |
| EIF3A | YTHDF2 | 3.29 | 0.05 | 0.15 | 0.00106058 | 0.00224285 |
| METTL1 | LRPPRC | 3.29 | 0.05 | 0.15 | 0.00106185 | 0.00224459 |
| DNMT3B | ZCCHC4 | 3.29 | 0.05 | 0.15 | 0.00106268 | 0.0022454 |
| TGS1 | NSUN4 | 3.29 | 0.05 | 0.15 | 0.00106495 | 0.00224927 |
| ELP1 | RPUSD4 | 3.29 | 0.05 | 0.15 | 0.00108088 | 0.00227911 |
| PUS7L | METTL16 | 3.28 | 0.05 | 0.15 | 0.00110117 | 0.00232091 |
| RPUSD2 | ZCCHC4 | 3.28 | 0.05 | 0.15 | 0.00110217 | 0.00232205 |
| TRMT11 | TET1 | 3.28 | 0.05 | 0.15 | 0.00111058 | 0.00233879 |
| DNMT3B | WTAP | 3.28 | 0.05 | 0.15 | 0.00111374 | 0.00234448 |
| ADAT2 | TET1 | 3.28 | 0.05 | 0.15 | 0.00111517 | 0.00234651 |
| DKC1 | RPUSD2 | 3.28 | 0.05 | 0.15 | 0.00112686 | 0.00237011 |
| G3BP1 | IGF2BP2 | 3.28 | 0.05 | 0.15 | 0.00112736 | 0.00237018 |
| ADAR | TARBP1 | 3.28 | 0.05 | 0.15 | 0.00112799 | 0.00237053 |
| TGS1 | TET2 | 3.27 | 0.05 | 0.15 | 0.00113517 | 0.00238461 |
| YTHDC1 | YTHDF3 | 3.27 | 0.05 | 0.15 | 0.00115739 | 0.00242825 |
| HNRNPA1 | CBLL1 | 3.27 | 0.05 | 0.15 | 0.0011583 | 0.00242916 |
| TRMT10A | YTHDC1 | 3.27 | 0.05 | 0.15 | 0.00116049 | 0.00243273 |
| WDR4 | TRMT44 | 3.26 | 0.05 | 0.15 | 0.00119923 | 0.00251186 |
| TGS1 | IGF2BP2 | 3.26 | 0.05 | 0.15 | 0.00120357 | 0.00251887 |
| RPUSD4 | RPUSD3 | 3.26 | 0.05 | 0.15 | 0.00121094 | 0.00253324 |
| DKC1 | SNORD48 | 3.25 | 0.05 | 0.15 | 0.0012281 | 0.00256807 |
| RPUSD1 | RBM15B | 3.25 | 0.05 | 0.15 | 0.00123057 | 0.00257112 |
| FTSJ1 | LRPPRC | 3.25 | 0.05 | 0.15 | 0.00123987 | 0.00258946 |
| CTU1 | ADAT2 | 3.24 | 0.05 | 0.15 | 0.00126925 | 0.00264974 |
| FTSJ3 | NSUN6 | 3.24 | 0.05 | 0.15 | 0.00128026 | 0.00267161 |
| EIF3A | ZCCHC4 | 3.24 | 0.05 | 0.15 | 0.00129624 | 0.00270384 |
| ALKBH8 | RBMX | 3.23 | 0.05 | 0.15 | 0.00131005 | 0.00273153 |
| G3BP2 | WTAP | 3.23 | 0.05 | 0.15 | 0.00131531 | 0.00274132 |
| YTHDF1 | METTL16 | 3.23 | 0.05 | 0.15 | 0.00131583 | 0.00274132 |
| CTU2 | PUS7 | 3.23 | 0.05 | 0.15 | 0.00132125 | 0.00275147 |
| NSUN4 | YTHDC2 | 3.23 | 0.05 | 0.15 | 0.00132371 | 0.00275545 |
| ALKBH1 | METTL5 | 3.23 | 0.05 | 0.15 | 0.00132748 | 0.00276216 |
| G3BP1 | METTL16 | 3.22 | 0.05 | 0.15 | 0.00135535 | 0.00281783 |
| HNRNPA2B1 | YTHDF2 | 3.22 | 0.05 | 0.15 | 0.00136785 | 0.00284147 |
| RPUSD4 | TRMT10A | 3.22 | 0.05 | 0.15 | 0.00138414 | 0.00287415 |
| ELP1 | YTHDF3 | 3.21 | 0.05 | 0.15 | 0.00141147 | 0.00292848 |
| RPUSD4 | RRP8 | 3.21 | 0.05 | 0.15 | 0.00141253 | 0.00292948 |
| ELP1 | ZCCHC4 | 3.21 | 0.05 | 0.15 | 0.00141519 | 0.00293379 |
| PUS7L | METTL3 | 3.21 | 0.05 | 0.15 | 0.00141875 | 0.00293996 |
| WDR4 | TRMT11 | 3.21 | 0.05 | 0.15 | 0.0014213 | 0.00294403 |
| RPUSD2 | TRMT10A | 3.21 | 0.05 | 0.15 | 0.00142295 | 0.00294624 |
| HENMT1 | YTHDF1 | 3.21 | 0.05 | 0.15 | 0.00142392 | 0.00294705 |
| TRMT61B | NKAP | 3.21 | 0.05 | 0.15 | 0.00142828 | 0.00295487 |
| NSUN4 | PRRC2A | 3.21 | 0.05 | 0.15 | 0.00143097 | 0.00295679 |
| LRPPRC | METTL14 | 3.21 | 0.05 | 0.15 | 0.00143376 | 0.00296062 |
| SNORD48 | YTHDC2 | 3.21 | 0.05 | 0.15 | 0.001434 | 0.00296062 |
| DNMT3A | NKAP | 3.21 | 0.05 | 0.15 | 0.00143597 | 0.00296348 |
| RPUSD4 | PUS1 | 3.20 | 0.05 | 0.15 | 0.00144465 | 0.00298017 |
| PUS3 | TRMT61B | 3.20 | 0.05 | 0.15 | 0.0014469 | 0.00298295 |
| TET1 | NSUN4 | 3.20 | 0.05 | 0.15 | 0.00144717 | 0.00298295 |
| RNMT | NSUN7 | 3.20 | 0.05 | 0.15 | 0.00146491 | 0.00301828 |
| MRM3 | LRPPRC | 3.20 | 0.05 | 0.15 | 0.00147403 | 0.00303583 |
| TRDMT1 | CBLL1 | 3.19 | 0.05 | 0.15 | 0.00149288 | 0.00307339 |
| FTSJ3 | ZCCHC4 | 3.19 | 0.05 | 0.15 | 0.00150896 | 0.00310524 |
| TET2 | YTHDF3 | 3.19 | 0.05 | 0.15 | 0.00151143 | 0.00310906 |
| NSUN3 | METTL16 | 3.19 | 0.05 | 0.15 | 0.00151324 | 0.0031115 |
| FTSJ3 | HNRNPA1 | 3.19 | 0.05 | 0.15 | 0.00151406 | 0.00311194 |
| PUS1 | YBX1 | 3.19 | 0.05 | 0.15 | 0.00153317 | 0.00314865 |
| TRMT6 | EIF3A | 3.19 | 0.05 | 0.15 | 0.00154032 | 0.00315946 |
| ALKBH3 | YTHDF2 | 3.18 | 0.05 | 0.15 | 0.0015444 | 0.00316654 |
| FTSJ3 | METTL3 | 3.18 | 0.05 | 0.15 | 0.00155427 | 0.00318549 |
| WDR4 | IGF2BP1 | 3.18 | 0.05 | 0.15 | 0.0015563 | 0.00318836 |
| MRM3 | TRMT61B | 3.18 | 0.05 | 0.15 | 0.00157725 | 0.00322997 |
| TRMT10B | PRRC2A | 3.17 | 0.05 | 0.15 | 0.00159862 | 0.00327108 |
| ALKBH8 | TRUB1 | 3.17 | 0.05 | 0.15 | 0.00160164 | 0.00327593 |
| TARBP1 | RRP8 | 3.17 | 0.05 | 0.14 | 0.00160643 | 0.0032844 |
| TRMT6 | RBM15B | 3.17 | 0.05 | 0.14 | 0.0016098 | 0.00328996 |
| RPUSD1 | DNMT1 | 3.17 | 0.05 | 0.14 | 0.00162912 | 0.00332675 |
| RPUSD4 | HNRNPA2B1 | 3.17 | 0.05 | 0.14 | 0.00162996 | 0.00332713 |
| NSUN6 | METTL14 | 3.17 | 0.05 | 0.14 | 0.00163214 | 0.00333021 |
| ZCCHC4 | RBM15 | 3.17 | 0.05 | 0.14 | 0.00163878 | 0.00334242 |
| TARBP1 | TRMT61B | 3.17 | 0.05 | 0.14 | 0.00164318 | 0.00335004 |
| NKAP | KIAA1429 | 3.16 | 0.05 | 0.14 | 0.0016546 | 0.00337196 |
| HENMT1 | HNRNPC | 3.16 | 0.05 | 0.14 | 0.00167018 | 0.00340235 |
| RPUSD4 | RPUSD2 | 3.16 | 0.05 | 0.14 | 0.00169291 | 0.00344725 |
| TRMT44 | HNRNPA2B1 | 3.16 | 0.05 | 0.14 | 0.00170459 | 0.00346964 |
| TRMT10A | G3BP1 | 3.15 | 0.05 | 0.14 | 0.00171222 | 0.00348375 |
| RPUSD2 | TRMT61B | 3.15 | 0.05 | 0.14 | 0.0017129 | 0.00348375 |
| FTSJ3 | FBL | 3.15 | 0.05 | 0.14 | 0.00171791 | 0.00349253 |
| FBL | CBLL1 | 3.15 | 0.05 | 0.14 | 0.00172176 | 0.00349894 |
| ADAR | FTO | 3.15 | 0.05 | 0.14 | 0.00173343 | 0.00352124 |
| NSUN6 | EIF3A | 3.15 | 0.05 | 0.14 | 0.00173618 | 0.00352429 |
| TRMT61A | NKAP | 3.15 | 0.05 | 0.14 | 0.00173632 | 0.00352429 |
| ELP3 | YTHDF3 | 3.15 | 0.05 | 0.14 | 0.00174421 | 0.00353887 |
| CTU1 | TARBP1 | 3.15 | 0.05 | 0.14 | 0.00174662 | 0.00354235 |
| CMTR2 | ALKBH1 | 3.14 | 0.05 | 0.14 | 0.00177406 | 0.00359654 |
| TGS1 | YTHDF1 | 3.14 | 0.05 | 0.14 | 0.00180821 | 0.00366138 |
| PUS7 | NSUN3 | 3.14 | 0.05 | 0.14 | 0.00180933 | 0.00366217 |
| MRM3 | RRP8 | 3.14 | 0.05 | 0.14 | 0.00182468 | 0.00369176 |
| RPUSD3 | FTSJ1 | 3.14 | 0.05 | 0.14 | 0.00182545 | 0.00369183 |
| ADAR | PUS1 | 3.13 | 0.05 | 0.14 | 0.00184138 | 0.00372108 |
| TRUB1 | HENMT1 | 3.13 | 0.05 | 0.14 | 0.00184561 | 0.00372813 |
| BUD23 | TRMT13 | 3.13 | 0.05 | 0.14 | 0.00187579 | 0.00378607 |
| TET1 | G3BP2 | 3.13 | 0.05 | 0.14 | 0.00187852 | 0.00379006 |
| TRMT112 | ALKBH3 | 3.13 | 0.05 | 0.14 | 0.00188146 | 0.00379447 |
| TRMT11 | METTL14 | 3.13 | 0.05 | 0.14 | 0.00188366 | 0.0037974 |
| NSUN5 | RBM15 | 3.12 | 0.05 | 0.14 | 0.00189733 | 0.00382342 |
| FTSJ1 | DNMT1 | 3.12 | 0.05 | 0.14 | 0.00189875 | 0.00382476 |
| TRMT10A | EIF3A | 3.12 | 0.05 | 0.14 | 0.00191108 | 0.00384654 |
| YBX1 | KIAA1429 | 3.12 | 0.05 | 0.14 | 0.00193718 | 0.00389596 |
| MRM1 | RRP8 | 3.12 | 0.05 | 0.14 | 0.00193866 | 0.00389738 |
| METTL1 | DNMT3A | 3.11 | 0.05 | 0.14 | 0.00195215 | 0.00392294 |
| ELP1 | TRDMT1 | 3.11 | 0.05 | 0.14 | 0.00195589 | 0.0039289 |
| YBX1 | DNMT1 | 3.11 | 0.05 | 0.14 | 0.00197225 | 0.00396019 |
| RNMT | TRMT44 | 3.11 | 0.05 | 0.14 | 0.0019787 | 0.00397155 |
| MRM1 | NSUN7 | 3.11 | 0.05 | 0.14 | 0.00198789 | 0.00398842 |
| NKAP | IGF2BP3 | 3.10 | 0.05 | 0.14 | 0.00204013 | 0.00409161 |
| WDR4 | NKAP | 3.10 | 0.05 | 0.14 | 0.00206093 | 0.00413168 |
| CTU1 | FBLL1 | 3.10 | 0.05 | 0.14 | 0.00207058 | 0.00414775 |
| WDR4 | NSUN6 | 3.10 | 0.05 | 0.14 | 0.00207059 | 0.00414775 |
| ADAT2 | FMR1 | 3.10 | 0.05 | 0.14 | 0.00207712 | 0.00415917 |
| ELP3 | ALKBH1 | 3.09 | 0.05 | 0.14 | 0.00209 | 0.00418331 |
| ADAT3 | FBLL1 | 3.09 | 0.05 | 0.14 | 0.00209922 | 0.0042001 |
| EIF3A | YTHDF1 | 3.09 | 0.05 | 0.14 | 0.00210333 | 0.00420665 |
| NSUN2 | HNRNPA1 | 3.09 | 0.05 | 0.14 | 0.00212098 | 0.00424028 |
| TRMT11 | TET3 | 3.09 | 0.05 | 0.14 | 0.00212763 | 0.00425188 |
| SNORD48 | TET2 | 3.09 | 0.05 | 0.14 | 0.0021418 | 0.00427683 |
| PUS1 | TRMT13 | 3.08 | 0.05 | 0.14 | 0.00216156 | 0.00431458 |
| ADAT2 | PUS10 | 3.08 | 0.05 | 0.14 | 0.00216443 | 0.0043186 |
| MRM2 | YBX1 | 3.08 | 0.05 | 0.14 | 0.00217113 | 0.00433026 |
| ALKBH1 | IGF2BP3 | 3.08 | 0.05 | 0.14 | 0.00218876 | 0.0043637 |
| TRMT11 | NSUN5 | 3.08 | 0.05 | 0.14 | 0.00219482 | 0.00437406 |
| FTSJ3 | RBMX | 3.08 | 0.05 | 0.14 | 0.00219829 | 0.00437923 |
| BUD23 | DNMT3B | 3.08 | 0.05 | 0.14 | 0.00219933 | 0.00437957 |
| METTL1 | ZCCHC4 | 3.08 | 0.05 | 0.14 | 0.00221542 | 0.00440988 |
| PUS3 | TRMT13 | 3.08 | 0.05 | 0.14 | 0.00222179 | 0.00442082 |
| HENMT1 | KIAA1429 | 3.07 | 0.05 | 0.14 | 0.00222846 | 0.00443235 |
| TRMT6 | NSUN4 | 3.07 | 0.05 | 0.14 | 0.00223809 | 0.00444799 |
| HENMT1 | TET1 | 3.07 | 0.05 | 0.14 | 0.00225918 | 0.00448815 |
| NSUN2 | RBMX | 3.07 | 0.05 | 0.14 | 0.00226392 | 0.004495 |
| RBMX | RBM15B | 3.07 | 0.05 | 0.14 | 0.00226441 | 0.004495 |
| ELP1 | HENMT1 | 3.07 | 0.05 | 0.14 | 0.00227052 | 0.00450357 |
| HNRNPC | YTHDF2 | 3.07 | 0.05 | 0.14 | 0.00228001 | 0.00452063 |
| ADARB1 | TRMT44 | 3.07 | 0.05 | 0.14 | 0.00230126 | 0.00456098 |
| TGS1 | METTL5 | 3.06 | 0.05 | 0.14 | 0.00230648 | 0.00456952 |
| TRMT61A | ALKBH5 | 3.06 | 0.05 | 0.14 | 0.00234746 | 0.00464707 |
| TRUB1 | ALYREF | 3.06 | 0.05 | 0.14 | 0.00235428 | 0.00465874 |
| ELP1 | IGF2BP2 | 3.06 | 0.05 | 0.14 | 0.00236491 | 0.00467612 |
| RNMT | TRMT11 | 3.06 | 0.05 | 0.14 | 0.00236858 | 0.00468154 |
| MRM2 | RBM15B | 3.06 | 0.05 | 0.14 | 0.00237751 | 0.00469736 |
| ELP3 | YTHDF2 | 3.05 | 0.05 | 0.14 | 0.00238055 | 0.00470152 |
| HNRNPA1 | METTL16 | 3.05 | 0.05 | 0.14 | 0.00238454 | 0.00470572 |
| NSUN2 | ZCCHC4 | 3.05 | 0.05 | 0.14 | 0.0024046 | 0.00473975 |
| RPUSD2 | RBM15B | 3.05 | 0.05 | 0.14 | 0.00240627 | 0.00474045 |
| CTU2 | NKAP | 3.05 | 0.05 | 0.14 | 0.00240683 | 0.00474045 |
| TGS1 | YTHDF2 | 3.05 | 0.05 | 0.14 | 0.00245213 | 0.0048259 |
| RPUSD2 | ALKBH3 | 3.04 | 0.05 | 0.14 | 0.00247279 | 0.00486466 |
| ADARB2 | DNMT1 | 3.04 | 0.05 | 0.14 | 0.00248225 | 0.00488137 |
| RNMT | CMTR2 | 3.04 | 0.05 | 0.14 | 0.00249234 | 0.00489891 |
| PUS7 | MRM3 | 3.04 | 0.05 | 0.14 | 0.00249311 | 0.00489891 |
| ELP3 | ALKBH3 | 3.04 | 0.05 | 0.14 | 0.00250695 | 0.00492418 |
| RPUSD3 | RBM15B | 3.03 | 0.05 | 0.14 | 0.00257332 | 0.0050483 |
| TRMT10C | ELAVL1 | 3.03 | 0.05 | 0.14 | 0.00257379 | 0.0050483 |
| MRM1 | ELAVL1 | 3.03 | 0.05 | 0.14 | 0.00257413 | 0.0050483 |
| CTU2 | TRMT10C | 3.03 | 0.05 | 0.14 | 0.00257925 | 0.00505638 |
| ADAT2 | WTAP | 3.03 | 0.05 | 0.14 | 0.0025945 | 0.00508429 |
| TRUB1 | YTHDF2 | 3.03 | 0.05 | 0.14 | 0.00260114 | 0.00509534 |
| PUS10 | ZCCHC4 | 3.02 | 0.05 | 0.14 | 0.00268136 | 0.00524839 |
| ALKBH8 | ADAT2 | 3.02 | 0.05 | 0.14 | 0.00269186 | 0.00526691 |
| HNRNPC | HNRNPA2B1 | 3.02 | 0.05 | 0.14 | 0.00270354 | 0.00528481 |
| ADAR | ALYREF | 3.02 | 0.05 | 0.14 | 0.00270415 | 0.00528481 |
| PUS7 | YTHDC1 | 3.01 | 0.05 | 0.14 | 0.00271984 | 0.00531136 |
| RPUSD4 | YTHDF3 | 3.01 | 0.05 | 0.14 | 0.00274872 | 0.00536568 |
| SNORD48 | TRMT44 | 3.01 | 0.05 | 0.14 | 0.00275998 | 0.00538559 |
| ADAR | NSUN6 | 3.01 | 0.05 | 0.14 | 0.00277024 | 0.00540352 |
| IGF2BP3 | YTHDF1 | 3.00 | 0.05 | 0.14 | 0.00280164 | 0.00546055 |
| NSUN7 | METTL14 | 3.00 | 0.05 | 0.14 | 0.00282708 | 0.00550589 |
| WDR4 | IGF2BP2 | 3.00 | 0.05 | 0.14 | 0.002846 | 0.00554059 |
| TGS1 | TRMT11 | 3.00 | 0.05 | 0.14 | 0.00287856 | 0.00560182 |
| LRPPRC | NKAP | 2.99 | 0.05 | 0.14 | 0.00289269 | 0.00562715 |
| HNRNPA2B1 | METTL5 | 2.99 | 0.05 | 0.14 | 0.00291838 | 0.00567276 |
| TRMT61B | NOP2 | 2.99 | 0.05 | 0.14 | 0.00293288 | 0.00569656 |
| PUS1 | CMTR1 | 2.99 | 0.05 | 0.14 | 0.00294104 | 0.00571021 |
| MRM1 | METTL5 | 2.99 | 0.05 | 0.14 | 0.00297369 | 0.00576917 |
| DNMT3A | YTHDF2 | 2.98 | 0.05 | 0.14 | 0.00298777 | 0.00579426 |
| SNORD48 | RBM15B | 2.98 | 0.05 | 0.14 | 0.00300403 | 0.00582354 |
| RPUSD1 | METTL5 | 2.98 | 0.05 | 0.14 | 0.00300832 | 0.00582962 |
| MRM1 | FTSJ1 | 2.98 | 0.05 | 0.14 | 0.00301806 | 0.00584627 |
| TRDMT1 | G3BP1 | 2.98 | 0.05 | 0.14 | 0.00305773 | 0.00591857 |
| PUS1 | TRMT11 | 2.98 | 0.05 | 0.14 | 0.00306202 | 0.00592459 |
| RPUSD1 | YBX1 | 2.98 | 0.05 | 0.14 | 0.00306826 | 0.0059344 |
| METTL1 | HNRNPA1 | 2.98 | 0.05 | 0.14 | 0.00307377 | 0.00594278 |
| TRMT13 | IGF2BP3 | 2.97 | 0.05 | 0.14 | 0.0030814 | 0.00595525 |
| PUS10 | METTL16 | 2.97 | 0.05 | 0.14 | 0.00309955 | 0.00598574 |
| PUS7L | ALKBH1 | 2.97 | 0.05 | 0.14 | 0.00311306 | 0.00600921 |
| TGS1 | FBL | 2.97 | 0.05 | 0.14 | 0.00311408 | 0.00600921 |
| ELP3 | NSUN3 | 2.97 | 0.05 | 0.14 | 0.00313799 | 0.00605121 |
| PUS10 | FTO | 2.97 | 0.05 | 0.14 | 0.00313824 | 0.00605121 |
| ADARB2 | TARBP1 | 2.97 | 0.05 | 0.14 | 0.00314613 | 0.00606179 |
| HENMT1 | NKAP | 2.97 | 0.05 | 0.14 | 0.00315543 | 0.0060774 |
| CTU1 | PRRC2A | 2.97 | 0.05 | 0.14 | 0.00316816 | 0.00609958 |
| ADARB1 | RBM15 | 2.97 | 0.05 | 0.14 | 0.0031695 | 0.00609984 |
| TRMT61B | YBX1 | 2.97 | 0.05 | 0.14 | 0.0031778 | 0.00611348 |
| TRMT61B | YTHDC1 | 2.96 | 0.05 | 0.14 | 0.00319544 | 0.00614507 |
| TRMT61A | METTL5 | 2.96 | 0.05 | 0.14 | 0.00324023 | 0.00622883 |
| TRMT10C | RBM15 | 2.96 | 0.05 | 0.14 | 0.00327415 | 0.00629165 |
| ALKBH8 | TRMT44 | 2.96 | 0.05 | 0.14 | 0.00328163 | 0.00630363 |
| TRMT10C | NSUN5 | 2.95 | 0.05 | 0.14 | 0.00330956 | 0.00635485 |
| NOP2 | KIAA1429 | 2.95 | 0.05 | 0.14 | 0.00332663 | 0.0063852 |
| CMTR2 | ZC3H13 | 2.95 | 0.05 | 0.13 | 0.00333593 | 0.00640063 |
| WDR4 | ZCCHC4 | 2.94 | 0.05 | 0.13 | 0.0033924 | 0.00650403 |
| TRUB1 | FMR1 | 2.94 | 0.05 | 0.13 | 0.00340152 | 0.00651903 |
| PUS7L | ALYREF | 2.94 | 0.05 | 0.13 | 0.00343214 | 0.00657524 |
| CMTR1 | ZCCHC4 | 2.94 | 0.05 | 0.13 | 0.00343562 | 0.0065794 |
| ADARB2 | TET1 | 2.94 | 0.05 | 0.13 | 0.00345257 | 0.00660935 |
| RBM15 | WTAP | 2.94 | 0.05 | 0.13 | 0.00346681 | 0.00663409 |
| ALKBH1 | ALYREF | 2.94 | 0.05 | 0.13 | 0.00347476 | 0.00664679 |
| ALKBH8 | RBM15B | 2.94 | 0.05 | 0.13 | 0.00348176 | 0.00665767 |
| RPUSD2 | NSUN4 | 2.94 | 0.05 | 0.13 | 0.00349056 | 0.00667095 |
| RPUSD4 | TRMT112 | 2.94 | 0.05 | 0.13 | 0.00349135 | 0.00667095 |
| MRM2 | PRRC2A | 2.93 | 0.05 | 0.13 | 0.00351213 | 0.00670812 |
| RPUSD2 | RRP8 | 2.93 | 0.05 | 0.13 | 0.00351745 | 0.00671573 |
| RPUSD4 | SNORD48 | 2.93 | 0.05 | 0.13 | 0.00356172 | 0.00679769 |
| TRMT11 | CMTR2 | 2.93 | 0.05 | 0.13 | 0.00356352 | 0.00679856 |
| CTU2 | YBX1 | 2.93 | 0.05 | 0.13 | 0.0035672 | 0.00680302 |
| RPUSD4 | HENMT1 | 2.93 | 0.05 | 0.13 | 0.0035698 | 0.00680493 |
| TGS1 | IGF2BP1 | 2.93 | 0.05 | 0.13 | 0.0035709 | 0.00680493 |
| PUS7 | CMTR1 | 2.92 | 0.05 | 0.13 | 0.00362686 | 0.00689854 |
| RNMT | METTL3 | 2.92 | 0.05 | 0.13 | 0.00362984 | 0.00690161 |
| KIAA1429 | METTL16 | 2.92 | 0.05 | 0.13 | 0.00364487 | 0.00692642 |
| SNORD48 | NSUN7 | 2.92 | 0.05 | 0.13 | 0.00364563 | 0.00692642 |
| RPUSD4 | G3BP1 | 2.92 | 0.05 | 0.13 | 0.00365814 | 0.00694757 |
| CTU2 | IGF2BP1 | 2.92 | 0.05 | 0.13 | 0.00368513 | 0.00699358 |
| PUS3 | ALYREF | 2.91 | 0.05 | 0.13 | 0.00374125 | 0.00709741 |
| TRMT13 | YTHDF1 | 2.91 | 0.05 | 0.13 | 0.0037675 | 0.00714453 |
| RNMT | ALKBH5 | 2.91 | 0.05 | 0.13 | 0.00380141 | 0.00720342 |
| CTU2 | TRMT61B | 2.91 | 0.05 | 0.13 | 0.00384122 | 0.00727067 |
| PUS3 | NOP2 | 2.90 | 0.05 | 0.13 | 0.00387209 | 0.00732541 |
| NSUN6 | RBM15B | 2.90 | 0.05 | 0.13 | 0.00387304 | 0.00732541 |
| NSUN2 | ZC3H13 | 2.90 | 0.05 | 0.13 | 0.00387967 | 0.0073352 |
| RRP8 | NSUN6 | 2.90 | 0.05 | 0.13 | 0.00388697 | 0.00734626 |
| TET1 | ALYREF | 2.90 | 0.05 | 0.13 | 0.00389508 | 0.00735882 |
| FTSJ3 | SNORD48 | 2.89 | 0.05 | 0.13 | 0.00397111 | 0.00749686 |
| MRM1 | FBL | 2.89 | 0.05 | 0.13 | 0.00398707 | 0.00752418 |
| TRMT13 | TRMT61A | 2.89 | 0.05 | 0.13 | 0.00404151 | 0.00762407 |
| ADARB2 | METTL3 | 2.89 | 0.05 | 0.13 | 0.00404745 | 0.00763242 |
| ADARB2 | FTO | 2.89 | 0.05 | 0.13 | 0.00406943 | 0.007671 |
| CMTR1 | LRPPRC | 2.89 | 0.05 | 0.13 | 0.00408858 | 0.00770422 |
| ADARB2 | ADAR | 2.88 | 0.05 | 0.13 | 0.0040981 | 0.00771929 |
| RPUSD4 | NSUN3 | 2.88 | 0.05 | 0.13 | 0.00417801 | 0.00786393 |
| CMTR1 | NSUN5 | 2.88 | 0.05 | 0.13 | 0.00418852 | 0.00788078 |
| TRMT10C | G3BP2 | 2.88 | 0.05 | 0.13 | 0.00419972 | 0.00789892 |
| SNORD48 | HNRNPA1 | 2.88 | 0.05 | 0.13 | 0.00420407 | 0.00790415 |
| TET3 | G3BP2 | 2.87 | 0.05 | 0.13 | 0.00424794 | 0.00798025 |
| RPUSD4 | TRMT61A | 2.87 | 0.05 | 0.13 | 0.00424929 | 0.00798025 |
| NSUN6 | HNRNPA1 | 2.87 | 0.05 | 0.13 | 0.00425675 | 0.00799129 |
| PUS1 | ALKBH5 | 2.87 | 0.05 | 0.13 | 0.00427632 | 0.00802506 |
| MRM2 | NSUN7 | 2.86 | 0.05 | 0.13 | 0.00436675 | 0.00818868 |
| ALKBH1 | FMR1 | 2.86 | 0.05 | 0.13 | 0.0043854 | 0.00822058 |
| SNORD48 | TRDMT1 | 2.86 | 0.05 | 0.13 | 0.00441713 | 0.008277 |
| MRM1 | TRMT61B | 2.86 | 0.05 | 0.13 | 0.0044375 | 0.00831208 |
| RPUSD3 | ALKBH1 | 2.86 | 0.05 | 0.13 | 0.00445437 | 0.00834059 |
| ALKBH1 | YTHDF2 | 2.86 | 0.05 | 0.13 | 0.00448226 | 0.00838659 |
| TRMT44 | RBMX | 2.85 | 0.05 | 0.13 | 0.00452763 | 0.00846834 |
| MRM1 | LRPPRC | 2.85 | 0.05 | 0.13 | 0.00455415 | 0.0085148 |
| TRUB1 | TRMT11 | 2.85 | 0.05 | 0.13 | 0.00459027 | 0.00857598 |
| TARBP1 | ZCCHC4 | 2.85 | 0.05 | 0.13 | 0.00462778 | 0.00864285 |
| RPUSD3 | ZCCHC4 | 2.84 | 0.05 | 0.13 | 0.00467416 | 0.00872625 |
| MRM1 | YTHDF1 | 2.84 | 0.05 | 0.13 | 0.00471654 | 0.00880212 |
| TRMT11 | TRMT10A | 2.84 | 0.05 | 0.13 | 0.00475737 | 0.00887175 |
| TRMT61A | NSUN7 | 2.84 | 0.05 | 0.13 | 0.00476084 | 0.00887496 |
| BUD23 | TARBP1 | 2.84 | 0.05 | 0.13 | 0.00476586 | 0.00887775 |
| TRMT11 | CBLL1 | 2.83 | 0.05 | 0.13 | 0.00478933 | 0.00891819 |
| FBLL1 | TRMT10B | 2.83 | 0.05 | 0.13 | 0.00480772 | 0.00894912 |
| TET3 | ALKBH5 | 2.83 | 0.05 | 0.13 | 0.00484104 | 0.00900451 |
| RPUSD2 | IGF2BP1 | 2.83 | 0.05 | 0.13 | 0.00491962 | 0.00914057 |
| SNORD48 | FMR1 | 2.82 | 0.05 | 0.13 | 0.00495439 | 0.00920178 |
| FBLL1 | ALYREF | 2.82 | 0.05 | 0.13 | 0.0049677 | 0.00922311 |
| CBLL1 | METTL16 | 2.82 | 0.05 | 0.13 | 0.00499353 | 0.00926768 |
| TRMT112 | MRM1 | 2.82 | 0.05 | 0.13 | 0.00500635 | 0.00928805 |
| TRMT61B | HNRNPA2B1 | 2.81 | 0.05 | 0.13 | 0.00510219 | 0.00945543 |
| MRM2 | RBMX | 2.81 | 0.05 | 0.13 | 0.00512821 | 0.00950017 |
| DKC1 | ZCCHC4 | 2.81 | 0.05 | 0.13 | 0.00513652 | 0.00951207 |
| TRMT10C | HNRNPA1 | 2.81 | 0.05 | 0.13 | 0.0051402 | 0.00951443 |
| RBMX | METTL16 | 2.81 | 0.05 | 0.13 | 0.00514156 | 0.00951443 |
| RPUSD3 | DNMT3A | 2.81 | 0.05 | 0.13 | 0.00523191 | 0.00967807 |
| PUS3 | TET1 | 2.80 | 0.05 | 0.13 | 0.00524399 | 0.00969686 |
| PUS7L | RBM15B | 2.80 | 0.05 | 0.13 | 0.00527218 | 0.00974406 |
| YTHDF2 | ZC3H13 | 2.80 | 0.05 | 0.13 | 0.00527337 | 0.00974406 |
| ALKBH5 | FMR1 | 2.80 | 0.05 | 0.13 | 0.00531802 | 0.00982297 |
| ALKBH5 | EIF3A | 2.80 | 0.05 | 0.13 | 0.00537334 | 0.00992152 |
| DKC1 | YTHDC1 | 2.79 | 0.05 | 0.13 | 0.00544152 | 0.01004374 |
| FTSJ3 | HENMT1 | 2.79 | 0.05 | 0.13 | 0.00550395 | 0.01015156 |
| MRM2 | ALKBH1 | 2.79 | 0.05 | 0.13 | 0.00551715 | 0.01016847 |
| TRMT44 | NSUN5 | 2.79 | 0.05 | 0.13 | 0.00552619 | 0.01018141 |
| ADAT2 | NSUN4 | 2.79 | 0.05 | 0.13 | 0.00552824 | 0.01018148 |
| HNRNPA1 | YTHDF2 | 2.79 | 0.05 | 0.13 | 0.00556398 | 0.01024356 |
| TRMT13 | NSUN5 | 2.79 | 0.05 | 0.13 | 0.00556789 | 0.01024703 |
| TRMT11 | DNMT3A | 2.78 | 0.05 | 0.13 | 0.00558833 | 0.0102809 |
| FTSJ1 | YTHDF1 | 2.78 | 0.05 | 0.13 | 0.00559058 | 0.01028129 |
| RRP8 | METTL16 | 2.78 | 0.05 | 0.13 | 0.00572076 | 0.01051688 |
| IGF2BP1 | WTAP | 2.77 | 0.05 | 0.13 | 0.00575108 | 0.01056876 |
| MRM3 | HNRNPA1 | 2.77 | 0.05 | 0.13 | 0.00576338 | 0.01058752 |
| LRPPRC | IGF2BP2 | 2.77 | 0.05 | 0.13 | 0.00578033 | 0.01061478 |
| TET3 | NKAP | 2.77 | 0.05 | 0.13 | 0.00581967 | 0.01067719 |
| RPUSD2 | YBX1 | 2.77 | 0.05 | 0.13 | 0.00582065 | 0.01067719 |
| PUS7 | G3BP2 | 2.77 | 0.05 | 0.13 | 0.00584543 | 0.01071875 |
| TRMT10A | WTAP | 2.77 | 0.05 | 0.13 | 0.0058566 | 0.01073532 |
| RPUSD4 | MRM3 | 2.77 | 0.05 | 0.13 | 0.005868 | 0.01075233 |
| ADAT2 | PRRC2A | 2.77 | 0.05 | 0.13 | 0.00588807 | 0.01078519 |
| TRMT61B | ELAVL1 | 2.76 | 0.05 | 0.13 | 0.00591875 | 0.01083352 |
| RBMX | IGF2BP3 | 2.76 | 0.05 | 0.13 | 0.00593774 | 0.01086434 |
| RPUSD4 | G3BP2 | 2.76 | 0.05 | 0.13 | 0.00596205 | 0.01090487 |
| CMTR1 | IGF2BP1 | 2.76 | 0.05 | 0.13 | 0.00598397 | 0.01093762 |
| ADARB1 | FMR1 | 2.76 | 0.05 | 0.13 | 0.00598429 | 0.01093762 |
| HENMT1 | NSUN5 | 2.76 | 0.05 | 0.13 | 0.00599979 | 0.01096198 |
| MRM1 | TRMT13 | 2.76 | 0.05 | 0.13 | 0.00600711 | 0.01097139 |
| METTL1 | HNRNPA2B1 | 2.76 | 0.05 | 0.13 | 0.00604376 | 0.01103434 |
| MRM2 | TET3 | 2.76 | 0.05 | 0.13 | 0.00604969 | 0.01104118 |
| RPUSD3 | WDR4 | 2.76 | 0.05 | 0.13 | 0.0060974 | 0.01112424 |
| TRMT6 | YTHDC1 | 2.75 | 0.05 | 0.13 | 0.00610301 | 0.01113045 |
| TRMT10B | TRMT61A | 2.75 | 0.05 | 0.13 | 0.00610731 | 0.01113427 |
| TRUB1 | HNRNPA1 | 2.75 | 0.05 | 0.13 | 0.00614042 | 0.01119059 |
| EIF3A | HNRNPC | 2.75 | 0.05 | 0.13 | 0.00615722 | 0.01121716 |
| ALYREF | ZCCHC4 | 2.75 | 0.05 | 0.13 | 0.0062657 | 0.01140655 |
| ALKBH3 | TRDMT1 | 2.74 | 0.05 | 0.13 | 0.00631561 | 0.01149327 |
| TRMT61A | NSUN6 | 2.74 | 0.05 | 0.13 | 0.006324 | 0.0115044 |
| DKC1 | G3BP2 | 2.74 | 0.05 | 0.13 | 0.00634072 | 0.01153066 |
| SNORD48 | KIAA1429 | 2.74 | 0.05 | 0.13 | 0.00647331 | 0.0117633 |
| ALKBH8 | ALKBH1 | 2.73 | 0.05 | 0.13 | 0.00649992 | 0.0118074 |
| ELP3 | MRM3 | 2.73 | 0.05 | 0.13 | 0.00650888 | 0.01181943 |
| DNMT3A | NSUN3 | 2.72 | 0.05 | 0.12 | 0.00668168 | 0.01212014 |
| ALKBH8 | YTHDF3 | 2.72 | 0.05 | 0.12 | 0.00676771 | 0.01225859 |
| RPUSD4 | ALYREF | 2.72 | 0.05 | 0.12 | 0.00678661 | 0.01228401 |
| YBX1 | NSUN5 | 2.72 | 0.05 | 0.12 | 0.00680811 | 0.01231409 |
| YTHDC2 | CBLL1 | 2.71 | 0.05 | 0.12 | 0.00694019 | 0.01254851 |
| YTHDF2 | KIAA1429 | 2.71 | 0.05 | 0.12 | 0.00694528 | 0.01255322 |
| ELP3 | TET1 | 2.71 | 0.05 | 0.12 | 0.00699162 | 0.01263244 |
| CMTR1 | G3BP1 | 2.71 | 0.05 | 0.12 | 0.00703286 | 0.01270242 |
| SNORD48 | RRP8 | 2.71 | 0.05 | 0.12 | 0.00704099 | 0.01271255 |
| DNMT3A | TRDMT1 | 2.71 | 0.05 | 0.12 | 0.00707347 | 0.01276207 |
| CMTR2 | ALKBH3 | 2.70 | 0.05 | 0.12 | 0.00708421 | 0.01277231 |
| ALKBH8 | TARBP1 | 2.70 | 0.05 | 0.12 | 0.00711682 | 0.01282652 |
| RNMT | WDR4 | 2.70 | 0.05 | 0.12 | 0.00713492 | 0.01285456 |
| TGS1 | METTL1 | 2.70 | 0.05 | 0.12 | 0.00715506 | 0.01288165 |
| PUS3 | IGF2BP3 | 2.70 | 0.05 | 0.12 | 0.00717711 | 0.01291675 |
| CTU2 | YTHDF1 | 2.69 | 0.05 | 0.12 | 0.00731107 | 0.01315315 |
| TRMT11 | G3BP1 | 2.69 | 0.05 | 0.12 | 0.00733562 | 0.01319262 |
| NKAP | YTHDC2 | 2.69 | 0.05 | 0.12 | 0.00739871 | 0.01329661 |
| PUS7 | IGF2BP1 | 2.69 | 0.05 | 0.12 | 0.0074613 | 0.01339957 |
| NOP2 | G3BP1 | 2.69 | 0.05 | 0.12 | 0.00747404 | 0.01341767 |
| ELP3 | YTHDC1 | 2.68 | 0.05 | 0.12 | 0.00752165 | 0.01349835 |
| MRM2 | FBL | 2.68 | 0.05 | 0.12 | 0.00757453 | 0.01358841 |
| RNMT | YTHDF2 | 2.68 | 0.05 | 0.12 | 0.00771605 | 0.01383246 |
| BUD23 | TRMT6 | 2.67 | 0.05 | 0.12 | 0.00779095 | 0.01395682 |
| HNRNPA1 | YTHDC2 | 2.67 | 0.05 | 0.12 | 0.00787688 | 0.01410577 |
| TRMT10B | CBLL1 | 2.67 | 0.05 | 0.12 | 0.00791521 | 0.01416938 |
| RPUSD4 | IGF2BP3 | 2.67 | 0.05 | 0.12 | 0.00793494 | 0.01419967 |
| WDR4 | HENMT1 | 2.67 | 0.05 | 0.12 | 0.00795507 | 0.01423064 |
| FTSJ1 | NKAP | 2.67 | 0.05 | 0.12 | 0.00796313 | 0.01423594 |
| RPUSD4 | RNMT | 2.66 | 0.05 | 0.12 | 0.00797875 | 0.01425785 |
| PUS10 | NSUN6 | 2.66 | 0.05 | 0.12 | 0.00798951 | 0.01427203 |
| SNORD48 | NSUN5 | 2.66 | 0.05 | 0.12 | 0.00799571 | 0.01427806 |
| ALKBH1 | TRMT10B | 2.66 | 0.05 | 0.12 | 0.00801269 | 0.01430331 |
| CTU1 | TET3 | 2.66 | 0.05 | 0.12 | 0.0080552 | 0.01437412 |
| TRUB1 | ZC3H13 | 2.66 | 0.05 | 0.12 | 0.00817587 | 0.01457914 |
| SNORD48 | YTHDF1 | 2.65 | 0.05 | 0.12 | 0.00824444 | 0.01469623 |
| TRMT44 | TRDMT1 | 2.65 | 0.05 | 0.12 | 0.00827546 | 0.01474633 |
| CMTR2 | G3BP1 | 2.65 | 0.05 | 0.12 | 0.0083257 | 0.0148306 |
| METTL1 | TRMT10A | 2.64 | 0.05 | 0.12 | 0.00844678 | 0.01504098 |
| G3BP1 | ZCCHC4 | 2.64 | 0.05 | 0.12 | 0.00847809 | 0.01509142 |
| TRMT11 | KIAA1429 | 2.64 | 0.05 | 0.12 | 0.00848713 | 0.01510219 |
| ADAT2 | PUS7L | 2.64 | 0.05 | 0.12 | 0.00863029 | 0.01535153 |
| PUS10 | NSUN3 | 2.64 | 0.05 | 0.12 | 0.0086672 | 0.01540963 |
| TRMT11 | FTSJ1 | 2.64 | 0.05 | 0.12 | 0.00866906 | 0.01540963 |
| CTU2 | DNMT3A | 2.64 | 0.05 | 0.12 | 0.0086738 | 0.01541262 |
| NKAP | METTL5 | 2.64 | 0.05 | 0.12 | 0.00868257 | 0.01542278 |
| IGF2BP2 | METTL5 | 2.63 | 0.05 | 0.12 | 0.00873806 | 0.01551044 |
| NOP2 | ALKBH5 | 2.63 | 0.05 | 0.12 | 0.00880184 | 0.01561816 |
| ADAT3 | RBM15B | 2.63 | 0.05 | 0.12 | 0.00880768 | 0.01562304 |
| ADAT2 | FBLL1 | 2.63 | 0.05 | 0.12 | 0.00881644 | 0.0156329 |
| NSUN4 | ZC3H13 | 2.63 | 0.05 | 0.12 | 0.00881943 | 0.0156329 |
| ALKBH8 | DNMT3B | 2.63 | 0.05 | 0.12 | 0.00889595 | 0.01576025 |
| PUS7L | IGF2BP2 | 2.63 | 0.05 | 0.12 | 0.00889752 | 0.01576025 |
| ADARB1 | G3BP1 | 2.63 | 0.05 | 0.12 | 0.00891607 | 0.01578757 |
| FTSJ3 | ALKBH5 | 2.62 | 0.05 | 0.12 | 0.00896817 | 0.01585758 |
| TRUB1 | RRP8 | 2.62 | 0.05 | 0.12 | 0.00905294 | 0.01598662 |
| MRM1 | DNMT1 | 2.62 | 0.05 | 0.12 | 0.00912596 | 0.01610839 |
| RPUSD1 | TARBP1 | 2.61 | 0.05 | 0.12 | 0.00929902 | 0.01640813 |
| CMTR1 | ALKBH5 | 2.61 | 0.05 | 0.12 | 0.00930924 | 0.01642043 |
| DNMT1 | ALKBH5 | 2.61 | 0.05 | 0.12 | 0.00931395 | 0.01642299 |
| TRMT10A | RBM15 | 2.61 | 0.05 | 0.12 | 0.0093441 | 0.01647041 |
| ELP3 | ADAR | 2.61 | 0.05 | 0.12 | 0.00945057 | 0.01664645 |
| FBL | TET1 | 2.60 | 0.05 | 0.12 | 0.00950211 | 0.01672556 |
| NSUN3 | HNRNPC | 2.60 | 0.05 | 0.12 | 0.00951444 | 0.01674143 |
| RPUSD1 | FBLL1 | 2.60 | 0.05 | 0.12 | 0.00959708 | 0.01687508 |
| METTL1 | RBMX | 2.60 | 0.05 | 0.12 | 0.00961157 | 0.0168888 |
| YTHDF1 | METTL14 | 2.60 | 0.05 | 0.12 | 0.00969222 | 0.01701276 |
| ELP1 | HNRNPA1 | 2.60 | 0.05 | 0.12 | 0.00970261 | 0.01702509 |
| TET3 | ALYREF | 2.59 | 0.05 | 0.12 | 0.00982396 | 0.01723199 |
| METTL5 | KIAA1429 | 2.59 | 0.05 | 0.12 | 0.00982735 | 0.01723199 |
| TGS1 | NSUN7 | 2.59 | 0.05 | 0.12 | 0.00992497 | 0.01738809 |
| HENMT1 | G3BP2 | 2.59 | 0.05 | 0.12 | 0.00992671 | 0.01738809 |
| TRDMT1 | G3BP2 | 2.59 | 0.05 | 0.12 | 0.0099833 | 0.01748115 |
| TRMT44 | METTL16 | 2.59 | 0.05 | 0.12 | 0.01000879 | 0.01751972 |
| ADAR | IGF2BP2 | 2.58 | 0.05 | 0.12 | 0.01004528 | 0.0175775 |
| NOP2 | CBLL1 | 2.58 | 0.05 | 0.12 | 0.01005535 | 0.01758903 |
| FBL | G3BP1 | 2.58 | 0.05 | 0.12 | 0.01012691 | 0.01770807 |
| RPUSD4 | IGF2BP2 | 2.58 | 0.05 | 0.12 | 0.01016504 | 0.01776267 |
| SNORD48 | ZCCHC4 | 2.58 | 0.05 | 0.12 | 0.01016517 | 0.01776267 |
| HNRNPA1 | YTHDF1 | 2.58 | 0.05 | 0.12 | 0.01025793 | 0.01791236 |
| YBX1 | RBMX | 2.58 | 0.05 | 0.12 | 0.01026841 | 0.01792446 |
| TRMT44 | TET1 | 2.58 | 0.05 | 0.12 | 0.01028211 | 0.01794217 |
| ALKBH5 | NKAP | 2.58 | 0.05 | 0.12 | 0.01029597 | 0.01796015 |
| FBL | PRRC2A | 2.58 | 0.05 | 0.12 | 0.01032882 | 0.01801124 |
| PUS1 | TRMT10A | 2.57 | 0.05 | 0.12 | 0.01039872 | 0.01812062 |
| NSUN7 | ELAVL1 | 2.57 | 0.05 | 0.12 | 0.01050975 | 0.01829515 |
| RNMT | ZCCHC4 | 2.57 | 0.05 | 0.12 | 0.01052586 | 0.01831687 |
| RPUSD4 | ZC3H13 | 2.56 | 0.05 | 0.12 | 0.01081896 | 0.01880749 |
| NSUN6 | ZC3H13 | 2.56 | 0.05 | 0.12 | 0.01085927 | 0.01887106 |
| ADAR | IGF2BP1 | 2.56 | 0.05 | 0.12 | 0.01087461 | 0.01889122 |
| CTU2 | PUS3 | 2.56 | 0.05 | 0.12 | 0.01092008 | 0.01896368 |
| RNMT | SNORD48 | 2.55 | 0.05 | 0.12 | 0.01094062 | 0.01899283 |
| TRMT11 | HNRNPC | 2.55 | 0.05 | 0.12 | 0.0109543 | 0.01901003 |
| METTL1 | WTAP | 2.55 | 0.05 | 0.12 | 0.01103535 | 0.01914411 |
| G3BP1 | YTHDF1 | 2.55 | 0.05 | 0.12 | 0.01106408 | 0.01918737 |
| FTSJ3 | YTHDF2 | 2.55 | 0.05 | 0.12 | 0.01107092 | 0.01919264 |
| TRMT13 | G3BP1 | 2.55 | 0.05 | 0.12 | 0.0111109 | 0.01925534 |
| ELP3 | TRMT10A | 2.55 | 0.05 | 0.12 | 0.01111561 | 0.01925689 |
| ALKBH5 | RBM15 | 2.55 | 0.05 | 0.12 | 0.01115577 | 0.01931984 |
| ALKBH3 | NSUN4 | 2.55 | 0.05 | 0.12 | 0.01120868 | 0.01940481 |
| PUS3 | DNMT3A | 2.55 | 0.05 | 0.12 | 0.01124483 | 0.01946072 |
| ADAT2 | RNMT | 2.54 | 0.05 | 0.12 | 0.01126863 | 0.01948856 |
| FBL | RBM15 | 2.54 | 0.05 | 0.12 | 0.01135446 | 0.01963027 |
| TRMT44 | ZC3H13 | 2.54 | 0.05 | 0.12 | 0.01137513 | 0.01965928 |
| FBLL1 | DNMT3A | 2.54 | 0.05 | 0.12 | 0.01142262 | 0.0197346 |
| MRM1 | TET3 | 2.54 | 0.05 | 0.12 | 0.01151961 | 0.01989536 |
| PUS7 | TRMT112 | 2.54 | 0.05 | 0.12 | 0.01154154 | 0.01992642 |
| PUS3 | RRP8 | 2.53 | 0.05 | 0.12 | 0.01157495 | 0.01997727 |
| ELAVL1 | ZC3H13 | 2.53 | 0.05 | 0.12 | 0.01160754 | 0.02002667 |
| ADARB1 | CMTR2 | 2.53 | 0.05 | 0.12 | 0.01164732 | 0.02008844 |
| MRM2 | FMR1 | 2.53 | 0.05 | 0.12 | 0.01166775 | 0.02011364 |
| YTHDF3 | YTHDF2 | 2.53 | 0.05 | 0.12 | 0.01167288 | 0.02011364 |
| ADAT3 | RPUSD2 | 2.53 | 0.05 | 0.12 | 0.01167388 | 0.02011364 |
| G3BP2 | RBM15 | 2.53 | 0.05 | 0.12 | 0.01172 | 0.02018622 |
| CTU1 | IGF2BP1 | 2.53 | 0.05 | 0.12 | 0.0117746 | 0.02027334 |
| PUS10 | RBMX | 2.53 | 0.05 | 0.12 | 0.01178644 | 0.02028682 |
| RPUSD2 | LRPPRC | 2.53 | 0.05 | 0.12 | 0.01180781 | 0.02031667 |
| CTU1 | TRMT44 | 2.52 | 0.05 | 0.12 | 0.01191269 | 0.02048784 |
| CTU2 | HENMT1 | 2.52 | 0.05 | 0.12 | 0.01197536 | 0.02058393 |
| TRMT44 | ALKBH3 | 2.52 | 0.05 | 0.12 | 0.01201446 | 0.0206441 |
| CTU1 | RBM15 | 2.52 | 0.05 | 0.12 | 0.01202121 | 0.02064867 |
| PUS3 | ELAVL1 | 2.52 | 0.05 | 0.12 | 0.01209522 | 0.02076873 |
| RRP8 | METTL14 | 2.52 | 0.05 | 0.12 | 0.01213595 | 0.02082452 |
| TRMT13 | METTL5 | 2.52 | 0.05 | 0.12 | 0.01214156 | 0.02082706 |
| BUD23 | NKAP | 2.51 | 0.05 | 0.11 | 0.0125749 | 0.02152653 |
| NSUN6 | YTHDF1 | 2.50 | 0.05 | 0.11 | 0.01271742 | 0.02175575 |
| PUS3 | EIF3A | 2.49 | 0.05 | 0.11 | 0.01294854 | 0.02213614 |
| ADAT3 | TET3 | 2.49 | 0.05 | 0.11 | 0.01299286 | 0.02220439 |
| ALKBH3 | NKAP | 2.49 | 0.05 | 0.11 | 0.01311233 | 0.02238583 |
| TRMT13 | YTHDF2 | 2.49 | 0.05 | 0.11 | 0.01313488 | 0.02240917 |
| ADARB1 | TRDMT1 | 2.49 | 0.05 | 0.11 | 0.01316145 | 0.02243933 |
| ELP3 | TRMT10C | 2.49 | 0.05 | 0.11 | 0.01318407 | 0.02246606 |
| CMTR2 | TRMT61B | 2.49 | 0.05 | 0.11 | 0.01318602 | 0.02246606 |
| DNMT1 | YTHDF2 | 2.49 | 0.05 | 0.11 | 0.01330125 | 0.02263947 |
| PUS10 | ALKBH1 | 2.48 | 0.05 | 0.11 | 0.01335784 | 0.02272813 |
| TRMT61B | IGF2BP2 | 2.48 | 0.05 | 0.11 | 0.01342538 | 0.02281999 |
| TRDMT1 | YTHDF2 | 2.48 | 0.05 | 0.11 | 0.01344623 | 0.02284406 |
| CTU1 | HNRNPA1 | 2.48 | 0.05 | 0.11 | 0.01350554 | 0.02293308 |
| TGS1 | METTL16 | 2.48 | 0.05 | 0.11 | 0.01354093 | 0.02298402 |
| ELP3 | TRMT10B | 2.48 | 0.05 | 0.11 | 0.01354464 | 0.02298402 |
| TRDMT1 | NSUN3 | 2.48 | 0.05 | 0.11 | 0.01360586 | 0.02307239 |
| MRM3 | YBX1 | 2.48 | 0.05 | 0.11 | 0.01366507 | 0.02316502 |
| ALKBH8 | FMR1 | 2.47 | 0.05 | 0.11 | 0.01374827 | 0.02329824 |
| EIF3A | IGF2BP1 | 2.47 | 0.05 | 0.11 | 0.01376453 | 0.02331796 |
| ALKBH3 | TRMT10B | 2.47 | 0.05 | 0.11 | 0.01379416 | 0.02336034 |
| YTHDF3 | RBM15 | 2.47 | 0.05 | 0.11 | 0.0138684 | 0.02347818 |
| NSUN4 | YTHDF3 | 2.47 | 0.05 | 0.11 | 0.01394923 | 0.02360315 |
| PUS1 | RBMX | 2.47 | 0.05 | 0.11 | 0.01399644 | 0.02367114 |
| TGS1 | NOP2 | 2.47 | 0.05 | 0.11 | 0.01403086 | 0.0237214 |
| ALKBH1 | METTL16 | 2.46 | 0.05 | 0.11 | 0.01406559 | 0.02377019 |
| ALKBH8 | HNRNPA1 | 2.46 | 0.05 | 0.11 | 0.01406913 | 0.02377019 |
| WDR4 | TRMT10C | 2.46 | 0.05 | 0.11 | 0.01420608 | 0.02399355 |
| NSUN4 | EIF3A | 2.46 | 0.05 | 0.11 | 0.0143145 | 0.0241605 |
| CMTR2 | HENMT1 | 2.46 | 0.05 | 0.11 | 0.01432912 | 0.02417711 |
| PUS7 | HENMT1 | 2.45 | 0.05 | 0.11 | 0.01446367 | 0.02439597 |
| TRMT6 | ZC3H13 | 2.45 | 0.05 | 0.11 | 0.01447223 | 0.02440225 |
| RRP8 | TRMT10A | 2.45 | 0.05 | 0.11 | 0.01452469 | 0.02448253 |
| WDR4 | HNRNPC | 2.45 | 0.05 | 0.11 | 0.01458304 | 0.02457268 |
| YTHDF1 | ZC3H13 | 2.45 | 0.05 | 0.11 | 0.01462675 | 0.02463812 |
| RPUSD3 | HNRNPC | 2.45 | 0.05 | 0.11 | 0.01464765 | 0.02466108 |
| CMTR1 | NOP2 | 2.45 | 0.05 | 0.11 | 0.0147137 | 0.0247598 |
| DNMT1 | IGF2BP1 | 2.45 | 0.05 | 0.11 | 0.01474073 | 0.02479704 |
| ALKBH3 | METTL14 | 2.44 | 0.05 | 0.11 | 0.01489102 | 0.02503317 |
| TRMT61B | TET2 | 2.44 | 0.05 | 0.11 | 0.01505582 | 0.02530179 |
| RPUSD4 | FTSJ1 | 2.44 | 0.05 | 0.11 | 0.01508629 | 0.0253365 |
| TRMT11 | IGF2BP2 | 2.44 | 0.05 | 0.11 | 0.0150865 | 0.0253365 |
| ADAR | MRM2 | 2.44 | 0.05 | 0.11 | 0.01515071 | 0.02543587 |
| HENMT1 | FBL | 2.43 | 0.05 | 0.11 | 0.01526924 | 0.02561783 |
| RPUSD4 | TET2 | 2.43 | 0.05 | 0.11 | 0.0155486 | 0.02606919 |
| BUD23 | YTHDF1 | 2.43 | 0.05 | 0.11 | 0.01559801 | 0.02614336 |
| RPUSD4 | WTAP | 2.42 | 0.05 | 0.11 | 0.01576683 | 0.02641754 |
| FBLL1 | DNMT3B | 2.42 | 0.05 | 0.11 | 0.01590481 | 0.02663106 |
| YBX1 | YTHDF2 | 2.42 | 0.05 | 0.11 | 0.01605524 | 0.02684734 |
| TRMT6 | G3BP2 | 2.42 | 0.05 | 0.11 | 0.01610308 | 0.02691842 |
| NSUN2 | ALKBH5 | 2.42 | 0.05 | 0.11 | 0.01611414 | 0.026928 |
| ZCCHC4 | WTAP | 2.41 | 0.05 | 0.11 | 0.01613352 | 0.02694394 |
| PUS3 | TRMT10A | 2.41 | 0.05 | 0.11 | 0.01613435 | 0.02694394 |
| MRM3 | FTSJ1 | 2.41 | 0.05 | 0.11 | 0.01615858 | 0.02697548 |
| FTSJ3 | IGF2BP1 | 2.41 | 0.05 | 0.11 | 0.0162825 | 0.02716439 |
| ALKBH8 | METTL3 | 2.41 | 0.05 | 0.11 | 0.01629064 | 0.027169 |
| TRMT61B | RBM15B | 2.41 | 0.05 | 0.11 | 0.01642522 | 0.02737536 |
| HENMT1 | ZCCHC4 | 2.41 | 0.05 | 0.11 | 0.01644244 | 0.02739503 |
| FTSJ3 | NSUN7 | 2.41 | 0.05 | 0.11 | 0.01645608 | 0.02740871 |
| TRUB1 | METTL1 | 2.41 | 0.05 | 0.11 | 0.01653822 | 0.02753643 |
| PUS7 | NKAP | 2.40 | 0.05 | 0.11 | 0.01658481 | 0.02760491 |
| ADAT3 | FBL | 2.40 | 0.05 | 0.11 | 0.01669021 | 0.02775291 |
| TRMT10B | ZCCHC4 | 2.40 | 0.05 | 0.11 | 0.01680895 | 0.02793194 |
| PUS1 | IGF2BP2 | 2.40 | 0.05 | 0.11 | 0.01689562 | 0.02806674 |
| NSUN4 | G3BP1 | 2.40 | 0.05 | 0.11 | 0.01691467 | 0.02808914 |
| CMTR1 | METTL16 | 2.40 | 0.05 | 0.11 | 0.01695779 | 0.02814224 |
| PUS1 | TRMT10B | 2.39 | 0.05 | 0.11 | 0.01704665 | 0.02826474 |
| PUS3 | MRM1 | 2.39 | 0.05 | 0.11 | 0.01734357 | 0.02874468 |
| PUS7 | YTHDF2 | 2.39 | 0.05 | 0.11 | 0.0174224 | 0.02886586 |
| G3BP1 | NKAP | 2.38 | 0.05 | 0.11 | 0.0174865 | 0.02896255 |
| ELP3 | RPUSD2 | 2.37 | 0.05 | 0.11 | 0.01796299 | 0.02972251 |
| ADARB2 | RBM15B | 2.37 | 0.05 | 0.11 | 0.018005 | 0.02978226 |
| RRP8 | YTHDF3 | 2.37 | 0.05 | 0.11 | 0.01808237 | 0.02989067 |
| TRMT10C | DNMT3A | 2.37 | 0.05 | 0.11 | 0.01809695 | 0.02990497 |
| ADAT2 | FTSJ3 | 2.37 | 0.05 | 0.11 | 0.01823843 | 0.03012891 |
| WDR4 | FMR1 | 2.37 | 0.05 | 0.11 | 0.01841282 | 0.03040704 |
| TRMT44 | NSUN2 | 2.36 | 0.05 | 0.11 | 0.01852117 | 0.03056599 |
| ELP3 | RBM15B | 2.36 | 0.05 | 0.11 | 0.0185353 | 0.03057932 |
| HENMT1 | NSUN6 | 2.36 | 0.05 | 0.11 | 0.01873608 | 0.03090045 |
| ELP1 | NOP2 | 2.36 | 0.05 | 0.11 | 0.01877912 | 0.03096133 |
| WDR4 | MRM3 | 2.36 | 0.05 | 0.11 | 0.01881052 | 0.03099456 |
| ADAT3 | NSUN2 | 2.36 | 0.05 | 0.11 | 0.0188218 | 0.03099456 |
| CMTR1 | YTHDF2 | 2.36 | 0.05 | 0.11 | 0.01882218 | 0.03099456 |
| CTU1 | METTL5 | 2.36 | 0.05 | 0.11 | 0.01882382 | 0.03099456 |
| TARBP1 | EIF3A | 2.36 | 0.05 | 0.11 | 0.01883257 | 0.03099886 |
| HNRNPA2B1 | METTL16 | 2.36 | 0.05 | 0.11 | 0.01889609 | 0.03108315 |
| TRMT44 | TRMT61B | 2.35 | 0.05 | 0.11 | 0.01915343 | 0.03148594 |
| ALKBH8 | NSUN6 | 2.35 | 0.05 | 0.11 | 0.01923699 | 0.03160466 |
| ALKBH8 | NSUN4 | 2.35 | 0.05 | 0.11 | 0.01935957 | 0.0317731 |
| RPUSD2 | DNMT3B | 2.34 | 0.05 | 0.11 | 0.01948582 | 0.03196992 |
| G3BP1 | IGF2BP1 | 2.34 | 0.05 | 0.11 | 0.01959471 | 0.03211726 |
| PUS1 | HENMT1 | 2.34 | 0.05 | 0.11 | 0.01962252 | 0.03215241 |
| TRMT112 | RRP8 | 2.34 | 0.05 | 0.11 | 0.01966042 | 0.03220405 |
| MRM1 | RBM15 | 2.34 | 0.05 | 0.11 | 0.01977791 | 0.03238601 |
| ADAR | RPUSD4 | 2.34 | 0.05 | 0.11 | 0.01982549 | 0.03244287 |
| ADAT3 | TRMT44 | 2.33 | 0.05 | 0.11 | 0.0201302 | 0.03290952 |
| ELP3 | LRPPRC | 2.33 | 0.05 | 0.11 | 0.02016612 | 0.03295757 |
| METTL1 | HENMT1 | 2.33 | 0.05 | 0.11 | 0.02017864 | 0.03296737 |
| DNMT3B | ALKBH5 | 2.33 | 0.05 | 0.11 | 0.02030371 | 0.03316097 |
| CMTR2 | NSUN4 | 2.33 | 0.05 | 0.11 | 0.02032688 | 0.03318808 |
| IGF2BP1 | YTHDF3 | 2.33 | 0.05 | 0.11 | 0.02037822 | 0.03326116 |
| DNMT1 | G3BP2 | 2.33 | 0.05 | 0.11 | 0.02043026 | 0.03333522 |
| TRDMT1 | LRPPRC | 2.33 | 0.05 | 0.11 | 0.0204368 | 0.03333522 |
| TRMT10A | TRDMT1 | 2.32 | 0.05 | 0.11 | 0.02053657 | 0.03347722 |
| PUS3 | ZCCHC4 | 2.32 | 0.05 | 0.11 | 0.02053711 | 0.03347722 |
| TRMT11 | YTHDF1 | 2.32 | 0.05 | 0.11 | 0.02064542 | 0.03364291 |
| ALKBH8 | HENMT1 | 2.32 | 0.05 | 0.11 | 0.02067003 | 0.03367214 |
| ALKBH3 | ALKBH1 | 2.32 | 0.05 | 0.11 | 0.020682 | 0.03368077 |
| CTU1 | IGF2BP3 | 2.31 | 0.05 | 0.11 | 0.0210997 | 0.03429465 |
| RPUSD4 | YTHDC2 | 2.31 | 0.05 | 0.11 | 0.02122478 | 0.03448686 |
| TET3 | YTHDF3 | 2.31 | 0.05 | 0.11 | 0.02141638 | 0.03476462 |
| TARBP1 | METTL14 | 2.31 | 0.05 | 0.11 | 0.02143199 | 0.03477878 |
| NKAP | ZCCHC4 | 2.31 | 0.05 | 0.11 | 0.02147759 | 0.03484158 |
| TET1 | ALKBH5 | 2.31 | 0.05 | 0.11 | 0.02152457 | 0.03490657 |
| TRMT10C | RBM15B | 2.31 | 0.05 | 0.11 | 0.02155308 | 0.03494158 |
| MRM2 | ALKBH3 | 2.31 | 0.05 | 0.11 | 0.02157134 | 0.03495996 |
| NOP2 | YTHDC1 | 2.31 | 0.05 | 0.11 | 0.02159233 | 0.03498276 |
| NSUN3 | IGF2BP2 | 2.30 | 0.05 | 0.11 | 0.02184157 | 0.03536505 |
| ALKBH8 | TRMT11 | 2.30 | 0.05 | 0.11 | 0.02213108 | 0.03580967 |
| RPUSD1 | IGF2BP1 | 2.29 | 0.05 | 0.11 | 0.02220625 | 0.03591978 |
| RNMT | NOP2 | 2.29 | 0.05 | 0.11 | 0.02253412 | 0.03643846 |
| FBL | ALKBH1 | 2.29 | 0.05 | 0.11 | 0.02263974 | 0.03659753 |
| RPUSD1 | DNMT3A | 2.29 | 0.05 | 0.10 | 0.02274489 | 0.03675574 |
| TET1 | FTO | 2.28 | 0.05 | 0.10 | 0.02293026 | 0.03703159 |
| NSUN4 | NSUN6 | 2.28 | 0.05 | 0.10 | 0.02300289 | 0.03713703 |
| RPUSD1 | PRRC2A | 2.28 | 0.05 | 0.10 | 0.02316381 | 0.03738486 |
| METTL1 | FTSJ3 | 2.28 | 0.05 | 0.10 | 0.02318355 | 0.03740477 |
| TRMT44 | EIF3A | 2.28 | 0.05 | 0.10 | 0.0232645 | 0.03752339 |
| FBLL1 | NSUN2 | 2.28 | 0.05 | 0.10 | 0.02331767 | 0.03759714 |
| BUD23 | SNORD48 | 2.27 | 0.05 | 0.10 | 0.02343041 | 0.0377666 |
| ALKBH1 | G3BP2 | 2.27 | 0.05 | 0.10 | 0.02343773 | 0.0377666 |
| NSUN3 | METTL5 | 2.27 | 0.05 | 0.10 | 0.02345 | 0.03777432 |
| TRMT112 | HENMT1 | 2.27 | 0.05 | 0.10 | 0.0234698 | 0.03779417 |
| FBLL1 | IGF2BP1 | 2.27 | 0.05 | 0.10 | 0.02347828 | 0.03779577 |
| FTSJ1 | DNMT3A | 2.27 | 0.05 | 0.10 | 0.02354823 | 0.0378963 |
| PUS3 | YTHDF1 | 2.27 | 0.05 | 0.10 | 0.023592 | 0.03794255 |
| RPUSD2 | RBMX | 2.27 | 0.05 | 0.10 | 0.0236133 | 0.03796471 |
| PUS3 | FTSJ1 | 2.27 | 0.05 | 0.10 | 0.02370817 | 0.03810511 |
| NSUN4 | HNRNPA2B1 | 2.27 | 0.05 | 0.10 | 0.02373429 | 0.03813495 |
| TRMT112 | TRMT10A | 2.26 | 0.05 | 0.10 | 0.02410393 | 0.03869194 |
| NKAP | YTHDF2 | 2.26 | 0.05 | 0.10 | 0.02411509 | 0.03869756 |
| ADAT3 | BUD23 | 2.26 | 0.05 | 0.10 | 0.02417442 | 0.03878044 |
| FTSJ3 | YTHDC2 | 2.26 | 0.05 | 0.10 | 0.02424382 | 0.03887943 |
| WDR4 | TRMT10B | 2.26 | 0.05 | 0.10 | 0.02433415 | 0.03901189 |
| ALKBH1 | DNMT3A | 2.26 | 0.05 | 0.10 | 0.02434746 | 0.03902084 |
| TARBP1 | METTL16 | 2.26 | 0.05 | 0.10 | 0.02442699 | 0.03913588 |
| MRM1 | IGF2BP1 | 2.26 | 0.05 | 0.10 | 0.02443535 | 0.03913686 |
| DNMT1 | NSUN3 | 2.26 | 0.05 | 0.10 | 0.02458799 | 0.03935638 |
| TRMT10B | FTO | 2.25 | 0.05 | 0.10 | 0.02462591 | 0.03940457 |
| NSUN7 | PRRC2A | 2.25 | 0.05 | 0.10 | 0.02472854 | 0.03955627 |
| NSUN7 | KIAA1429 | 2.25 | 0.05 | 0.10 | 0.02486381 | 0.03976005 |
| TRMT6 | TRDMT1 | 2.25 | 0.05 | 0.10 | 0.02495953 | 0.03990048 |
| IGF2BP2 | YTHDF1 | 2.25 | 0.05 | 0.10 | 0.02509084 | 0.0400977 |
| SNORD48 | LRPPRC | 2.25 | 0.05 | 0.10 | 0.02519577 | 0.04025265 |
| TGS1 | NSUN6 | 2.24 | 0.05 | 0.10 | 0.02541178 | 0.04058491 |
| FBLL1 | YBX1 | 2.24 | 0.05 | 0.10 | 0.02569772 | 0.04102862 |
| ADARB1 | DNMT3A | 2.24 | 0.05 | 0.10 | 0.02580019 | 0.04117919 |
| RNMT | MRM2 | 2.23 | 0.05 | 0.10 | 0.0260765 | 0.0415939 |
| PUS3 | PUS1 | 2.23 | 0.05 | 0.10 | 0.02612893 | 0.04165123 |
| NSUN7 | LRPPRC | 2.23 | 0.05 | 0.10 | 0.02622461 | 0.04179055 |
| FBLL1 | CMTR1 | 2.23 | 0.05 | 0.10 | 0.02643875 | 0.04210523 |
| ADARB2 | RBM15 | 2.23 | 0.05 | 0.10 | 0.02646292 | 0.04213044 |
| ELP3 | HNRNPA1 | 2.22 | 0.05 | 0.10 | 0.02689763 | 0.04280903 |
| ELP1 | TRMT10A | 2.22 | 0.05 | 0.10 | 0.02702502 | 0.04299822 |
| FMR1 | HNRNPA1 | 2.21 | 0.05 | 0.10 | 0.02725025 | 0.043302 |
| TET2 | YTHDF1 | 2.21 | 0.05 | 0.10 | 0.02736154 | 0.04346517 |
| FBLL1 | IGF2BP2 | 2.21 | 0.05 | 0.10 | 0.02747973 | 0.04362547 |
| FMR1 | ZCCHC4 | 2.21 | 0.05 | 0.10 | 0.02758353 | 0.0437644 |
| ALKBH8 | SNORD48 | 2.21 | 0.05 | 0.10 | 0.02758457 | 0.0437644 |
| IGF2BP1 | YTHDF1 | 2.21 | 0.05 | 0.10 | 0.02774354 | 0.04397517 |
| ADAT3 | RBM15 | 2.21 | 0.05 | 0.10 | 0.02780013 | 0.04405104 |
| PUS10 | TRMT10A | 2.20 | 0.05 | 0.10 | 0.02810813 | 0.0444972 |
| CMTR1 | ALKBH3 | 2.20 | 0.05 | 0.10 | 0.0283485 | 0.04482152 |
| IGF2BP1 | HNRNPA2B1 | 2.20 | 0.05 | 0.10 | 0.02842571 | 0.04492953 |
| ADARB2 | METTL16 | 2.20 | 0.05 | 0.10 | 0.02848348 | 0.04500675 |
| ELP3 | PUS3 | 2.20 | 0.05 | 0.10 | 0.02852789 | 0.04506282 |
| TRMT61B | NSUN5 | 2.20 | 0.05 | 0.10 | 0.02858317 | 0.04513603 |
| TARBP1 | YTHDF2 | 2.20 | 0.05 | 0.10 | 0.02864237 | 0.04521537 |
| ELP3 | ALKBH5 | 2.19 | 0.05 | 0.10 | 0.02879807 | 0.04541857 |
| TRMT44 | DNMT3B | 2.19 | 0.05 | 0.10 | 0.02888023 | 0.04553393 |
| HNRNPA1 | METTL14 | 2.19 | 0.05 | 0.10 | 0.02890576 | 0.04555996 |
| G3BP2 | FMR1 | 2.19 | 0.05 | 0.10 | 0.02899427 | 0.04568519 |
| PUS7L | MRM2 | 2.19 | 0.05 | 0.10 | 0.0291036 | 0.04584316 |
| MRM1 | ALKBH5 | 2.18 | 0.05 | 0.10 | 0.02940837 | 0.04630879 |
| SNORD48 | NSUN4 | 2.18 | 0.05 | 0.10 | 0.02948308 | 0.04641195 |
| NKAP | HNRNPA1 | 2.18 | 0.05 | 0.10 | 0.02951671 | 0.04645042 |
| TET2 | ALKBH5 | 2.18 | 0.05 | 0.10 | 0.02957176 | 0.04652255 |
| RNMT | WTAP | 2.18 | 0.05 | 0.10 | 0.02968456 | 0.04668546 |
| WDR4 | NSUN7 | 2.18 | 0.05 | 0.10 | 0.03004132 | 0.04718776 |
| MRM3 | ELAVL1 | 2.18 | 0.05 | 0.10 | 0.03008161 | 0.04723635 |
| DKC1 | YTHDF2 | 2.17 | 0.05 | 0.10 | 0.03018806 | 0.04738878 |
| FTSJ3 | NSUN4 | 2.17 | 0.05 | 0.10 | 0.03022985 | 0.04743493 |
| ALKBH3 | METTL5 | 2.17 | 0.05 | 0.10 | 0.03033915 | 0.04758158 |
| IGF2BP2 | RBM15 | 2.17 | 0.05 | 0.10 | 0.03042487 | 0.04770121 |
| YTHDC2 | KIAA1429 | 2.17 | 0.05 | 0.10 | 0.03048108 | 0.04777173 |
| PRRC2A | G3BP2 | 2.17 | 0.05 | 0.10 | 0.03054498 | 0.04784496 |
| TRMT10A | NSUN7 | 2.17 | 0.05 | 0.10 | 0.03057871 | 0.04787444 |
| ADAR | ALKBH1 | 2.17 | 0.05 | 0.10 | 0.03058276 | 0.04787444 |
| CTU2 | CMTR2 | 2.17 | 0.05 | 0.10 | 0.03068512 | 0.04801979 |
| PUS1 | EIF3A | 2.17 | 0.05 | 0.10 | 0.03073864 | 0.04807374 |
| NSUN5 | HNRNPC | 2.17 | 0.05 | 0.10 | 0.03086058 | 0.04824952 |
| ALKBH3 | WTAP | 2.16 | 0.05 | 0.10 | 0.03116055 | 0.04869878 |
| ADAR | WTAP | 2.16 | 0.05 | 0.10 | 0.03116722 | 0.04869878 |
| TRMT10A | TRMT6 | 2.16 | 0.05 | 0.10 | 0.03128541 | 0.04886833 |
| FTSJ1 | WTAP | 2.15 | 0.05 | 0.10 | 0.03181303 | 0.04964641 |
| IGF2BP1 | RBM15B | 2.15 | 0.05 | 0.10 | 0.03202933 | 0.04996852 |
| NSUN5 | NSUN3 | -2.15 | 0.05 | -0.10 | 0.03175841 | 0.0495765 |
| ADAT2 | HNRNPC | -2.16 | 0.05 | -0.10 | 0.03134237 | 0.04894217 |
| DKC1 | TET2 | -2.17 | 0.05 | -0.10 | 0.03071724 | 0.04805517 |
| TRMT112 | RBM15B | -2.17 | 0.05 | -0.10 | 0.03048877 | 0.04777173 |
| ADARB1 | RBMX | -2.17 | 0.05 | -0.10 | 0.03023625 | 0.04743493 |
| RPUSD2 | G3BP2 | -2.18 | 0.05 | -0.10 | 0.02985833 | 0.04691492 |
| TET2 | NOP2 | -2.18 | 0.05 | -0.10 | 0.02974496 | 0.04675133 |
| ELP3 | TRMT61A | -2.18 | 0.05 | -0.10 | 0.02969678 | 0.04669014 |
| FTSJ1 | FTO | -2.19 | 0.05 | -0.10 | 0.02870259 | 0.04528213 |
| TRMT44 | METTL5 | -2.19 | 0.05 | -0.10 | 0.028671 | 0.04524643 |
| ADAR | FBLL1 | -2.20 | 0.05 | -0.10 | 0.02831002 | 0.0447747 |
| RPUSD3 | TET3 | -2.20 | 0.05 | -0.10 | 0.02815534 | 0.04454401 |
| RPUSD1 | ZCCHC4 | -2.20 | 0.05 | -0.10 | 0.02812679 | 0.04451279 |
| BUD23 | NSUN3 | -2.20 | 0.05 | -0.10 | 0.02803037 | 0.044388 |
| FBLL1 | METTL14 | -2.20 | 0.05 | -0.10 | 0.02797919 | 0.04432085 |
| RNMT | METTL5 | -2.21 | 0.05 | -0.10 | 0.02771698 | 0.04394686 |
| ADARB2 | ZCCHC4 | -2.21 | 0.05 | -0.10 | 0.0277065 | 0.04394403 |
| MRM1 | YTHDF2 | -2.21 | 0.05 | -0.10 | 0.02742436 | 0.04355126 |
| TARBP1 | IGF2BP3 | -2.22 | 0.05 | -0.10 | 0.02712525 | 0.04311694 |
| PUS3 | TRMT44 | -2.22 | 0.05 | -0.10 | 0.02706059 | 0.04302771 |
| RPUSD2 | TET3 | -2.22 | 0.05 | -0.10 | 0.02705921 | 0.04302771 |
| RPUSD3 | NSUN3 | -2.23 | 0.05 | -0.10 | 0.02625437 | 0.04182478 |
| MRM1 | G3BP1 | -2.23 | 0.05 | -0.10 | 0.0261163 | 0.04164424 |
| HENMT1 | HNRNPA1 | -2.23 | 0.05 | -0.10 | 0.02605725 | 0.04157634 |
| PUS7L | TRMT61A | -2.26 | 0.05 | -0.10 | 0.0245808 | 0.03935638 |
| MRM1 | METTL14 | -2.27 | 0.05 | -0.10 | 0.02389696 | 0.03837191 |
| RPUSD1 | TET3 | -2.27 | 0.05 | -0.10 | 0.02387124 | 0.03834279 |
| TRMT44 | TRMT10C | -2.27 | 0.05 | -0.10 | 0.02358676 | 0.03794255 |
| RRP8 | CBLL1 | -2.28 | 0.05 | -0.10 | 0.02290965 | 0.03701014 |
| RRP8 | DNMT3B | -2.30 | 0.05 | -0.11 | 0.02207758 | 0.03573454 |
| RPUSD2 | METTL14 | -2.30 | 0.05 | -0.11 | 0.0218423 | 0.03536505 |
| FTSJ1 | TRDMT1 | -2.31 | 0.05 | -0.11 | 0.02133382 | 0.03464174 |
| RPUSD3 | DNMT1 | -2.31 | 0.05 | -0.11 | 0.02128256 | 0.03456961 |
| ADAT3 | YBX1 | -2.32 | 0.05 | -0.11 | 0.02102441 | 0.03418328 |
| MRM3 | YTHDC1 | -2.32 | 0.05 | -0.11 | 0.02098835 | 0.03413565 |
| PUS7 | FBLL1 | -2.32 | 0.05 | -0.11 | 0.0209028 | 0.03400745 |
| FBLL1 | ZCCHC4 | -2.32 | 0.05 | -0.11 | 0.02088041 | 0.03398197 |
| RPUSD1 | METTL16 | -2.32 | 0.05 | -0.11 | 0.02084133 | 0.03392932 |
| MRM3 | IGF2BP2 | -2.33 | 0.05 | -0.11 | 0.02011864 | 0.03290128 |
| IGF2BP3 | YTHDC2 | -2.34 | 0.05 | -0.11 | 0.01992625 | 0.03259721 |
| ADARB2 | PUS3 | -2.34 | 0.05 | -0.11 | 0.01980528 | 0.03242031 |
| MRM1 | TRDMT1 | -2.34 | 0.05 | -0.11 | 0.01956672 | 0.0320818 |
| PUS1 | ZC3H13 | -2.34 | 0.05 | -0.11 | 0.0195502 | 0.03206513 |
| FBL | TRDMT1 | -2.35 | 0.05 | -0.11 | 0.01927683 | 0.03164759 |
| ELP3 | BUD23 | -2.35 | 0.05 | -0.11 | 0.01925096 | 0.03161539 |
| CTU2 | ELP3 | -2.35 | 0.05 | -0.11 | 0.01923816 | 0.03160466 |
| ADARB1 | RPUSD1 | -2.35 | 0.05 | -0.11 | 0.01906774 | 0.03135529 |
| TARBP1 | FBL | -2.36 | 0.05 | -0.11 | 0.01887803 | 0.03106356 |
| ADARB1 | PUS3 | -2.36 | 0.05 | -0.11 | 0.01844088 | 0.03044343 |
| CTU2 | YTHDF2 | -2.37 | 0.05 | -0.11 | 0.01803969 | 0.02982988 |
| TRMT10C | ZC3H13 | -2.38 | 0.05 | -0.11 | 0.01768351 | 0.02926966 |
| ADARB2 | NSUN5 | -2.38 | 0.05 | -0.11 | 0.01763431 | 0.02919779 |
| TRMT44 | FBL | -2.39 | 0.05 | -0.11 | 0.01704839 | 0.02826474 |
| ELP3 | NSUN2 | -2.40 | 0.05 | -0.11 | 0.01697701 | 0.02816489 |
| CTU1 | ELP3 | -2.40 | 0.05 | -0.11 | 0.0169372 | 0.02811731 |
| BUD23 | KIAA1429 | -2.40 | 0.05 | -0.11 | 0.01670871 | 0.02777452 |
| PUS10 | TET1 | -2.40 | 0.05 | -0.11 | 0.01667823 | 0.02774212 |
| ALKBH3 | LRPPRC | -2.40 | 0.05 | -0.11 | 0.01663164 | 0.02767373 |
| ELP3 | IGF2BP3 | -2.41 | 0.05 | -0.11 | 0.01631289 | 0.02719712 |
| MRM1 | YBX1 | -2.41 | 0.05 | -0.11 | 0.0162616 | 0.0271385 |
| TRMT112 | FMR1 | -2.42 | 0.05 | -0.11 | 0.01605127 | 0.02684734 |
| FBLL1 | EIF3A | -2.42 | 0.05 | -0.11 | 0.01605095 | 0.02684734 |
| CMTR2 | NSUN5 | -2.42 | 0.05 | -0.11 | 0.01591161 | 0.02663362 |
| TET2 | IGF2BP1 | -2.42 | 0.05 | -0.11 | 0.01579383 | 0.02645401 |
| TRUB1 | BUD23 | -2.43 | 0.05 | -0.11 | 0.01541001 | 0.02584542 |
| PUS10 | FBLL1 | -2.44 | 0.05 | -0.11 | 0.01522675 | 0.02555503 |
| ADARB1 | TRMT6 | -2.45 | 0.05 | -0.11 | 0.01474797 | 0.02480094 |
| CTU2 | TET1 | -2.45 | 0.05 | -0.11 | 0.01465015 | 0.02466108 |
| TRMT44 | IGF2BP1 | -2.46 | 0.05 | -0.11 | 0.01425512 | 0.02406832 |
| ADARB1 | RRP8 | -2.47 | 0.05 | -0.11 | 0.01395157 | 0.02360315 |
| PUS7 | FTO | -2.48 | 0.05 | -0.11 | 0.01356765 | 0.02301533 |
| RPUSD2 | IGF2BP2 | -2.48 | 0.05 | -0.11 | 0.01344859 | 0.02284406 |
| RPUSD1 | KIAA1429 | -2.48 | 0.05 | -0.11 | 0.01342142 | 0.02281999 |
| ADARB2 | RPUSD2 | -2.48 | 0.05 | -0.11 | 0.01340887 | 0.02280727 |
| CTU1 | YTHDF3 | -2.49 | 0.05 | -0.11 | 0.01321835 | 0.02250595 |
| FTSJ3 | CMTR2 | -2.49 | 0.05 | -0.11 | 0.013207 | 0.02249422 |
| TARBP1 | IGF2BP2 | -2.49 | 0.05 | -0.11 | 0.01316015 | 0.02243933 |
| RPUSD3 | CMTR2 | -2.49 | 0.05 | -0.11 | 0.01312175 | 0.02239434 |
| ADARB2 | MRM3 | -2.49 | 0.05 | -0.11 | 0.01307173 | 0.02232405 |
| TRDMT1 | METTL5 | -2.49 | 0.05 | -0.11 | 0.01299946 | 0.02220814 |
| RRP8 | TET3 | -2.50 | 0.05 | -0.11 | 0.01282257 | 0.02192819 |
| TRMT112 | DNMT3A | -2.50 | 0.05 | -0.11 | 0.01260778 | 0.0215755 |
| ALKBH3 | EIF3A | -2.51 | 0.05 | -0.12 | 0.01244798 | 0.02131649 |
| ELP1 | MRM3 | -2.51 | 0.05 | -0.12 | 0.01234609 | 0.02114916 |
| ALYREF | YTHDC1 | -2.52 | 0.05 | -0.12 | 0.01220148 | 0.02090855 |
| TRMT10C | TRMT10B | -2.52 | 0.05 | -0.12 | 0.01218555 | 0.02088833 |
| MRM3 | METTL14 | -2.52 | 0.05 | -0.12 | 0.01216151 | 0.0208542 |
| RPUSD1 | TRMT13 | -2.52 | 0.05 | -0.12 | 0.0121319 | 0.02082452 |
| MRM1 | CMTR2 | -2.52 | 0.05 | -0.12 | 0.0119154 | 0.02048784 |
| RPUSD2 | G3BP1 | -2.54 | 0.05 | -0.12 | 0.01124942 | 0.01946201 |
| FBL | G3BP2 | -2.57 | 0.05 | -0.12 | 0.01062657 | 0.01847941 |
| CMTR2 | DNMT3A | -2.57 | 0.05 | -0.12 | 0.01055814 | 0.01836673 |
| CTU2 | ALKBH8 | -2.57 | 0.05 | -0.12 | 0.01047994 | 0.01824955 |
| CTU1 | YTHDC1 | -2.57 | 0.05 | -0.12 | 0.01043979 | 0.01818591 |
| ALKBH8 | ALKBH5 | -2.57 | 0.05 | -0.12 | 0.01034881 | 0.01803986 |
| PUS10 | TRMT6 | -2.58 | 0.05 | -0.12 | 0.01025637 | 0.01791236 |
| ELP3 | MRM2 | -2.59 | 0.05 | -0.12 | 0.00988736 | 0.01733119 |
| RPUSD3 | WTAP | -2.60 | 0.05 | -0.12 | 0.00967407 | 0.01698681 |
| ADAT3 | KIAA1429 | -2.60 | 0.05 | -0.12 | 0.00964258 | 0.01693741 |
| ADAT3 | ALKBH1 | -2.60 | 0.05 | -0.12 | 0.00960936 | 0.0168888 |
| MRM3 | ZC3H13 | -2.60 | 0.05 | -0.12 | 0.00958316 | 0.01685649 |
| ADARB1 | HNRNPA1 | -2.60 | 0.05 | -0.12 | 0.00948178 | 0.0166956 |
| ALKBH3 | TET1 | -2.61 | 0.05 | -0.12 | 0.00937641 | 0.01652159 |
| METTL1 | G3BP2 | -2.62 | 0.05 | -0.12 | 0.00905381 | 0.01598662 |
| MRM3 | G3BP1 | -2.62 | 0.05 | -0.12 | 0.0090283 | 0.01595273 |
| NSUN6 | G3BP2 | -2.62 | 0.05 | -0.12 | 0.00899134 | 0.01589299 |
| BUD23 | TET1 | -2.63 | 0.05 | -0.12 | 0.00893755 | 0.01580897 |
| TRMT10C | TET2 | -2.63 | 0.05 | -0.12 | 0.0089269 | 0.01579567 |
| ADARB1 | PUS1 | -2.63 | 0.05 | -0.12 | 0.00892552 | 0.01579567 |
| ADARB2 | TRMT10A | -2.63 | 0.05 | -0.12 | 0.00869779 | 0.01544439 |
| ELP3 | YBX1 | -2.66 | 0.05 | -0.12 | 0.0080937 | 0.01443772 |
| CTU2 | YTHDF3 | -2.67 | 0.05 | -0.12 | 0.00796367 | 0.01423594 |
| PUS1 | ALKBH3 | -2.67 | 0.05 | -0.12 | 0.00778153 | 0.01394489 |
| TRUB1 | FBLL1 | -2.68 | 0.05 | -0.12 | 0.00765222 | 0.01372291 |
| PUS10 | METTL1 | -2.69 | 0.05 | -0.12 | 0.00744444 | 0.01337404 |
| ADAT3 | TRMT61B | -2.69 | 0.05 | -0.12 | 0.00734152 | 0.01319853 |
| BUD23 | HNRNPA1 | -2.70 | 0.05 | -0.12 | 0.00715419 | 0.01288165 |
| CMTR2 | PRRC2A | -2.70 | 0.05 | -0.12 | 0.00708175 | 0.01277231 |
| TRMT61A | EIF3A | -2.71 | 0.05 | -0.12 | 0.00706113 | 0.01274435 |
| TRMT61A | WTAP | -2.72 | 0.05 | -0.12 | 0.00679726 | 0.01229888 |
| TRMT10B | TRMT10A | -2.72 | 0.05 | -0.12 | 0.00678029 | 0.01227697 |
| CMTR2 | DNMT3B | -2.72 | 0.05 | -0.12 | 0.00676597 | 0.01225859 |
| METTL1 | TET3 | -2.72 | 0.05 | -0.12 | 0.00673894 | 0.01221524 |
| CTU2 | RNMT | -2.72 | 0.05 | -0.12 | 0.00672358 | 0.01219177 |
| RRP8 | ALYREF | -2.73 | 0.05 | -0.13 | 0.00658075 | 0.01194136 |
| RPUSD2 | FMR1 | -2.73 | 0.05 | -0.13 | 0.00654144 | 0.01187429 |
| MRM1 | TET2 | -2.74 | 0.05 | -0.13 | 0.00635409 | 0.01155081 |
| MRM3 | DNMT1 | -2.75 | 0.05 | -0.13 | 0.00626469 | 0.01140655 |
| ADAT3 | RBMX | -2.77 | 0.05 | -0.13 | 0.00591356 | 0.01082795 |
| TRMT61A | FTO | -2.77 | 0.05 | -0.13 | 0.00581918 | 0.01067719 |
| NSUN5 | METTL16 | -2.79 | 0.05 | -0.13 | 0.00551594 | 0.01016847 |
| ADAT3 | CBLL1 | -2.79 | 0.05 | -0.13 | 0.00547946 | 0.01011008 |
| RPUSD2 | FTO | -2.82 | 0.05 | -0.13 | 0.00506791 | 0.00939536 |
| TRMT13 | ALKBH5 | -2.82 | 0.05 | -0.13 | 0.00504888 | 0.00936351 |
| ELP1 | BUD23 | -2.83 | 0.05 | -0.13 | 0.00489801 | 0.00910376 |
| TRUB1 | NSUN5 | -2.83 | 0.05 | -0.13 | 0.00489524 | 0.00910198 |
| TRMT61A | G3BP1 | -2.83 | 0.05 | -0.13 | 0.00481953 | 0.0089678 |
| NSUN5 | G3BP1 | -2.84 | 0.05 | -0.13 | 0.00476271 | 0.00887517 |
| RRP8 | IGF2BP1 | -2.84 | 0.05 | -0.13 | 0.00472353 | 0.00881191 |
| TRMT112 | METTL16 | -2.85 | 0.05 | -0.13 | 0.00455825 | 0.0085193 |
| ADARB1 | FTSJ1 | -2.86 | 0.05 | -0.13 | 0.00448031 | 0.00838606 |
| TRMT112 | PRRC2A | -2.87 | 0.05 | -0.13 | 0.00431482 | 0.0080943 |
| ADAT2 | TRMT112 | -2.87 | 0.05 | -0.13 | 0.0042345 | 0.0079584 |
| ALYREF | NSUN4 | -2.88 | 0.05 | -0.13 | 0.00414266 | 0.0078003 |
| CTU2 | TET3 | -2.90 | 0.05 | -0.13 | 0.00395224 | 0.00746402 |
| MRM3 | CMTR2 | -2.91 | 0.05 | -0.13 | 0.00383064 | 0.00725337 |
| ALKBH8 | METTL1 | -2.91 | 0.05 | -0.13 | 0.00382511 | 0.00724562 |
| TRMT10B | G3BP2 | -2.91 | 0.05 | -0.13 | 0.00379714 | 0.00719803 |
| TRMT10B | IGF2BP1 | -2.92 | 0.05 | -0.13 | 0.00367479 | 0.00697658 |
| TRMT61A | METTL14 | -2.92 | 0.05 | -0.13 | 0.00361498 | 0.00687853 |
| NSUN4 | IGF2BP2 | -2.93 | 0.05 | -0.13 | 0.00361148 | 0.00687447 |
| ADAT3 | TRMT10C | -2.93 | 0.05 | -0.13 | 0.00360613 | 0.00686687 |
| FBL | TRMT10B | -2.93 | 0.05 | -0.13 | 0.00357805 | 0.00681597 |
| ADARB1 | YBX1 | -2.95 | 0.05 | -0.13 | 0.00335413 | 0.00643311 |
| ELP3 | ELAVL1 | -2.97 | 0.05 | -0.14 | 0.00314491 | 0.00606176 |
| ELP1 | METTL5 | -2.97 | 0.05 | -0.14 | 0.00308891 | 0.00596747 |
| PUS1 | TRDMT1 | -2.98 | 0.05 | -0.14 | 0.00302096 | 0.00584963 |
| RBM15B | WTAP | -2.99 | 0.05 | -0.14 | 0.00295339 | 0.00573198 |
| TGS1 | BUD23 | -2.99 | 0.05 | -0.14 | 0.00293088 | 0.00569487 |
| ADAT3 | ZC3H13 | -2.99 | 0.05 | -0.14 | 0.00289407 | 0.00562767 |
| RPUSD3 | RBM15 | -3.00 | 0.05 | -0.14 | 0.00280393 | 0.0054629 |
| METTL5 | METTL14 | -3.01 | 0.05 | -0.14 | 0.0027905 | 0.00544094 |
| RPUSD1 | RBMX | -3.02 | 0.05 | -0.14 | 0.00270557 | 0.00528554 |
| ALKBH8 | PUS1 | -3.02 | 0.05 | -0.14 | 0.00269751 | 0.00527591 |
| RPUSD3 | YTHDC1 | -3.02 | 0.05 | -0.14 | 0.00264101 | 0.00517141 |
| ELP3 | RPUSD1 | -3.04 | 0.05 | -0.14 | 0.002533 | 0.00497342 |
| IGF2BP1 | METTL14 | -3.05 | 0.05 | -0.14 | 0.0024401 | 0.00480409 |
| TRMT10B | METTL5 | -3.05 | 0.05 | -0.14 | 0.00239938 | 0.00473131 |
| TRMT112 | CMTR2 | -3.05 | 0.05 | -0.14 | 0.00238956 | 0.00471377 |
| TARBP1 | WTAP | -3.05 | 0.05 | -0.14 | 0.00238297 | 0.00470445 |
| FBLL1 | TRMT10A | -3.06 | 0.05 | -0.14 | 0.00235901 | 0.00466628 |
| MRM1 | YTHDF3 | -3.06 | 0.05 | -0.14 | 0.00233686 | 0.00462789 |
| TRMT44 | YBX1 | -3.07 | 0.05 | -0.14 | 0.00226779 | 0.00449993 |
| PUS10 | ALKBH5 | -3.07 | 0.05 | -0.14 | 0.00223434 | 0.00444229 |
| RPUSD3 | FMR1 | -3.09 | 0.05 | -0.14 | 0.00213911 | 0.00427315 |
| ALKBH8 | RPUSD2 | -3.12 | 0.05 | -0.14 | 0.00191963 | 0.0038622 |
| MRM3 | FTO | -3.12 | 0.05 | -0.14 | 0.00190818 | 0.00384224 |
| TRMT61A | TET2 | -3.13 | 0.05 | -0.14 | 0.00184711 | 0.00372967 |
| ADARB2 | ALYREF | -3.13 | 0.05 | -0.14 | 0.00182754 | 0.00369459 |
| YBX1 | NSUN6 | -3.14 | 0.05 | -0.14 | 0.00180543 | 0.0036572 |
| ADARB1 | TRMT61B | -3.14 | 0.05 | -0.14 | 0.00178001 | 0.00360716 |
| PUS10 | DNMT1 | -3.17 | 0.05 | -0.14 | 0.00161744 | 0.00330423 |
| METTL1 | CMTR2 | -3.18 | 0.05 | -0.15 | 0.00159345 | 0.00326182 |
| PUS10 | HNRNPC | -3.19 | 0.05 | -0.15 | 0.00153862 | 0.00315726 |
| ADAR | RPUSD3 | -3.19 | 0.05 | -0.15 | 0.0015384 | 0.00315726 |
| CTU1 | TRMT10A | -3.19 | 0.05 | -0.15 | 0.00152543 | 0.00313402 |
| METTL5 | RBM15B | -3.21 | 0.05 | -0.15 | 0.00142947 | 0.00295491 |
| NSUN5 | YTHDC2 | -3.21 | 0.05 | -0.15 | 0.00142907 | 0.00295491 |
| NOP2 | G3BP2 | -3.22 | 0.05 | -0.15 | 0.00138666 | 0.00287818 |
| PUS10 | WDR4 | -3.22 | 0.05 | -0.15 | 0.00136125 | 0.00282894 |
| ALKBH3 | TET3 | -3.23 | 0.05 | -0.15 | 0.00133972 | 0.00278648 |
| PUS7L | TRMT112 | -3.25 | 0.05 | -0.15 | 0.00122929 | 0.0025695 |
| CTU2 | NSUN3 | -3.26 | 0.05 | -0.15 | 0.00120175 | 0.0025161 |
| YBX1 | METTL14 | -3.27 | 0.05 | -0.15 | 0.00116871 | 0.00244895 |
| RNMT | MRM3 | -3.27 | 0.05 | -0.15 | 0.00115575 | 0.00242583 |
| RPUSD1 | PUS10 | -3.27 | 0.05 | -0.15 | 0.00114165 | 0.00239723 |
| TRMT61A | YTHDC2 | -3.29 | 0.05 | -0.15 | 0.00107546 | 0.00226862 |
| EIF3A | METTL5 | -3.29 | 0.05 | -0.15 | 0.0010673 | 0.00225234 |
| HNRNPC | METTL16 | -3.29 | 0.05 | -0.15 | 0.00106608 | 0.00225072 |
| METTL1 | YTHDC1 | -3.30 | 0.05 | -0.15 | 0.00103 | 0.00218367 |
| PUS7L | BUD23 | -3.31 | 0.05 | -0.15 | 0.00098857 | 0.00209936 |
| ELP1 | RPUSD1 | -3.32 | 0.05 | -0.15 | 0.0009664 | 0.00205574 |
| PUS1 | YTHDC2 | -3.33 | 0.05 | -0.15 | 0.00094612 | 0.002016 |
| ADARB2 | TRMT10C | -3.36 | 0.05 | -0.15 | 0.00083422 | 0.00179499 |
| ADAT3 | WTAP | -3.37 | 0.05 | -0.15 | 0.00081591 | 0.00175784 |
| RPUSD1 | FTO | -3.37 | 0.05 | -0.15 | 0.00080758 | 0.00174355 |
| YBX1 | FTO | -3.39 | 0.05 | -0.15 | 0.0007498 | 0.0016237 |
| CTU2 | G3BP2 | -3.39 | 0.05 | -0.15 | 0.00074769 | 0.00162042 |
| METTL1 | METTL16 | -3.41 | 0.05 | -0.16 | 0.0007155 | 0.00155308 |
| RPUSD1 | EIF3A | -3.41 | 0.05 | -0.16 | 0.00071441 | 0.0015524 |
| YTHDC1 | METTL5 | -3.41 | 0.05 | -0.16 | 0.00070338 | 0.00153106 |
| IGF2BP2 | YTHDF2 | -3.41 | 0.05 | -0.16 | 0.00069387 | 0.00151167 |
| ALYREF | METTL14 | -3.45 | 0.05 | -0.16 | 0.00061859 | 0.00135394 |
| ADARB1 | ZCCHC4 | -3.47 | 0.05 | -0.16 | 0.00056721 | 0.00125236 |
| CTU1 | G3BP1 | -3.47 | 0.05 | -0.16 | 0.00056231 | 0.00124274 |
| NSUN5 | EIF3A | -3.48 | 0.05 | -0.16 | 0.00054535 | 0.00120631 |
| CMTR2 | ALKBH5 | -3.50 | 0.05 | -0.16 | 0.00051172 | 0.00113641 |
| BUD23 | TET3 | -3.51 | 0.05 | -0.16 | 0.00048731 | 0.00108794 |
| RPUSD1 | TGS1 | -3.51 | 0.05 | -0.16 | 0.00048335 | 0.00108006 |
| FTSJ1 | METTL16 | -3.52 | 0.05 | -0.16 | 0.00047128 | 0.00105682 |
| MRM3 | TET3 | -3.53 | 0.05 | -0.16 | 0.00046348 | 0.0010398 |
| ADAT2 | TRUB1 | -3.53 | 0.05 | -0.16 | 0.00046299 | 0.00103916 |
| RPUSD1 | TET1 | -3.53 | 0.05 | -0.16 | 0.00045898 | 0.00103107 |
| RPUSD2 | ZC3H13 | -3.53 | 0.05 | -0.16 | 0.00045783 | 0.00102895 |
| NOP2 | METTL14 | -3.56 | 0.05 | -0.16 | 0.0004068 | 0.0009233 |
| PUS10 | HNRNPA1 | -3.56 | 0.05 | -0.16 | 0.00040611 | 0.00092215 |
| IGF2BP1 | YTHDF2 | -3.59 | 0.05 | -0.16 | 0.00036779 | 0.0008408 |
| RRP8 | IGF2BP3 | -3.59 | 0.05 | -0.16 | 0.00036726 | 0.00083998 |
| ALKBH8 | MRM3 | -3.61 | 0.05 | -0.16 | 0.00034217 | 0.00078543 |
| NSUN5 | YTHDC1 | -3.61 | 0.05 | -0.16 | 0.00033952 | 0.00077971 |
| ADARB2 | BUD23 | -3.62 | 0.05 | -0.16 | 0.00032939 | 0.00075816 |
| ELP3 | IGF2BP2 | -3.62 | 0.05 | -0.17 | 0.0003218 | 0.00074205 |
| ADARB1 | LRPPRC | -3.62 | 0.05 | -0.17 | 0.00032151 | 0.00074171 |
| ADAT2 | FTO | -3.63 | 0.05 | -0.17 | 0.00030987 | 0.00071684 |
| ALYREF | ZC3H13 | -3.64 | 0.05 | -0.17 | 0.00030636 | 0.00070936 |
| TRMT112 | NSUN3 | -3.64 | 0.05 | -0.17 | 0.00029893 | 0.00069375 |
| RRP8 | IGF2BP2 | -3.66 | 0.05 | -0.17 | 0.00027861 | 0.00064837 |
| METTL5 | METTL16 | -3.66 | 0.05 | -0.17 | 0.00027659 | 0.00064428 |
| TRMT112 | TRMT44 | -3.67 | 0.05 | -0.17 | 0.00026706 | 0.00062379 |
| CMTR2 | RBM15B | -3.68 | 0.05 | -0.17 | 0.00026497 | 0.0006192 |
| ADARB1 | TRUB1 | -3.68 | 0.05 | -0.17 | 0.00026372 | 0.00061685 |
| DNMT3B | FTO | -3.68 | 0.05 | -0.17 | 0.00026042 | 0.0006097 |
| ADARB2 | MRM2 | -3.68 | 0.05 | -0.17 | 0.0002588 | 0.00060675 |
| CTU1 | ZCCHC4 | -3.70 | 0.05 | -0.17 | 0.00024471 | 0.00057694 |
| CTU1 | NSUN3 | -3.70 | 0.05 | -0.17 | 0.00024342 | 0.00057442 |
| TRMT61A | YTHDF3 | -3.70 | 0.05 | -0.17 | 0.00023962 | 0.00056625 |
| CTU1 | TET2 | -3.71 | 0.05 | -0.17 | 0.00022925 | 0.00054302 |
| RRP8 | NSUN2 | -3.72 | 0.05 | -0.17 | 0.00022515 | 0.00053405 |
| RPUSD3 | EIF3A | -3.72 | 0.05 | -0.17 | 0.00022105 | 0.00052483 |
| METTL1 | TRMT10B | -3.73 | 0.05 | -0.17 | 0.00021162 | 0.00050386 |
| RPUSD1 | CMTR2 | -3.74 | 0.05 | -0.17 | 0.00021052 | 0.00050147 |
| YBX1 | METTL3 | -3.74 | 0.05 | -0.17 | 0.00020838 | 0.00049708 |
| ADAT3 | LRPPRC | -3.75 | 0.05 | -0.17 | 0.00019602 | 0.00046848 |
| CTU2 | ADARB2 | -3.76 | 0.05 | -0.17 | 0.00018802 | 0.00045042 |
| FBL | YTHDC2 | -3.78 | 0.05 | -0.17 | 0.00017892 | 0.00042944 |
| TET2 | HNRNPC | -3.78 | 0.05 | -0.17 | 0.0001777 | 0.00042672 |
| ALKBH8 | TRMT112 | -3.79 | 0.05 | -0.17 | 0.00016849 | 0.00040576 |
| CTU2 | G3BP1 | -3.80 | 0.05 | -0.17 | 0.00016453 | 0.00039717 |
| TRMT112 | CMTR1 | -3.82 | 0.05 | -0.17 | 0.00015246 | 0.00036945 |
| PUS10 | NSUN2 | -3.82 | 0.05 | -0.17 | 0.00015221 | 0.00036902 |
| ADAR | BUD23 | -3.82 | 0.05 | -0.17 | 0.00015221 | 0.00036902 |
| TRUB1 | TRMT10B | -3.85 | 0.05 | -0.18 | 0.00013317 | 0.00032523 |
| FTSJ1 | TRMT10B | -3.87 | 0.05 | -0.18 | 0.00012607 | 0.0003089 |
| ELP3 | ALYREF | -3.87 | 0.05 | -0.18 | 0.00012353 | 0.00030326 |
| MRM3 | FMR1 | -3.87 | 0.05 | -0.18 | 0.00012276 | 0.00030153 |
| IGF2BP1 | YTHDC2 | -3.89 | 0.05 | -0.18 | 0.00011537 | 0.00028475 |
| TRMT10C | YTHDC2 | -3.90 | 0.05 | -0.18 | 0.00010907 | 0.00027026 |
| TARBP1 | HNRNPC | -3.90 | 0.05 | -0.18 | 0.00010822 | 0.0002683 |
| ADAT3 | TRMT10A | -3.91 | 0.05 | -0.18 | 0.0001073 | 0.00026626 |
| CMTR2 | ALYREF | -3.92 | 0.05 | -0.18 | 0.00010376 | 0.00025825 |
| ADAT3 | HNRNPC | -3.96 | 0.05 | -0.18 | 8.81E-05 | 0.00022112 |
| ADAT3 | NSUN3 | -3.96 | 0.05 | -0.18 | 8.64E-05 | 0.00021747 |
| ALKBH8 | RPUSD1 | -3.98 | 0.05 | -0.18 | 7.98E-05 | 0.00020185 |
| PUS10 | RBM15B | -3.99 | 0.05 | -0.18 | 7.68E-05 | 0.0001947 |
| FTSJ1 | YTHDC2 | -3.99 | 0.05 | -0.18 | 7.53E-05 | 0.00019128 |
| TRMT112 | TET1 | -4.00 | 0.05 | -0.18 | 7.37E-05 | 0.00018751 |
| TRMT10B | IGF2BP3 | -4.00 | 0.05 | -0.18 | 7.27E-05 | 0.00018494 |
| ADAT2 | YTHDF3 | -4.01 | 0.05 | -0.18 | 7.07E-05 | 0.0001806 |
| RPUSD1 | G3BP1 | -4.02 | 0.05 | -0.18 | 6.77E-05 | 0.0001735 |
| ADAT3 | METTL14 | -4.04 | 0.05 | -0.18 | 6.20E-05 | 0.00015955 |
| CTU2 | YTHDC1 | -4.04 | 0.05 | -0.18 | 6.13E-05 | 0.00015804 |
| ADARB2 | FTSJ1 | -4.04 | 0.05 | -0.18 | 6.12E-05 | 0.00015787 |
| CMTR2 | HNRNPA1 | -4.05 | 0.05 | -0.18 | 5.95E-05 | 0.00015367 |
| YBX1 | YTHDC1 | -4.06 | 0.05 | -0.18 | 5.66E-05 | 0.00014638 |
| RPUSD3 | METTL14 | -4.07 | 0.05 | -0.18 | 5.60E-05 | 0.00014512 |
| TARBP1 | ALYREF | -4.08 | 0.05 | -0.18 | 5.40E-05 | 0.00014035 |
| ADARB1 | DKC1 | -4.08 | 0.05 | -0.19 | 5.25E-05 | 0.00013658 |
| PUS10 | EIF3A | -4.08 | 0.05 | -0.19 | 5.25E-05 | 0.00013656 |
| TRMT112 | G3BP2 | -4.09 | 0.05 | -0.19 | 5.08E-05 | 0.00013252 |
| YBX1 | NSUN7 | -4.09 | 0.05 | -0.19 | 5.02E-05 | 0.0001312 |
| TRMT112 | TARBP1 | -4.11 | 0.05 | -0.19 | 4.75E-05 | 0.00012449 |
| RPUSD3 | RNMT | -4.11 | 0.05 | -0.19 | 4.60E-05 | 0.00012082 |
| RPUSD1 | TRUB1 | -4.16 | 0.05 | -0.19 | 3.79E-05 | 0.00010106 |
| BUD23 | G3BP1 | -4.16 | 0.05 | -0.19 | 3.73E-05 | 9.94E-05 |
| ALKBH3 | NOP2 | -4.17 | 0.05 | -0.19 | 3.59E-05 | 9.58E-05 |
| METTL1 | EIF3A | -4.18 | 0.05 | -0.19 | 3.51E-05 | 9.38E-05 |
| CTU2 | ADARB1 | -4.19 | 0.05 | -0.19 | 3.34E-05 | 8.96E-05 |
| BUD23 | YTHDC1 | -4.20 | 0.05 | -0.19 | 3.24E-05 | 8.72E-05 |
| RNMT | BUD23 | -4.21 | 0.05 | -0.19 | 3.05E-05 | 8.23E-05 |
| NSUN5 | METTL14 | -4.21 | 0.05 | -0.19 | 3.03E-05 | 8.19E-05 |
| ADAT3 | TRDMT1 | -4.24 | 0.05 | -0.19 | 2.73E-05 | 7.43E-05 |
| MRM3 | G3BP2 | -4.24 | 0.05 | -0.19 | 2.70E-05 | 7.34E-05 |
| METTL5 | ZC3H13 | -4.25 | 0.05 | -0.19 | 2.61E-05 | 7.11E-05 |
| RPUSD2 | TET2 | -4.25 | 0.05 | -0.19 | 2.53E-05 | 6.92E-05 |
| TRMT61A | ZC3H13 | -4.26 | 0.05 | -0.19 | 2.51E-05 | 6.86E-05 |
| YBX1 | METTL16 | -4.26 | 0.05 | -0.19 | 2.45E-05 | 6.72E-05 |
| ALKBH3 | IGF2BP1 | -4.27 | 0.05 | -0.19 | 2.34E-05 | 6.41E-05 |
| ELP3 | FTSJ1 | -4.28 | 0.05 | -0.19 | 2.30E-05 | 6.32E-05 |
| HENMT1 | RRP8 | -4.28 | 0.05 | -0.19 | 2.25E-05 | 6.19E-05 |
| TRMT112 | G3BP1 | -4.28 | 0.05 | -0.19 | 2.23E-05 | 6.14E-05 |
| MRM3 | TET2 | -4.33 | 0.05 | -0.20 | 1.83E-05 | 5.10E-05 |
| CTU1 | ALKBH8 | -4.35 | 0.05 | -0.20 | 1.64E-05 | 4.61E-05 |
| RPUSD3 | CBLL1 | -4.37 | 0.05 | -0.20 | 1.56E-05 | 4.39E-05 |
| TRMT112 | TRMT10B | -4.37 | 0.05 | -0.20 | 1.54E-05 | 4.35E-05 |
| FTSJ1 | METTL14 | -4.39 | 0.05 | -0.20 | 1.38E-05 | 3.91E-05 |
| ELP1 | METTL1 | -4.40 | 0.05 | -0.20 | 1.37E-05 | 3.89E-05 |
| CTU1 | TRUB1 | -4.40 | 0.05 | -0.20 | 1.33E-05 | 3.79E-05 |
| FBL | METTL14 | -4.41 | 0.05 | -0.20 | 1.26E-05 | 3.59E-05 |
| CTU2 | METTL16 | -4.42 | 0.05 | -0.20 | 1.21E-05 | 3.48E-05 |
| FTSJ1 | YTHDC1 | -4.43 | 0.05 | -0.20 | 1.18E-05 | 3.39E-05 |
| NSUN5 | G3BP2 | -4.45 | 0.05 | -0.20 | 1.09E-05 | 3.14E-05 |
| BUD23 | YTHDC2 | -4.45 | 0.05 | -0.20 | 1.09E-05 | 3.13E-05 |
| ALKBH8 | BUD23 | -4.45 | 0.05 | -0.20 | 1.09E-05 | 3.13E-05 |
| ADARB1 | RPUSD2 | -4.45 | 0.05 | -0.20 | 1.05E-05 | 3.04E-05 |
| ADARB1 | FBL | -4.45 | 0.05 | -0.20 | 1.05E-05 | 3.04E-05 |
| CTU2 | ELP1 | -4.47 | 0.05 | -0.20 | 9.77E-06 | 2.84E-05 |
| BUD23 | METTL14 | -4.48 | 0.05 | -0.20 | 9.24E-06 | 2.70E-05 |
| FBLL1 | G3BP2 | -4.51 | 0.05 | -0.20 | 8.29E-06 | 2.44E-05 |
| PUS10 | FTSJ1 | -4.53 | 0.05 | -0.20 | 7.39E-06 | 2.19E-05 |
| CTU1 | ZC3H13 | -4.53 | 0.05 | -0.20 | 7.38E-06 | 2.19E-05 |
| RPUSD1 | TRDMT1 | -4.55 | 0.05 | -0.21 | 6.90E-06 | 2.05E-05 |
| METTL1 | FTO | -4.55 | 0.05 | -0.21 | 6.81E-06 | 2.03E-05 |
| NOP2 | FTO | -4.59 | 0.05 | -0.21 | 5.65E-06 | 1.69E-05 |
| ADAT3 | G3BP1 | -4.60 | 0.05 | -0.21 | 5.45E-06 | 1.64E-05 |
| ADARB1 | RPUSD3 | -4.60 | 0.05 | -0.21 | 5.37E-06 | 1.61E-05 |
| ADARB2 | HNRNPC | -4.61 | 0.05 | -0.21 | 5.26E-06 | 1.58E-05 |
| RPUSD3 | PUS7L | -4.62 | 0.05 | -0.21 | 4.85E-06 | 1.47E-05 |
| ADARB2 | METTL1 | -4.63 | 0.05 | -0.21 | 4.79E-06 | 1.45E-05 |
| ALYREF | METTL16 | -4.63 | 0.05 | -0.21 | 4.64E-06 | 1.41E-05 |
| CTU1 | PUS10 | -4.65 | 0.05 | -0.21 | 4.33E-06 | 1.32E-05 |
| ADAT3 | CMTR2 | -4.73 | 0.05 | -0.21 | 2.92E-06 | 9.16E-06 |
| ALKBH3 | DNMT3A | -4.74 | 0.05 | -0.21 | 2.87E-06 | 9.02E-06 |
| FTO | HNRNPC | -4.74 | 0.05 | -0.21 | 2.84E-06 | 8.93E-06 |
| NSUN5 | YTHDF3 | -4.75 | 0.05 | -0.21 | 2.76E-06 | 8.70E-06 |
| CTU2 | METTL14 | -4.77 | 0.05 | -0.21 | 2.51E-06 | 7.94E-06 |
| RPUSD1 | YTHDC1 | -4.77 | 0.05 | -0.22 | 2.44E-06 | 7.75E-06 |
| BUD23 | G3BP2 | -4.78 | 0.05 | -0.22 | 2.37E-06 | 7.54E-06 |
| PUS10 | ELAVL1 | -4.84 | 0.05 | -0.22 | 1.73E-06 | 5.59E-06 |
| TRMT61A | NSUN3 | -4.85 | 0.05 | -0.22 | 1.70E-06 | 5.51E-06 |
| ADARB1 | MRM3 | -4.87 | 0.05 | -0.22 | 1.55E-06 | 5.03E-06 |
| BUD23 | YTHDF3 | -4.87 | 0.05 | -0.22 | 1.53E-06 | 4.98E-06 |
| TRMT44 | HNRNPC | -4.91 | 0.05 | -0.22 | 1.24E-06 | 4.07E-06 |
| RPUSD3 | TET2 | -4.93 | 0.05 | -0.22 | 1.15E-06 | 3.78E-06 |
| ADAT3 | ZCCHC4 | -4.93 | 0.05 | -0.22 | 1.14E-06 | 3.78E-06 |
| TRDMT1 | NOP2 | -4.95 | 0.05 | -0.22 | 1.03E-06 | 3.44E-06 |
| BUD23 | TET2 | -4.95 | 0.05 | -0.22 | 1.02E-06 | 3.42E-06 |
| ELP1 | RPUSD3 | -4.97 | 0.05 | -0.22 | 9.51E-07 | 3.18E-06 |
| PUS1 | TET2 | -4.97 | 0.05 | -0.22 | 9.48E-07 | 3.18E-06 |
| RPUSD1 | YTHDC2 | -5.01 | 0.04 | -0.23 | 7.78E-07 | 2.65E-06 |
| ADAT3 | TGS1 | -5.02 | 0.04 | -0.23 | 7.27E-07 | 2.48E-06 |
| PUS10 | PRRC2A | -5.02 | 0.04 | -0.23 | 7.26E-07 | 2.48E-06 |
| G3BP2 | METTL3 | -5.05 | 0.04 | -0.23 | 6.20E-07 | 2.14E-06 |
| PUS10 | FBL | -5.09 | 0.04 | -0.23 | 5.24E-07 | 1.83E-06 |
| CTU1 | FTO | -5.09 | 0.04 | -0.23 | 5.14E-07 | 1.79E-06 |
| METTL1 | METTL14 | -5.14 | 0.04 | -0.23 | 4.12E-07 | 1.45E-06 |
| CMTR2 | ELAVL1 | -5.14 | 0.04 | -0.23 | 3.97E-07 | 1.40E-06 |
| HNRNPC | YTHDC2 | -5.20 | 0.04 | -0.23 | 2.99E-07 | 1.08E-06 |
| RPUSD3 | ZC3H13 | -5.22 | 0.04 | -0.23 | 2.65E-07 | 9.59E-07 |
| TRMT10B | HNRNPC | -5.24 | 0.04 | -0.24 | 2.44E-07 | 8.86E-07 |
| RPUSD1 | NSUN3 | -5.35 | 0.04 | -0.24 | 1.36E-07 | 5.08E-07 |
| TRUB1 | TRMT61A | -5.38 | 0.04 | -0.24 | 1.15E-07 | 4.33E-07 |
| CMTR2 | FBL | -5.39 | 0.04 | -0.24 | 1.13E-07 | 4.26E-07 |
| RPUSD2 | YTHDC2 | -5.39 | 0.04 | -0.24 | 1.09E-07 | 4.13E-07 |
| TET2 | YBX1 | -5.42 | 0.04 | -0.24 | 9.38E-08 | 3.56E-07 |
| TET2 | ALYREF | -5.52 | 0.04 | -0.25 | 5.61E-08 | 2.18E-07 |
| TRMT112 | RNMT | -5.55 | 0.04 | -0.25 | 4.88E-08 | 1.91E-07 |
| ELP3 | NSUN5 | -5.58 | 0.04 | -0.25 | 4.13E-08 | 1.63E-07 |
| PUS10 | IGF2BP3 | -5.59 | 0.04 | -0.25 | 3.78E-08 | 1.50E-07 |
| TARBP1 | G3BP2 | -5.64 | 0.04 | -0.25 | 3.02E-08 | 1.21E-07 |
| CTU2 | YTHDC2 | -5.65 | 0.04 | -0.25 | 2.84E-08 | 1.14E-07 |
| TRMT10B | ALYREF | -5.66 | 0.04 | -0.25 | 2.61E-08 | 1.05E-07 |
| TRMT112 | FTO | -5.69 | 0.04 | -0.25 | 2.29E-08 | 9.27E-08 |
| MRM3 | YTHDC2 | -5.70 | 0.04 | -0.25 | 2.16E-08 | 8.80E-08 |
| PUS10 | DNMT3B | -5.70 | 0.04 | -0.25 | 2.16E-08 | 8.79E-08 |
| TRMT10A | FTO | -5.70 | 0.04 | -0.25 | 2.09E-08 | 8.54E-08 |
| RPUSD1 | YTHDF3 | -5.73 | 0.04 | -0.26 | 1.75E-08 | 7.24E-08 |
| ADAT3 | YTHDF3 | -5.74 | 0.04 | -0.26 | 1.68E-08 | 6.95E-08 |
| BUD23 | FTO | -5.77 | 0.04 | -0.26 | 1.48E-08 | 6.18E-08 |
| NSUN5 | FTO | -5.81 | 0.04 | -0.26 | 1.12E-08 | 4.78E-08 |
| RRP8 | YBX1 | -5.83 | 0.04 | -0.26 | 1.03E-08 | 4.40E-08 |
| ADAR | TRMT112 | -5.86 | 0.04 | -0.26 | 8.86E-09 | 3.82E-08 |
| CMTR2 | NOP2 | -5.86 | 0.04 | -0.26 | 8.66E-09 | 3.73E-08 |
| YTHDC2 | METTL5 | -5.90 | 0.04 | -0.26 | 7.12E-09 | 3.09E-08 |
| CTU1 | G3BP2 | -5.92 | 0.04 | -0.26 | 6.15E-09 | 2.69E-08 |
| FBL | TET2 | -5.94 | 0.04 | -0.26 | 5.56E-09 | 2.44E-08 |
| BUD23 | EIF3A | -5.94 | 0.04 | -0.26 | 5.48E-09 | 2.41E-08 |
| PUS1 | CMTR2 | -5.97 | 0.04 | -0.27 | 4.57E-09 | 2.03E-08 |
| RPUSD3 | G3BP2 | -5.99 | 0.04 | -0.27 | 4.27E-09 | 1.91E-08 |
| PUS1 | METTL14 | -6.02 | 0.04 | -0.27 | 3.44E-09 | 1.56E-08 |
| FBL | FTO | -6.04 | 0.04 | -0.27 | 3.10E-09 | 1.42E-08 |
| PUS10 | YBX1 | -6.05 | 0.04 | -0.27 | 3.03E-09 | 1.39E-08 |
| RPUSD3 | FTO | -6.06 | 0.04 | -0.27 | 2.75E-09 | 1.27E-08 |
| ADAT2 | G3BP2 | -6.08 | 0.04 | -0.27 | 2.52E-09 | 1.17E-08 |
| TRMT10C | FTO | -6.09 | 0.04 | -0.27 | 2.39E-09 | 1.11E-08 |
| METTL1 | YTHDC2 | -6.22 | 0.04 | -0.28 | 1.09E-09 | 5.23E-09 |
| RPUSD1 | G3BP2 | -6.23 | 0.04 | -0.28 | 1.06E-09 | 5.11E-09 |
| ADARB2 | METTL5 | -6.23 | 0.04 | -0.28 | 1.02E-09 | 4.91E-09 |
| ADARB1 | BUD23 | -6.24 | 0.04 | -0.28 | 9.67E-10 | 4.68E-09 |
| CTU2 | EIF3A | -6.28 | 0.04 | -0.28 | 7.95E-10 | 3.87E-09 |
| FTSJ1 | ZC3H13 | -6.31 | 0.04 | -0.28 | 6.45E-10 | 3.17E-09 |
| TARBP1 | YBX1 | -6.32 | 0.04 | -0.28 | 6.21E-10 | 3.06E-09 |
| PUS10 | PUS1 | -6.33 | 0.04 | -0.28 | 5.75E-10 | 2.84E-09 |
| TET2 | NSUN5 | -6.34 | 0.04 | -0.28 | 5.46E-10 | 2.71E-09 |
| NSUN5 | ZC3H13 | -6.35 | 0.04 | -0.28 | 4.99E-10 | 2.49E-09 |
| PUS10 | IGF2BP2 | -6.39 | 0.04 | -0.28 | 3.95E-10 | 2.00E-09 |
| RPUSD3 | G3BP1 | -6.39 | 0.04 | -0.28 | 3.91E-10 | 1.98E-09 |
| TRMT61A | G3BP2 | -6.40 | 0.04 | -0.28 | 3.71E-10 | 1.88E-09 |
| ADARB1 | ALYREF | -6.45 | 0.04 | -0.29 | 2.84E-10 | 1.46E-09 |
| PUS1 | G3BP2 | -6.57 | 0.04 | -0.29 | 1.33E-10 | 7.15E-10 |
| TRMT112 | YTHDC1 | -6.57 | 0.04 | -0.29 | 1.31E-10 | 7.06E-10 |
| RPUSD3 | YTHDC2 | -6.59 | 0.04 | -0.29 | 1.22E-10 | 6.57E-10 |
| YBX1 | YTHDC2 | -6.62 | 0.04 | -0.29 | 9.91E-11 | 5.42E-10 |
| DKC1 | PUS10 | -6.63 | 0.04 | -0.29 | 9.44E-11 | 5.18E-10 |
| ADARB2 | TRMT112 | -6.75 | 0.04 | -0.30 | 4.39E-11 | 2.53E-10 |
| MRM1 | G3BP2 | -6.78 | 0.04 | -0.30 | 3.64E-11 | 2.11E-10 |
| ADAT3 | G3BP2 | -6.83 | 0.04 | -0.30 | 2.66E-11 | 1.57E-10 |
| PUS10 | IGF2BP1 | -6.84 | 0.04 | -0.30 | 2.49E-11 | 1.48E-10 |
| TRMT112 | METTL14 | -6.86 | 0.04 | -0.30 | 2.23E-11 | 1.34E-10 |
| PUS10 | ALYREF | -6.87 | 0.04 | -0.30 | 2.06E-11 | 1.24E-10 |
| CTU1 | METTL14 | -6.92 | 0.04 | -0.30 | 1.50E-11 | 9.23E-11 |
| METTL1 | ZC3H13 | -6.95 | 0.04 | -0.31 | 1.23E-11 | 7.69E-11 |
| ALYREF | FTO | -6.95 | 0.04 | -0.31 | 1.23E-11 | 7.65E-11 |
| BUD23 | ZC3H13 | -7.05 | 0.04 | -0.31 | 6.60E-12 | 4.24E-11 |
| ALYREF | YTHDC2 | -7.05 | 0.04 | -0.31 | 6.50E-12 | 4.19E-11 |
| FTO | METTL5 | -7.17 | 0.04 | -0.31 | 2.92E-12 | 1.95E-11 |
| MRM1 | WTAP | -7.20 | 0.04 | -0.32 | 2.48E-12 | 1.67E-11 |
| ADAT3 | TRUB1 | -7.24 | 0.04 | -0.32 | 1.81E-12 | 1.24E-11 |
| TET2 | METTL5 | -7.29 | 0.04 | -0.32 | 1.29E-12 | 9.00E-12 |
| FTSJ1 | TET2 | -7.38 | 0.04 | -0.32 | 7.31E-13 | 5.29E-12 |
| ADARB1 | TRMT10A | -7.41 | 0.04 | -0.32 | 5.87E-13 | 4.29E-12 |
| ADARB1 | METTL1 | -7.53 | 0.04 | -0.33 | 2.55E-13 | 1.91E-12 |
| RPUSD1 | TET2 | -7.54 | 0.04 | -0.33 | 2.41E-13 | 1.82E-12 |
| RPUSD1 | ZC3H13 | -7.55 | 0.04 | -0.33 | 2.34E-13 | 1.76E-12 |
| TRMT10B | YBX1 | -7.59 | 0.04 | -0.33 | 1.70E-13 | 1.31E-12 |
| ELP1 | TRMT112 | -7.61 | 0.04 | -0.33 | 1.55E-13 | 1.20E-12 |
| PUS1 | FTO | -7.61 | 0.04 | -0.33 | 1.53E-13 | 1.18E-12 |
| CTU1 | CMTR2 | -7.67 | 0.04 | -0.33 | 9.96E-14 | 7.87E-13 |
| TRMT112 | YTHDC2 | -7.67 | 0.04 | -0.33 | 9.71E-14 | 7.69E-13 |
| PUS10 | NOP2 | -7.70 | 0.04 | -0.34 | 8.15E-14 | 6.49E-13 |
| TRMT112 | EIF3A | -7.81 | 0.04 | -0.34 | 3.77E-14 | 3.12E-13 |
| METTL1 | TET2 | -7.84 | 0.04 | -0.34 | 3.01E-14 | 2.53E-13 |
| CTU2 | TET2 | -8.07 | 0.04 | -0.35 | 5.84E-15 | 5.33E-14 |
| TRMT112 | TET3 | -8.09 | 0.04 | -0.35 | 5.22E-15 | 4.80E-14 |
| ADARB1 | METTL5 | -8.27 | 0.04 | -0.36 | 1.44E-15 | 1.41E-14 |
| RPUSD1 | METTL14 | -8.30 | 0.04 | -0.36 | 1.10E-15 | 1.09E-14 |
| CTU2 | ZC3H13 | -8.45 | 0.04 | -0.36 | 3.73E-16 | 3.86E-15 |
| ADARB1 | TRMT112 | -8.73 | 0.04 | -0.37 | 4.55E-17 | 5.21E-16 |
| ADARB1 | TRMT10C | -8.79 | 0.04 | -0.38 | 2.93E-17 | 3.42E-16 |
| ADARB1 | HNRNPC | -8.96 | 0.04 | -0.38 | 7.64E-18 | 9.39E-17 |
| TRMT112 | TET2 | -9.78 | 0.04 | -0.41 | 1.13E-20 | 1.77E-19 |
| TRMT112 | ZC3H13 | -10.51 | 0.04 | -0.44 | 2.50E-23 | 4.80E-22 |

Table S6. The RNA modification pattern and RMScore of LUAD patients.

| SampleID | RNA Modification Cluster | RMScore |
| --- | --- | --- |
| TCGA-93-A4JO-01A | A | Low_Score |
| TCGA-91-6849-01A | A | Low_Score |
| TCGA-91-8499-01A | B | High_Score |
| TCGA-73-4677-01A | A | High_Score |
| TCGA-99-8032-01A | A | Low_Score |
| TCGA-91-A4BC-01A | A | High_Score |
| TCGA-55-8616-01A | A | High_Score |
| TCGA-NJ-A4YP-01A | A | High_Score |
| TCGA-L9-A5IP-01A | B | High_Score |
| TCGA-91-6830-01A | B | High_Score |
| TCGA-64-5775-01A | B | High_Score |
| TCGA-05-5715-01A | B | Low_Score |
| TCGA-MP-A4TC-01A | B | High_Score |
| TCGA-91-6835-01A | A | Low_Score |
| TCGA-MN-A4N5-01A | A | High_Score |
| TCGA-95-8494-01A | B | High_Score |
| TCGA-95-7562-01A | B | High_Score |
| TCGA-05-4415-01A | B | High_Score |
| TCGA-44-2666-01A | A | Low_Score |
| TCGA-55-A490-01A | B | High_Score |
| TCGA-86-A4D0-01A | B | High_Score |
| TCGA-55-6975-01A | B | High_Score |
| TCGA-55-7727-01A | A | High_Score |
| TCGA-50-6591-01A | B | High_Score |
| TCGA-05-4250-01A | B | High_Score |
| TCGA-86-8055-01A | B | High_Score |
| TCGA-69-8255-01A | B | High_Score |
| TCGA-73-4670-01A | B | High_Score |
| TCGA-38-A44F-01A | A | Low_Score |
| TCGA-69-7978-01A | B | High_Score |
| TCGA-86-8056-01A | A | Low_Score |
| TCGA-62-8394-01A | B | High_Score |
| TCGA-67-6216-01A | A | Low_Score |
| TCGA-05-5423-01A | B | Low_Score |
| TCGA-55-8092-01A | A | High_Score |
| TCGA-MP-A4T8-01A | A | High_Score |
| TCGA-97-8174-01A | A | High_Score |
| TCGA-MP-A4T7-01A | A | High_Score |
| TCGA-55-6712-01A | B | High_Score |
| TCGA-78-7146-01A | B | High_Score |
| TCGA-67-6215-01A | A | High_Score |
| TCGA-55-6978-01A | B | High_Score |
| TCGA-86-6851-01A | A | Low_Score |
| TCGA-78-7158-01A | A | Low_Score |
| TCGA-35-4123-01A | B | High_Score |
| TCGA-69-7974-01A | A | High_Score |
| TCGA-93-8067-01A | A | High_Score |
| TCGA-69-7761-01A | A | High_Score |
| TCGA-55-7914-01A | A | Low_Score |
| TCGA-50-6595-01A | B | High_Score |
| TCGA-L9-A443-01A | A | Low_Score |
| TCGA-69-7760-01A | B | High_Score |
| TCGA-71-8520-01A | B | Low_Score |
| TCGA-44-7662-01A | B | High_Score |
| TCGA-91-6848-01A | B | High_Score |
| TCGA-35-4122-01A | B | High_Score |
| TCGA-69-7973-01A | B | High_Score |
| TCGA-55-6979-01A | B | High_Score |
| TCGA-78-7536-01A | B | High_Score |
| TCGA-50-5072-01A | B | High_Score |
| TCGA-71-6725-01A | A | Low_Score |
| TCGA-50-5936-01A | B | High_Score |
| TCGA-78-7166-01A | B | High_Score |
| TCGA-49-AAR9-01A | B | High_Score |
| TCGA-44-8120-01A | A | Low_Score |
| TCGA-35-5375-01A | B | High_Score |
| TCGA-49-4507-01A | B | High_Score |
| TCGA-05-4418-01A | B | High_Score |
| TCGA-05-5429-01A | B | High_Score |
| TCGA-73-4676-01A | B | High_Score |
| TCGA-50-5930-01A | B | High_Score |
| TCGA-44-8119-01A | B | Low_Score |
| TCGA-44-A47B-01A | B | Low_Score |
| TCGA-78-7161-01A | A | Low_Score |
| TCGA-4B-A93V-01A | A | Low_Score |
| TCGA-05-4396-01A | A | Low_Score |
| TCGA-L9-A444-01A | A | Low_Score |
| TCGA-MP-A4TD-01A | A | Low_Score |
| TCGA-50-7109-01A | A | High_Score |
| TCGA-91-6831-01A | B | High_Score |
| TCGA-78-8660-01A | B | High_Score |
| TCGA-78-7542-01A | B | High_Score |
| TCGA-91-6828-01A | A | Low_Score |
| TCGA-50-6593-01A | B | High_Score |
| TCGA-MP-A4TF-01A | B | High_Score |
| TCGA-MP-A4TJ-01A | A | Low_Score |
| TCGA-73-A9RS-01A | A | High_Score |
| TCGA-55-7907-01A | B | High_Score |
| TCGA-44-A47G-01A | A | High_Score |
| TCGA-86-8585-01A | A | High_Score |
| TCGA-38-4631-01A | B | High_Score |
| TCGA-49-6761-01A | B | High_Score |
| TCGA-05-4422-01A | A | Low_Score |
| TCGA-50-6594-01A | B | High_Score |
| TCGA-91-6840-01A | B | Low_Score |
| TCGA-55-8620-01A | B | High_Score |
| TCGA-86-6562-01A | A | High_Score |
| TCGA-95-7944-01A | B | High_Score |
| TCGA-49-4490-01A | B | High_Score |
| TCGA-44-8117-01A | A | High_Score |
| TCGA-67-3774-01A | A | Low_Score |
| TCGA-69-7979-01A | A | High_Score |
| TCGA-44-A4SU-01A | A | Low_Score |
| TCGA-69-7980-01A | B | Low_Score |
| TCGA-62-A46Y-01A | A | High_Score |
| TCGA-69-7764-01A | A | Low_Score |
| TCGA-86-A4P7-01A | A | Low_Score |
| TCGA-44-A4SS-01A | B | High_Score |
| TCGA-55-8619-01A | A | Low_Score |
| TCGA-91-6836-01A | B | High_Score |
| TCGA-55-8507-01A | B | Low_Score |
| TCGA-67-6217-01A | A | Low_Score |
| TCGA-86-8668-01A | A | Low_Score |
| TCGA-53-7813-01A | A | Low_Score |
| TCGA-69-8253-01A | A | High_Score |
| TCGA-05-4384-01A | A | Low_Score |
| TCGA-67-3773-01A | B | Low_Score |
| TCGA-49-4505-01A | B | Low_Score |
| TCGA-MP-A4TI-01A | A | High_Score |
| TCGA-50-5931-01A | B | High_Score |
| TCGA-91-8497-01A | A | Low_Score |
| TCGA-L4-A4E6-01A | A | Low_Score |
| TCGA-55-6543-01A | A | High_Score |
| TCGA-97-8179-01A | A | High_Score |
| TCGA-55-8505-01A | B | High_Score |
| TCGA-55-A4DF-01A | B | High_Score |
| TCGA-55-7725-01A | A | Low_Score |
| TCGA-L9-A50W-01A | A | Low_Score |
| TCGA-86-8359-01A | B | High_Score |
| TCGA-62-8398-01A | B | High_Score |
| TCGA-55-8615-01A | A | Low_Score |
| TCGA-38-6178-01A | B | High_Score |
| TCGA-05-4417-01A | B | Low_Score |
| TCGA-05-5420-01A | B | High_Score |
| TCGA-05-4434-01A | B | High_Score |
| TCGA-50-5939-01A | B | High_Score |
| TCGA-55-8087-01A | A | Low_Score |
| TCGA-55-6970-01A | A | High_Score |
| TCGA-44-A47A-01A | A | High_Score |
| TCGA-73-4668-01A | B | High_Score |
| TCGA-97-8176-01A | B | High_Score |
| TCGA-55-8299-01A | A | High_Score |
| TCGA-L9-A8F4-01A | A | High_Score |
| TCGA-95-7948-01A | A | Low_Score |
| TCGA-55-8097-01A | A | Low_Score |
| TCGA-95-7947-01A | A | High_Score |
| TCGA-55-8302-01A | B | High_Score |
| TCGA-50-5051-01A | B | Low_Score |
| TCGA-55-A494-01A | A | High_Score |
| TCGA-97-7941-01A | A | Low_Score |
| TCGA-44-A479-01A | B | Low_Score |
| TCGA-55-7573-01A | A | Low_Score |
| TCGA-49-6742-01A | B | Low_Score |
| TCGA-91-7771-01A | A | Low_Score |
| TCGA-97-8177-01A | B | Low_Score |
| TCGA-44-6779-01A | B | High_Score |
| TCGA-95-7043-01A | B | Low_Score |
| TCGA-91-8496-01A | B | Low_Score |
| TCGA-S2-AA1A-01A | A | Low_Score |
| TCGA-55-8621-01A | A | Low_Score |
| TCGA-55-8204-01A | B | High_Score |
| TCGA-55-8514-01A | A | Low_Score |
| TCGA-49-6745-01A | B | High_Score |
| TCGA-93-A4JQ-01A | B | High_Score |
| TCGA-93-7348-01A | A | Low_Score |
| TCGA-55-8301-01A | B | Low_Score |
| TCGA-55-8614-01A | B | High_Score |
| TCGA-55-7911-01A | A | High_Score |
| TCGA-55-8510-01A | A | Low_Score |
| TCGA-97-A4M3-01A | A | Low_Score |
| TCGA-55-8094-01A | B | Low_Score |
| TCGA-97-8172-01A | A | Low_Score |
| TCGA-55-A57B-01A | A | Low_Score |
| TCGA-55-8203-01A | A | High_Score |
| TCGA-J2-A4AD-01A | A | High_Score |
| TCGA-97-8175-01A | B | High_Score |
| TCGA-55-8511-01A | B | High_Score |
| TCGA-95-A4VN-01A | A | High_Score |
| TCGA-44-7661-01A | A | High_Score |
| TCGA-97-7937-01A | A | High_Score |
| TCGA-L9-A7SV-01A | B | High_Score |
| TCGA-55-7903-01A | A | High_Score |
| TCGA-97-A4M6-01A | A | Low_Score |
| TCGA-97-8171-01A | A | Low_Score |
| TCGA-95-7567-01A | B | High_Score |
| TCGA-67-3772-01A | B | Low_Score |
| TCGA-44-7669-01A | B | Low_Score |
| TCGA-05-4403-01A | B | High_Score |
| TCGA-L4-A4E5-01A | B | High_Score |
| TCGA-MP-A4TK-01A | B | High_Score |
| TCGA-78-7147-01A | B | High_Score |
| TCGA-69-A59K-01A | B | Low_Score |
| TCGA-44-7660-01A | B | High_Score |
| TCGA-78-7154-01A | B | High_Score |
| TCGA-62-A46P-01A | B | Low_Score |
| TCGA-44-6145-01A | A | High_Score |
| TCGA-55-A492-01A | B | Low_Score |
| TCGA-55-8090-01A | A | High_Score |
| TCGA-55-8205-01A | B | High_Score |
| TCGA-55-8091-01A | A | Low_Score |
| TCGA-97-A4M1-01A | A | Low_Score |
| TCGA-NJ-A55R-01A | A | Low_Score |
| TCGA-55-7994-01A | B | High_Score |
| TCGA-91-A4BD-01A | B | Low_Score |
| TCGA-86-7954-01A | B | Low_Score |
| TCGA-95-A4VP-01A | A | Low_Score |
| TCGA-55-8512-01A | A | Low_Score |
| TCGA-05-4382-01A | B | High_Score |
| TCGA-55-A4DG-01A | A | Low_Score |
| TCGA-55-7283-01A | A | Low_Score |
| TCGA-05-4405-01A | A | Low_Score |
| TCGA-67-3771-01A | B | High_Score |
| TCGA-67-3770-01A | B | Low_Score |
| TCGA-97-A4LX-01A | A | Low_Score |
| TCGA-NJ-A7XG-01A | A | Low_Score |
| TCGA-55-8508-01A | A | High_Score |
| TCGA-97-A4M2-01A | A | Low_Score |
| TCGA-50-5044-01A | B | High_Score |
| TCGA-86-7714-01A | A | Low_Score |
| TCGA-78-7148-01A | B | High_Score |
| TCGA-55-A491-01A | B | Low_Score |
| TCGA-97-8552-01A | A | Low_Score |
| TCGA-64-1677-01A | B | High_Score |
| TCGA-97-A4M7-01A | A | Low_Score |
| TCGA-55-A48Y-01A | B | High_Score |
| TCGA-97-A4M5-01A | A | Low_Score |
| TCGA-55-A48Z-01A | A | High_Score |
| TCGA-95-A4VK-01A | A | High_Score |
| TCGA-55-7726-01A | B | High_Score |
| TCGA-97-A4M0-01A | A | Low_Score |
| TCGA-50-5935-01A | A | Low_Score |
| TCGA-86-8358-01A | B | High_Score |
| TCGA-97-8547-01A | A | Low_Score |
| TCGA-44-6774-01A | A | Low_Score |
| TCGA-99-AA5R-01A | A | Low_Score |
| TCGA-L9-A743-01A | A | High_Score |
| TCGA-78-7150-01A | B | High_Score |
| TCGA-05-4425-01A | B | High_Score |
| TCGA-55-7576-01A | B | Low_Score |
| TCGA-05-5428-01A | B | Low_Score |
| TCGA-55-8208-01A | B | Low_Score |
| TCGA-49-AAQV-01A | A | High_Score |
| TCGA-49-6767-01A | B | High_Score |
| TCGA-93-7347-01A | A | Low_Score |
| TCGA-55-A48X-01A | A | Low_Score |
| TCGA-69-7763-01A | A | Low_Score |
| TCGA-44-7659-01A | A | Low_Score |
| TCGA-86-8075-01A | B | High_Score |
| TCGA-78-7160-01A | A | High_Score |
| TCGA-86-8280-01A | A | Low_Score |
| TCGA-55-1592-01A | B | Low_Score |
| TCGA-55-8089-01A | B | High_Score |
| TCGA-44-6148-01A | A | Low_Score |
| TCGA-55-7728-01A | A | Low_Score |
| TCGA-44-6775-01A | A | High_Score |
| TCGA-55-7724-01A | B | High_Score |
| TCGA-73-4659-01A | B | Low_Score |
| TCGA-93-A4JN-01A | A | Low_Score |
| TCGA-44-7672-01A | B | High_Score |
| TCGA-55-8096-01A | B | Low_Score |
| TCGA-J2-8194-01A | A | Low_Score |
| TCGA-44-6146-01A | A | Low_Score |
| TCGA-05-4433-01A | A | High_Score |
| TCGA-05-4397-01A | B | Low_Score |
| TCGA-86-A4JF-01A | B | High_Score |
| TCGA-J2-8192-01A | A | High_Score |
| TCGA-86-8073-01A | A | Low_Score |
| TCGA-MP-A4TH-01A | A | Low_Score |
| TCGA-99-7458-01A | A | Low_Score |
| TCGA-55-6984-01A | B | High_Score |
| TCGA-05-4432-01A | B | Low_Score |
| TCGA-44-2668-01A | B | High_Score |
| TCGA-05-4430-01A | B | Low_Score |
| TCGA-55-7815-01A | A | Low_Score |
| TCGA-97-7554-01A | A | High_Score |
| TCGA-50-6592-01A | B | High_Score |
| TCGA-05-4426-01A | A | Low_Score |
| TCGA-55-8513-01A | A | Low_Score |
| TCGA-78-7539-01A | A | Low_Score |
| TCGA-05-4427-01A | A | High_Score |
| TCGA-38-7271-01A | A | Low_Score |
| TCGA-73-4666-01A | B | High_Score |
| TCGA-86-A4P8-01A | A | Low_Score |
| TCGA-86-8674-01A | B | Low_Score |
| TCGA-78-7220-01A | B | High_Score |
| TCGA-44-4112-01A | B | High_Score |
| TCGA-69-8453-01A | B | High_Score |
| TCGA-83-5908-01A | B | High_Score |
| TCGA-55-7570-01A | B | High_Score |
| TCGA-78-7145-01A | B | High_Score |
| TCGA-MN-A4N1-01A | B | High_Score |
| TCGA-50-8460-01A | A | Low_Score |
| TCGA-95-8039-01A | A | Low_Score |
| TCGA-86-8671-01A | A | Low_Score |
| TCGA-91-6847-01A | B | High_Score |
| TCGA-44-6147-01A | A | Low_Score |
| TCGA-44-5645-01A | A | Low_Score |
| TCGA-49-4487-01A | B | Low_Score |
| TCGA-86-8673-01A | B | High_Score |
| TCGA-44-5644-01A | B | High_Score |
| TCGA-64-5779-01A | B | Low_Score |
| TCGA-38-4629-01A | B | High_Score |
| TCGA-64-5815-01A | B | High_Score |
| TCGA-49-4488-01A | B | High_Score |
| TCGA-55-7281-01A | B | Low_Score |
| TCGA-49-AAR4-01A | A | Low_Score |
| TCGA-44-7670-01A | B | High_Score |
| TCGA-05-5425-01A | B | High_Score |
| TCGA-55-8206-01A | A | Low_Score |
| TCGA-44-7671-01A | A | High_Score |
| TCGA-55-7995-01A | A | Low_Score |
| TCGA-49-4510-01A | A | Low_Score |
| TCGA-MP-A4TE-01A | B | Low_Score |
| TCGA-86-A456-01A | A | Low_Score |
| TCGA-55-8085-01A | A | Low_Score |
| TCGA-49-4512-01A | B | Low_Score |
| TCGA-62-A472-01A | A | High_Score |
| TCGA-05-4420-01A | B | High_Score |
| TCGA-05-4424-01A | B | Low_Score |
| TCGA-73-4675-01A | B | High_Score |
| TCGA-53-7626-01A | A | Low_Score |
| TCGA-55-5899-01A | B | High_Score |
| TCGA-86-8669-01A | A | Low_Score |
| TCGA-86-8278-01A | A | Low_Score |
| TCGA-86-7701-01A | A | High_Score |
| TCGA-86-8279-01A | A | High_Score |
| TCGA-78-7535-01A | B | High_Score |
| TCGA-MP-A4TA-01A | B | High_Score |
| TCGA-55-7227-01A | A | High_Score |
| TCGA-78-7156-01A | A | Low_Score |
| TCGA-55-8207-01A | A | Low_Score |
| TCGA-44-6777-01A | A | Low_Score |
| TCGA-J2-A4AG-01A | A | Low_Score |
| TCGA-86-8076-01A | A | Low_Score |
| TCGA-55-7574-01A | A | Low_Score |
| TCGA-55-6982-01A | B | High_Score |
| TCGA-86-7953-01A | B | High_Score |
| TCGA-49-4506-01A | B | High_Score |
| TCGA-44-5643-01A | B | High_Score |
| TCGA-44-3919-01A | A | Low_Score |
| TCGA-44-3918-01A | A | Low_Score |
| TCGA-55-7910-01A | B | High_Score |
| TCGA-53-7624-01A | B | High_Score |
| TCGA-86-7711-01A | B | High_Score |
| TCGA-99-8025-01A | B | Low_Score |
| TCGA-53-A4EZ-01A | B | High_Score |
| TCGA-86-7955-01A | B | High_Score |
| TCGA-38-4630-01A | B | Low_Score |
| TCGA-J2-A4AE-01A | A | Low_Score |
| TCGA-49-4494-01A | B | High_Score |
| TCGA-44-7667-01A | B | High_Score |
| TCGA-99-8028-01A | B | Low_Score |
| TCGA-50-8459-01A | A | High_Score |
| TCGA-50-8457-01A | A | Low_Score |
| TCGA-64-1680-01A | B | High_Score |
| TCGA-05-4390-01A | B | High_Score |
| TCGA-44-3396-01A | B | High_Score |
| TCGA-49-AARN-01A | A | High_Score |
| TCGA-38-4627-01A | B | High_Score |
| TCGA-86-8054-01A | B | High_Score |
| TCGA-86-7713-01A | B | High_Score |
| TCGA-44-2661-01A | B | Low_Score |
| TCGA-44-3398-01A | B | High_Score |
| TCGA-64-1681-01A | B | Low_Score |
| TCGA-78-7155-01A | B | High_Score |
| TCGA-MN-A4N4-01A | A | High_Score |
| TCGA-55-1594-01A | B | Low_Score |
| TCGA-44-3917-01A | A | Low_Score |
| TCGA-73-7498-01A | A | Low_Score |
| TCGA-64-1678-01A | B | Low_Score |
| TCGA-62-A470-01A | A | Low_Score |
| TCGA-78-7540-01A | B | High_Score |
| TCGA-78-8648-01A | A | Low_Score |
| TCGA-78-7152-01A | A | Low_Score |
| TCGA-62-8395-01A | A | Low_Score |
| TCGA-49-AARE-01A | B | High_Score |
| TCGA-55-6985-01A | B | High_Score |
| TCGA-50-5932-01A | A | Low_Score |
| TCGA-62-A471-01A | B | High_Score |
| TCGA-91-6829-01A | B | Low_Score |
| TCGA-MP-A4T9-01A | B | High_Score |
| TCGA-50-6597-01A | B | Low_Score |
| TCGA-95-7039-01A | B | Low_Score |
| TCGA-44-2662-01A | B | High_Score |
| TCGA-97-7546-01A | A | Low_Score |
| TCGA-50-6590-01A | B | High_Score |
| TCGA-62-8397-01A | A | Low_Score |
| TCGA-55-6968-01A | B | High_Score |
| TCGA-44-2665-01A | A | High_Score |
| TCGA-64-5778-01A | B | Low_Score |
| TCGA-44-2655-01A | A | Low_Score |
| TCGA-44-2657-01A | B | Low_Score |
| TCGA-38-4632-01A | B | High_Score |
| TCGA-44-2659-01A | A | Low_Score |
| TCGA-05-4389-01A | B | Low_Score |
| TCGA-55-6981-01A | B | High_Score |
| TCGA-55-6971-01A | A | Low_Score |
| TCGA-49-4501-01A | B | Low_Score |
| TCGA-44-2656-01A | A | High_Score |
| TCGA-05-4398-01A | B | High_Score |
| TCGA-NJ-A4YQ-01A | A | Low_Score |
| TCGA-50-5066-01A | B | High_Score |
| TCGA-62-A46O-01A | B | High_Score |
| TCGA-50-5941-01A | B | High_Score |
| TCGA-38-4628-01A | B | Low_Score |
| TCGA-62-8402-01A | B | High_Score |
| TCGA-50-5068-01A | B | Low_Score |
| TCGA-MP-A4SY-01A | A | High_Score |
| TCGA-05-4249-01A | A | Low_Score |
| TCGA-78-7633-01A | A | Low_Score |
| TCGA-73-7499-01A | B | High_Score |
| TCGA-64-5781-01A | B | High_Score |
| TCGA-73-4658-01A | B | Low_Score |
| TCGA-50-5946-01A | B | Low_Score |
| TCGA-49-6743-01A | B | High_Score |
| TCGA-78-7537-01A | A | Low_Score |
| TCGA-55-6972-01A | B | Low_Score |
| TCGA-62-A46S-01A | A | Low_Score |
| TCGA-49-6744-01A | A | High_Score |
| TCGA-49-4514-01A | B | Low_Score |
| TCGA-62-A46R-01A | A | High_Score |
| TCGA-64-1676-01A | B | Low_Score |
| TCGA-50-5944-01A | A | Low_Score |
| TCGA-MP-A4SW-01A | A | Low_Score |
| TCGA-MP-A4T6-01A | A | Low_Score |
| TCGA-O1-A52J-01A | A | Low_Score |
| TCGA-50-5055-01A | B | Low_Score |
| TCGA-50-5942-01A | A | Low_Score |
| TCGA-44-6778-01A | A | Low_Score |
| TCGA-97-7553-01A | A | Low_Score |
| TCGA-49-AAR3-01A | B | High_Score |
| TCGA-97-7552-01A | A | Low_Score |
| TCGA-97-7547-01A | A | Low_Score |
| TCGA-78-7159-01A | A | Low_Score |
| TCGA-55-1596-01A | B | High_Score |
| TCGA-55-6980-01A | A | Low_Score |
| TCGA-55-6987-01A | B | Low_Score |
| TCGA-NJ-A4YF-01A | A | Low_Score |
| TCGA-50-5045-01A | B | High_Score |
| TCGA-62-A46V-01A | A | Low_Score |
| TCGA-49-AAR2-01A | A | Low_Score |
| TCGA-MP-A5C7-01A | A | Low_Score |
| TCGA-NJ-A4YG-01A | A | Low_Score |
| TCGA-49-4486-01A | B | Low_Score |
| TCGA-78-8655-01A | B | Low_Score |
| TCGA-50-5933-01A | B | High_Score |
| TCGA-55-6642-01A | A | Low_Score |
| TCGA-64-1679-01A | B | High_Score |
| TCGA-73-4662-01A | A | Low_Score |
| TCGA-44-6776-01A | A | Low_Score |
| TCGA-MP-A4T4-01A | A | High_Score |
| TCGA-MP-A4SV-01A | B | High_Score |
| TCGA-64-5774-01A | B | Low_Score |
| TCGA-78-7167-01A | A | Low_Score |
| TCGA-62-8399-01A | B | High_Score |
| TCGA-55-6983-01A | A | Low_Score |
| TCGA-38-4625-01A | B | High_Score |
| TCGA-50-5049-01A | B | Low_Score |
| TCGA-78-7162-01A | A | Low_Score |
| TCGA-55-6986-01A | A | Low_Score |
| TCGA-78-8662-01A | B | High_Score |
| TCGA-78-7153-01A | B | Low_Score |
| TCGA-38-4626-01A | A | Low_Score |
| TCGA-49-AARO-01A | B | Low_Score |
| TCGA-78-7149-01A | A | Low_Score |
| TCGA-49-AAR0-01A | A | Low_Score |
| TCGA-78-7143-01A | B | High_Score |
| TCGA-49-AARR-01A | A | Low_Score |
| TCGA-49-AARQ-01A | A | Low_Score |
| TCGA-78-8640-01A | B | Low_Score |
| TCGA-78-7163-01A | B | Low_Score |

Table S7. Survival associated DEGs in univariate Cox regression analysis in TCGA-LUAD cohort.

| Gene.Name | coef | exp.coef | se.coef | z | pval | lower.95 | upper.95 | p.adj |
| --- | --- | --- | --- | --- | --- | --- | --- | --- |
| LINC00942 | 0.45 | 1.57 | 0.16 | 2.87 | 0.0040 | 1.15 | 2.13 | 0.0333 |
| INMT-MINDY4 | -0.44 | 0.64 | 0.16 | -2.82 | 0.0048 | 0.47 | 0.87 | 0.0363 |
| FGF12-AS2 | 0.42 | 1.52 | 0.16 | 2.69 | 0.0070 | 1.12 | 2.06 | 0.0455 |
| BANCR | -0.62 | 0.54 | 0.16 | -3.92 | 0.0001 | 0.40 | 0.73 | 0.0059 |
| LINC02802 | 0.56 | 1.76 | 0.16 | 3.57 | 0.0004 | 1.29 | 2.39 | 0.0094 |
| KIF20A | 0.46 | 1.58 | 0.16 | 2.93 | 0.0034 | 1.16 | 2.14 | 0.0297 |
| LINC01843 | 0.50 | 1.66 | 0.16 | 3.23 | 0.0012 | 1.22 | 2.25 | 0.0166 |
| LOC101929128 | 0.50 | 1.65 | 0.16 | 3.19 | 0.0014 | 1.21 | 2.24 | 0.0175 |
| LOC101929237 | -0.54 | 0.58 | 0.16 | -3.45 | 0.0006 | 0.43 | 0.79 | 0.0111 |
| MIR6503 | -0.48 | 0.62 | 0.16 | -3.05 | 0.0023 | 0.46 | 0.84 | 0.0233 |
| LINC01117 | 0.52 | 1.67 | 0.16 | 3.28 | 0.0010 | 1.23 | 2.28 | 0.0152 |
| CDKN3 | 0.58 | 1.79 | 0.16 | 3.65 | 0.0003 | 1.31 | 2.44 | 0.0087 |
| ABCA8 | -0.43 | 0.65 | 0.16 | -2.78 | 0.0054 | 0.48 | 0.88 | 0.0384 |
| SPAG5 | 0.61 | 1.84 | 0.16 | 3.89 | 0.0001 | 1.35 | 2.50 | 0.0059 |
| CENPE | 0.51 | 1.66 | 0.16 | 3.24 | 0.0012 | 1.22 | 2.25 | 0.0164 |
| RAD51AP1 | 0.42 | 1.52 | 0.16 | 2.68 | 0.0074 | 1.12 | 2.06 | 0.0468 |
| IGF2BP3 | 0.43 | 1.54 | 0.16 | 2.79 | 0.0052 | 1.14 | 2.09 | 0.0382 |
| MTHFD2 | 0.49 | 1.64 | 0.16 | 3.15 | 0.0016 | 1.21 | 2.23 | 0.0187 |
| LINC01833 | 0.55 | 1.73 | 0.16 | 3.51 | 0.0004 | 1.27 | 2.35 | 0.0100 |
| CHAD | -0.45 | 0.64 | 0.16 | -2.84 | 0.0045 | 0.47 | 0.87 | 0.0353 |
| CHEK1 | 0.44 | 1.56 | 0.16 | 2.84 | 0.0045 | 1.15 | 2.11 | 0.0357 |
| ZWINT | 0.43 | 1.54 | 0.16 | 2.79 | 0.0053 | 1.14 | 2.10 | 0.0382 |
| MTFR2 | 0.49 | 1.63 | 0.16 | 3.14 | 0.0017 | 1.20 | 2.21 | 0.0191 |
| CDCA5 | 0.53 | 1.70 | 0.16 | 3.40 | 0.0007 | 1.25 | 2.31 | 0.0126 |
| OIP5 | 0.55 | 1.74 | 0.16 | 3.51 | 0.0004 | 1.28 | 2.37 | 0.0100 |
| FCRL1 | -0.53 | 0.59 | 0.16 | -3.39 | 0.0007 | 0.43 | 0.80 | 0.0126 |
| CKS1B | 0.47 | 1.59 | 0.16 | 2.99 | 0.0028 | 1.17 | 2.17 | 0.0267 |
| CLCN1 | 0.50 | 1.64 | 0.16 | 3.17 | 0.0015 | 1.21 | 2.23 | 0.0180 |
| ADH1A | -0.44 | 0.64 | 0.16 | -2.82 | 0.0048 | 0.47 | 0.87 | 0.0363 |
| ADM | 0.48 | 1.62 | 0.16 | 3.07 | 0.0021 | 1.19 | 2.19 | 0.0224 |
| ADHFE1 | -0.49 | 0.61 | 0.16 | -3.13 | 0.0017 | 0.45 | 0.83 | 0.0193 |
| BEST3 | 0.44 | 1.56 | 0.16 | 2.85 | 0.0044 | 1.15 | 2.11 | 0.0349 |
| E2F7 | 0.58 | 1.79 | 0.16 | 3.74 | 0.0002 | 1.32 | 2.44 | 0.0080 |
| C16orf89 | -0.41 | 0.66 | 0.15 | -2.68 | 0.0074 | 0.49 | 0.89 | 0.0468 |
| KIF18B | 0.57 | 1.77 | 0.16 | 3.65 | 0.0003 | 1.30 | 2.41 | 0.0087 |
| SPC24 | 0.44 | 1.55 | 0.16 | 2.84 | 0.0046 | 1.15 | 2.11 | 0.0358 |
| CHIAP2 | -0.55 | 0.58 | 0.16 | -3.50 | 0.0005 | 0.43 | 0.79 | 0.0102 |
| SGO2 | 0.47 | 1.60 | 0.16 | 3.03 | 0.0025 | 1.18 | 2.18 | 0.0243 |
| CTSV | 0.59 | 1.81 | 0.16 | 3.76 | 0.0002 | 1.33 | 2.46 | 0.0080 |
| SGO1 | 0.53 | 1.70 | 0.16 | 3.38 | 0.0007 | 1.25 | 2.30 | 0.0126 |
| RAET1L | 0.46 | 1.58 | 0.16 | 2.94 | 0.0033 | 1.17 | 2.15 | 0.0294 |
| LOC157273 | 0.50 | 1.65 | 0.16 | 3.19 | 0.0014 | 1.21 | 2.24 | 0.0175 |
| CDCA2 | 0.52 | 1.68 | 0.16 | 3.34 | 0.0009 | 1.24 | 2.29 | 0.0131 |
| ESCO2 | 0.41 | 1.51 | 0.15 | 2.66 | 0.0078 | 1.11 | 2.05 | 0.0477 |
| CYP4A11 | -0.41 | 0.66 | 0.16 | -2.66 | 0.0078 | 0.49 | 0.90 | 0.0477 |
| CYP4B1 | -0.52 | 0.59 | 0.16 | -3.33 | 0.0009 | 0.44 | 0.81 | 0.0131 |
| TTC16 | -0.43 | 0.65 | 0.16 | -2.78 | 0.0055 | 0.48 | 0.88 | 0.0387 |
| CYP17A1 | -0.62 | 0.54 | 0.16 | -3.95 | 0.0001 | 0.39 | 0.73 | 0.0059 |
| CLEC4F | -0.44 | 0.65 | 0.16 | -2.79 | 0.0052 | 0.48 | 0.88 | 0.0382 |
| RHOV | 0.42 | 1.52 | 0.16 | 2.67 | 0.0077 | 1.12 | 2.06 | 0.0477 |
| AGER | -0.49 | 0.61 | 0.16 | -3.10 | 0.0019 | 0.45 | 0.84 | 0.0210 |
| ECT2 | 0.50 | 1.65 | 0.16 | 3.22 | 0.0013 | 1.22 | 2.24 | 0.0167 |
| NIBAN3 | -0.48 | 0.62 | 0.16 | -3.03 | 0.0024 | 0.46 | 0.84 | 0.0242 |
| CYP4Z1 | -0.48 | 0.62 | 0.16 | -3.05 | 0.0023 | 0.46 | 0.84 | 0.0235 |
| CFAP221 | -0.45 | 0.64 | 0.16 | -2.87 | 0.0041 | 0.47 | 0.87 | 0.0333 |
| CREG2 | 0.44 | 1.55 | 0.16 | 2.83 | 0.0046 | 1.15 | 2.11 | 0.0358 |
| ZSCAN4 | -0.45 | 0.64 | 0.16 | -2.86 | 0.0043 | 0.47 | 0.87 | 0.0345 |
| MS4A15 | -0.47 | 0.62 | 0.16 | -3.02 | 0.0025 | 0.46 | 0.85 | 0.0246 |
| SKA3 | 0.54 | 1.72 | 0.16 | 3.44 | 0.0006 | 1.26 | 2.34 | 0.0113 |
| ADGRF5 | -0.45 | 0.64 | 0.16 | -2.91 | 0.0036 | 0.47 | 0.86 | 0.0311 |
| DKK1 | 0.66 | 1.94 | 0.16 | 4.17 | 0.0000 | 1.42 | 2.65 | 0.0052 |
| TPX2 | 0.50 | 1.64 | 0.16 | 3.17 | 0.0015 | 1.21 | 2.23 | 0.0180 |
| FOXM1 | 0.53 | 1.69 | 0.16 | 3.37 | 0.0007 | 1.25 | 2.30 | 0.0127 |
| TNFRSF13B | -0.49 | 0.61 | 0.16 | -3.15 | 0.0016 | 0.45 | 0.83 | 0.0187 |
| KIF4A | 0.45 | 1.56 | 0.16 | 2.88 | 0.0040 | 1.15 | 2.12 | 0.0333 |
| C11orf86 | 0.43 | 1.54 | 0.16 | 2.78 | 0.0054 | 1.14 | 2.09 | 0.0384 |
| HMCN2 | -0.44 | 0.65 | 0.16 | -2.81 | 0.0050 | 0.48 | 0.88 | 0.0371 |
| GAP43 | 0.42 | 1.53 | 0.16 | 2.72 | 0.0065 | 1.13 | 2.08 | 0.0430 |
| DNAH1 | -0.45 | 0.63 | 0.16 | -2.87 | 0.0041 | 0.47 | 0.87 | 0.0333 |
| GDF10 | -0.45 | 0.64 | 0.16 | -2.87 | 0.0041 | 0.47 | 0.87 | 0.0333 |
| STEAP1 | 0.67 | 1.95 | 0.16 | 4.21 | 0.0000 | 1.43 | 2.66 | 0.0052 |
| GJB2 | 0.55 | 1.73 | 0.16 | 3.47 | 0.0005 | 1.27 | 2.36 | 0.0111 |
| GJB3 | 0.57 | 1.77 | 0.16 | 3.64 | 0.0003 | 1.30 | 2.40 | 0.0087 |
| CPAMD8 | -0.42 | 0.66 | 0.15 | -2.72 | 0.0065 | 0.48 | 0.89 | 0.0430 |
| CHIA | -0.42 | 0.66 | 0.16 | -2.71 | 0.0067 | 0.48 | 0.89 | 0.0437 |
| UBE2S | 0.46 | 1.59 | 0.16 | 2.97 | 0.0030 | 1.17 | 2.16 | 0.0279 |
| GNG4 | 0.44 | 1.55 | 0.16 | 2.83 | 0.0046 | 1.15 | 2.11 | 0.0358 |
| ADGRD1 | -0.60 | 0.55 | 0.16 | -3.83 | 0.0001 | 0.40 | 0.75 | 0.0068 |
| LINC01559 | 0.51 | 1.67 | 0.16 | 3.27 | 0.0011 | 1.23 | 2.26 | 0.0156 |
| SLC13A5 | 0.46 | 1.58 | 0.16 | 2.95 | 0.0032 | 1.17 | 2.15 | 0.0292 |
| RSPO1 | -0.49 | 0.61 | 0.16 | -3.12 | 0.0018 | 0.45 | 0.83 | 0.0202 |
| KRT6C | 0.70 | 2.01 | 0.16 | 4.40 | 0.0000 | 1.47 | 2.74 | 0.0036 |
| UBE2T | 0.53 | 1.70 | 0.16 | 3.36 | 0.0008 | 1.25 | 2.31 | 0.0129 |
| RACGAP1 | 0.46 | 1.59 | 0.16 | 3.00 | 0.0027 | 1.17 | 2.16 | 0.0264 |
| HBQ1 | 0.42 | 1.52 | 0.16 | 2.69 | 0.0072 | 1.12 | 2.06 | 0.0462 |
| HLF | -0.54 | 0.58 | 0.16 | -3.44 | 0.0006 | 0.43 | 0.79 | 0.0113 |
| HMGA1 | 0.54 | 1.71 | 0.16 | 3.43 | 0.0006 | 1.26 | 2.32 | 0.0113 |
| HMMR | 0.61 | 1.84 | 0.16 | 3.85 | 0.0001 | 1.35 | 2.50 | 0.0066 |
| HOXB7 | 0.46 | 1.58 | 0.16 | 2.94 | 0.0033 | 1.17 | 2.15 | 0.0293 |
| HOXB9 | 0.50 | 1.65 | 0.16 | 3.22 | 0.0013 | 1.22 | 2.24 | 0.0168 |
| BIRC5 | 0.55 | 1.74 | 0.16 | 3.51 | 0.0004 | 1.28 | 2.37 | 0.0100 |
| HTR1D | 0.46 | 1.59 | 0.16 | 2.97 | 0.0029 | 1.17 | 2.16 | 0.0279 |
| GPIHBP1 | -0.42 | 0.66 | 0.16 | -2.72 | 0.0065 | 0.48 | 0.89 | 0.0430 |
| IGFBP1 | 0.45 | 1.57 | 0.16 | 2.89 | 0.0038 | 1.16 | 2.14 | 0.0325 |
| FAM131C | 0.41 | 1.51 | 0.16 | 2.65 | 0.0081 | 1.11 | 2.04 | 0.0485 |
| IL11 | 0.50 | 1.65 | 0.16 | 3.20 | 0.0014 | 1.21 | 2.24 | 0.0171 |
| PTPRQ | -0.49 | 0.61 | 0.16 | -3.11 | 0.0019 | 0.45 | 0.83 | 0.0206 |
| TEPP | -0.52 | 0.59 | 0.16 | -3.35 | 0.0008 | 0.44 | 0.80 | 0.0129 |
| KCNF1 | 0.60 | 1.83 | 0.16 | 3.82 | 0.0001 | 1.34 | 2.50 | 0.0068 |
| SLC26A5 | -0.41 | 0.66 | 0.16 | -2.64 | 0.0084 | 0.49 | 0.90 | 0.0493 |
| KIF11 | 0.54 | 1.71 | 0.16 | 3.46 | 0.0005 | 1.26 | 2.33 | 0.0111 |
| KPNA2 | 0.52 | 1.69 | 0.16 | 3.34 | 0.0008 | 1.24 | 2.29 | 0.0131 |
| KRT6A | 0.41 | 1.51 | 0.15 | 2.65 | 0.0079 | 1.11 | 2.04 | 0.0481 |
| KRT16 | 0.49 | 1.64 | 0.16 | 3.15 | 0.0016 | 1.20 | 2.23 | 0.0187 |
| CENPW | 0.44 | 1.55 | 0.16 | 2.81 | 0.0049 | 1.14 | 2.11 | 0.0371 |
| KRT81 | 0.47 | 1.59 | 0.16 | 2.95 | 0.0032 | 1.17 | 2.17 | 0.0292 |
| LAMC2 | 0.45 | 1.57 | 0.16 | 2.91 | 0.0036 | 1.16 | 2.13 | 0.0311 |
| RPL13AP17 | -0.45 | 0.64 | 0.16 | -2.83 | 0.0047 | 0.47 | 0.87 | 0.0360 |
| SH2D5 | 0.44 | 1.56 | 0.16 | 2.85 | 0.0044 | 1.15 | 2.11 | 0.0349 |
| RAB44 | -0.42 | 0.66 | 0.16 | -2.69 | 0.0072 | 0.48 | 0.89 | 0.0462 |
| LOC401478 | -0.44 | 0.64 | 0.16 | -2.79 | 0.0053 | 0.47 | 0.88 | 0.0382 |
| SNX30 | -0.55 | 0.58 | 0.16 | -3.46 | 0.0005 | 0.42 | 0.79 | 0.0111 |
| C16orf74 | 0.42 | 1.52 | 0.16 | 2.67 | 0.0075 | 1.12 | 2.06 | 0.0470 |
| MIR186 | -0.45 | 0.64 | 0.16 | -2.76 | 0.0058 | 0.46 | 0.88 | 0.0401 |
| MIR23A | -0.48 | 0.62 | 0.16 | -3.08 | 0.0021 | 0.45 | 0.84 | 0.0221 |
| MIR23B | -0.48 | 0.62 | 0.16 | -3.08 | 0.0021 | 0.45 | 0.84 | 0.0223 |
| MAD2L1 | 0.53 | 1.69 | 0.16 | 3.36 | 0.0008 | 1.24 | 2.30 | 0.0129 |
| LCN10 | -0.48 | 0.62 | 0.16 | -3.06 | 0.0022 | 0.45 | 0.84 | 0.0227 |
| MFAP4 | -0.41 | 0.66 | 0.16 | -2.64 | 0.0084 | 0.49 | 0.90 | 0.0493 |
| MKI67 | 0.58 | 1.78 | 0.16 | 3.68 | 0.0002 | 1.31 | 2.41 | 0.0083 |
| PLIN5 | -0.57 | 0.56 | 0.16 | -3.59 | 0.0003 | 0.41 | 0.77 | 0.0092 |
| PRDM16-DT | -0.63 | 0.53 | 0.16 | -3.95 | 0.0001 | 0.39 | 0.73 | 0.0059 |
| MUSK | -0.56 | 0.57 | 0.16 | -3.53 | 0.0004 | 0.42 | 0.78 | 0.0098 |
| ASTN1 | -0.45 | 0.64 | 0.16 | -2.88 | 0.0040 | 0.47 | 0.87 | 0.0333 |
| NEK2 | 0.58 | 1.79 | 0.16 | 3.69 | 0.0002 | 1.31 | 2.43 | 0.0083 |
| NFIX | -0.41 | 0.66 | 0.16 | -2.66 | 0.0078 | 0.49 | 0.90 | 0.0477 |
| NTSR1 | 0.42 | 1.53 | 0.16 | 2.71 | 0.0066 | 1.12 | 2.07 | 0.0437 |
| NUSAP1 | 0.56 | 1.75 | 0.16 | 3.56 | 0.0004 | 1.28 | 2.37 | 0.0096 |
| GTSE1 | 0.73 | 2.07 | 0.16 | 4.57 | 0.0000 | 1.51 | 2.82 | 0.0033 |
| PI3 | 0.41 | 1.51 | 0.16 | 2.67 | 0.0076 | 1.12 | 2.05 | 0.0475 |
| SERPINB5 | 0.56 | 1.75 | 0.16 | 3.58 | 0.0003 | 1.29 | 2.37 | 0.0094 |
| PITX3 | 0.78 | 2.17 | 0.16 | 4.88 | 0.0000 | 1.59 | 2.97 | 0.0015 |
| PKP2 | 0.52 | 1.68 | 0.16 | 3.34 | 0.0008 | 1.24 | 2.29 | 0.0131 |
| PLK1 | 0.62 | 1.86 | 0.16 | 3.96 | 0.0001 | 1.37 | 2.54 | 0.0059 |
| ANLN | 0.62 | 1.85 | 0.16 | 3.92 | 0.0001 | 1.36 | 2.52 | 0.0059 |
| PIMREG | 0.47 | 1.60 | 0.16 | 3.02 | 0.0025 | 1.18 | 2.17 | 0.0246 |
| ERCC6L | 0.47 | 1.61 | 0.16 | 3.04 | 0.0023 | 1.18 | 2.18 | 0.0237 |
| ACSM5 | -0.50 | 0.61 | 0.16 | -3.17 | 0.0015 | 0.45 | 0.83 | 0.0180 |
| PARPBP | 0.50 | 1.65 | 0.16 | 3.20 | 0.0014 | 1.21 | 2.23 | 0.0171 |
| CRTAC1 | -0.46 | 0.63 | 0.16 | -2.93 | 0.0033 | 0.46 | 0.86 | 0.0294 |
| CEP55 | 0.43 | 1.54 | 0.16 | 2.75 | 0.0059 | 1.13 | 2.08 | 0.0404 |
| NEIL3 | 0.56 | 1.76 | 0.16 | 3.56 | 0.0004 | 1.29 | 2.40 | 0.0097 |
| HJURP | 0.58 | 1.79 | 0.16 | 3.73 | 0.0002 | 1.32 | 2.44 | 0.0080 |
| FAM72C | 0.66 | 1.94 | 0.16 | 4.20 | 0.0000 | 1.42 | 2.65 | 0.0052 |
| DEPDC1 | 0.43 | 1.54 | 0.16 | 2.76 | 0.0058 | 1.13 | 2.08 | 0.0398 |
| ASF1B | 0.41 | 1.51 | 0.16 | 2.66 | 0.0078 | 1.12 | 2.05 | 0.0477 |
| PRR11 | 0.43 | 1.54 | 0.16 | 2.79 | 0.0053 | 1.14 | 2.09 | 0.0382 |
| DEPDC1B | 0.62 | 1.86 | 0.16 | 3.93 | 0.0001 | 1.37 | 2.54 | 0.0059 |
| PBK | 0.52 | 1.68 | 0.16 | 3.33 | 0.0009 | 1.24 | 2.29 | 0.0131 |
| SUSD2 | -0.43 | 0.65 | 0.15 | -2.81 | 0.0050 | 0.48 | 0.88 | 0.0372 |
| PRMT8 | -0.52 | 0.59 | 0.16 | -3.33 | 0.0009 | 0.44 | 0.81 | 0.0131 |
| ARNTL2 | 0.46 | 1.59 | 0.16 | 2.97 | 0.0030 | 1.17 | 2.16 | 0.0281 |
| SPC25 | 0.51 | 1.66 | 0.16 | 3.24 | 0.0012 | 1.22 | 2.25 | 0.0164 |
| SUGT1P4-STRA6LP-CCDC180 | -0.43 | 0.65 | 0.16 | -2.75 | 0.0060 | 0.48 | 0.88 | 0.0405 |
| RAD51 | 0.42 | 1.52 | 0.16 | 2.70 | 0.0070 | 1.12 | 2.06 | 0.0455 |
| RASGRF1 | -0.46 | 0.63 | 0.16 | -2.94 | 0.0033 | 0.46 | 0.86 | 0.0294 |
| RRM2 | 0.60 | 1.83 | 0.16 | 3.81 | 0.0001 | 1.34 | 2.50 | 0.0068 |
| RS1 | -0.55 | 0.57 | 0.16 | -3.53 | 0.0004 | 0.42 | 0.78 | 0.0098 |
| SCN2A | -0.51 | 0.60 | 0.16 | -3.21 | 0.0013 | 0.44 | 0.82 | 0.0169 |
| CIDEC | 0.41 | 1.50 | 0.15 | 2.64 | 0.0083 | 1.11 | 2.04 | 0.0493 |
| CLSPN | 0.46 | 1.58 | 0.16 | 2.95 | 0.0032 | 1.17 | 2.14 | 0.0292 |
| BLK | -0.42 | 0.65 | 0.16 | -2.73 | 0.0063 | 0.48 | 0.89 | 0.0424 |
| CENPK | 0.55 | 1.74 | 0.16 | 3.53 | 0.0004 | 1.28 | 2.36 | 0.0099 |
| NCAPG | 0.42 | 1.52 | 0.16 | 2.69 | 0.0070 | 1.12 | 2.06 | 0.0455 |
| MS4A4E | -0.64 | 0.53 | 0.16 | -3.99 | 0.0001 | 0.39 | 0.72 | 0.0059 |
| SFTPB | -0.52 | 0.60 | 0.16 | -3.29 | 0.0010 | 0.44 | 0.81 | 0.0147 |
| SFTPD | -0.43 | 0.65 | 0.16 | -2.76 | 0.0057 | 0.48 | 0.88 | 0.0396 |
| MIR924HG | 0.53 | 1.69 | 0.16 | 3.37 | 0.0007 | 1.25 | 2.30 | 0.0127 |
| SLC2A1 | 0.63 | 1.88 | 0.16 | 3.99 | 0.0001 | 1.38 | 2.56 | 0.0059 |
| FAM72B | 0.58 | 1.79 | 0.16 | 3.69 | 0.0002 | 1.31 | 2.43 | 0.0083 |
| SLIT3 | -0.51 | 0.60 | 0.16 | -3.22 | 0.0013 | 0.44 | 0.82 | 0.0167 |
| SPOCK1 | 0.48 | 1.62 | 0.16 | 3.06 | 0.0022 | 1.19 | 2.19 | 0.0227 |
| BUB1 | 0.44 | 1.55 | 0.16 | 2.81 | 0.0049 | 1.14 | 2.10 | 0.0371 |
| BUB1B | 0.58 | 1.79 | 0.16 | 3.72 | 0.0002 | 1.32 | 2.44 | 0.0082 |
| TK1 | 0.56 | 1.74 | 0.16 | 3.54 | 0.0004 | 1.28 | 2.37 | 0.0098 |
| LHFPL3-AS2 | -0.50 | 0.61 | 0.16 | -3.18 | 0.0015 | 0.45 | 0.83 | 0.0180 |
| TTK | 0.54 | 1.71 | 0.16 | 3.45 | 0.0006 | 1.26 | 2.32 | 0.0111 |
| FAM72D | 0.64 | 1.89 | 0.16 | 4.04 | 0.0001 | 1.39 | 2.58 | 0.0059 |
| TYMS | 0.50 | 1.65 | 0.16 | 3.23 | 0.0012 | 1.22 | 2.24 | 0.0166 |
| VGF | 0.45 | 1.57 | 0.16 | 2.92 | 0.0035 | 1.16 | 2.13 | 0.0308 |
| VIPR1 | -0.41 | 0.66 | 0.16 | -2.65 | 0.0081 | 0.49 | 0.90 | 0.0485 |
| CA4 | -0.42 | 0.66 | 0.16 | -2.68 | 0.0073 | 0.48 | 0.89 | 0.0462 |
| IL1R2 | 0.56 | 1.75 | 0.16 | 3.55 | 0.0004 | 1.29 | 2.39 | 0.0097 |
| AUNIP | 0.43 | 1.53 | 0.16 | 2.75 | 0.0060 | 1.13 | 2.08 | 0.0407 |
| CENPM | 0.41 | 1.51 | 0.16 | 2.65 | 0.0081 | 1.11 | 2.06 | 0.0485 |
| DSCC1 | 0.45 | 1.57 | 0.16 | 2.87 | 0.0041 | 1.15 | 2.13 | 0.0333 |
| KREMEN2 | 0.43 | 1.54 | 0.16 | 2.78 | 0.0055 | 1.14 | 2.09 | 0.0387 |
| CENPU | 0.57 | 1.77 | 0.16 | 3.63 | 0.0003 | 1.30 | 2.41 | 0.0089 |
| SHCBP1 | 0.58 | 1.78 | 0.16 | 3.64 | 0.0003 | 1.30 | 2.43 | 0.0087 |
| GREB1L | 0.49 | 1.64 | 0.16 | 3.16 | 0.0016 | 1.21 | 2.23 | 0.0187 |
| FOSL1 | 0.51 | 1.66 | 0.16 | 3.26 | 0.0011 | 1.23 | 2.26 | 0.0159 |
| FAM83D | 0.62 | 1.85 | 0.16 | 3.89 | 0.0001 | 1.36 | 2.52 | 0.0059 |
| DIAPH3 | 0.53 | 1.69 | 0.16 | 3.39 | 0.0007 | 1.25 | 2.30 | 0.0126 |
| KIF18A | 0.59 | 1.80 | 0.16 | 3.76 | 0.0002 | 1.33 | 2.45 | 0.0080 |
| CDC45 | 0.41 | 1.51 | 0.16 | 2.65 | 0.0080 | 1.11 | 2.05 | 0.0481 |
| DRC3 | -0.53 | 0.59 | 0.16 | -3.38 | 0.0007 | 0.43 | 0.80 | 0.0126 |
| CDCA3 | 0.58 | 1.79 | 0.16 | 3.71 | 0.0002 | 1.32 | 2.44 | 0.0082 |
| NUF2 | 0.46 | 1.58 | 0.16 | 2.95 | 0.0032 | 1.17 | 2.15 | 0.0292 |
| KCNK16 | -0.41 | 0.66 | 0.16 | -2.64 | 0.0084 | 0.49 | 0.90 | 0.0493 |
| ESYT3 | -0.53 | 0.59 | 0.16 | -3.39 | 0.0007 | 0.43 | 0.80 | 0.0126 |
| GINS4 | 0.50 | 1.66 | 0.16 | 3.24 | 0.0012 | 1.22 | 2.24 | 0.0164 |
| FIBCD1 | 0.46 | 1.58 | 0.16 | 2.95 | 0.0032 | 1.17 | 2.15 | 0.0292 |
| TNS4 | 0.56 | 1.75 | 0.16 | 3.59 | 0.0003 | 1.29 | 2.38 | 0.0092 |
| ZIC5 | 0.41 | 1.51 | 0.16 | 2.63 | 0.0085 | 1.11 | 2.04 | 0.0493 |
| CCNA2 | 0.63 | 1.88 | 0.16 | 4.00 | 0.0001 | 1.38 | 2.56 | 0.0059 |
| CCNB1 | 0.54 | 1.72 | 0.16 | 3.43 | 0.0006 | 1.26 | 2.33 | 0.0113 |
| CCNE1 | 0.49 | 1.63 | 0.16 | 3.13 | 0.0017 | 1.20 | 2.22 | 0.0193 |
| CDKL2 | -0.43 | 0.65 | 0.16 | -2.77 | 0.0056 | 0.48 | 0.88 | 0.0391 |
| PRC1 | 0.70 | 2.00 | 0.16 | 4.40 | 0.0000 | 1.47 | 2.73 | 0.0036 |
| CCNB2 | 0.52 | 1.69 | 0.16 | 3.35 | 0.0008 | 1.24 | 2.29 | 0.0129 |
| EXO1 | 0.51 | 1.67 | 0.16 | 3.29 | 0.0010 | 1.23 | 2.26 | 0.0147 |
| AURKB | 0.45 | 1.57 | 0.16 | 2.89 | 0.0038 | 1.16 | 2.13 | 0.0325 |
| CACNA2D2 | -0.51 | 0.60 | 0.16 | -3.23 | 0.0013 | 0.44 | 0.82 | 0.0167 |
| TSPOAP1 | -0.50 | 0.60 | 0.16 | -3.18 | 0.0015 | 0.44 | 0.82 | 0.0180 |
| TRIP13 | 0.48 | 1.61 | 0.16 | 3.03 | 0.0024 | 1.18 | 2.19 | 0.0242 |
| ABCC12 | -0.43 | 0.65 | 0.16 | -2.77 | 0.0056 | 0.48 | 0.88 | 0.0391 |
| KIF23 | 0.56 | 1.76 | 0.16 | 3.61 | 0.0003 | 1.29 | 2.39 | 0.0092 |
| TESMIN | 0.67 | 1.96 | 0.16 | 4.28 | 0.0000 | 1.44 | 2.66 | 0.0051 |
| RIMS2 | 0.57 | 1.77 | 0.16 | 3.61 | 0.0003 | 1.30 | 2.42 | 0.0092 |
| ESPL1 | 0.57 | 1.77 | 0.16 | 3.65 | 0.0003 | 1.30 | 2.41 | 0.0087 |
| KIAA0408 | -0.44 | 0.65 | 0.16 | -2.79 | 0.0052 | 0.48 | 0.88 | 0.0382 |
| PCLAF | 0.56 | 1.76 | 0.16 | 3.59 | 0.0003 | 1.29 | 2.39 | 0.0092 |
| DLGAP5 | 0.62 | 1.85 | 0.16 | 3.89 | 0.0001 | 1.36 | 2.52 | 0.0059 |
| ARHGAP11A | 0.54 | 1.71 | 0.16 | 3.45 | 0.0006 | 1.26 | 2.33 | 0.0111 |
| CDK1 | 0.62 | 1.86 | 0.16 | 3.90 | 0.0001 | 1.36 | 2.53 | 0.0059 |
| MELK | 0.47 | 1.60 | 0.16 | 2.99 | 0.0028 | 1.18 | 2.17 | 0.0265 |
| CDC6 | 0.48 | 1.62 | 0.16 | 3.09 | 0.0020 | 1.19 | 2.20 | 0.0215 |
| KIF14 | 0.64 | 1.90 | 0.16 | 4.10 | 0.0000 | 1.40 | 2.59 | 0.0059 |
| CDC25C | 0.59 | 1.80 | 0.16 | 3.74 | 0.0002 | 1.32 | 2.45 | 0.0080 |

Table S8. A total of 117 survival associated DEGs were determined to be bond and regulated by several RNA regulators in publicly available CLIP-seq data in GEO database.

| Gene.Name | GSE146207 | GSE146207 | GSE102336 | GSE98085 | GSE191170 | GSE86214 |
| --- | --- | --- | --- | --- | --- | --- |
| CCNB1 | TRM10A | FTO | ZCCHC4 | YTHDC2 | - | YTHDF3 |
| CCNE1 | TRM10A | - | - | - | - | - |
| CDCA5 | TRM10A | FTO | - | - | - | YTHDF3 |
| CDK1 | TRM10A | - | ZCCHC4 | YTHDC2 | - | YTHDF3 |
| CDKL2 | TRM10A | - | - | YTHDC2 | - | - |
| CENPM | TRM10A | FTO | - | - | - | YTHDF3 |
| CLCN1 | TRM10A | - | - | - | - | - |
| ESPL1 | TRM10A | - | - | YTHDC2 | - | - |
| FAM83D | TRM10A | - | - | - | METTL3 | YTHDF3 |
| HOXB9 | TRM10A | FTO | - | - | - | - |
| KPNA2 | TRM10A | FTO | - | YTHDC2 | - | YTHDF3 |
| LHFPL3-AS2 | TRM10A | - | - | - | - | - |
| MAD2L1 | TRM10A | - | - | - | - | YTHDF3 |
| MKI67 | TRM10A | FTO | - | - | METTL3 | YTHDF3 |
| NUSAP1 | TRM10A | FTO | - | YTHDC2 | METTL3 | YTHDF3 |
| PCLAF | TRM10A | - | - | - | - | - |
| PLK1 | TRM10A | FTO | - | - | METTL3 | YTHDF3 |
| PRC1 | TRM10A | FTO | - | - | METTL3 | YTHDF3 |
| PTPRQ | TRM10A | - | ZCCHC4 | - | - | - |
| RASGRF1 | TRM10A | - | - | - | - | - |
| RRM2 | TRM10A | FTO | - | - | - | YTHDF3 |
| SLC26A5 | TRM10A | - | - | - | - | - |
| SPAG5 | TRM10A | - | - | - | - | YTHDF3 |
| TPX2 | TRM10A | - | ZCCHC4 | - | METTL3 | YTHDF3 |
| UBE2S | TRM10A | - | - | YTHDC2 | METTL3 | YTHDF3 |
| ANLN | - | FTO | - | YTHDC2 | METTL3 | YTHDF3 |
| BIRC5 | - | FTO | - | - | - | YTHDF3 |
| CCNA2 | - | FTO | ZCCHC4 | - | - | YTHDF3 |
| CDC45 | - | FTO | - | YTHDC2 | METTL3 | YTHDF3 |
| CENPU | - | FTO | - | - | METTL3 | - |
| CENPW | - | FTO | - | - | - | - |
| CKS1B | - | FTO | - | - | METTL3 | YTHDF3 |
| EXO1 | - | FTO | - | - | METTL3 | YTHDF3 |
| FOXM1 | - | FTO | ZCCHC4 | - | METTL3 | YTHDF3 |
| HMGA1 | - | FTO | ZCCHC4 | - | - | YTHDF3 |
| IGF2BP3 | - | FTO | - | - | METTL3 | - |
| KIF11 | - | FTO | - | - | METTL3 | YTHDF3 |
| KIF18A | - | FTO | - | - | METTL3 | - |
| KIF23 | - | FTO | - | YTHDC2 | METTL3 | - |
| KIF4A | - | FTO | - | - | - | YTHDF3 |
| MIR23A | - | FTO | - | - | - | - |
| MIR924HG | - | FTO | - | - | - | - |
| MTHFD2 | - | FTO | - | - | METTL3 | - |
| NCAPG | - | FTO | - | YTHDC2 | METTL3 | - |
| NEIL3 | - | FTO | - | - | METTL3 | - |
| PBK | - | FTO | - | - | METTL3 | - |
| RACGAP1 | - | FTO | - | - | METTL3 | - |
| RIMS2 | - | FTO | - | - | - | - |
| SLC2A1 | - | FTO | - | - | METTL3 | YTHDF3 |
| TK1 | - | FTO | ZCCHC4 | - | - | YTHDF3 |
| TRIP13 | - | FTO | - | - | METTL3 | YTHDF3 |
| TYMS | - | FTO | - | - | METTL3 | - |
| ZWINT | - | FTO | - | - | - | YTHDF3 |
| CEP55 | - | - | ZCCHC4 | - | METTL3 | - |
| ABCC12 | - | - | - | YTHDC2 | - | - |
| ARHGAP11A | - | - | - | YTHDC2 | METTL3 | YTHDF3 |
| CDCA2 | - | - | - | YTHDC2 | METTL3 | YTHDF3 |
| CRTAC1 | - | - | - | YTHDC2 | - | - |
| CYP17A1 | - | - | - | YTHDC2 | - | - |
| DNAH1 | - | - | - | YTHDC2 | METTL3 | - |
| ESYT3 | - | - | - | YTHDC2 | - | - |
| GREB1L | - | - | - | YTHDC2 | METTL3 | - |
| HJURP | - | - | - | YTHDC2 | METTL3 | - |
| KIF18B | - | - | - | YTHDC2 | METTL3 | YTHDF3 |
| KIF20A | - | - | - | YTHDC2 | - | YTHDF3 |
| NEK2 | - | - | - | YTHDC2 | - | YTHDF3 |
| NUF2 | - | - | - | YTHDC2 | METTL3 | - |
| PARPBP | - | - | - | YTHDC2 | METTL3 | - |
| SHCBP1 | - | - | - | YTHDC2 | METTL3 | - |
| SKA3 | - | - | - | YTHDC2 | - | - |
| SLC13A5 | - | - | - | YTHDC2 | - | - |
| SLIT3 | - | - | - | YTHDC2 | - | - |
| SNX30 | - | - | - | YTHDC2 | METTL3 | YTHDF3 |
| SPC25 | - | - | - | YTHDC2 | METTL3 | - |
| TTC16 | - | - | - | YTHDC2 | - | - |
| ARNTL2 | - | - | - | - | METTL3 | - |
| BUB1 | - | - | - | - | METTL3 | - |
| BUB1B | - | - | - | - | METTL3 | - |
| C16orf74 | - | - | - | - | METTL3 | YTHDF3 |
| CCNB2 | - | - | - | - | METTL3 | - |
| CDC25C | - | - | - | - | METTL3 | - |
| CDC6 | - | - | - | - | METTL3 | - |
| CENPE | - | - | - | - | METTL3 | - |
| CENPK | - | - | - | - | METTL3 | - |
| CHEK1 | - | - | - | - | METTL3 | - |
| CLSPN | - | - | - | - | METTL3 | YTHDF3 |
| DEPDC1 | - | - | - | - | METTL3 | - |
| DEPDC1B | - | - | - | - | METTL3 | - |
| DIAPH3 | - | - | - | - | METTL3 | - |
| E2F7 | - | - | - | - | METTL3 | YTHDF3 |
| ECT2 | - | - | - | - | METTL3 | - |
| ESCO2 | - | - | - | - | METTL3 | - |
| FAM131C | - | - | - | - | METTL3 | YTHDF3 |
| GTSE1 | - | - | - | - | METTL3 | - |
| KIF14 | - | - | - | - | METTL3 | - |
| MELK | - | - | - | - | METTL3 | YTHDF3 |
| OIP5 | - | - | - | - | METTL3 | YTHDF3 |
| PRMT8 | - | - | - | - | METTL3 | - |
| PRR11 | - | - | - | - | METTL3 | YTHDF3 |
| RAD51 | - | - | - | - | METTL3 | - |
| RAD51AP1 | - | - | - | - | METTL3 | - |
| SH2D5 | - | - | - | - | METTL3 | - |
| SPOCK1 | - | - | - | - | METTL3 | - |
| TTK | - | - | - | - | METTL3 | - |
| ADGRD1 | - | - | - | - | - | YTHDF3 |
| ASF1B | - | - | - | - | - | YTHDF3 |
| CDCA3 | - | - | - | - | - | YTHDF3 |
| FAM72B | - | - | - | - | - | YTHDF3 |
| FAM72C | - | - | - | - | - | YTHDF3 |
| FAM72D | - | - | - | - | - | YTHDF3 |
| FIBCD1 | - | - | - | - | - | YTHDF3 |
| HTR1D | - | - | - | - | - | YTHDF3 |
| KREMEN2 | - | - | - | - | - | YTHDF3 |
| LINC00942 | - | - | - | - | - | YTHDF3 |
| NTSR1 | - | - | - | - | - | YTHDF3 |
| RHOV | - | - | - | - | - | YTHDF3 |
| ZIC5 | - | - | - | - | - | YTHDF3 |

Table S9. Multivariate Cox regression analysis identified five genes which was used to construct RMScore in LUAD.

| Gene.Name | coef | exp(coef) | se(coef) | z | Pr(>\|z\|) | lower .95 | upper .95 |
| --- | --- | --- | --- | --- | --- | --- | --- |
| CYP17A1 | -0.95 | 0.39 | 0.45 | -2.10 | 0.0355 | 0.16 | 0.94 |
| NTSR1 | 0.30 | 1.36 | 0.10 | 3.16 | 0.0016 | 1.12 | 1.64 |
| PITX3 | 1.20 | 3.32 | 0.42 | 2.83 | 0.0046 | 1.45 | 7.60 |
| KRT6A | 0.10 | 1.10 | 0.04 | 2.65 | 0.0081 | 1.03 | 1.18 |
| ANLN | 0.23 | 1.26 | 0.09 | 2.59 | 0.0097 | 1.06 | 1.50 |
