# Supplementary material for: Crosstalk of Eight Types of RNA Modification Regulators Defines Tumor Microenvironments, Cancer Hallmarks, and Prognosis of Lung Adenocarcinoma
Source: J Oncol. 2022 Jul 11;2022:1285632. doi: 10.1155/2022/1285632 (PMC9293558; doi:10.1155/2022/1285632)
Supplement: Supplementary Materials — Supplementary Figure 1. Mutations of eight types of RNA modification regulators in LUAD. Supplementary Figure 2. The prognostic value of eight types of RNA regulators in LUAD. Supplementary Figure 3. Kaplan-Meier curves of RNA regulators in LUAD patients in the GSE50081 dataset. Supplementary Figure 4. Top 10 interactions of RNA modification regulators in LUAD. Supplementary Figure 5. The mutation and expression pattern of RNA regulators in two RNA modification clusters in the TCGA-LUAD cohort. Supplementary Figure 6. Two RNA modification patterns of LUAD in the GSE41271 dataset. Supplementary Figure 7. RNA modification patterns of BRCA and COAD patients in the TCGA datasets. Supplementary Figure 8. The dysregulation and prognostic value of five RMScore-related genes in LUAD. Supplementary Table 1. Clinical information of LUAD patients in TCGA and GEO datasets. Supplementary Table 2. 100 RNA regulators of eight types of RNA modifications included in this study. Supplementary Table 3. Clinical information of 56 tumor and paired normal tissues in the TCGA-LUAD cohort. Supplementary Table 4. Multivariate Cox regression analysis of RNA regulators in TCGA-LUAD cohort. Supplementary Table 5. Correlations among eight types of RNA regulators in LUAD. Supplementary Table 6. The RNA modification pattern and RMScore of LUAD patients. Supplementary Table 7. Survival associated DEGs in univariate Cox regression analysis in TCGA-LUAD cohort. Supplementary Table 8. A total of 117 survival-associated DEGs were determined to be bonded and regulated by several RNA regulators in publicly available CLIP-seq data in the GEO database. Supplementary Table 9. Multivariate Cox regression analysis identified five genes that were used to construct the RMScore in LUAD. [file 1285632.f1.zip › 1285632.f1/Supplementary_figures_R1.docx]

Supplementary Figures

## Supplementary Figure 1

**
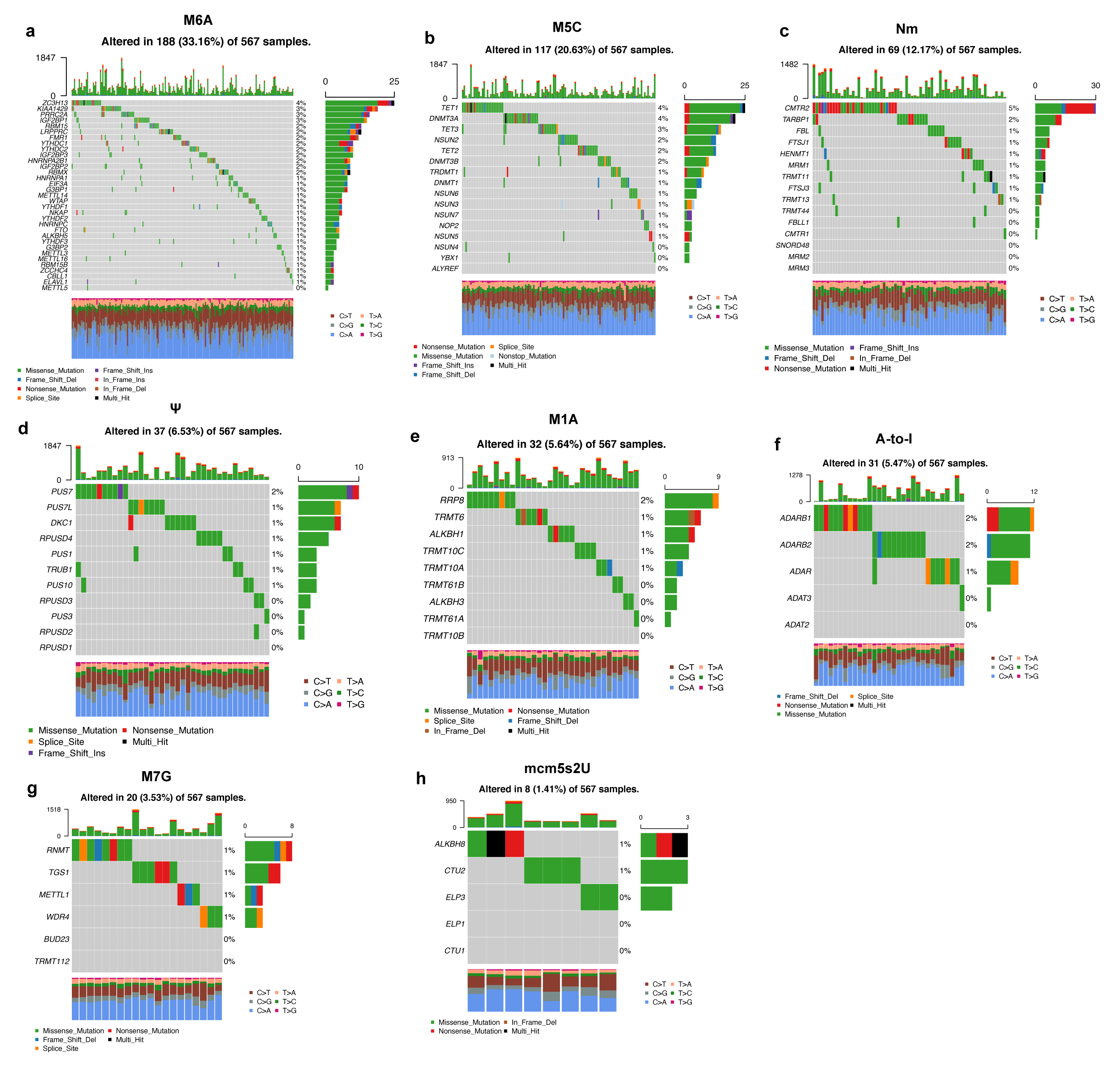
**

**Supplementary Figure 1.** **Mutations of eight types of RNA modification regulators in LUAD.** A-H. The mutation spectrum of RNA regulators of m^6^A (A), m^5^C (B), Nm (C), Ψ (D), m^1^A (E), A-to-I (F), m^7^G (G) and mcm^5^s^2^U (H) in TCGA-LUAD cohort, with each column representing one patient and the percentage on the right side representing the corresponding gene mutation rate.

## Supplementary Figure 2

**
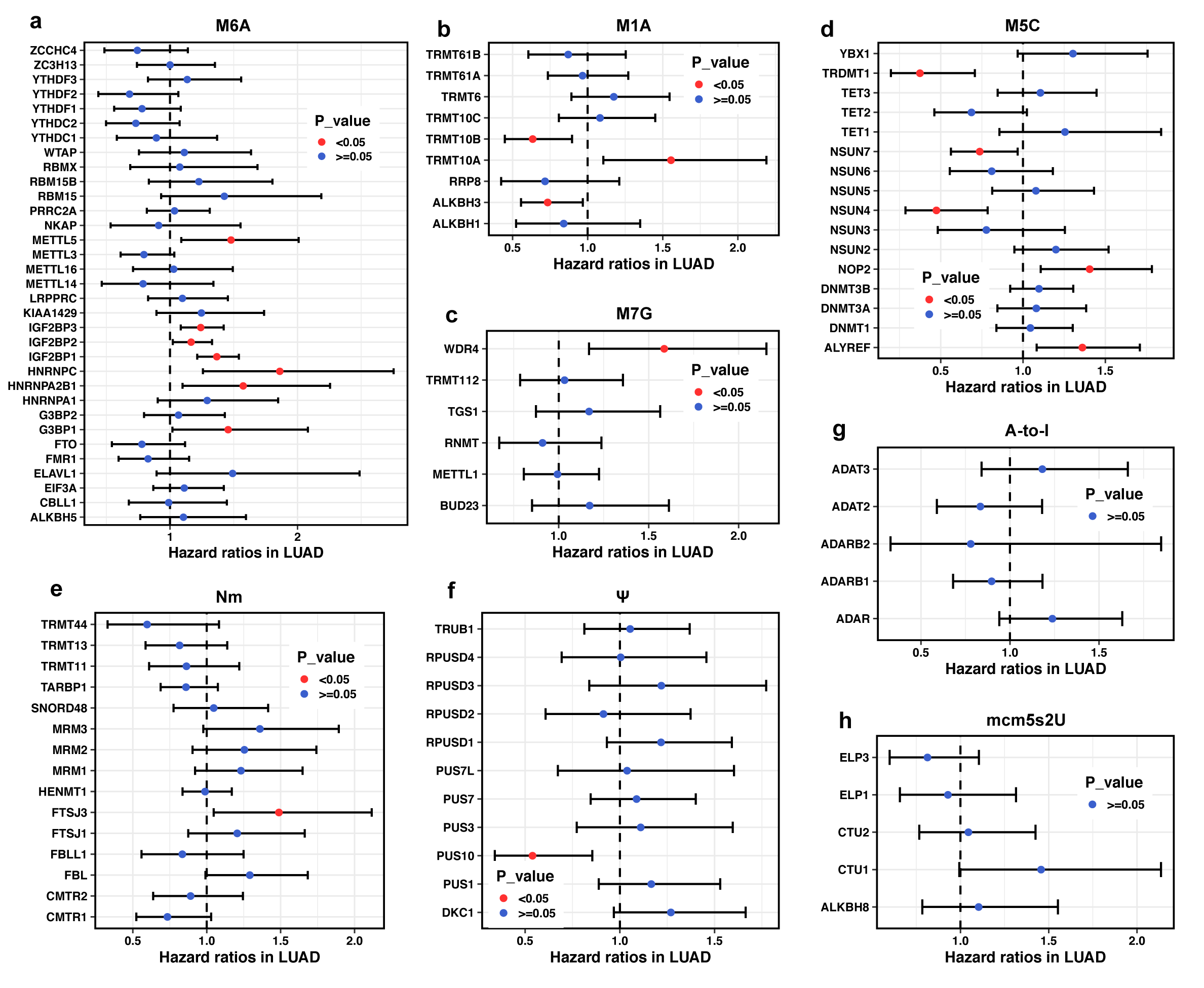
**

**Supplementary Figure 2.** **The prognostic value of eight types of RNA regulators in LUAD.** A-H. The forest plot showed the hazard ratios (HRs) of RNA regulators of m^6^A (A), m^1^A (B), m^7^G (C), m^5^C (D), Nm (E), Ψ (F), A-to-I (G) and mcm^5^s^2^U (H) in univariate cox regression analysis in TCGA-LUAD cohort. The dotted horizontal line represents the hazard ratios and the 95% confidence interval of each gene, with the color of dot point representing the statical significance.

## Supplementary Figure 3


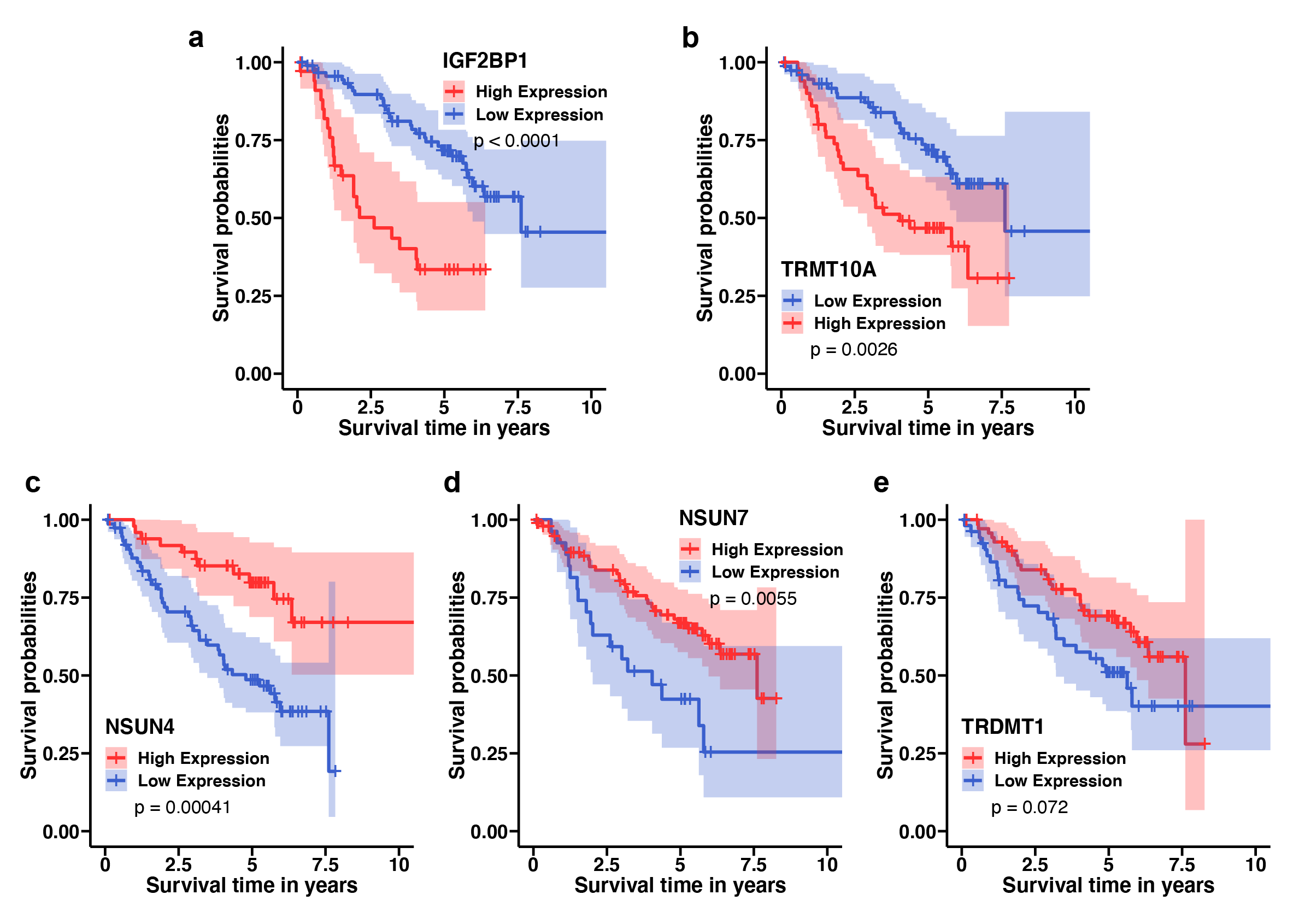


**Supplementary Figure 3.** **Kaplan-Meier curves of RNA regulators in LUAD patients in GSE50081 dataset.** A. Kaplan-Meier curves showed the overall survival of LUAD patients in IGF2BP1-low group was longer than whom in IGF2BP1-high group in GSE50081 datasets. B. Kaplan-Meier curves showed the overall survival of LUAD patients in TRMT10A-low group was longer than whom in TRMT10A-high group in GSE50081 dataset. C. Kaplan-Meier curves showed the overall survival of LUAD patients in NSUN4-low group was shorter than whom in NSUN4-high group in GSE50081 dataset. D. Kaplan-Meier curves showed the overall survival of LUAD patients in NSUN7-low group was shorter than whom in NSUN7-high group in GSE50081 dataset. E. Kaplan-Meier curves showed the overall survival of LUAD patients in TRDMT1-low group was shorter than whom in TRDMT1-high group in GSE50081 dataset.

## Supplementary Figure 4

**
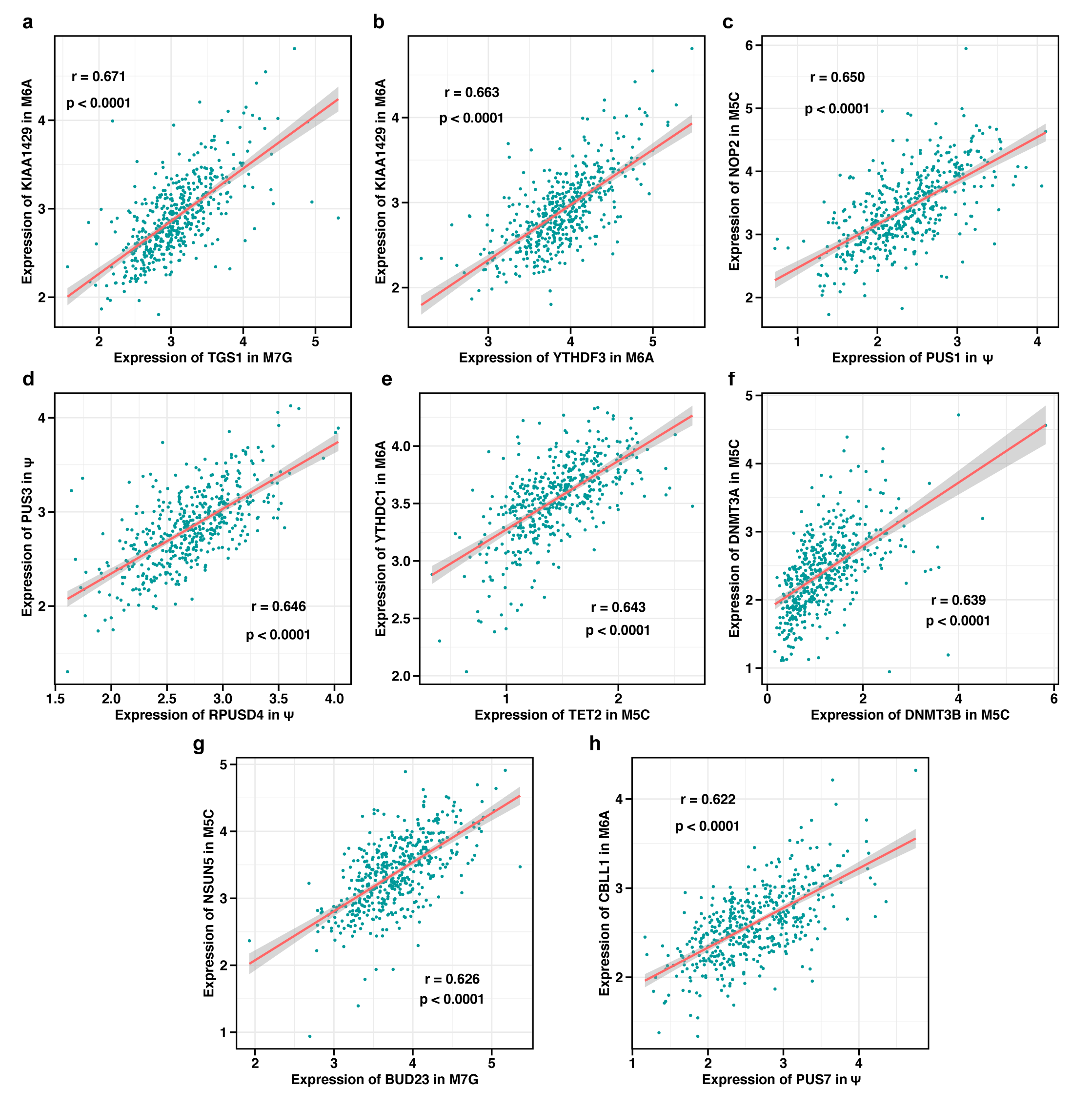
**

**Supplementary Figure 4.** **Top 10 interactions of RNA modification regulators in LUAD.** A. The level of KIAA1429 of m^6^A was positively correlated with the level of TGS1 of m^7^G in LUAD. B. The level of KIAA1429 of m^6^A was positively correlated with the level of YTHDF3 of m^6^A in LUAD. C. The level of NOP2 of m^5^C was positively correlated with the level of PUS1 of Ψ in LUAD. D. The level of PUS3 of Ψ was positively correlated with the level of RPUSD4 of Ψ in LUAD. E. The level of YTHDC1 of m^6^A was positively correlated with the level of TET2 of m^5^C in LUAD. F. The level of DNMT3A of m^5^C was positively correlated with the level of DNMT3B of m^5^C in LUAD. G. The level of NSUN5 of m^5^C was positively correlated with the level of BUD23 of m^7^G in LUAD. H. The level of CBLL1 of m^6^A was positively correlated with the level of PUS7 of Ψ in LUAD.

## Supplementary Figure 5


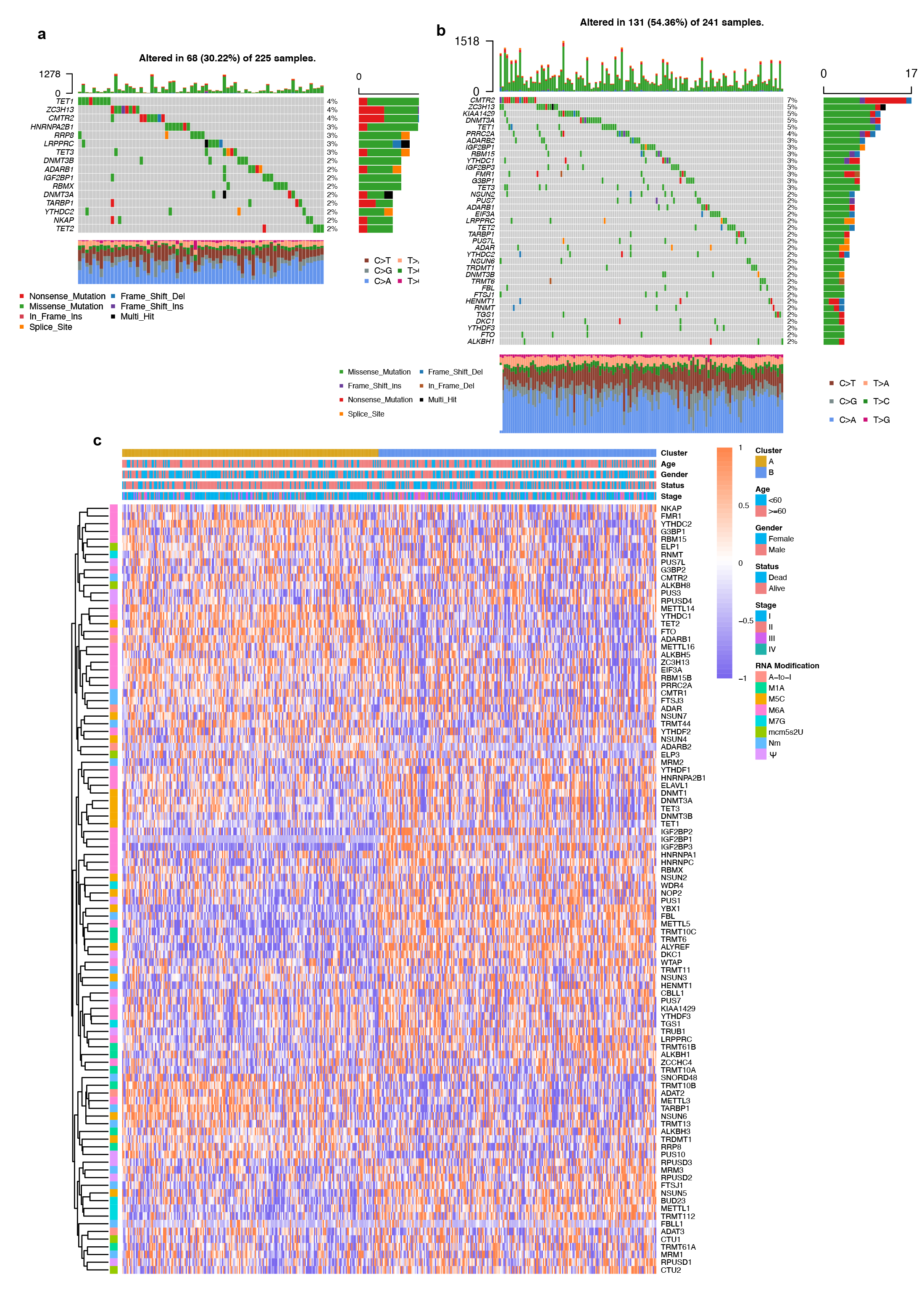


**Supplementary Figure 5. The mutation and expression pattern of RNA regulators in two RNA modification clusters in TCGA-LUAD cohort.** A-B. The mutation spectrum of RNA regulators with mutation rates ≥ 2% in Cluster A (A) and Cluster B (B), with each column representing one patient and the percentage on the right side representing the corresponding gene mutation rate. C. The heatmap showed the expression of 100 RNA regulators and clinical characteristics of each LUAD patient in two RNA modification clusters.

## Supplementary Figure 6


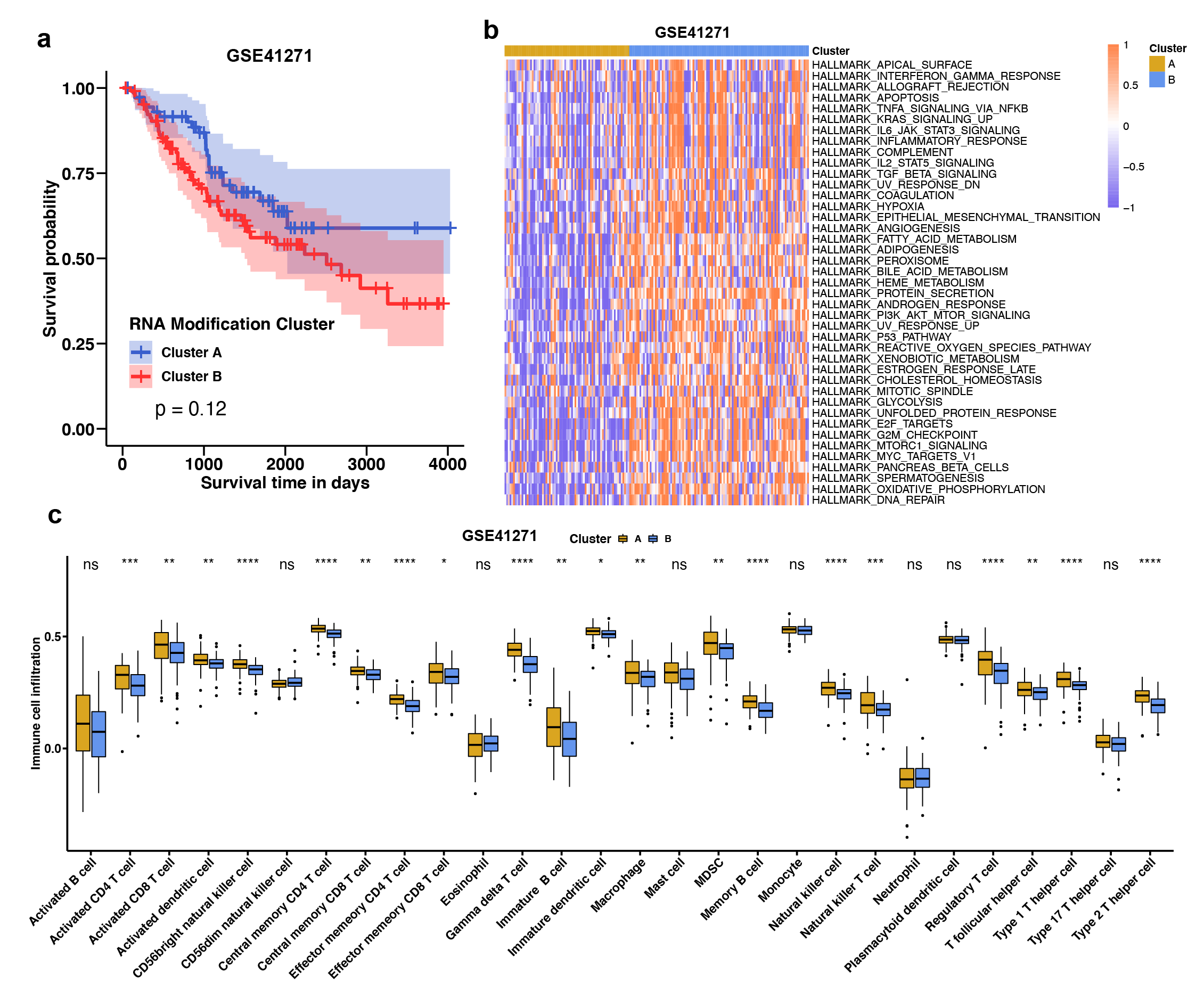


**Supplementary Figure 6. Two RNA modification patterns of LUAD in GSE41271 dataset.** A. Kaplan-Meier curves showed the different overall survival of LUAD patients in two RNA modification clusters in GSE41271 dataset. B. The heatmap revealed that most cancer hallmarks were enriched in LUAD patients in RNA modification cluster B in GSE41271 dataset. C. The box plot showed that immune cells infiltrated in tumor microenvironments were distinct in two RNA modification clusters in GSE41271 dataset.

## Supplementary Figure 7


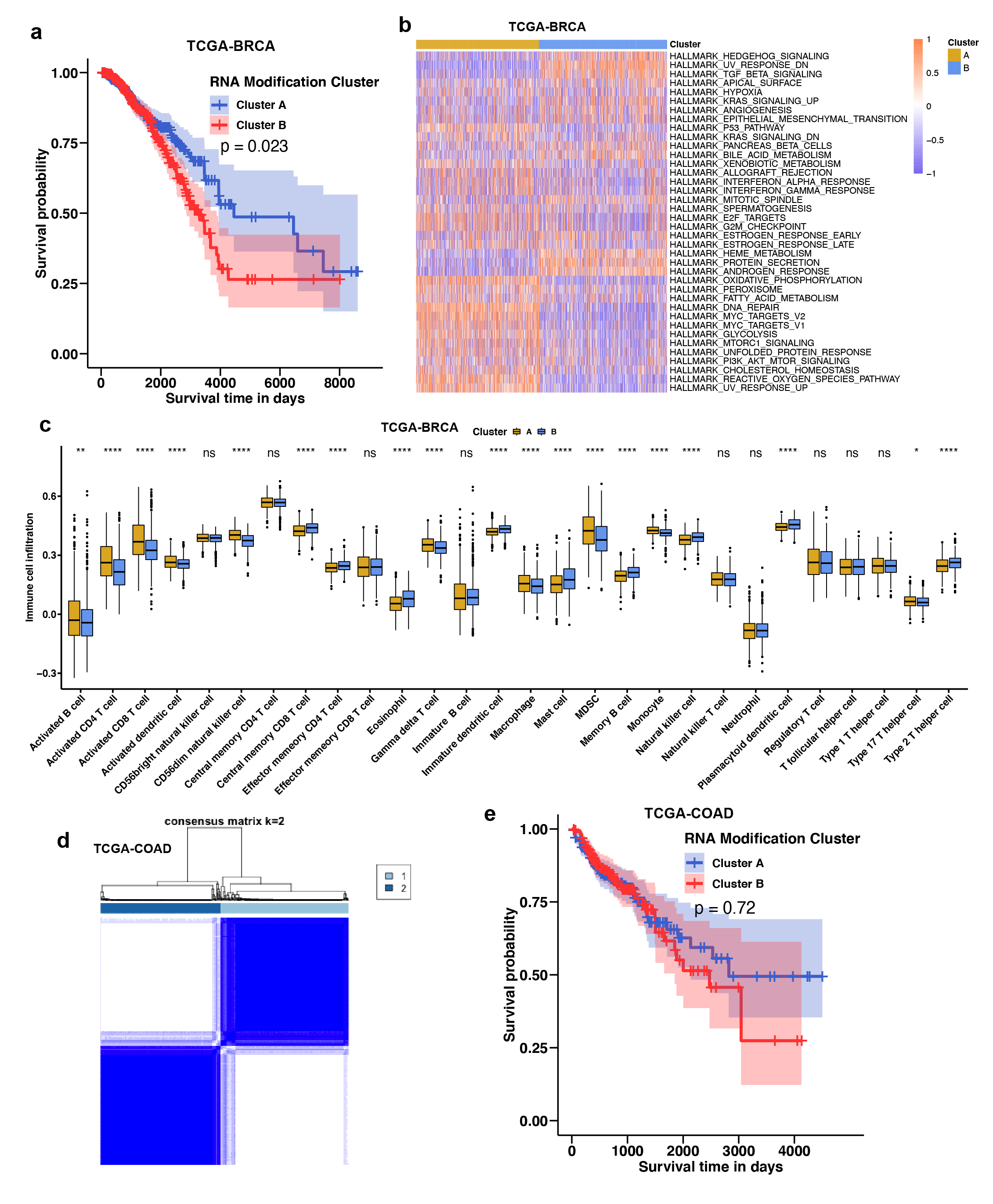


**Supplementary Figure 7. RNA modification patterns of BRCA and COAD patients in TCGA datasets.** A. Kaplan-Meier curves showed the different overall survival of BRCA patients in two RNA modification clusters in TCGA dataset. B. The heatmap revealed that different cancer hallmarks were enriched in different RNA modification clusters in TCGA-BRCA cohort. C. The box plot showed that immune cells infiltrated in tumor microenvironments were distinct in two RNA modification clusters in TCGA-BRCA cohort. D. Patients in TCGA-COAD cohort were divided into two clusters basing on the expression of 100 RNA regulators. E. Kaplan-Meier curves showed that the overall survival of COAD patients in two clusters were not statistically significant. BRCA, breast cancer; COAD, colon cancer.

## Supplementary Figure 8


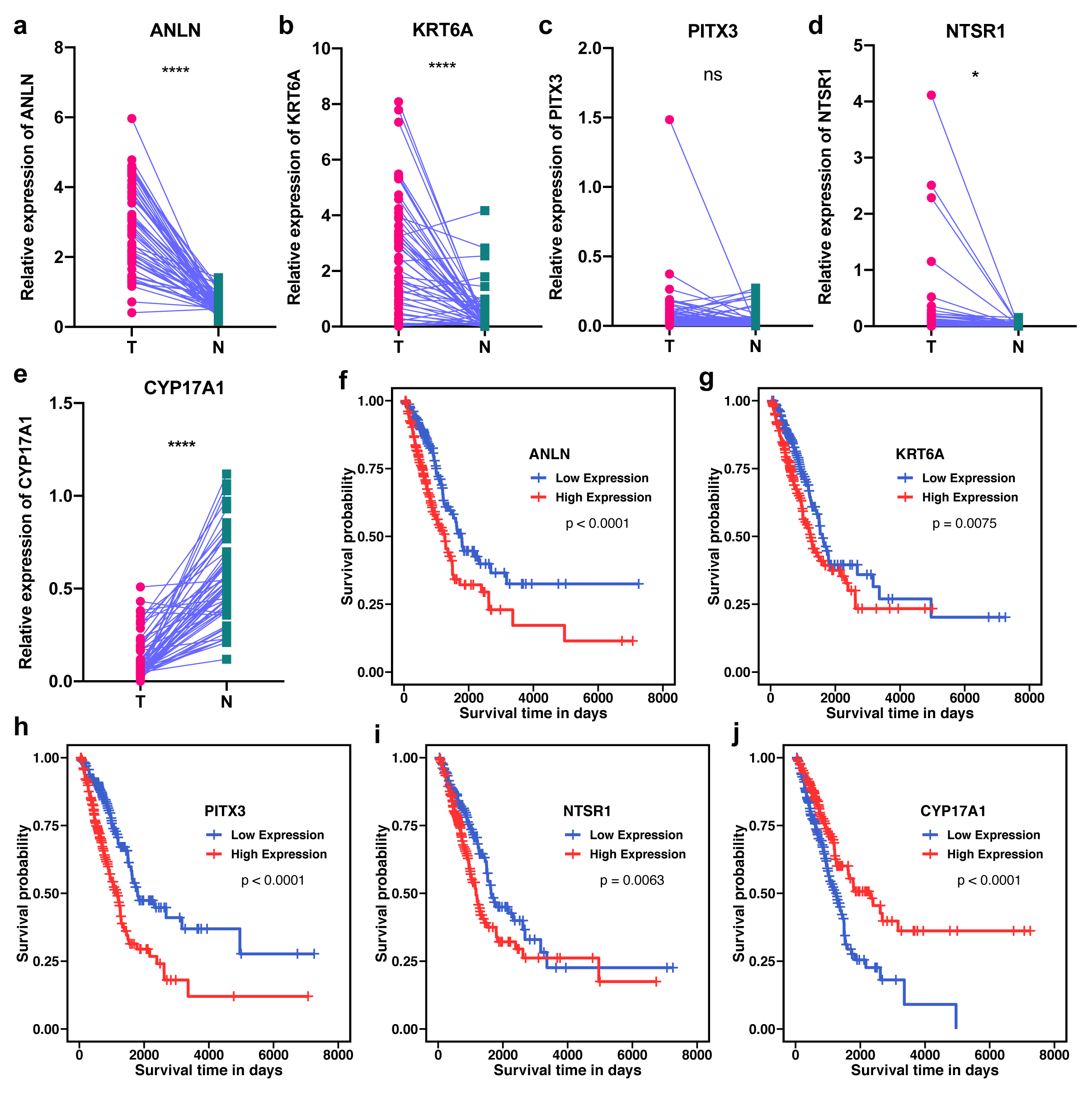


**Supplementary Figure 8.** **The dysregulation and prognostic value of five RMScore-related genes in LUAD.**A-E. The relative expressions of five RMScore-related genes including ANLN (A), KRT6A (B), PITX3 (C), NTSR1 (D) and CYP17A1 (E) in TCGA-LUAD tumor and paired normal lung tissues. F-J. Kaplan-Meier curves showed the overall survival of LUAD patients with low- or high-ANLN (F), KRT6A (G), PITX3 (H), NTSR1 (I) and CYP17A1 (J) expressions.
